# A novel co-target of ACY1 governing plasma membrane translocation of SphK1 contributes to inflammatory and neuropathic pain

**DOI:** 10.1016/j.isci.2023.106989

**Published:** 2023-05-28

**Authors:** Baowen Liu, Wenyao Wu, LingLing Cui, Xuemei Zheng, Ningbo Li, Xianwei Zhang, Guangyou Duan

**Affiliations:** 1 Department of Anesthesiology, The Second Affiliated Hospital, Chongqing Medical University, Chongqing, China; 2 Department of Anesthesiology, Hubei Key Laboratory of Geriatric Anesthesia and Perioperative Brain Health, and Wuhan Clinical Research Center for Geriatric Anesthesia, Tongji Hospital, Tongji Medical College, Huazhong University of Science and Technology, Wuhan, China; 3 Department of Anesthesiology, The Central Hospital of Wuhan, Tongji Medical College, Huazhong University of Science and Technology, Wuhan, China; 4 Department of Anesthesiology, Wuhan third Hospital/Tongren Hospital of Wuhan University, Wuhan, Hubei Province, China

**Keywords:** Biological sciences, Biochemistry, Physiology

## Abstract

Previous studies validate that inhibiting sodium channel 1.8 (Nav1.8) effectively relieves inflammatory and neuropathic pain. However, Nav1.8 blockers have cardiac side effects in addition to analgesic effects. Here, we constructed a spinal differential protein expression profile using Nav1.8 knockout mice to screen common downstream proteins of Nav1.8 in inflammatory and neuropathic pain. We found that aminoacylase 1 (ACY1) expression was increased in wild-type mice compared to Nav1.8 knockout mice in both pain models. Moreover, spinal ACY1 overexpression induced mechanical allodynia in naive mice, while ACY1 suppression alleviated inflammatory and neuropathic pain. Further, ACY1 could interact with sphingosine kinase 1 and promote its membrane translocation, resulting in sphingosine-1-phosphate upregulation and the activation of glutamatergic neurons and astrocytes. In conclusion, ACY1 acts as a common downstream effector protein of Nav1.8 in inflammatory and neuropathic pain and could be a new and precise therapeutic target for chronic pain.

## Highlights

• Constructed spinal differential protein expression profile with Nav1.8 knockout mice
• Screened common downstream proteins of Nav1.8 in inflammatory & neuropathic pain
• ACY1 acted as a common downstream effector protein of Nav1.8 in both pain types
• This could be a new and precise therapeutic target for chronic pain

## Graphical abstract

**Figure:**
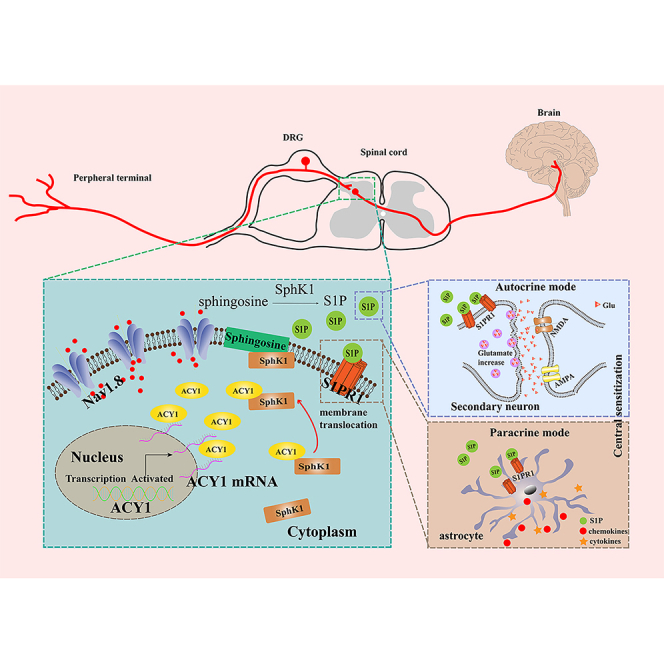

## Introduction

According to the latest epidemiological data, chronic pain severely affects the quality of life of > 30% of the world’s population.^1^ However, treatment of chronic pain remains a challenge. It is well known that inflammatory pain and neuropathic pain are the two common types of chronic pain. Despite the differences in etiology and associated mechanisms between inflammatory and neuropathic pain, each pain state shares common mechanisms.^2^^,^^3^ To date, pharmacological strategies for the treatment of these two types of chronic pain are currently diverse. However, there is still a lack of drugs that can effectively treat both inflammatory pain and neuropathic pain with few adverse reactions. Recently, emerging evidence suggests common pathways, such as common genetic factors, activation of glial cell, and enhanced glutamate release in inflammatory and neuropathic pain.^2^^,^^4^ However, the shared mechanism underlying inflammatory and neuropathic pain remains largely unknown. Therefore, exploring the common target mechanism of inflammatory and neuropathic pain is of great clinical significance for the treatment of chronic pain.

Tetrodotoxin-resistant sodium channel 1.8 (Nav1.8) has attracted strong interest due to the increasing evidence of its effect in human pain disorders.^5^^,^^6^ Abnormal activation of Nav1.8 is associated with a variety of pain conditions including inflammatory pain, neuropathic pain, visceral pain, and cancer pain.^7^^,^^8^^,^^9^^,^^10^ Specifically, Nav1.8 is associated with inflammatory and neuropathic pain states.^11^^,^^12^ Potent and selective blockers of Nav1.8 have been explored and are effective in animal models, providing preclinical evidence for targeting Nav1.8 *in vivo*.^13^^,^^14^^,^^15^ Targeted inhibition of Nav1.8 effectively relieves chronic inflammatory and neuropathic pain.^11^^,^^15^^,^^16^ However, with the increased knowledge on Nav1.8, new questions emerge regarding the role of Nav1.8 in human diseases. Recent evidence associates SCN10A variants with cardiac rhythm disorders, autism spectrum disorder, and Pitt–Hopkins syndrome.^17^^,^^18^^,^^19^^,^^20^^,^^21^ Notably, despite highly selective Nav1.8 blockers, cardiac side effects occur in addition to analgesic effects (10).^22^ For example, A-803467, a selective blocker of Nav1.8, may interact with cardiac electrophysiology in intracardiac neurons and ventricular cardiomyocytes.^23^^,^^24^ Although Nav1.8 targeted inhibition of inflammatory and neuropathic pain might be an effective strategy, its adverse effects on other systems, especially cardiac innervation, may occur. Therefore, exploring the common downstream mechanisms of Nav1.8-mediated inflammatory and neuropathic pain might provide a more precise target for alleviating chronic pain.

Hyperexcitatory and hypoexcitatory neurons depend on the presence or absence of Nav1.8, which plays a critical role in both the inflammatory and neuropathic pain states.^25^^,^^26^ Therefore, we believe that the synchronous role of Nav1.8 in the two types of pain provides the possibility to explore common pathways and downstream mechanisms. In this study, we analyzed the differential protein expression profiles in the spinal cord of normal and Nav1.8 knockout mice by proteomics analysis after complete Freund adjuvant (CFA) and spared nerve injury (SNI) induced inflammatory and neuropathic pain models. Based on the differential protein expression profiles after the two types of pain models, this research also screened the critical candidates for exploring the common downstream mechanism underlying inflammatory and neuropathic pain. Thus, our findings may provide new insights into inflammatory and neuropathic pain from the common downstream mechanisms of Nav1.8 and provide more precise therapeutic targets for chronic pain.

## Results

### Screening downstream differential proteins of Nav1.8 in chronic pain by proteomics

**Figure 1:**
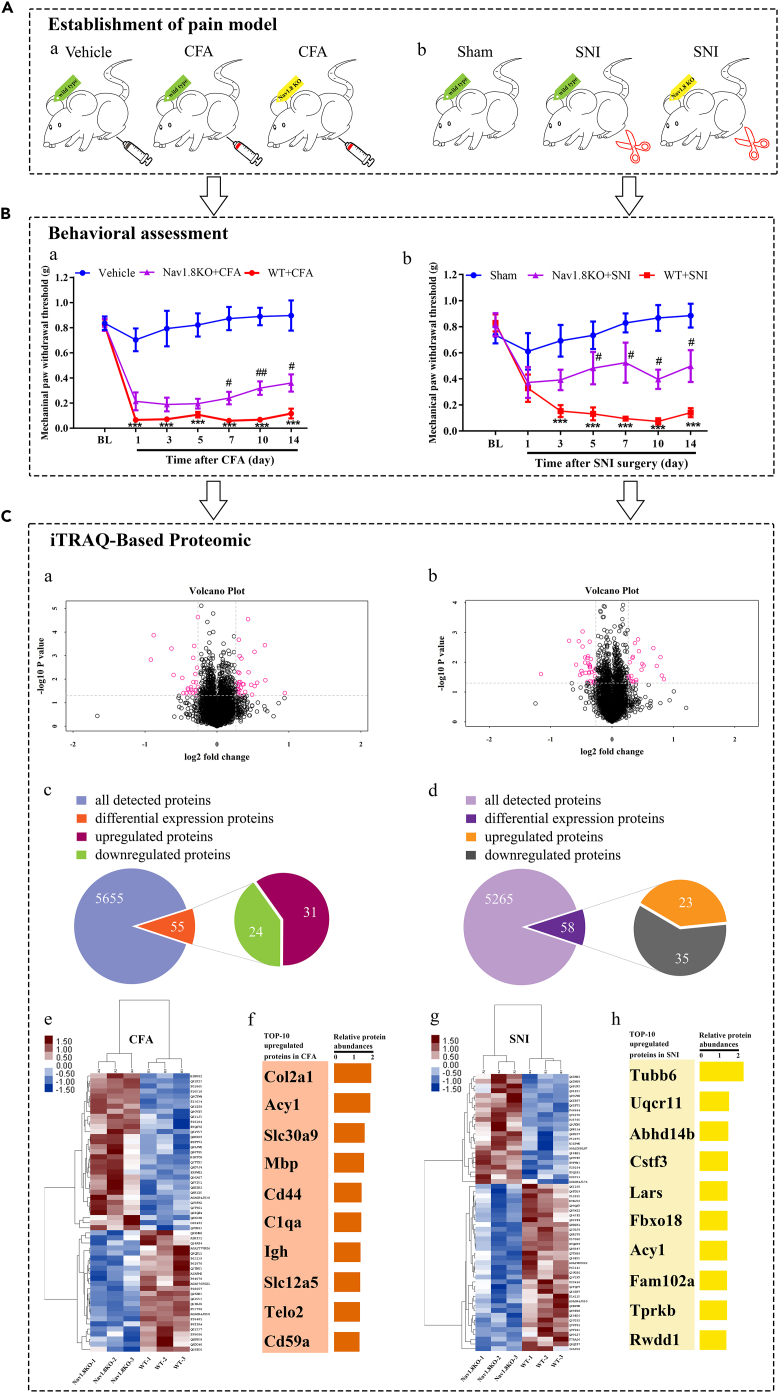
Experimental workflow (A) Representation of the six behavioral groups used in this study. (a) Vehicle and complete Freund adjuvant (CFA) were injected into the left paw of wild-type (WT) mice and TTX-resistant sodium channel 1.8 (Nav1.8) knockout (KO) mice. (b) Sham operation and spared nerve injury (SNI) surgery were performed on WT mice and Nav1.8 KO mice. (B) Behavioral assessment: (a) intraplanar CFA injection induced mechanical allodynia in WT mice. The paw withdrawal threshold of Nav1.8 KO mice was significantly recovered in the later stage. ^∗∗∗^p < 0.001 versus (vs.) the vehicle group; ^#^p < 0.05, ^##^p < 0.01 vs. the WT + CFA group; n = 6–8 mice per group; (b) SNI surgery induced mechanical allodynia in WT mice. The paw withdrawal threshold of Nav1.8 KO mice is significantly recovered in the later stage. ^∗∗∗^p < 0.001 vs. the sham group; ^#^p < 0.05 vs. the WT + SNI group; n = 6–8 mice per group. (C) Isobaric tags for relative and absolute quantitation (iTRAQ)-based proteomic analysis: (a–b) Volcano plot for all proteins profiled in the CFA and SNI models (WT/Nav1.8 KO). (c–d) The number of all detected proteins and differentially expressed proteins in the CFA and SNI models. (e) Heatmap of 55 differentially expressed proteins in the CFA model (WT/Nav1.8 KO; n = 3 per group). As shown in the legend at the top left, blue to red colors in the heatmap represent the abundance of proteins. (f) Top 10 upregulated proteins in the CFA model. (g) Heatmap of 58 differentially expressed proteins in the SNI model (WT/Nav1.8KO; n = 3 per group). As shown in the legend at the top left, blue to red colors in the heatmap represent the abundance of proteins. (h) Top 10 upregulated proteins in the SNI model.

As shown in Figure 1B, the baseline PWTs did not differ significantly between wild-type and Nav1.8 knockout mice. After establishing the inflammatory and neuropathic pain models, PWTs were significantly decreased in wild-type mice. Additionally, the decrease in PWTs in Nav1.8 knockout mice was small in both chronic pain conditions and showed a tendency to recover from mechanical allodynia.

Our proteomics results showed that 5655 and 5265 proteins were detected in the CFA-induced inflammatory pain models and SNI-induced neuropathic pain models, respectively (Figure 1C). The 55 and 58 proteins in inflammatory and neuropathic pain, respectively, showed significantly differential expression profiles in each pain paradigm compared with Nav1.8 knockout mice, and the abundances of 31 and 24 proteins in the spinal cord were clearly elevated in wild-type mice after CFA injection and SNI surgery, respectively. The abundances of 23 and 35 proteins in the spinal cord were dramatically downregulated in wild-type mice after CFA injection and SNI surgery, respectively. Thus, < 1% of all quantified proteins presented remarkable upregulation and downregulation in each pain model, indicating high specificity of the pain model. Therefore, these differentially expressed proteins may be the downstream effector molecules of Nav1.8 in the different pain models when Nav1.8 gene knockout mice were used as controls in the proteomic analysis. Raw proteomics data were shown in Data S1.

### Expression of ACY1 in the spinal cord was upregulated in both chronic pain models

**Figure 2:**
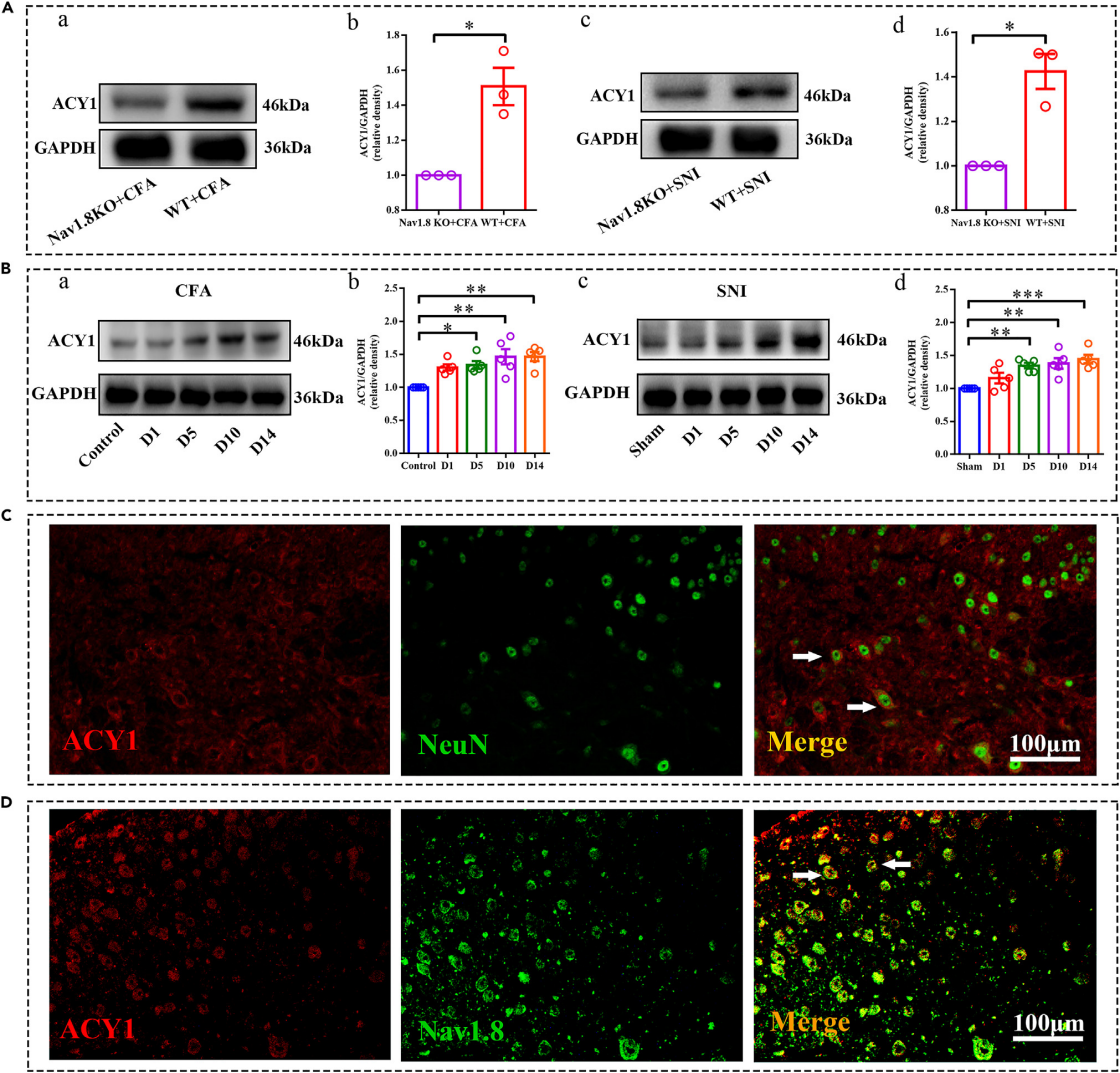
Expression and cellular localization of aminoacylase 1 (ACY1) in the spinal dorsal horn (A) Western blot analysis validates the expression of ACY1 in proteomic samples. Tissues were collected 14 days after CFA injection or SNI surgery (^∗^p < 0.05, n = 3 mice per group). (B) Western blot analysis shows the time course of ACY1 expression in wild-type (WT) mice after complete Freund adjuvant (CFA) injection or spared nerve injury (SNI) surgery (^∗^p < 0.05, ^∗∗^p < 0.01, ^∗∗∗^p < 0.001, n = 5 mice per group). (C) Immunofluorescence double staining of ACY1 with the neuronal marker NeuN in the spinal cord. (D) Representative photomicrographs of ACY1 double fluorescence labeling with Nav1.8 in the spinal cord. The scale bars represent 100 μm. Arrows point to examples of co-labeled neurons. KO, knockout; D, day.

As shown in Figure 1C, the abundance of ACY1 was significantly altered in both pain models among all differentially expressed proteins, according to our proteomic results. We also confirmed the expression level of ACY1 in the original protein lysis buffer by Western blot analysis. As shown in Figure 2A, the abundance of ACY1 in the spinal cord was significantly upregulated in both CFA and SNI-induced chronic pain models in wild-type mice compared with Nav1.8 knockout mice. The verified results are consistent with the trend of the proteomics results, indicating that the proteomics results have high credibility. To clarify the role of ACY1 in chronic pain, we reconstructed two chronic pain models in wild-type mice and detected the expression of ACY1 at different time points. As shown in Figure 2B, we observed a significant upregulation of ACY1 in the spinal cord from day 5–14 after CFA injection and SNI surgery. These results indicate that ACY1 may be an important downstream candidate of Nav1.8 in chronic inflammatory and neuropathic pain. First, we examined the cellular localization of ACY1 in the spinal dorsal horn. We conducted double immunofluorescence of ACY1 with the neuronal marker NeuN, astrocyte marker GFAP, and microglial marker IBA1 to determine the localization of ACY1 in the spinal dorsal horn. As shown in Figure 2C, ACY1 was mainly colocalized with neuronal marker NeuN and sparsely colocalized with GFAP or IBA1 in the dorsal horn of the spinal cord (Figure S1). We then performed double immunofluorescence staining of ACY1 with Nav1.8. As shown in Figure 2D, ACY1 colocalized with Nav1.8, suggesting that ACY1 was expressed in Nav1.8-positive cells.

### Overexpression of spinal ACY1-induced mechanical allodynia and neuronal hyperexcitability in naive mice

**Figure 3:**
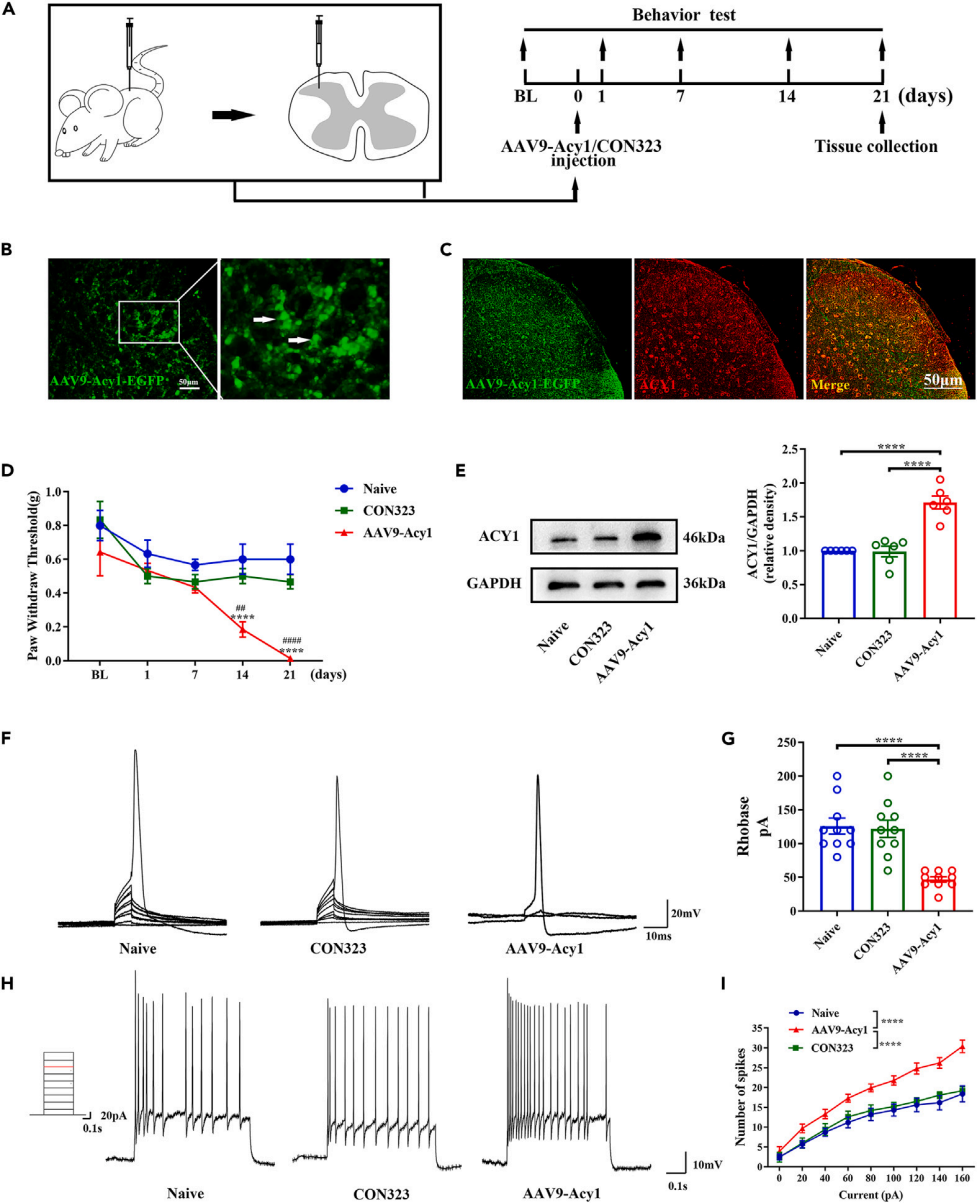
Specific upregulation of aminoacylase 1 (ACY1) alters the mechanical thresholds and excitability of spinal neurons in mice (A) Schematic diagram of the AAV9-Acy1 injection and behavioral tests. (B) Spinal cord section showing significant green fluorescence in the spinal dorsal horn at 21 days after the AAV9-Acy1 injection. (C) Representative photomicrographs of AAV9-Acy1-EGFP (green) double fluorescence labeling with anti-ACY1 (red) in the spinal dorsal horn at 21 days after the AAV9-Acy1 injection (scale bars: 50 μm). (D) Intradorsal horn injection of AAV9-Acy1 markedly decreases the mechanical thresholds (^∗∗∗∗^p < 0.0001 vs. naive, ^##^p < 0.01 vs. CON323, ^####^p < 0.0001 vs. CON323, n = 6). (E) Intradorsal horn injection of AAV9-Acy1 significantly increases the expression of ACY1 in the spinal cord (^∗∗∗∗^p < 0.0001, n = 6). (F) Representative graphs of the rheobase in naive mice, CON323 mice, and mice with ACY1 overexpression. (G) The rheobase is decreased in mice with ACY1 overexpression but not in CON323 mice compared with naive mice (^∗∗∗∗^p < 0.0001, one-way analysis of variance [ANOVA] with the Tukey multiple comparison test; n = 10 neurons/4 mice in each group). (H) Representative traces of the action potentials with a 120-pA current injection in each group. (I) The action potential frequency is increased in mice with ACY1 overexpression but not in CON323 mice compared with naive mice (^∗∗∗∗^p < 0.0001, two-way ANOVA with the Bonferroni post hoc test; n = 10 neurons/4 mice in each group).

To assess the role of ACY1 in pain modulation, we constructed AAV9-Acy1 to specifically upregulate the expression of ACY1 and detect the mechanical thresholds of naive mice following intradorsal horn injection of AAV9-Acy1 versus the vehicle. The recombinant AAV9-Acy1 was fused to enhanced green fluorescent protein (EGFP) C-terminus to provide a fluorescent tag to identify neurons expressing ACY1. As shown in Figure 3B, immunohistochemical characterization of the spinal dorsal horn 3 weeks after injection demonstrated successful transduction of AAV9-Acy1. Western blot and immunofluorescence analyses were performed 21 days after injection to validate successful transduction of the spinal cord. As shown in Figure 3C, EGFP provides a fluorescent tag for identifying neurons expressing the ACY1 peptide aptamer (hereafter referred to as EGFP-ACY1). In addition, results of Western blot analysis showed that the protein expression of ACY1 in the spinal cord was markedly increased 21 days after AAV9-Acy1 injection, suggesting successful virus transfection (Figure 3E). As shown in Figure 3D, the baseline mechanical thresholds were not statistically different between the vehicle and AAV9-Acy1 groups. However, the mechanical thresholds significantly declined from 14 to 21 days after AAV9-Acy1 injection. These data indicate that overexpression of spinal ACY1 is sufficient to contribute to mechanical allodynia in normal animals.

To determine the role of ACY1 in regulating the excitability of neurons in mice spinal cord, we tested the activity of spinal neurons in lamina II 21 days after treatment with an AAV9-Acy1 intradorsal horn injection using whole-cell patch-clamp recordings. As shown in Figures 3F and 3G, rheobase was significantly decreased in mice with spinal ACY1 overexpression compared with naive mice and CON323 mice, suggesting that neurons in the spinal cord more easily initiated an action potential after the AAV9-Acy1 injection. Additionally, as shown in Figures 3H and 3I, spinal ACY1 overexpression significantly increased the number of APs. Notably, rheobase and the AP frequency did not differ significantly between naive mice and CON323 mice, indicating that adeno-associated viruse (AAV) did not affect the excitability of spinal neurons. These data indicate that the overexpression of spinal ACY1 increases the activity of spinal dorsal horn neurons.

### Suppressing spinal ACY1 alleviated CFA-induced inflammatory and spinal hyperexcitability

**Figure 4:**
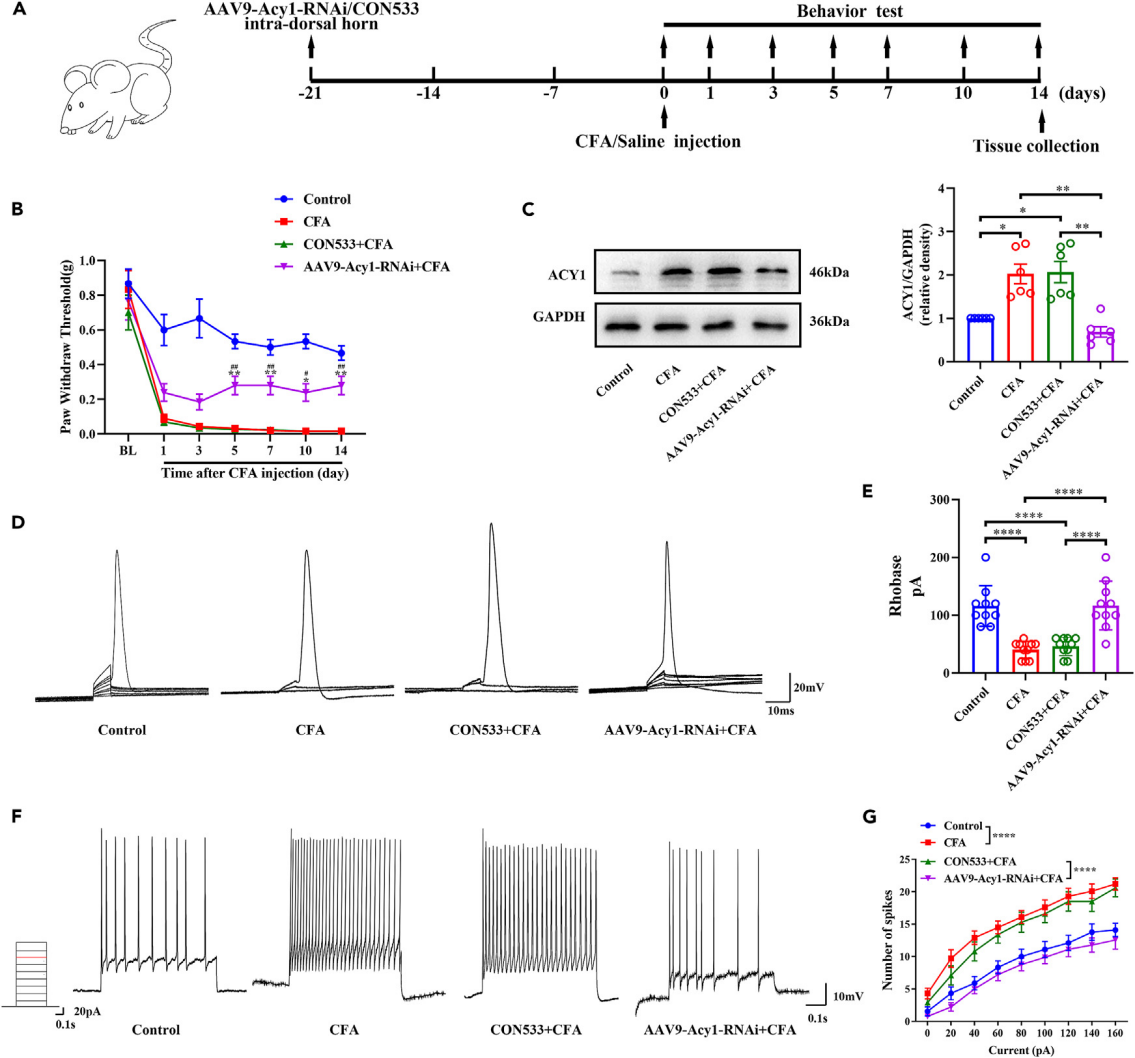
Effect of AAV9-Acy1-RNAi on pain behavior and spinal excitability in complete Freund adjuvant (CFA)-injected mice (A) Experimental diagram shows the timeline of the AAV9-Acy1-RNAi injection, CFA injection, and behavioral tests. (B) Pre-injection of AAV9-Acy1-RNAi markedly increases the mechanical thresholds in CFA-induced inflammatory pain mice (^∗^p < 0.05 vs. CFA, ^∗∗^p < 0.01 vs. CFA, ^#^p < 0.05 vs. CON533+ CFA, ^##^p < 0.01 vs. CON533+ CFA, two-way ANOVA with the Bonferroni post hoc test, n = 6). (C) Intradorsal horn injection of AAV9-Acy1-RNAi decreases the expression of ACY1 in the spinal cord after CFA injection (^∗^p < 0.05, ^∗∗^p < 0.01, n = 6). (D) Representative graphs of rheobase in the control, CFA, CON533+ CFA, and CFA mice with AAV9-Acy1-RNAi pretreatment. (E) The rheobase level is increased in AAV9-Acy1-RNAi pretreatment mice (^∗∗∗∗^p < 0.0001, one-way analysis of variance [ANOVA] with the Tukey multiple comparison test; n = 9, 10, 10, and 9 neurons in the control, CFA, CON533+ CFA, and AAV9-Acy1-RNAi+CFA groups, respectively). (F) Representative traces of the action potentials with a 120-pA current injection in each group. (G) The action potential frequency is decreased in AAV9-Acy1-RNAi pretreatment mice (^∗∗∗∗^p < 0.0001, two-way ANOVA with the Bonferroni post hoc test; n = 9, 10, 10, and 9 neurons/4 mice in the control, CFA, CON533+ CFA, and AAV9-Acy1-RNAi+CFA groups, respectively).

For ACY1 knockdown, we expressed AAV-Acy1-RNAi in the spinal cord via intradorsal horn microinjection. Behavioral data showed that the baseline mechanical thresholds showed no statistical differences between the groups. The mechanical thresholds significantly decreased after CFA injection. Additionally, reducing the expression of ACY1 using AAV9-Acy1-RNAi significantly alleviated CFA-induced mechanical allodynia (Figures 4A and 4B). Western blot analysis results revealed that the protein expression of ACY1 was significantly increased after CFA injection. Furthermore, the expression of ACY1 in the spinal cord of mice pre-injected with AAV9-Acy1-RNAi was reduced after CFA injection (Figure 4C). Moreover, as shown in Figures 4D and 4E, the rheobase of spinal cord neurons was signally decreased in CFA mice in contrast with control mice. However, spinal ACY1 knockdown significantly increased rheobase levels after CFA injection. In addition, as shown in Figures 4F and 4G, the number of APs in AAV9-Acy1-RNAi injected mice with CFA was significantly lower than that in CFA mice and CON533+ CFA mice. Notably, the rheobase and AP frequency did not differ significantly between CFA mice and CON533+ CFA mice, indicating that AAV did not affect the excitability of spinal neurons. These data indicate that spinal ACY1 knockdown could decrease the activity of spinal dorsal horn neurons and alleviate inflammatory mechanical hyperalgesia.

### Suppressing spinal ACY1 alleviated SNI-induced pain behavior and spinal hyperexcitability

**Figure 5:**
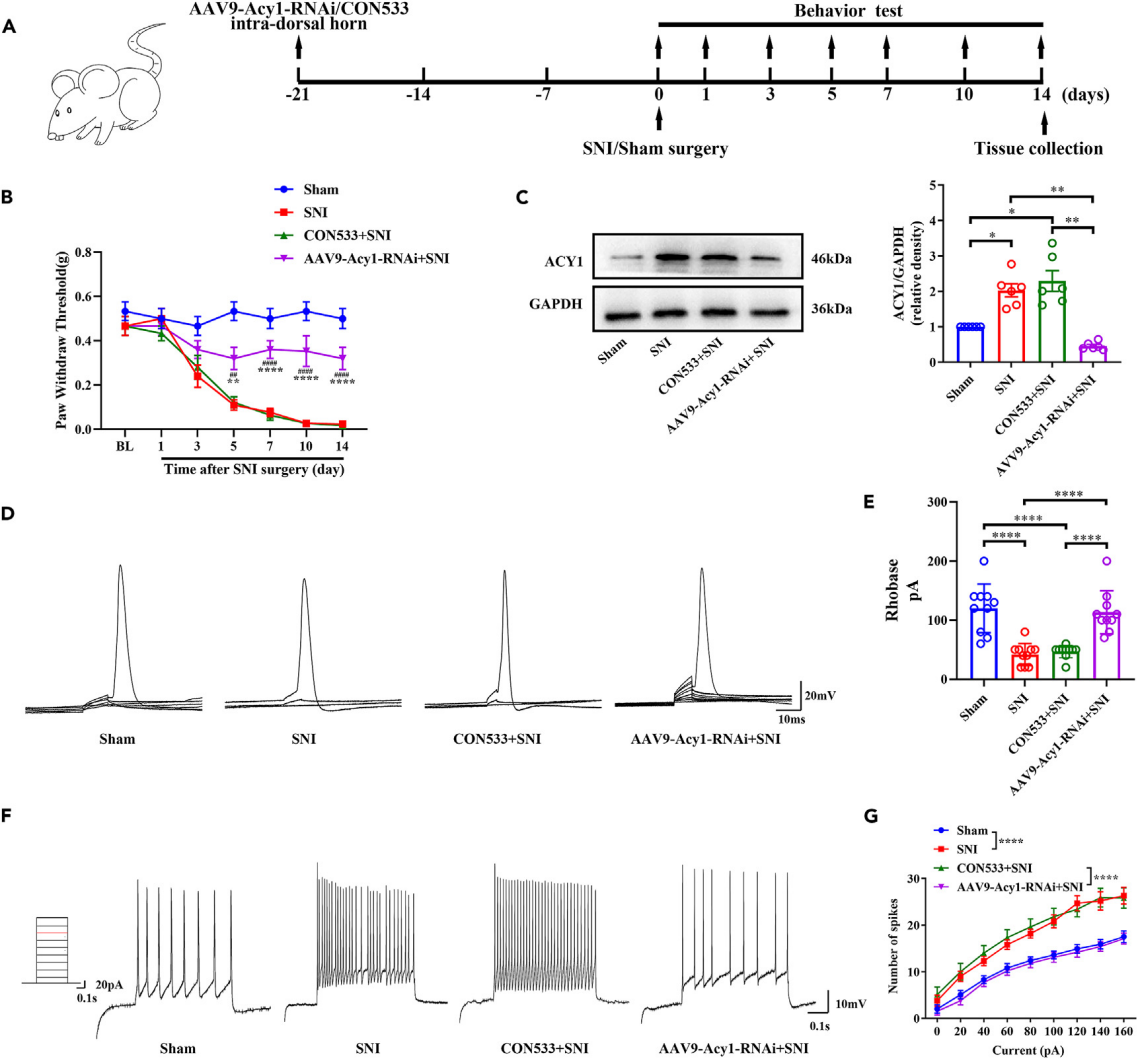
Effect of AAV9-Acy1-RNAi on pain behavior and spinal excitability in mice with spared nerve injury (SNI) (A) Experimental diagram shows the timeline of the AAV9-Acy1-RNAi injection, SNI surgery, and behavioral tests. (B) Pre-injection of AAV9-Acy1-RNAi markedly increases the mechanical thresholds in SNI-induced neuropathic pain mice (^∗∗^p < 0.01 vs. SNI, ^∗∗∗∗^p < 0.0001 vs. SNI, ^##^p < 0.01, ^####^p < 0.0001 vs. CON533+ SNI, two-way ANOVA with the Bonferroni post hoc test, n = 6). (C) Intradorsal horn injection of AAV9-Acy1-RNAi significantly decreases the expression of ACY1 in the spinal cord after SNI surgery (^∗^p < 0.05, ^∗∗^p < 0.01, n = 6). (D) Representative graphs of the rheobase levels in the sham, SNI, CON533+ SNI, and SNI mice with AAV9-Acy1-RNAi pretreatment. (E) The rheobase level is increased in SNI mice with Acy1 knockdown (^∗∗∗∗^p < 0.0001, one-way analysis of variance [ANOVA] with the Tukey multiple comparison test; n = 10 neurons in each group). (F) Representative traces of the action potentials with a 120-pA current injection in each group. (G) The action potential frequency is decreased in Acy1 knockdown SNI mice (^∗∗∗∗^p < 0.0001, two-way ANOVA with the Bonferroni post hoc test; n = 10 neurons/4 mice in each group).

As shown in Figures 5A and 5B, mechanical thresholds were decreased after SNI surgery, and AAV9-Acy1-RNAi pretreatment significantly alleviated SNI-induced mechanical allodynia. Western blot analysis results exhibited that the expression of ACY1 was significantly increased after SNI surgery. Moreover, the SNI surgery-induced upregulation of ACY1 in mice pre-injected with AAV9-Acy1-RNAi in the spinal cord was significantly suppressed (Figure 5C). Furthermore, as shown in Figures 5D and 5E, the rheobase of SNI mice with spinal ACY1 knockdown was increased compared with SNI mice without AAV injection. In addition, as shown in Figures 5F and 5G, the number of APs in spinal neurons significantly decreased in SNI mice with AAV9-Acy1-RNAi injection. Notably, the rheobase and AP frequency did not differ between SNI mice and CON533+ SNI mice, indicating that AAV did not affect the excitability of spinal neurons. These data indicated that spinal ACY1 knockdown could decrease the activity of neurons in the spinal dorsal horn to alleviate neuropathic mechanical hyperalgesia.

### ACY1 interacted with SphK1 and promoted its membrane translocation in both chronic pain models

**Figure 6:**
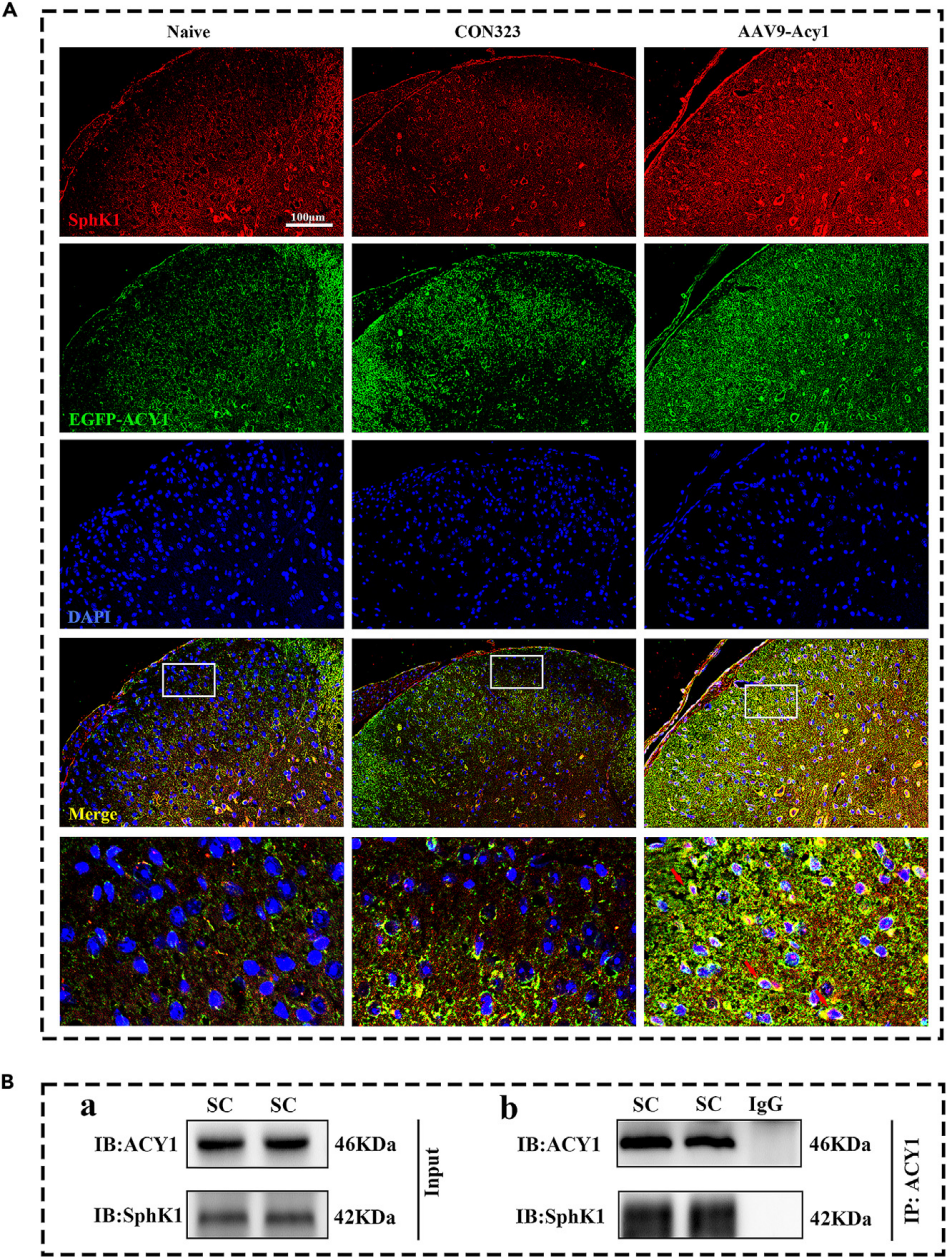
The relationship of aminoacylase 1 (ACY1) and sphingosine kinase 1 (SphK1) in the spinal dorsal horn (A) Representative photomicrographs of EGFP-ACY1 (green) double fluorescence labeling with SphK1 (red) in the superficial layers of the spinal dorsal horn on day 21 after AAV9-Acy1 injection (n = 3, Scale bars: 100 μm). (B) The co-immunoprecipitation results showed that ACY1 interacted with SphK1. ACY1 was immunoprecipitated protein. SphK1 was detected protein. SC: spinal cord.

Our results indicated that ACY1 plays an important role in the development of chronic inflammatory and neuropathic pain. However, the underlying mechanisms remain unclear. Therefore, we explored the involvement of spinal ACY1 in chronic pain. Recently, sphingosine kinase 1 (SphK1) and its product, sphingosine-1-phosphate (S1P), were found to be involved in pain modulation according to a growing body of evidence. SphK1 translocates from the cytoplasm to the membrane after activation to catalyze formation of the important bioactive sphingolipid mediator S1P.^27^ ACY1 can interact with and activate SphK1 and increase S1P secretion.^28^ We hypothesized that spinal ACY1 interacts with SphK1 and promotes its plasma membrane translocation, resulting in the upregulation of S1P and the development of chronic pain. To determine the cellular localization of ACY1 and SphK1 in the spinal dorsal horn, we first conducted immunofluorescence of ACY1 and SphK1 in spinal cord sections of mice that were pre-transfected with AAV9-Acy1. As previously mentioned, recombinant AAV9-Acy1 was fused to the C-terminus of EGFP, which provided a fluorescent tag for identifying AAV-targeted neurons expressing the ACY1 peptide aptamer. As shown in Figure 6A, EGFP-ACY1 was highly colocalized with SphK1 in the spinal dorsal horn. Next, we examined the relationship between ACY1 and SphK1 using co-immunoprecipitation. Co-immunoprecipitation results showed that there was an interactive relationship between ACY1 and SphK1 in the spinal cord of mice (Figure 6B).

**Figure 7:**
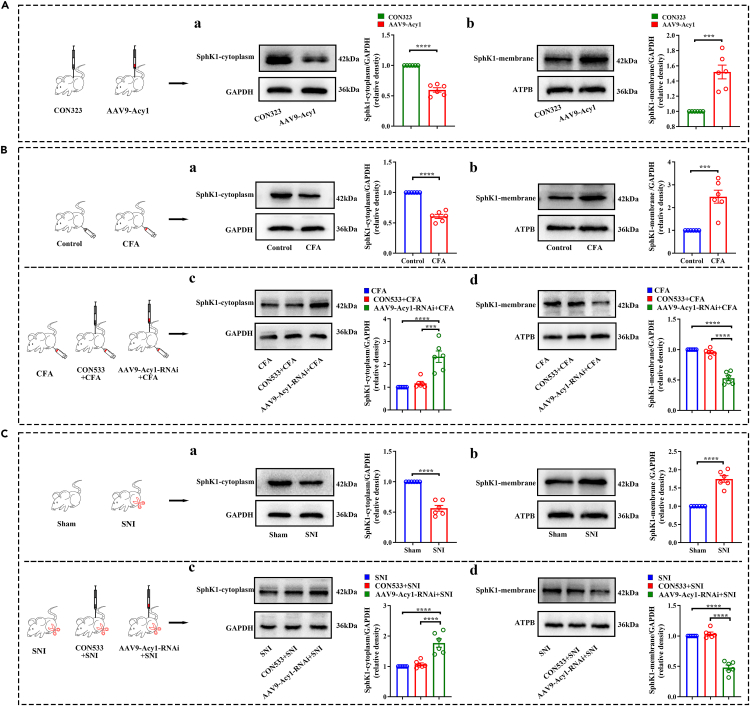
Aminoacylase 1 (ACY1) interacts with sphingosine kinase 1 (SphK1) and promoted its membrane translocation in the spinal cord of mice with chronic inflammatory and neuropathic pain (A) Intradorsal horn injection of AAV9-Acy1 promotes membrane translocation of SphK1 in the spinal cord. (a–b) The expression of SphK1 in the cytoplasm is decreased, while the expression of SphK1 in the plasma membrane is significantly increased in the spinal cord on day 21 after AAV9-Acy1 injection. (B) Pretreatment with AAV9-Acy1-RNAi prevents complete Freund adjuvant (CFA)-induced plasma membrane translocation of SphK1 in the spinal cord. (a–b) The expression of SphK1 in the cytoplasm is decreased while the expression of SphK1 in the plasma membrane is significantly increased in the spinal cord on day 14 after CFA injection. (c–d) Pretreatment with AAV9-Acy1-RNAi inhibits CFA-induced cytoplasmic SphK1 reduction and an increase of SphK1 in the plasma membrane. (C) Pretreatment with AAV9-Acy1-RNAi prevents SNI-induced plasma membrane translocation of SphK1 in the spinal cord. (a–b) The expression of SphK1 in the cytoplasm is decreased, while the expression of SphK1 in the plasma membrane is significantly increased in the spinal cord on day 14 after SNI surgery. (c–d) Pretreatment with AAV9-Acy1-RNAi inhibits SNI-induced cytoplasmic SphK1 reduction and an increase of SphK1 in the plasma membrane. (^∗∗∗^p < 0.001, ^∗∗∗∗^p < 0.0001, n = 6).

Next, we performed Western blot analysis on the subcellular fraction to confirm the subcellular expression of SphK1. As shown in Figure 7A, the expression of SphK1 was significantly increased in the plasma membrane on day 21 after AAV9-Acy1 spinal injection in naive mice, suggesting that ACY1 promotes the translocation of SphK1 from the cytoplasm to the plasma membrane. As shown in Figures 7B and 7C, the expression of SphK1 was upregulated in the plasma membrane on day 14 after CFA injection and SNI surgery. This finding suggests that the plasma membrane translocation of SphK1 was increased in mice with chronic pain. Next, we examined whether plasma membrane translocation of SphK1 in mice with chronic pain could be regulated by ACY1. As expected, suppression of spinal ACY1 by pretreatment with AAV9-Acy1-RNAi significantly decreased membrane translocation of SphK1 in both chronic pain models.

### ACY1 promoted S1P-induced glutamatergic neurons and astrocyte activation in the CFA and SNI models

**Figure 8:**
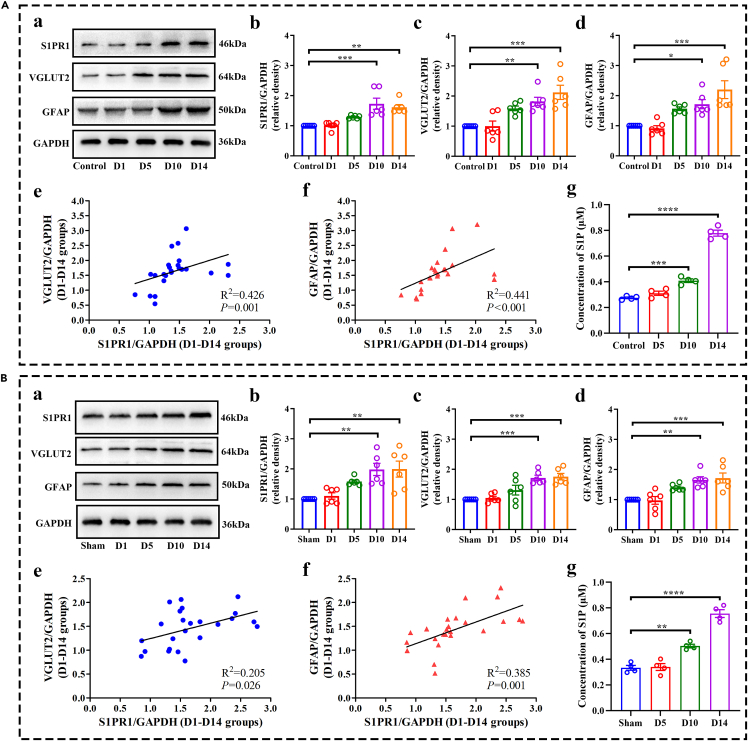
sphingosine-1-phosphate (S1P) induces the activation of glutamatergic neurons and astrocytes in complete Freund adjuvant (CFA) and spared nerve injury (SNI) mice (A and B) Expression and correlation analysis of S1P signaling between VGLUT2 and GFAP in CFA and SNI mice. (a) Western blot bands of S1PR1, VGLUT2, and GFAP in the spinal cord of CFA and SNI mice. (b-d) Western blot analysis shows S1PR1, VGLUT2, and GFAP protein expressions were obviously upregulated from 10 to 14 days in the spinal cord of CFA and SNI mice. (e-f) correlation analysis of S1PR1 between VGLUT2 and GFAP in CFA and SNI mice. (g) The expression level of S1P in the spinal cord is upregulated from 10 to 14 days after CFA injection and SNI surgery. (^∗^p < 0.05, ^∗∗^p < 0.01, ^∗∗∗^p < 0.001, ^∗∗∗∗^p < 0.0001, n = 4–6).

Altered sphingolipid metabolism is linked to nociceptive processing in an increasing number of studies. Previous studies have validated that S1P increases glutamate release through autocrine action.^29^^,^^30^ Glutamate, the most abundant excitatory neurotransmitter, plays a key role in central sensitization and chronic pain.^31^ In addition, recent animal studies from Chen et al. indicated that increased S1P in the spinal cord selectively activates astrocytes via S1PR1, resulting in the development of neuropathic pain.^32^ Accordingly, we speculated that ACY1 may interact with SphK1 to drive chronic pain through S1P-induced glutamatergic neuron and astrocyte activation. Therefore, we first examined the expression level of S1P, S1PR1, the glutamatergic neuronal marker VGLUT2, and the astrocyte marker GFAP. The ELISA results showed that S1P expression was increased in the spinal cord of CFA- and SNI-induced chronic pain mice (Figures 8A–8G, and 8B–8G). Western blot results showed that S1PR1, VGLUT2, and GFAP (Figures 8A and 8B) also significantly increased in both chronic pain mice models. Next, we performed correlation analysis of S1PR1 between VGLUT2 and GFAP. The results showed that expression levels of VGLUT2 and GFAP were significantly determined by S1PR1 in the cytomembrane when CFA and SNI models were established.

**Figure 9:**
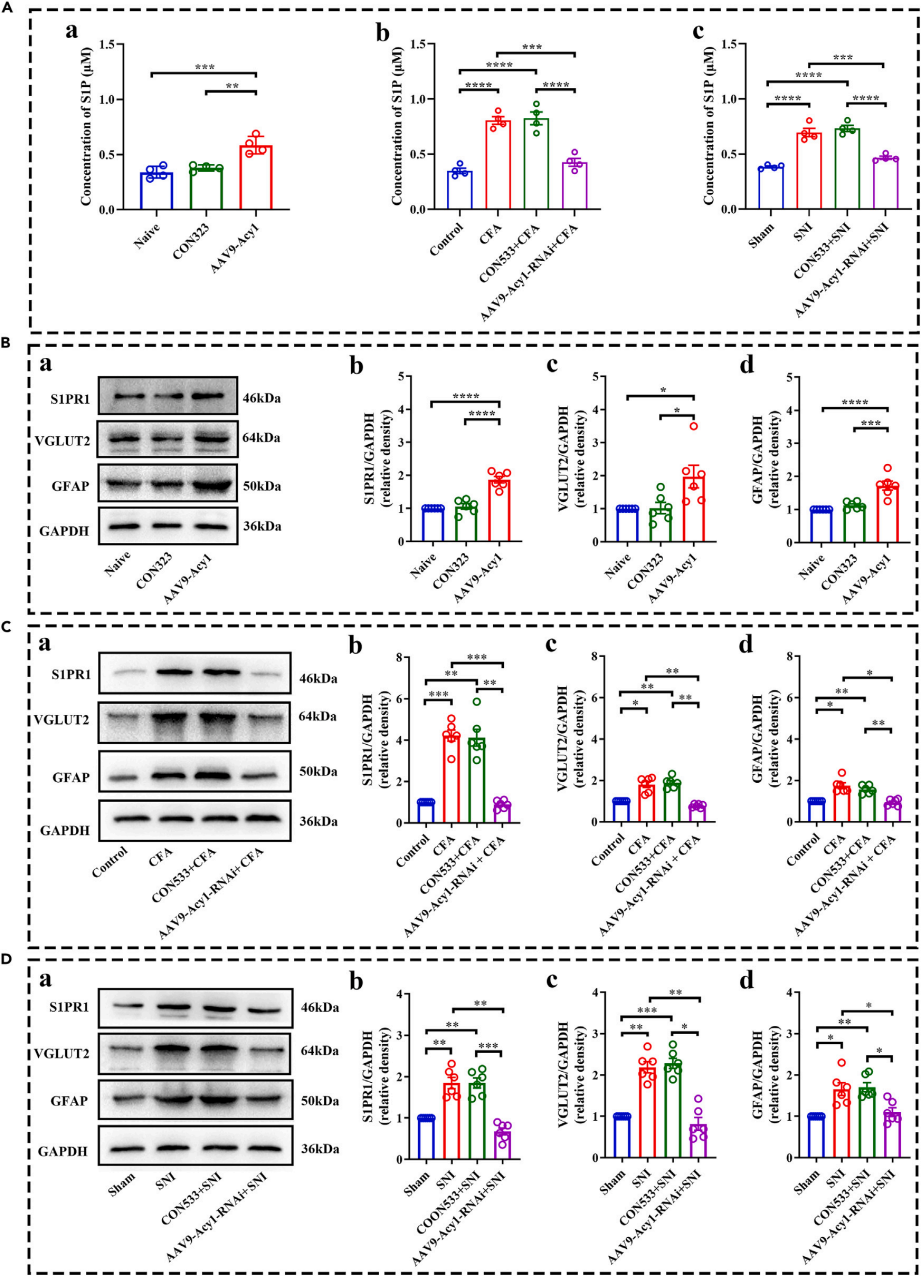
Aminoacylase 1 (ACY1)-mediated sphingosine-1-phosphate (S1P) induces the activation of glutamatergic neurons and astrocytes (A) ELISA shows the expression level of S1P in each group. (a) The expression level of S1P in the spinal cord is upregulated at 21 days after the AAV9-Acy1 injection. (b-c) Intradorsal horn injection of AAV9-Acy1-RNAi reduces the CFA- and SNI-induced upregulation of S1P in the spinal cord. (B) AAV9-Acy1 promotes the activation of glutamatergic neurons and astrocytes in naive mice. (a-d) Western blot analysis shows S1PR1, VGLUT2, and GFAP protein expressions were obviously upregulated 21 days after the AAV9-Acy1 injection in the spinal cord of naive mice. (C and D) AAV9-Acy1-RNAi inhibits the activation of glutamatergic neurons and astrocytes in complete Freund adjuvant (CFA) and spared nerve injury (SNI) mice. (a–d) Western blot analysis shows that intradorsal horn injection of AAV9-Acy1-RNAi inhibits the upregulation of S1PR1, VGLUT2, and GFAP in CFA and SNI mice. (^∗^p < 0.05, ^∗∗^p < 0.01, ^∗∗∗^p < 0.001, ^∗∗∗∗^p < 0.0001, n = 4–6).

Next, to further investigate whether ACY1 is involved in S1P-induced glutamatergic neuron and astrocyte activation, we intervened with spinal ACY1 using an intradorsal horn injection of AAV9-Acy1 and AAV9-Acy1-RNAi. As shown in Figure 9A, the ELISA results showed that the levels of S1P were significantly increased in the spinal cord of wild-type mice with ACY1 overexpression. Additionally, the expression of S1P in CFA and SNI mice were upregulated. Suppressing spinal ACY1 by pretreating with AAV9-Acy1-RNAi could reverse the upregulation of S1P in CFA and SNI mice. These results indicated that ACY1 mediates the release of S1P in both CFA and SNI pain models. As shown in Figure 9B, the Western blot results showed that the expression of S1PR1, VGLUT2, and GFAP were upregulated in the spinal cord of wild-type mice with ACY1 overexpression. Additionally, the knockdown of spinal ACY1 by AAV9-Acy1-RNAi pretreatment reversed the upregulation of S1PR1, VGLUT2, and GFAP in CFA and SNI mice (Figures 9C and 9D), indicating that ACY1 mediates the activation of S1P in both CFA and SNI pain models. Together, these results suggest that ACY1 interacts with SphK1 to promote the production of S1P, resulting in glutamatergic neuron and astrocyte activation in both the CFA and SNI models.

## Discussion

In this study, Nav1.8 knockout mice served as the control to screen for a differential protein expression profile in the spinal cord of wild-type chronic pain mice. In the preliminary models, it seemed that Nav1.8 KO had a more prominent effect on the neuropathic model. However, the paw withdrawal threshold (PWT) of the CFA group declined during the first day, and the drop of PWT was even greater in the CFA group. Thus, pain sensitization occurs earlier and present more severe hypersensitivity for wild-type mice in the CFA model compared to the SNI model. Moreover, significant differences exist with the methods for constructing the SNI and CFA models. Whether Nav1.8 may be more effective for neuropathic treatment needs further investigation. Through proteomic analysis, we found that < 1% of all quantified proteins were significantly upregulated and downregulated in each pain model, indicating the high specificity of these differentially expressed proteins downstream of Nav1.8. Among these differentially expressed proteins, the abundance of ACY1 was significantly increased in both inflammatory and neuropathic pain mice when compared with Nav1.8 knockout mice with chronic pain. Then, we examined the protein levels of ACY1 in the original protein lysis buffer using Western blot analysis, and the validation results were consistent with the proteomic results.

ACY1 is the most widely expressed aminoacylase that catalyzes amino acid deacylation in protein degradation.^33^ Reportedly, ACY1 is involved in various human diseases.^34^^,^^35^^,^^36^ ACY1 deficiency due to mutations in *ACY1* is a rare inborn error of metabolism. To date, patients with ACY1 deficiency have various clinical presentations but most present with neurological symptoms, such as mental retardation, bradykinesia, muscle hypotonia, language developmental disorders, growth retardation, and autism behavior.^37^^,^^38^^,^^39^ Recent studies have also demonstrated that the overexpression of ACY1 improves cell growth, invasion, migration, and tumorigenesis.^36^^,^^40^ Hence, we speculated that ACY1 might play a role in promoting neuronal excitability in the nervous system. However, the effects of ACY1 on neuronal excitability and pain modulation have not yet been described. Thus, considering its expression in neurons and its role in neurological diseases, we chose ACY1 as a potential candidate to perform further study.

To verify the involvement of ACY1 in chronic pain, we established two chronic pain models using wild-type mice and examined the expression patterns of ACY1. Expression of spinal ACY1 was significantly upregulated from day 5–14 after CFA injection and SNI surgery, suggesting that ACY1 may be implicated in inflammatory and neuropathic pain signaling. In a previous study, ACY1 is abundantly expressed in the kidneys and central nervous system.^33^ Through cellular localization of spinal ACY1, we found that Nav1.8 and ACY1 were mainly colocalized with NeuN in the dorsal horn of the spinal cord. These results suggest that increased expression of ACY1 in the spinal cord is involved in the SNI and CFA procedures.

To further test the central role of ACY1 in pain modulation, we constructed AAV9-Acy1 and AAV9-Acy1-RNAi to specifically upregulate and knockdown the expression of ACY1, respectively. We found that overexpression of ACY1 by intradorsal horn injection of AAV9-Acy1 resulted in mechanical hyperalgesia in normal mice. In addition, the overexpression of ACY1 enhanced the excitability of spinal neurons. Furthermore, suppression of spinal ACY1 by AAV9-Acy1-RNAi decreased the activity of spinal dorsal horn neurons and reversed mechanical allodynia in mice with inflammatory and neuropathic pain. Taken together, these results experimentally validate the critical role of ACY1 in chronic pain. However, the precise mechanisms by which ACY1 is involved in the development of chronic pain remain unclear.

In recent decades, alterations in sphingolipid metabolism have been associated with numerous neurological diseases.^41^ Sphingolipids are a critical class of bioactive lipids that are abundantly expressed in the central nervous system and comprise ceramides and their metabolites, such as sphingosine and S1P.^42^ SphK1, a key enzyme in maintaining sphingolipid metabolism homeostasis, is translocated from the cytoplasm to the membrane after activation to catalyze the formation of the important bioactive sphingolipid mediator S1P.^27^ Indeed, ACY1 can interact with SphK1 and improve its membrane translocation.^28^ More recently, preclinical and clinical studies have clearly shown that the dyshomeostasis of S1P contributes to the development of chronic pain.^43^ Therefore, we speculated that ACY1 may participate in the development of chronic pain by affecting the SphK1-dependent spinal sphingolipid metabolism balance.

To test our hypothesis, we first examined the relationship between ACY1 and SphK1 in the spinal cord, and the immunofluorescence results showed that ACY1 was highly co-labeled with SphK1 in the spinal dorsal horn. Additionally, there was protein interaction between ACY1 and SphK1 in the spinal cord of mice. Further, our findings showed that plasma membrane SphK1 was significantly increased after ACY1 was overexpressed in the spinal cord of naive mice. Inhibition of ACY1 significantly decreased membrane translocation of SphK1 in both chronic pain models. Meanwhile, using ELISA, we found that the expression level of S1P was significantly upregulated after ACY1 overexpression. These findings suggest a facilitation effect of ACY1 on the translocation of SphK1, resulting in an increase in S1P in chronic pain.

In recent years, S1P signaling has been shown as a vital modulator of pain pathways according to accumulating evidence.^43^^,^^44^ S1P also performs strong inflammatory and nociceptive actions.^44^ S1P acts in an autocrine or paracrine manner after synthesis, and the cellular functions of S1P are mainly mediated through its receptors, comprising five G protein-coupled receptors (S1PR1–5).^43^ In addition, S1P can trigger glutamate secretion and enhance neuronal excitability.^45^^,^^46^ Glutamate, the most abundant excitatory neurotransmitter, plays an important role in central sensitization and chronic pain.^31^ Our recent study and other studies have indicated that the activation of glutamatergic neurons plays a critical role in the development of chronic pain.^47^^,^^48^^,^^49^ Our results also showed that overexpression of spinal ACY1 increased the excitability of mouse spinal neurons. It is noteworthy to mention that Chen et al. reported that increased S1P in the spinal cord selectively activated astrocytes via S1PR1, resulting in the development of neuropathic pain.^32^ Glial cell activation plays an indispensable role in the maintenance of chronic pain.^50^ Therefore, we hypothesized that ACY1-induced upregulation of S1P would trigger glutamate secretion and enhance neuron excitability in an autocrine manner, as well as activate astrocytes in a paracrine manner.

To confirm this hypothesis, we detected the protein levels of the glutamatergic neuron marker VGLUT2 and astrocyte marker GFAP. As expected, the expressions of S1P, S1PR1, VGLUT2, and GFAP were significantly increased in both mouse models of chronic pain. In addition, we analyzed the correlation of S1PR1 between VGLUT2 and GFAP, indicating that S1PR1 had a significant effect on the expression levels of VGLUT2 and GFAP in both CFA and SNI models. Furthermore, overexpressing ACY1 upregulated the expression of S1P, S1PR1, VGLUT2, and GFAP in the spinal cord of naive mice, and suppressing spinal ACY1 by pretreating with AAV-Acy1-RNAi could reverse the expression of these proteins in both chronic pain mouse models. As reported in a previous study, S1P-based drugs have been approved for numbers of clinical indications and can also be used for chronic pain treatment.^43^ For example, proof-of-concept clinical trials of FTY720 in patients with breast cancer have been initiated to explore its effectiveness in preventing and/or treating chemotherapy-induced neuropathic pain.^43^ However, the clinical application of S1P-based drugs in pain therapy has not yet made breakthrough progress, which may be attributed to the diversity of receptor types, drug specificity, and side effects.^51^ Combined with our results that targeted ACY1 could directly inhibit the increase of S1P, which may have a more effective analgesic effect, suggesting that ACY1 is a potential analgesic target.

### Limitations of the study

Several limitations should be considered when interpreting the current study. First, it is important to analyze the role of the dorsal root ganglion cells in pain processing because this is where Nav1.8 is majorly expressed. As a large number of studies and our previous study have explored the potential mechanism of Nav1.8 underlying chronic pain processing in the DRG, this study mainly focused on the downstream pathway of Nav1.8 in the spinal cord.^11^^,^^52^ Therefore, we did not analyze the DRG in this study. Second, gender differences have a large impact on pain syndromes. One mechanism that has emerged is the influence of estrogen on the glutamate pathway, ultimately affecting neuronal physiology and behavior.^53^ However, only male mice were used in our study. Therefore, the present findings need to be confirmed in female mice in further studies. Third, since we have found the protein of interest and focused on exploring the underlying mechanism of ACY1 in chronic pain, we did not perform further analysis and mining on the proteomics data in this study.

**Figure 10:**
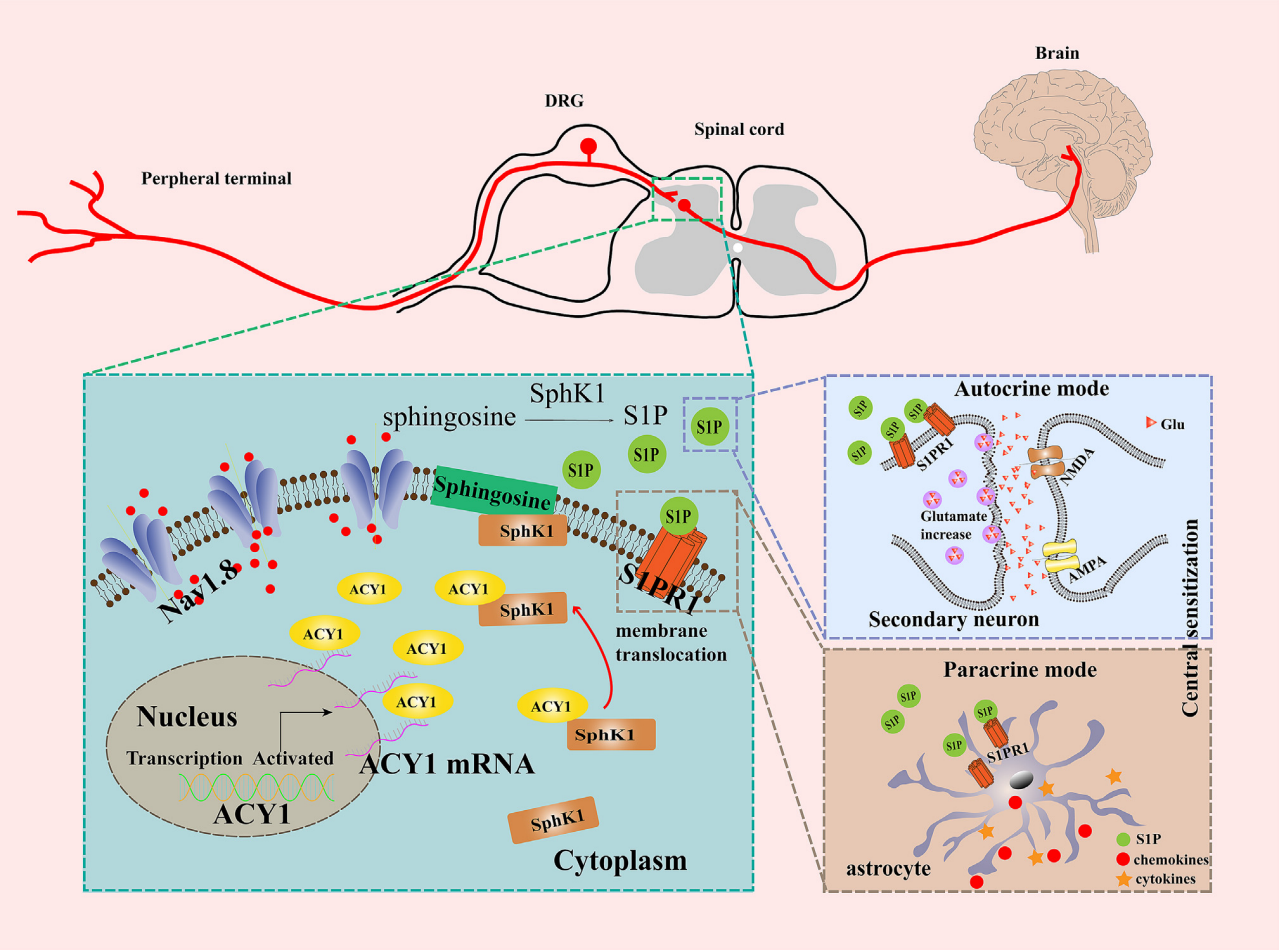
Schematic diagram of aminoacylase 1 (ACY1) as a downstream of TTX-resistant sodium channel 1.8 (Nav1.8) promotes the plasma membrane translocation of sphingosine kinase 1 (SphK1) and triggers the development of chronic pain Complete Freund adjuvant (CFA)- and spared nerve injury (SNI)-induced the increased expression and activation of Nav1.8, which led to the upregulation of ACY1. ACY1 interacted with SphK1 and promoted its transportation to the plasma membrane, which resulted in the production of sphingosine-1-phosphate (S1P) on the cell membrane. Then, the upregulated S1P trigged the activation of glutamatergic neurons and astrocytes by autocrine and paracrine mode, respectively, which in turn resulted in neuronal hyperexcitability and the development of chronic pain. DRG, dorsal root ganglion; S1PR1, sphingosine-1-phosphate receptor 1; Glu, glutamate; AMPA, alpha-amino-3-hydroxy-5-methyl-4-isoxazolepropionic acid subtype receptor; NMDA, N-methyl-D-aspartate subtype receptor; mRNA, messenger RNA.

In conclusion, this study demonstrated that ACY1, a downstream effector protein of Nav1.8, regulates the plasma membrane translocation of SphK1 and promotes the production of S1P on the cell membrane. The upregulation of S1P triggers the activation of glutamatergic neurons and astrocytes, which in turn results in neuronal hyperexcitability and the development of chronic pain (Figure 10). This study provides a novel co-target that can be effective for both inflammatory and neuropathic pain.

## STAR★Methods

### Key resources table

**Table:** 

REAGENT or RESOURCE	SOURCE	IDENTIFIER
**Antibodies**		

rabbit anti-ACY1	ABclonal	Cat# A6351
rabbit anti-VGLUT2	ABclonal	Cat# A13648
55133-1-AP rabbit anti-S1PR1	Proteintech	Cat# 55133-1-AP
anti-GFAP	Cell Signaling Technology	Cat# 3670S
rabbit anti-GAPDH	Proteintech	Cat# 10494-1-AP
ACY1	Biotechne	Cat# AF2700
sphingosine kinase 1	Santa Cruz	Cat# sc-365401
rabbit anti-Nav1.8	Alomone	Cat# ASC-016
mouse anti-NeuN	Abcam	Cat# ab104224
rabbit anti-SphK1	Proteintech	Cat# 10670-1-AP
mouse anti-GFP-Tag	ABclonal	Cat# AE012
Alexa Fluor 594-labeled donkey anti-rabbit	Jackson ImmunoResearch	Cat# 711-515-152
Alexa Fluor 488-labeled donkey anti-mouse	Jackson ImmunoResearch	Cat#715-485-150

**Chemicals, peptides, and recombinant proteins**		

CFA	Sigma	Cat# F5881
RIPA lysis buffer	Beyotime Biotechnology	Cat# P0013C
enhanced chemiluminescence reagents	YEASEN	Cat# 36222ES60
Triton X-100	Sigma-Aldrich	Cat# 9036-19-5
pentobarbital sodium	Sigma-Aldrich	Cat# P3761-5G
Sucrose	Sigma-Aldrich	Cat# V900116
KCl	Sigma-Aldrich	Cat# P9333
NaH2PO4	Sigma-Aldrich	Cat# 106342
CaCl2	Sigma-Aldrich	Cat# C4901
MgCl2	Sigma-Aldrich	Cat# M2393
NaHCO3	Sigma-Aldrich	Cat# W700433
Glucose	Sigma-Aldrich	Cat# D9434
HEPES	Sigma-Aldrich	Cat# H3375
EGTA	Sigma-Aldrich	Cat# E3889
Mg-ATP	Sigma-Aldrich	Cat# A2383
Na3-GTP	Sigma-Aldrich	Cat# G8877

**Critical commercial assays**		

membrane and cytoplasmic protein extraction kit	Abbkine	Cat#KTP3005
sphingosine-1-phosphate ELISA	Echelon Biosciences	Cat#K-1900

**Deposited data**		

The original proteomics data have been deposited to ProteomeXchange Consortium at https://proteomecentral.proteomexchange.org/cgi/GetDataset?ID=PXD042455	GeneChem	N/A

**Experimental models: Organisms/strains**		

Mice: Adult male C57BL/6J	Experimental Animal Research Center of Hubei Province, Wuhan, China	N/A
Mice: homozygous Nav1.8 knockout with a C57BL/6J background	Akopian et al. and Nassar et al.	N/A

**Recombinant DNA**		

AAV9-Acy1-RNAi	GeneChem	N/A
CON533	GeneChem	N/A
CON323	GeneChem	N/A
AAV9-Acy1	GeneChem	N/A

**Software and algorithms**		

Isobaric tags for relative and absolute quantitation (iTRAQ)-based proteomics analysis	GeneChem	N/A
Image Lab software analysis system V 5.2	Bio-Rad	https://www.bio-rad.com/
EPC10 amplifier and Patchmaster software	HEKA	https://www.heka.com
Clampfit software PClamp 10	Molecular Devices	https://www.moleculardevices.com/products/axon-patch-clamp-system/acquisition-and-analysis-software/pclamp-software-suite
GraphPad Prism V 7.0	GraphPad Software	https://www.graphpad.com/

**Other**		

stereotaxic instrument	RWD Life Sciences	Cat#68535
ChemiDoc XRS system	Bio-Rad	Cat#1708265
rotary slicer	Leica	Cat#CM1900
fluorescence microscope	Olympus	Cat#DP70
vibrating microtome	Leica	Cat#VT1200S
Patch electrode recording pipettes (64–10 6 MΩ)/borosilicate glass	WPI	Cat#1B150F-4
two-step horizontal puller	Sutter Health	Cat#P-1000
Spectrophotometer	Bio-Rad	Cat#680

### Resource availability

#### Lead contact

Further information and requests for resources and reagents should be directed to and will be fulfilled by the lead contact, Guangyou Duan (duangy@hospital.cqmu.edu.cn).

#### Materials availability

This study did not generate new unique reagents.

### Experimental model and subject details

#### Ethics statements

All animal procedures were approved by the Institutional Animal Care and Use Committee of Tongji Hospital, Tongji Medical College, Huazhong University of Science and Technology (Permit Number: TJH-20211013) and were performed in accordance with the National Institutes of Health Guide for the Care and Use of Laboratory Animals (National Institutes of Health Publications No. 8023, revised 1978).

#### Animals

Adult male C57BL/6J mice were provided by the Experimental Animal Research Center of Hubei Province, Wuhan, China. A colony of homozygous Nav1.8 knockout mice with a C57BL/6J background was generated as described previously^54^^,^^55^ and provided by Professor Stephen G. Waxman (Yale University School of Medicine, New Haven, CT, USA). All mice were kept at 23°C ± 1°C with a relative humidity of 60% ± 10% and a standard 12-h alternating light-dark cycle, with abundant food and drinking. For all mouse studies, mice of male sex were used.

### Method details

#### Establishment of the inflammatory pain model

The inflammatory pain model was induced using CFA according to a previously described method.^56^ After anesthetizing the mice with isoflurane, their left hind paws were swabbed with iodophor and then injected with CFA (20 μL, F5881; Sigma, St. Louis, USA) or a saline solution subcutaneously using a 27-gauge needle.

#### Establishment of the neuropathic pain model

The neuropathic pain model was induced by spared nerve injury surgery, according to the method described previously.^57^ The mice were anesthetized by intraperitoneal injection of pentobarbital sodium (50 mg/kg). Briefly, the left sciatic nerve trifurcates were exposed to blunt dissection. The common peroneal and tibial nerves were ligated using 6-0 silk suture. The distal adjacent nerve was transected at 2–4 mm. The sural nerve was protected from damage. The muscles and skin were closed using sutures. The same procedure was performed, excluding nerve ligation and transection, in the sham-operated group.

#### Behavioral tests

Mechanical allodynia was assayed by testing the mice’s paw withdrawal threshold (PWT) in response to stimulation by von Frey filaments.^58^^,^^59^ The mice were placed in isolated Plexiglas chambers on an elevated mesh floor and allowed to acclimatize for 30 min before testing. The PWT was assessed using the Dixon up-down method. Briefly, the tips of the von Frey filaments (0.008 g, 0.02 g, 0.04 g, 0.16 g, 0.4 g, 0.6 g, 1.0 g, 1.4 g, and 2 g) were stuck perpendicularly to the mid-plantar surface of the hind paws of mice with gradually increasing force. Abrupt paw lifting or licking in response to the filaments was defined as a positive response. When a positive response occurred, the next descending filament was tested after resting for 5 min. When no positive response occurred, the next-ascending filament was tested. The lowest force-eliciting positive response was determined to be the PWT.

#### Proteomics

Isobaric tags for relative and absolute quantitation (iTRAQ)-based proteomics analysis (Genechem Co., Ltd., Shanghai, China) was performed to profile changes in the abundance of spinal cord proteins in two well-established animal pain models of chronic pain using wild-type and Nav1.8 knockout mice. Both the CFA-induced inflammatory pain and SNI-induced neuropathic pain models simulated the features of mechanical hypersensitivity in patients with chronic pain. Mice were assessed for nociceptive thresholds before and 1, 3, 5, 7, 10, and 14 days after the induction of inflammatory and neuropathic pain, and the mice were sacrificed for dissection of the lumbar spinal cord 14 days after the last behavioral test according to our preliminary results and the findings in previous studies.^11^^,^^60^^,^^61^ Twelve samples were prepared for iTRAQ analysis, consisting of three biological replicates per sample. The samples included four conditions: CFA-induced inflammatory pain in wild-type mice versus CFA-induced inflammatory pain in Nav1.8 knockout mice, and SNI-induced neuropathic pain in wild-type mice versus SNI-induced neuropathic pain in Nav1.8 knockout mice. Only mice that experienced significant mechanical allodynia were included in our study.

#### Construction of adeno-associated virus and stereotaxic injection

The following recombinant adeno-associated viruses (AAVs) were used: AAV9-Acy1-RNAi (titer: 1.77 × 10^13^ V.G./mL) and vector control CON533 (titer: 1.08 × 10^13^ V.G./mL), AAV9-Acy1 (titer: 1.01 × 10^14^ V.G./mL) and vector control CON323 (titer: 1.82 × 10^13^ V.G./mL) were generated by GeneChem Co., Ltd. (Shanghai, China). The sequence of AAVs were shown in Table S1. Both recombinant AAVs were fused to enhanced green fluorescent protein (EGFP) C-terminus to provide a fluorescent tag to identify neurons expressing EGFP-aminoacylase 1 (ACY1). Viruses and non-targeting control viruses were injected into the dorsal horn of mice’s lumbar spinal. Intradorsal horn injections were performed as previously described.^62^^,^^63^^,^^64^ Briefly, the surgical area is prepared by wiping bench and heating pad with disinfectant. This is followed by anesthetizing the mouse with pentobarbital sodium (50 mg/kg). The fur from the lower back to the neck of the mouse is shaved, and the skin on the back is disinfected with 75% alcohol. The area of skin incision is identified by pressing the fingers gently at the last rib to locate the L1 vertebra, and a 3–4 cm skin incision is made to expose the muscle. Then, the muscles are separated to expose the vertebral column. T11 and adjoining T12 and T13 are located, laminectomies of the dorsal aspects of T11-T13 are performed, and the vertebral column is fixed with a stereotaxic instrument (RWD Life Science, Shenzhen, China). The tip of the needle is positioned at the midline blood vessel of the spinal cord. The needle is moved 0.5 mm to the left using the Vernier scale on the micromanipulator of the stereotaxic instrument. Then, the needle is lowered to the spinal cord until it has punctured the dura and inserted to a depth of 0.3 mm. Once the needle is in place, 0.2 μL viral particles are administered at a rate of 100 nL/min. On completion of the injection, the needle is left in the spinal cord for 5 min before removal. The incision is closed with 4.0 nylon thread, ensuring that the wound is clear of all debris before closing. After surgery, the mice were allowed to recover from anesthesia in an insulation can before being returned to their home cages. Subsequent experiments were performed 3 weeks after virus injection when the viral expression was maximal and stable.

#### Western blot analysis and co-immunoprecipitation

Mice were anesthetized by overdoses of pentobarbital sodium (60 mg/kg), and L4–L6 spinal cord segments were rapidly collected and stored at −80°C. Total protein was homogenized in a RIPA lysis buffer containing protease inhibitors (Beyotime Biotechnology, Jiangsu, China) on ice. Cytosol and plasma membrane proteins were fractionated using a membrane and cytoplasmic protein extraction kit (Abbkine, Wuhan, China) according to the manufacturer’s instructions. For Western blot analysis, equal amounts of protein (40 μg) were separated by 10% sodium dodecyl sulfate polyacrylamide gel electrophoresis (SDS-PAGE) and electro-transferred to polyvinylidene difluoride membranes (IPVH00010, Millipore, Burlington, MA, USA). After blocking with 5% fat-free milk in Tris-buffered saline containing 0.1% Tween 20 for 1 h at room temperature, the membranes were incubated with primary antibodies overnight at 4°C. The following primary antibodies were used: rabbit anti-ACY1 (A6351, ABclonal, Wuhan, China), rabbit anti-VGLUT2 (A13648, ABclonal), rabbit anti-S1PR1 (55133-1-AP, Proteintech), anti-GFAP (3670S, Cell Signaling Technology, Danvers, MA, USA), and rabbit anti-GAPDH (10494-1-AP, Proteintech). Then, the washed membranes were incubated with horseradish peroxidase-conjugated secondary antibodies for 2 h at room temperature. Signals were detected using enhanced chemiluminescence reagents (YEASEN, Shanghai, China) and visualized on a ChemiDoc XRS system (Bio-Rad, Hercules, CA, USA). The Image Lab software analysis system (Bio-Rad) was used to quantify the integrated optical density of each band. Co-immunoprecipitation analysis was performed according to the manufacturer’s instructions (Thermo Scientific, Waltham, MA, USA). Briefly, the proteins were incubated with the ACY1 antibody (AF2700, Biotechne, RWD Life Science) and protein A/G. After washing, the mixture was separated by SDS-PAGE and detected using the sphingosine kinase 1 (SphK1) antibody (sc-365401, Santa Cruz, CA, USA).

#### Immunofluorescence

Mice were deeply anesthetized by overdoses of pentobarbital sodium (60 mg/kg) and perfused intracardially with cold phosphate-buffered saline (PBS), followed by 4% cold paraformaldehyde (PFA). The L4–L6 segments of the spinal cord were dissected and kept in 4% PFA for 24 h at 4°C for post-fixation, and then placed in 30% sucrose solution for dehydration. The tissues were cross-cut transversely into 20-μm sections using a rotary slicer (CM1900, Leica, Wetzlar, Germany). The sections were infiltrated with 0.3% Triton X-100 for 15 min and blocked with PBS containing 10% donkey serum for 2 h at room temperature. The sections were incubated for 24 h at 4°C with the following primary antibodies: rabbit anti-ACY1 (A6351, ABclonal)，rabbit anti-Nav1.8 (ASC-016, Alomone)，mouse anti-NeuN (ab104224, Abcam), rabbit anti-SphK1 (10670-1-AP, Proteintech), mouse anti-GFP-Tag (AE012, ABclonal), washed three times, and incubated with Alexa Fluor 594-labeled donkey anti-rabbit secondary antibody (711-515-152, Jackson ImmunoResearch Laboratories, West Grove, PA, USA) and Alexa Fluor 488-labeled donkey anti-mouse secondary antibody (715-485-150, Jackson ImmunoResearch Laboratories) for 2 h. The stained sections were imaged using a fluorescence microscope (DP70, Olympus, Tokyo, Japan).

#### Spinal cord slice preparation and patch-clamp recording

Mice were deeply anesthetized, and the L4–L6 sections of the lumbar spinal cord were rapidly removed and transferred to an ice-cold cutting solution containing 213 mM of sucrose, 3 mM of KCl, 1 mM of NaH2PO4, 0.5 nM of CaCl2, 5 mM of MgCl2, 26 mM of NaHCO3, and 10 mM of glucose. Next, the spinal cord tissue was glued onto a vibratome using an agar block. Several coronal slices (450 μm thick) of the spinal cord were cut in an ice-cold cutting solution using a vibrating microtome (Leica) and incubated in oxygenated artificial cerebrospinal fluid (aCSF) with 95% O_2_ and 5% CO_2_ for >1 h at 34° CRT before recording. The aCSF contained 125 mM of NaCl, 5 mM of KCl, 1.2 mM of NaH2PO4, 2.6 mM of CaCl2, 1.3 mM of MgCl2, 26 mM of NaHCO3, and 10 mM of glucose, with pH of 7.2–7.4 (adjusted osmolarity with sucrose to 310–320 mOsm). All chemicals were obtained from Sigma-Aldrich. After recovery, spinal cord slices were transferred to the recording chamber, which was perfused continuously with oxygenated aCSF at a rate of 3 mL/min at 34°C. Patch electrode recording pipettes (64–10 6 MΩ) were made of borosilicate glass (WPI, Worcester, MA, USA) on a two-step horizontal puller (P-1000, Sutter Health, Sacramento, CA, USA). The recording pipettes were filled with the following internal solution: 145 mM of KCl, 5 mM of NaCl, 10 mM of HEPES, 5 mM of EGTA, 4 mM of Mg-ATP, and 0.3 mM of Na3- GTP, with pH adjusted to 7.3 with KOH. Whole-cell patch-clamp recordings were performed in neurons in the superficial laminae of the spinal dorsal horn because they predominantly receive nociceptive input. Action potential (AP) was recorded using ramp stimulation in a current clamp. The current step increment was 20 pA (from 0 to 200 pA with a pulse duration of 3 ms) to determine the rheobase, and the phasic pattern of stimulation (duration, 500 ms; amplitude, 20 pA) was performed to record the number of APs. The AP signals were acquired using the EPC10 amplifier and Patchmaster software (HEKA, Lambrecht, Germany), filtered, and sampled at 10 kHz with a Bessel filter amplifier. All data obtained were analyzed using the Clampfit software (pClamp10, Molecular Devices, San Jose, CA).

#### Enzyme-linked immunosorbent assay

The expression of sphingosine-1-phosphate (S1P) in spinal cord tissue samples was quantified using a commercially available enzyme-linked immunosorbent assay (ELISA) kit (Echelon Biosciences, Salt Lake City, UT, USA). This procedure was performed according to the manufacturer’s instructions. In brief, spinal cord tissue samples were homogenized in a RIPA lysis buffer containing protease inhibitors (Beyotime Biotechnology) and centrifuged to extract proteins. After incubation with a solid-phase monoclonal antibody and biotin-labeled polyclonal antibody, the streptavidin–peroxidase conjugate was applied to combine the biotinylated antibody. The absorbance of the mixture was detected at 450 nm using a spectrophotometer (Model 680, Bio-Rad). A standard curve was plotted for each experiment, and the concentration of S1P was calculated by comparison with the standard curve.

### Quantification and statistical analysis

All data are expressed as mean ± standard error of the mean. Behavioral data were analyzed using two-way repeated-measures analysis of variance (ANOVA) followed by the Bonferroni post hoc test. Western blot analysis and ELISA data were analyzed using one-way ANOVA followed by the Bonferroni post hoc test. Electrophysiology data were analyzed using one-way ANOVA with the Tukey multiple tests or two-way ANOVA with Bonferroni post hoc comparisons. Statistical analyses were performed using GraphPad Prism, version 7.0 (GraphPad Software, San Diego, CA, USA). Values with p < 0.05 were considered statistically significant.

## Data Availability

• All data reported in this paper can be shared by the lead contact on request. • Any additional information required to reanalyze the data reported in this paper is available from the lead contact upon request. • The original proteomics data have been deposited to the ProteomeXchange Consortium at https://proteomecentral.proteomexchange.org/cgi/GetDataset?ID=PXD042455. • This article does not report original code.

## Supplemental information

Document S1. Figures S1 and Table S1

Data S1. Data file of exported proteomics datasets, related to Figure 1
