# Supplementary material for: A novel co-target of ACY1 governing plasma membrane translocation of SphK1 contributes to inflammatory and neuropathic pain
Source: iScience. 2023 May 28;26(6):106989. doi: 10.1016/j.isci.2023.106989 (PMC10291574; doi:10.1016/j.isci.2023.106989)
Supplement: Document S1. Figures S1 and Table S1 [file mmc1.pdf]

iScience, Volume *26*

Supplemental information

A novel co-target of ACY1 governing plasma membrane translocation of SphK1 contributes to inflammatory and neuropathic pain

Baowen Liu, Wenyao Wu, LingLing Cui, Xuemei Zheng, Ningbo Li, Xianwei Zhang, and Guangyou Duan

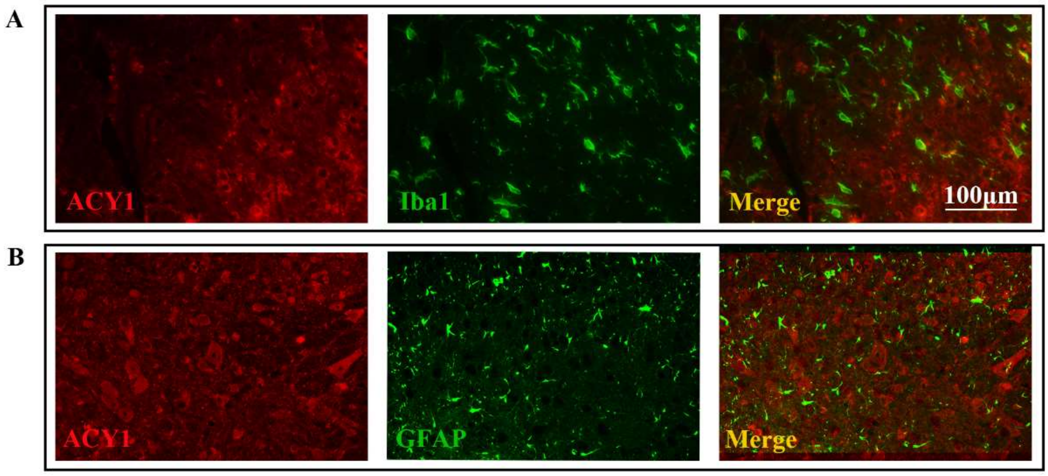

Figure S1. ACY1 was sparsely colocalized with GFAP or Iba1 in the dorsal horn of the spinal dorsal horn, related to Figure 1.

A. Immunofluorescence double staining of ACY1 with the microglial marker IBA1 in the spinal cord. **B.** Representative photomicrographs of ACY1 double fluorescence labeling with astrocyte marker GFAP in the spinal cord. The scale bars represent 100 μm.

Table S1. The sequence of AAV9-Acy1 and AAV9-Acy1-RNAi, related to STAR Methods

ID	Sequence
AAV9-Acy1(68787-1)-p1	TGGAGGTAGTGGAATGGATCTCGCCACCATGACC ACCAAGGATCCCGAGTCTGAG
AAV9-Acy1(68787-1)-p1	TCACCATGGTGGCGGGATCCATGCTTTCACTAGGC AGGGTGGGCAC
AAV9-Acy1-RNAi(102714-13)-1	ACCGCTAGCTAACTGGAGGCTTGCTGAAGGCTGT ATGCTGTTCTGAGCAAACTCAAAGGTGGTTTTGG
AAV9-Acy1-RNAi(102714-13)-2	ACCAAGCTTGGGCCATTTGTTCCATGTGAGTGCTA GTAACAGGCCTTGTGTCCTGTTCTGAG
AAV9-Acy1-RNAi(102714-13)-3	AAACTCAAAGGTGGTTTTGGCCACTGACTGACCA CCTTTGTTTGCTCAGAACAGGACACAAGGC
