# Supplementary material for: A novel co-target of ACY1 governing plasma membrane translocation of SphK1 contributes to inflammatory and neuropathic pain
Source: iScience. 2023 May 28;26(6):106989. doi: 10.1016/j.isci.2023.106989 (PMC10291574; doi:10.1016/j.isci.2023.106989)
Supplement: Data S1. Data file of exported proteomics datasets, related to Figure 1 [file mmc2.zip › Date S1/1-M-GSGC0160906正式实验报告/iTRAQ方法说明文档V1.3.docx]

**等重同位素多标签相对定量蛋白质组学（iTRAQ）方法说明文档**

**V1.3版**

**2018.01**

**目 录**

[1. 实验仪器和分析软件 3](#_Toc504995424)

[2. 试剂和耗材 3](#_Toc504995425)

[3. 实验方法 4](#_Toc504995426)

[3.1 样品制备方法 4](#_Toc504995427)

[3.2 SDS-PAGE电泳 6](#_Toc504995428)

[3.3 FASP酶解^[3]^ 6](#_Toc504995429)

[3.4 iTRAQ标记 6](#_Toc504995430)

[3.5 High PH RP分级 6](#_Toc504995431)

[3.6 质谱分析 7](#_Toc504995432)

[3.7 数据分析 7](#_Toc504995433)

[4. Gene Ontology (GO)功能注释 8](#_Toc504995434)

[5. KEGG通路注释 9](#_Toc504995435)

[6. GO注释与KEGG注释的富集分析 9](#_Toc504995436)

[7. 蛋白质聚类分析(Clustering) 9](#_Toc504995437)

[8. 蛋白质相互作用网路分析（PPI） 9](#_Toc504995438)

[9. 参考文献 10](#_Toc504995439)

1. **实验仪器和分析软件**

| **实验仪器：** | |
| --- | --- |
| - Easy nLC色谱系统 (Thermo Fisher Scientific) - Agilent 1260 infinity II HPLC系统 - 低温高速离心机 (Eppendorf 5430R) - 电泳仪 (BIO-RAD) - 超声破碎仪 (宁波新芝JY96-IIN) - Votex振荡器 (GENIE Votex-2) - Nano Drop（Thermo Fisher scientific ND2000） | - Q Exactive Plus质谱仪 (Thermo Fisher Scientific) - Multiskcan FC酶标仪 (Thermo Fisher Scientific) - 真空离心浓缩仪 (太仓华美LNG-T98) - MP Fastprep-24匀浆仪 (MP Fastprep-24 5G) - 恒温培养箱（上海精宏 GNP-9080） - 电子天平 (OHAUS AX324Z) - 精巧型恒温混匀仪（大龙 HCM-100 pro） |
| **分析软件：** | |
| - Proteome Discoverer 2.1 (Thermo Fisher Scientific) - MASCOT 2.6 (Matrix Science) | - Perseus 1.3 ([Max Planck Institute of Biochemistry](https://en.wikipedia.org/wiki/Max_Planck_Institute_of_Biochemistry) in [Martinsried](https://en.wikipedia.org/wiki/Martinsried), [Germany](https://en.wikipedia.org/wiki/Germany)) - R version 3.0.3 |

1. **试剂和耗材**

| - Urea (BIO-RAD，161-0731) - SDS (生工，SB0485-500g) - Tris (生工，T0826-500g) - 碘乙酰胺 ( IAA , Sigma，I1149-5G) - NH_3_•H_2_O(Sigma, 17837) - C18 Empore™ 固相萃取圆盘（Sigma，66883-U） - BCA定量试剂盒（碧云天，P0012） - NH_4_HCO_3_ (Sigma，A6141-25G) - 甲酸（Thermo Fisher Scientific, A117） - 乙腈 (Merck，1000304008) - C18 Cartridge (Waters，WAT023590) - iTRAQ Reagent‐4/8plex Multiplex Kit (AB SCIEX) | - SDS-PAGE蛋白上样缓冲液（碧云天，P0015F**）** - Lysing Matrix A(MP, 6910-100-99219) - 二硫苏糖醇 (DTT, Sigma， 43819-5G) - HCOONH_4_ (Sigma, 17843) - HCl（国药，10011018） - BSA（生工， A0332） - Trypsin (Promega, V5117) - 三氟乙酸（TFA , Sigma, T6508） - 30kD超滤离心管 (Sartorius, VN01H22) - 0.22 μm超滤离心管（Corning Spin-X, 8160） - Multiple Affinity Removal LC Column – Human 14 / Mouse 3 (Agilent) - Dissolution buffer（AB SCIEX） |
| --- | --- |
| RP 色谱柱：Waters，XBridge Peptide BEH C18 Column, 130Å, 5 µm, 4.6 mm X 100 mm,1/pkg | |
| C18 分析柱：Thermo Fisher Scientific，Acclaim PepMap RSLC 50um X 15cm, nano viper,P/N164943 | |
| SDT 裂解液：4%SDS，100mM Tris-HCl，pH 7.6 | |
| UA buffer：8M Urea，150mM Tris‐HCl，pH 8.5 | |
| High PH RP Buffer A：10mM HCOONH_4_, 5% ACN, pH 10.0 | |
| High PH RP Buffer B：10mM HCOONH_4_, 85% ACN, pH 10.0 | |
| EASY nLC流动相A：0.1%FA | |
| EASY nLC流动相B：0.1% FA, 80% ACN | |

1. **实验方法**
   1. **样品制备方法**

**具体选择以下哪种裂解方法参见项目方案或者预备实验报告。**

1. **TCA/丙酮沉淀+SDT裂解法^[1]^：**

**适用样品类型：植物组织（根、茎、叶等）、动物坚硬组织（皮肤、软骨、毛发等）、真菌**

取适量样本在液氮中用研钵磨碎成细粉状，加入5倍体积的TCA/丙酮（1:9），涡旋混匀，置于-20℃沉淀4h以上。4℃ 6000g离心40min，弃上清。加入预冷丙酮，洗涤三次。通风橱中干燥沉淀。称取20-30mg干燥后的粉末，加入30倍体积（m/v）的SDT裂解液，Votex重悬沉淀，沸水浴5min。超声破碎，沸水浴15min。14000g离心15min，取上清采用0.22µm滤膜过滤，收集滤液。采用BCA法进行蛋白质定量。分装样品，-80℃保存。

1. **匀浆+SDT裂解法^[2]^：**

**适用样品类型：动物柔软组织（脑、肝、肌肉等）、软体动物、微生物菌体等**

取组织或微生物菌体沉淀加入适量SDT裂解液，转移至Lysing Matrix A管中，应用MP匀浆仪进行匀浆破碎（24×2， 6.0M/S，60s，两次）。超声后，沸水浴10min。14000g离心15min，取上清采用0.22µm离心管过滤，收集滤液。采用BCA法进行蛋白质定量。分装样品，-80℃保存。

1. **SDT裂解法^[3]^：**

**适用样品类型：细胞、蛋白质沉淀粉末、体液、浓缩后的发酵液或者细胞分泌上清等。**

取样品加入适量SDT裂解液，超声（溶液类样品此操作可跳过），沸水浴15min。14000g离心15min，取上清。采用BCA法进行蛋白质定量。分装样品，-80℃保存。

1. **去血清高丰度样品处理法：**

**适用样品类型：人、小鼠、大鼠的血清**

取适量样本采用对应物种的去血清高丰度亲和色谱柱Agilent Multiple Affinity Removal LC Column–Human14/Mouse3（人选择Human14柱子,小鼠与大鼠选择Mouse3的柱子）按照Agilent对应protocol**^[4-6]^**中的操作方法去除高丰度蛋白质，得到低丰度组分溶液。应用5kD超滤管进行超滤浓缩，加入一倍体积的SDT裂解液，沸水浴10min，14000g离心15min，取上清。采用BCA法进行蛋白质定量。分装样品，-80℃保存。

- 1. **SDS-PAGE电泳**

各样品取蛋白质20µg分别加入6X上样缓冲液，沸水浴5min，进行12% SDS-PAGE电泳（恒压250V，40min），考马斯亮蓝染色。

- 1. **FASP酶解^[3]^**

各样品取30 μL蛋白质溶液，分别加入DTT至终浓度为100mM，沸水浴5min，冷却至室温。加入200 μL UA buffer混匀，转入30kD超滤离心管中，离心12500g 25min，弃滤液（重复该步骤两次）。加入100μL IAA buffer（100mM IAA in UA），600rpm振荡1min，室温避光反应30min，离心12500g 25min。加入100μL UA buffer离心12500g 15min，重复该步骤两次。加入100μL 10倍稀释的Dissolution buffer，离心12500g 15min，重复该步骤两次。加入40μL Trypsin buffer（4μg Trypsin in 40μL Dissolution buffer），600rpm振荡1min，37℃放置 16-18h。换新收集管，离心12500g 15min；再加入20μL Dissolution buffer，离心12500g 15min，收集滤液。肽段定量（Nano Drop 2000）。

- 1. **iTRAQ标记**

各样品分别取100 μg肽段，按照AB SCIEX公司iTRAQ标记试剂盒说明书^[7]^进行标记。

- 1. **High PH RP分级**

将每组标记后的肽段混合，采用Agilent 1260 infinity II HPLC系统进行分级。缓冲液A液为10mM HCOONH_4_, 5% ACN, pH 10.0，B液为10mM HCOONH_4_, 85% ACN, pH 10.0。色谱柱以A液平衡，样品由手自动进样器上样到色谱柱进行分离，流速为1mL/min。液相梯度如下：0min-25min，B液0%； 25min-30min，B液线性梯度从0%-7%；30min-65min，B液线性梯度从7%-40%；65min-70min，B液线性梯度从40%-100%；70min-85min，B液维持在100%。洗脱过程中监测214nm的吸光度值，每隔1min收集洗脱组分，共计收集洗脱组分约36份。将样品冻干后用0.1%FA复溶合并为N份（N的数目参见项目实验方案）。

- 1. **质谱分析**
     1. **Easy nLC色谱**

每份样品采用纳升流速Easy nLC系统进行分离。缓冲液A液为0.1%甲酸水溶液，B液为0.1%甲酸乙腈水溶液（乙腈为80%）。色谱柱以100%的A液平衡，样品由自动进样器上样到分析柱（Thermo Fisher Scientific，Acclaim PepMap RSLC 50um X 15cm, nano viper,P/N164943）分离，流速为300 nL/min。根据项目实验方案选择相应的液相梯度：

1. 1小时梯度：0min-5min，B液6%；5min-45min，B液线性梯度从6%-28%；45min-50min，B液线性梯度从28%-38%；50min-55min，B液线性梯度从38%-100%；55min-60min，B液维持在100%。
2. 2小时梯度：0min-5min，B液6%；5min-105min，B液线性梯度从6%-28%；105min-110min，B液线性梯度从28%-38%；110min-115min，B液线性梯度从38%-100%；115min-120min，B液维持在100%。
   - 1. **质谱鉴定**

样品经色谱分离后用Q-Exactive Plus 质谱仪进行质谱分析。分析时长为60/120min(根据具体实验方案而定)，检测方式为正离子，母离子扫描范围350-1800 *m*/*z*，一级质谱分辨率为70,000，AGC target为3e6，一级Maximum IT为50ms。多肽和多肽碎片的质量电荷比按照下列方法采集：每次全扫描（full scan）后采集10个碎片图谱（MS2 scan），MS2 Activation Type为HCD，Isolation window为2 *m/z*，二级质谱分辨率17,500， Microscans为1，二级Maximum IT为45ms，Normalized Collision Energy为30eV。

- 1. **数据分析**

质谱分析原始数据为raw文件，用软件Mascot2.6和Proteome Discoverer2.1^[8]^进行查库鉴定及定量分析。相关参数和说明如下:

| Item | Value |
| --- | --- |
| - Protein Database（查库所使用的数据库） | - 公共库命名如uniprot_mouse（物种）_20141212（下载日期）.fasta；自建库一般命名为项目号。具体数据库信息见项目报告。 |
| - Enzyme | - Trypsin |
| - Max Missed Cleavages   （允许的最大漏切位点数目） | - 2 |
| - Instrument | - ESI-TRAP |
| - Precursor Mass Tolerance(一级离子质量容差) | - ± 20 ppm |
| - Fragment Mass Tolerance(二级离子质量容差) | - 0.1Da |
| - Use Average Precursor Mass | - False |
| - Modification Groups   From Quan Method | - iTRAQ 4/8plex(据具体项目而定) |
| - Dynamic modifications(可变修饰) | - Oxidation (M),Acetyl (Protein N-term), - Deamidated (NQ) |
| - Static modifications(固定修饰) | - Carbamidomethyl (C), |
| - Database pattern（用于计算FDR的数据库模式） | - decoy |
| - Peptide FDR（可信蛋白质的筛选标准） | - ≤0.01 |

1. **Gene Ontology (GO)功能注释**

利用Blast2GO^[9]^对目标蛋白质集合进行GO注释的过程大致可以归纳为序列比对（Blast）、GO条目提取（Mapping）、GO注释（Annotation）和补充注释（Annotation Augmentation）等四个步骤。

首先，利用本地化序列比对工具NCBI BLAST+ (ncbi-blast-2.2.28+-win32.exe) 将目标蛋白质集合与适当的蛋白质序列数据库进行比对，并保留满足E-value<=1*e*-3的前10条比对序列进行后续分析。其次，利用Blast2GO Command Line对目标蛋白质集合及Blast保留结果中Bit-Score最高的比对序列所关联的GO条目进行提取（数据库版本：go_201504.obo，下载地址：[www.geneontology.org](http://www.geneontology.org)）。在Annotation过程中，Blast2GO Command Line通过综合考量目标蛋白质序列和比对序列的相似性、GO条目来源的可靠度，以及GO有向无环图的结构，将Mapping过程中提取的GO条目注释给目标蛋白质序列。完成Annotation后，为进一步提高注释效率，可以通过InterProScan^[10]^搜索EBI数据库中与目标蛋白质匹配的保守基序（motif），并将motif相关的功能信息注释给目标蛋白质序列；并运行ANNEX对注释信息进一步补充，并在不同的GO类别之间建立联系，以提高注释的准确性。

1. **KEGG通路注释**

在KEGG数据库中，KO (KEGG Orthology) 是一个基因及其产物的分类体系。在同一条通路上具有相似功能的直系同源基因及其产物被归为一组，并赋予同一个KO（或者K）标签。对目标蛋白质集合进行KEGG通路注释时，利用KAAS (KEGG Automatic Annotation Server) 软件^[11]^，首先通过比对KEGG GENES数据库，将目标蛋白质序列进行KO归类，并根据KO归类自动获取目标蛋白质序列参与的通路信息。

1. **GO注释与KEGG注释的富集分析**

在对目标蛋白质集合进行GO注释或KEGG通路注释的富集分析时，通过Fisher精确检验（Fisher’s Exact Test），比较各个GO分类或KEGG通路在目标蛋白质集合和总体蛋白质集合中的分布情况，来评价某个GO term或KEGG通路蛋白质富集度的显著性水平。

1. **蛋白质聚类分析(Clustering)**

进行聚类分析时，首先对目标蛋白质集合的定量信息进行归一化处理（归一化到（-1,1）区间）。其次，使用Cluster 3.0软件同时对样品和蛋白质的表达量两个维度进行分类（距离算法：欧几里得，连接方式：Average linkage）。最后，使用Java Trewview软件生成层次聚类热图。

1. **蛋白质相互作用网路分析（PPI）**

首先从目标蛋白质序列来源的数据库中获取目标蛋白质的Gene Symbol，利用这些Gene Symbol在IntAct (<http://www.ebi.ac.uk/intact/main.xhtml>) 数据库中查找有实验证据的目标蛋白质之间的直接和间接相互作用关系，并使用CytoScape软件（版本号：3.2.1）生成相互作用网络并对网络进行分析。

1. **参考文献**
2. [Plant Proteomics: Methods and Protocols](http://www.smarter.com.cn/redir.php?bt=b2ZmZXI%3D&ch=1011&oi=28025221&mc=1&dp=1&pr=0&rr=0&sb=&cb=). Hervé Thiellement, Michel Zivy, Catherine Damerval, and Valerie Mechin, 2007. METHODS IN MOLECULAR BIOLOGY 355.
3. Proteomic analysis of solid pseudopapillary tumor of the pancreas reveals dysfunction of the endoplasmic reticulum protein processing pathway. Zhu Y et al. Mol Cell Proteomics.2014. 13(10):2593-603.
4. Universal sample preparation method for proteome analysis. Wisniewski, J. R., A. Zougman, et al. Nat Methods.2009. 6(5): 359-362.
5. Agilent Human 14 Multiple Affinity Removal System Columns for the Fractionation of High-Abundant Proteins from Human Proteomic Samples. Agilent Technologies, Inc. 2007.
6. Agilent Multiple Affinity Removal Columns – for Mouse Serum Proteins. Agilent Technologies, Inc. 2005.
7. Immunodepletion of High-Abundant Proteins from Rat Serum with the Agilent Multiple Affinity Removal System for Mouse. Agilent Technologies, Inc. 2004.
8. Applied Biosystems iTRAQ™ Reagents Amine-Modifying Labeling Reagents for Multiplexed Relative and Absolute Protein. Applied Biosystems. 2004.
9. Proteome Discoverer Version 2.1. Thermo Fisher Scientific Inc. 2014.
10. High-throughput functional annotation and data mining with the Blast2GO suite. Götz S, García-Gómez JM, et al. Nucleic Acids Res. 2008; 36(10): 3420-35.
11. InterProScan: protein domains identifier. Quevillon E, Silventoinen V, et al. Nucleic Acids Res. 2005; 33(Web Server issue): W116-20.
12. KAAS: an automatic genome annotation and pathway reconstruction server. Moriya Y, Itoh M, Okuda S, Yoshizawa AC, Kanehisa M. Nucleic Acids Res. 2007 Jul;35(Web Server issue):W182-5.
