# Supplementary material for: A novel co-target of ACY1 governing plasma membrane translocation of SphK1 contributes to inflammatory and neuropathic pain
Source: iScience. 2023 May 28;26(6):106989. doi: 10.1016/j.isci.2023.106989 (PMC10291574; doi:10.1016/j.isci.2023.106989)
Supplement: Data S1. Data file of exported proteomics datasets, related to Figure 1 [file mmc2.zip › Date S1/1-M-GSGC0160906正式实验报告/iTRAQ结果说明V1.6.docx]

**等重同位素多标签相对定量蛋白质组学**

**（iTRAQ）结果说明**

**V1.6版**

**吉凯基因**

**目 录**

[附件1. 蛋白质鉴定列表 3](#_Toc505074889)

[附件2. 肽段鉴定列表 3](#_Toc505074890)

[附件3. 蛋白质定量和差异分析列表 4](#_Toc505074891)

[附件4：GO分析结果 6](#_Toc505074892)

[**GO功能注释统计表** 6](#_Toc505074893)

[附件5：KEGG分析结果 7](#_Toc505074894)

[**KEGG通路注释统计表** 7](#_Toc505074895)

[**Map文件夹** 8](#_Toc505074896)

[附件6：聚类分析结果 8](#_Toc505074897)

[附件7：PPI分析结果 9](#_Toc505074898)

[参考文献 9](#_Toc505074899)

**附件1. 蛋白质鉴定列表**

| 表头 | 定义 | 描述 |
| --- | --- | --- |
| Accession | 蛋白质登录号 | 蛋白质序列数据库（FASTA database）中的蛋白质编号 |
| Gene Name | 基因名 | 显示Fasta header列中注释的基因名称，如果数据库中的注释信息不完整或没有基因名称，则不显示该内容。 |
| Description | 蛋白质信息描述 | 基于蛋白质序列的数据库中的蛋白质功能描述。 |
| Coverage | 肽段覆盖率 | 鉴定到的氨基酸数目占蛋白质总氨基酸数目的比例。The percentage of the protein sequence covered by identified peptides |
| Peptides | 肽段数 | The number of distinct peptide sequences in the protein group （该蛋白质（组）的所有鉴定肽段数目） |
| PSMs | 匹配到肽段的质谱图谱总数 | 全称是peptide spectrum matches，为该蛋白质组的所有肽段匹配到全部质谱图的数量。The total number of identified peptide sequences for the protein, including those redundantly identified. |
| Unique Peptides | 唯一肽段数 | The number of peptide sequences unique to a protein group （该蛋白质（组）的特有肽段数目） |
| AAs | 氨基酸个数 | 蛋白质的氨基酸总数 |
| MW [kDa] | 分子量 | 蛋白质的理论分子量。该分子量是软件根据数据库中的蛋白质序列计算得到的。如果用以参加计算的蛋白质序列不是完整的全长序列，比如由转录组翻译而来的蛋白质序列，由此计算得到的分子量会小于完整蛋白质的分子量。 |
| calc. pI | 等电点 | 蛋白质的理论等电点 |
| Abundances XXX | 蛋白丰度 | 对应样品中蛋白的相对表达量 |

**附件2. 肽段鉴定列表**

| **表头** | **定义** | **描述** |
| --- | --- | --- |
| Sequence | 肽段氨基酸序列 | 描述肽段氨基酸的组成 |
| Modifications | 修饰 | 描述修饰氨基酸、位置及修饰方式。如： C(Carbamidomethyl)：半胱氨酸修饰；M(Oxidation)：甲硫氨酸氧化；S/T/Y（Phosphorylation）：丝氨酸/苏氨酸/酪氨酸磷酸化 |
| Qvality PEP | 后验错误概率 | The posterior error probability (PEP) is the probability that the observed PSM is incorrect. 相当于局部的FDR（Local FDR）。This value essentially operates as a p-value, where smaller is better. For example, if the PEP associated with (EAMRPK, *s*) is 5 percent, there is a 95 percent chance that the EAMRPK peptide was in the mass spectrometer when spectrum *s* was generated. The FDR measures the error rate associated with a collection of PSMs, and the PEP measures the probability of error for a single PSM. |
| Qvality q-value | 最小的错误发生率 | A q-value is the minimal false discovery rate at which the identification is considered correct. 相当于全局的FDR （Global FDR），报告结果给出的所有鉴定数据都满足FDR<0.01的筛选标准. q-values are estimated using the distribution of scores from the decoy database search. A q-value of 0.01 for the EAMRQPK peptide matching spectrum, *s*, means that if you try all possible FDR thresholds, 1 percent is the minimal FDR threshold at which the PSM of EAMRQPK to *s* appears in the output list. Although the q-value is associated with a single PSM, it also depends on the data set that the PSM occurs in. |
| PSMs | 匹配到肽段的质谱图谱总数 | 全称是peptide spectrum matches，为该肽段匹配到全部质谱图的数量。The total number of identified peptide sequences for the protein, including those redundantly identified. |
| Master Protein Accessions | 蛋白质登录号 | 蛋白质序列数据库（FASTA database）中的蛋白质编号 |
| Missed Cleavages | 漏切位点数量 | 鉴定肽段序列中含有胰蛋白酶漏切位点（即K和R）的数量 |
| Theo. MH+ [Da] | 肽段分子量 | 带一个电荷（质子化）的肽段理论分子量 |
| Abundances XXX | 肽段丰度 | 对应样品中肽段的相对丰度 |
| Ions Score Mascot | 肽段得分 | MASCOT肽段得分 |
| Charge Mascot | 电荷 | 肽段电荷数 |
| DeltaM [ppm] Mascot | 理论分子量和实验分子量的差值 | 肽段理论分子量和实验测得分子量的差异 |
| RT [min] Mascot | 保留时间 | 全称为retention time，指被分离样品组分从进样开始到柱后出现该组分浓度极大值时的时间，即从进样开始到出现某组分色谱峰的顶点时为止所经历的时间，称为此组分的保留时间，用RT表示，常以分（min）为时间单位。 |

**附件3. 蛋白质定量和差异分析列表**

| **包含表格** | **表格说明** | |
| --- | --- | --- |
| Sheet 各比较组定量分析拆分表格（表格数目等于比较组数） | 包含标题对应比较组的相对表达量比值和P-value。差异表达蛋白质采用红色标识上调差异表达蛋白质，绿色标识下调差异表达蛋白质。 | |
| **表头** | **定义** | **描述** |
| Accession | 蛋白质登录号 | 蛋白质序列数据库（FASTA database）中的蛋白质编号 |
| Gene Name | 基因名 | 显示Fasta header列中注释的基因名称，如果数据库中的注释信息不完整或没有基因名称，则不显示该内容。 |
| Description | 蛋白质信息描述 | 基于蛋白质序列的数据库中的蛋白质功能描述。 |
| Coverage | 肽段覆盖率 | 鉴定到的氨基酸数目占蛋白质总氨基酸数目的比例。The percentage of the protein sequence covered by identified peptides |
| Peptides | 肽段数 | The number of distinct peptide sequences in the protein group （该蛋白质（组）的所有鉴定肽段数目） |
| PSMs | 匹配到肽段的质谱图谱总数 | 全称是peptide spectrum matches，为该蛋白质组的所有肽段匹配到全部质谱图的数量。The total number of identified peptide sequences for the protein, including those redundantly identified. |
| Unique Peptides | 唯一肽段数 | The number of peptide sequences unique to a protein group （该蛋白质（组）的特有肽段数目） |
| AAs | 氨基酸个数 | 蛋白质的氨基酸总数 |
| MW [kDa] | 分子量 | 蛋白质的理论分子量。该分子量是软件根据数据库中的蛋白质序列计算得到的。如果用以参加计算的蛋白质序列不是完整的全长序列，比如由转录组翻译而来的蛋白质序列，由此计算得到的分子量会小于完整蛋白质的分子量。 |
| calc. pI | 等电点 | 蛋白质的理论等电点 |
| Abundances XXX | 蛋白丰度 | 对应样品中蛋白的相对表达量 |
| Average XXX | 对应样品组蛋白质丰度平均值 | 对应组别蛋白的平均相对表达量 |
| XXX/YYY | 组间蛋白表达差异倍数 | 蛋白质在XXX组样品中相对表达量的平均值与在YYY组样品中相对表达量的平均值的比值 |
| P value | 两组样品间的蛋白质相对表达量的统计学检验 | 对于P value的两种常用计算方法适用情况如下：   1. t-test算法：适用于每组样品至少包括三次及以上生物学重复或者技术重复的实验设计 2. significance A算法^[1]^: 适用于每组样品无重复或只有两次生物学重复或者技术重复的实验设计 |
| FDR | False Discovery Rate（错误发现率） | 是多重假设检验中的错误控制指标，是对P-value的Benjamini-Hochberg校正（不需要对P值进行校正的项目不给出该数据） |

**附件4：GO分析结果**

**GO功能注释统计表**

| **表头** | **定义及描述** |
| --- | --- |
| **Sheet TopBlastHits** | |
| Sequence name | 目标蛋白质ID |
| Sequence desc. | 根据序列比对结果推测的目标蛋白质可能的名称和描述 |
| Sequence length | 目标蛋白质序列长度 |
| Hit desc. | 比对序列的蛋白质名称和描述 |
| Hit ACC | 比对序列的蛋白质ID号 |
| E-Value | 比对结果可靠性评价，表示在搜索同样大小的数据库时，随机得到相似或更高的比对分数（Score）的序列数，越低越好 |
| Similarity | Positives/Alignment |
| Bit-Score | 标准化的比对分数，用来评价比对质量的高低，分数越高表明两序列的相似程度越高 |
| Alignment length | 比对区段的氨基酸序列长度（含Gap） |
| Positives | 一致的和理化性质相似的氨基酸数目 |
| **Sheet protein2GO** | |
| SeqName | 目标蛋白质ID |
| Hit-Desc | 比对序列的蛋白质名称和描述 |
| GO-Group | 所注释GO term的类别（P: Biology Process; F: Molecular Function; C: Cellular Component） |
| GO-ID | 所注释GO term的ID |
| Term | 所注释GO term的名称 |
| **Sheet BP / MF / CC** | |
| Level | GO term在ontologies的树形分支结构中所处的层次：Level数字越小表示层次越高，注释的内容概括性越强；Level数字越大表示注释的内容越明确 |
| GO ID | 所注释GO term的ID |
| GO Name | 所注释GO term的名称 |
| GO Type | GO term所属类别（Biology Process, Molecular Function, Cellular Component） |
| #Seqs | 与该GO term相关的蛋白质数目 |
| Sequence Names | 与该GO term相关的蛋白质ID |
| **Sheet Enrichment** | |
| GO-ID | 所注释GO term的ID |
| Term | 所注释GO term的名称 |
| Category | 所注释GO term的类别  P: Biology Process; F: Molecular Function; C: Cellular Component |
| #Diff | 目标蛋白质集合（通常为差异蛋白质集合）中与该GO term相关的蛋白质数目 |
| #Ref | 背景蛋白质集合（通常为所有定性蛋白质集合）中与该GO term相关的蛋白质数目 |
| %Diff | 目标蛋白质集合（通常为差异蛋白质集合）中与该GO term相关的蛋白质比例 |
| %Ref | 背景蛋白质集合（通常为所有定性蛋白质集合）中与该GO term相关的蛋白质比例 |
| Over/Under | 相对与该GO term相关的背景蛋白质比例，与该GO term相关的目标蛋白质比例偏高（%Test>%Ref）或者偏低（%Test<%Ref），通常只保留在目标蛋白质集合中Over-represented GO term |
| P-Value | 富集分析的显著性指标，P-value越小表示该GO term在特定的生物学处理下所受的影响越显著，由超几何分布计算 |
| FDR | False Discovery Rate，译为错误发现率，是多重假设检验中的错误控制指标，是对P-value的Benjamini-Hochberg校正 |
| TestSeqs | 目标蛋白质集合（通常为差异蛋白质集合）中与该GO term相关的蛋白质ID |

**附件5：KEGG分析结果**

**KEGG通路注释统计表**

| **表头** | **定义及描述** |
| --- | --- |
| **Sheet query2map** | |
| Protein ID | 目标蛋白质ID |
| KO | 目标蛋白质在KEGG数据库中对应的蛋白质ID |
| Name | 目标蛋白质在KEGG数据库中的简称（多与通路中蛋白质节点显示的名称相同） |
| Definition | 目标蛋白质在KEGG数据库中的名称 |
| Map ID | 目标蛋白质可能参与的通路ID |
| Map Name | 目标蛋白质可能参与的通路名称 |
| URL | 可直接链接到KEGG数据库中目标蛋白质可能参与的通路图，参与该通路的所有目标蛋白质以红色框线和字体标识 |
| **Sheet map2query** | |
| Map ID | 目标蛋白质可能参与的通路ID |
| Map Name | 目标蛋白质可能参与的通路名称 |
| Seqs | 参与该通路的目标蛋白质ID |
| #Seqs | 参与该通路的目标蛋白质数目 |
| URL | 可直接链接到KEGG数据库中目标蛋白质可能参与的通路图，参与该通路的所有目标蛋白质以红色框线和字体标识 |
| **Sheet Enrichment** | |
| Map ID | 所注释KEGG通路的ID |
| Map Name | 所注释KEGG通路的名称 |
| #Diff | 目标蛋白质集合（通常为差异蛋白质集合）中与该通路相关的蛋白质数目 |
| #Ref | 背景蛋白质集合（通常为所有定性蛋白质集合）中与该通路相关的蛋白质数目 |
| %Test | 目标蛋白质集合（通常为差异蛋白质集合）中与该通路相关的蛋白质比例 |
| %Ref | 背景蛋白质集合（通常为所有定性蛋白质集合）中与该通路相关的蛋白质比例 |
| Over/Under | 相对与该GO term相关的背景蛋白质比例，与该GO term相关的目标蛋白质比例偏高（%Test>%Ref）或者偏低（%Test<%Ref），通常只保留在目标蛋白质集合中Over-represented GO term |
| P_value | 富集分析的显著性指标，P-value越小表示该GO term在特定的生物学处理下所受的影响越显著，由超几何分布计算 |
| FDR | False Discovery Rate，译为错误发现率，是多重假设检验中的错误控制指标，是对P-value的Benjamini-Hochberg校正 |
| DiffSeqs | 目标蛋白质集合（通常为差异蛋白质集合）中与该通路相关的蛋白质ID |

**Map文件夹**

| **结果** | **定义及描述** |
| --- | --- |
| **koXXXXX** | 图片格式，目标蛋白质相关的KEGG通路图，文件名为通路ID。绿色标注的节点为目标蛋白质。 |

**附件6：聚类分析结果**

| **文件** | **定义及描述** |
| --- | --- |
| **cluster** | 图片格式，层次聚类分析结果，热图全图 |
| **cluster_SmallSized** | 图片格式，蛋白质数据较多时，将行高压缩至较小尺寸的热图全图，便于展示 |
| **cluster_colorbar** | 图片格式，色轴，显示蛋白质在各组样品中相对上下调的程度，红色代表上调，蓝色代表下调，白色代表无明显变化 |
| **cluster_list** | TXT格式，与热图中显示的蛋白质顺序一致的蛋白质ID list |

**附件7：PPI分析结果**

| **文件** | **定义及描述** |
| --- | --- |
| **dPPI（direct PPI）** | 图片格式，目标蛋白质之间直接的相互作用关系网络 |
| **PPI** | 图片格式，目标蛋白质的相互作用关系网络，黄色节点为目标蛋白质，蓝色节点为与目标蛋白质直接相互作用的其他蛋白质 |
| **PPI.cys** | 蛋白质相互作用网络分析CytoScape原始文件 |
| **Protein-Gene.list** | TXT格式，目标蛋白质ID号与对应的Gene Symbol列表 |

**参考文献**

1. Cox J, Mann M. MaxQuant enables high peptide identification rates, individualized p.p.b.-range mass accuracies and proteome-wide protein quantification. Nat Biotechnol. 2008; 26(12): 1367-1372.
