# Supplementary material for: A novel co-target of ACY1 governing plasma membrane translocation of SphK1 contributes to inflammatory and neuropathic pain
Source: iScience. 2023 May 28;26(6):106989. doi: 10.1016/j.isci.2023.106989 (PMC10291574; doi:10.1016/j.isci.2023.106989)
Supplement: Data S1. Data file of exported proteomics datasets, related to Figure 1 [file mmc2.zip › Date S1/1-M-GSGC0160906正式实验报告/M-GSGC0160906正式实验报告.docx]

**合同编号：M-GSGC0160906**

**等重同位素多标签相对定量蛋白质组学**

**（iTRAQ）分析报告**

**吉凯基因**

**目 录**

[1. 项目结果展示 3](#_Toc515525707)

[**1.1** **样本标记相关信息** 3](#_Toc515525708)

[**1.2** **蛋白质定性结果展示** 3](#_Toc515525709)

[**1.3** **蛋白质定量结果统计** 3](#_Toc515525710)

[2. 项目概述 3](#_Toc515525711)

[**2.1** **iTRAQ原理** 3](#_Toc515525712)

[**2.2** **项目流程** 5](#_Toc515525713)

[**2.3** **数据分析流程** 6](#_Toc515525714)

[3. 质谱原始数据处理 6](#_Toc515525715)

[**3.1** **质谱raw文件处理** 6](#_Toc515525716)

[**3.2** **数据库选择** 6](#_Toc515525717)

[**3.3** **蛋白质定性和定量分析参数** 7](#_Toc515525718)

[4. 质谱鉴定和定量结果评估 8](#_Toc515525719)

[**4.1** **肽段离子得分分布** 8](#_Toc515525720)

[**4.2** **蛋白质相对分子质量分布** 8](#_Toc515525721)

[**4.3** **蛋白质等电点分布** 9](#_Toc515525722)

[**4.4** **肽段序列长度分布** 10](#_Toc515525723)

[**4.5** **蛋白序列覆盖度分布** 10](#_Toc515525724)

[**4.6** **鉴定肽段数量分布** 11](#_Toc515525725)

[**4.7** **蛋白质丰度比分布** 12](#_Toc515525726)

[**4.8** **火山图（Volcano plot）** 12](#_Toc515525727)

[5. 生物信息分析内容 13](#_Toc515525728)

[**5.1** **Gene Ontology (GO) 功能注释** 13](#_Toc515525729)

[**5.2** **差异表达蛋白质GO富集分析** 15](#_Toc515525730)

[**5.3** **KEGG通路注释** 16](#_Toc515525731)

[**5.4** **差异表达蛋白质KEGG通路富集分析** 18](#_Toc515525732)

[**5.5** **蛋白质聚类分析（Clustering）** 18](#_Toc515525733)

[6. 参考文献 19](#_Toc515525734)

[7. 输出文件及保存位置 19](#_Toc515525735)

1. **项目结果展示**
   1. **样本标记相关信息**

表1-1 样本标记信息

| **iTRAQ标记** | **113** | **115** | **116** | **117** | **118** | **121** | **No.** |
| --- | --- | --- | --- | --- | --- | --- | --- |
| **样本组别** | 病理组 | | | 对照组 | | |  |
| **样本名称** | SP 1.8-/- 2  SP 1.8-/- 3 | SP 1.8-/- 4  SP 1.8-/- 5 | SP 1.8-/- 6  SP 1.8-/- 7 | SNI-1、SNI-2、SNI-3 | SNI-4、SNI-5、SNI-6 | SNI-7、SNI-8、SNI-9 |  |
| **样本编号** | A1 | A2 | A3 | B1 | B2 | B3 | **1** |

**备注：No.：**多个8-plex实验的标记实验序号。

- 1. **蛋白质定性结果展示**

表1-2 蛋白质鉴定结果统计

| **Database** | **No.** | **Total spectra** | **Spectra (PSM)** | **Peptides** | **Unique peptides** | **Protein groups** |
| --- | --- | --- | --- | --- | --- | --- |
| MusMusculus | 1 | 247549 | 93835 | 45702 | 33700 | 5265 |

**备注：Database：**选择使用的数据库物种名称；**No.：**标记实验序号；**Total spectra：**二级质谱谱图总数；**Spectra（PSM, Peptide Spectrum Match）：**鉴定肽段匹配到的谱图数； **Peptides：**鉴定到的肽段总数；**Unique peptides：**鉴定到的唯一肽段总数；**Protein groups：**鉴定到的蛋白质总数。

输出文件：

1. 质谱鉴定和定量结果文件夹\\附件1_蛋白质鉴定列表
2. 质谱鉴定和定量结果文件夹\\附件2_肽段鉴定列表
   1. **蛋白质定量结果统计**

符合表达差异倍数大于1.2倍（上下调）且P value（t test）小于0.05筛选标准的蛋白质视为差异表达蛋白质。

表1-3 蛋白质定量结果统计

| **Comparisons** | **Up-** | **Down-** | **All-** |
| --- | --- | --- | --- |
| A/B | 23 | 35 | 58 |

**备注：Comparisons：**差异比较组；**Up-：**上调差异表达蛋白质；**Down-：**下调差异表达蛋白质；**All：**所有差异表达蛋白质。

输出文件：

1. 质谱鉴定和定量结果文件夹\\附件3_蛋白质定量和差异分析列表（红色表示上调，绿色表示下调）
2. **项目概述**
   1. **iTRAQ原理**

iTRAQ (isobaric tags for relative and absolute quantitation)技术是由AB SCIEX公司研发的一种基于体外等重同位素标记的相对与绝对定量技术。该技术利用4种或8种同位素试剂标记多肽末端氨基或赖氨酸侧链氨基基团，经高分辨质谱仪串联分析，可同时比较多达8种样品之间的蛋白质表达量，是近年来定量蛋白质组学常用的高通量筛选技术^[1]^。


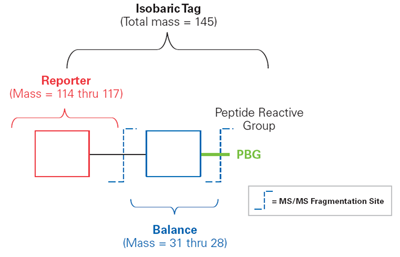


图2-1-1 iTRAQ试剂结构（以4标iTRAQ试剂为例）

iTRAQ试剂由三部分组成：报告基团（4标实验相对分子质量分别为114、115、116和117；8标实验相对分子质量分别为113、114、115、116、117、118、119和121），质量平衡基团（4标实验相对分子质量分别为31、30、29 和28；8标实验相对分子质量分别为192、191、190、189、188、187、186和184）和肽反应标记试剂基团。其中每一种报告基团及其对应的平衡基团的总分子质量均为145Da（4标试剂）或者305Da（8标试剂）。

图2-1-2 iTRAQ试剂标记肽段

iTRAQ试剂是可与氨基酸末端氨基及赖氨酸侧链氨基连接的胺标记等重元素(isobaric)。在质谱图中，任何一种iTRAQ试剂标记的不同样本中同一肽段表现为相同的质荷比。在串联质谱中，报告基团、质量平衡基团和多肽反应基团之间的键断裂，质量平衡基团丢失，带不同同位素标签的同一多肽产生4种或8种不同质量的报告离子（图2-1-3中红色框标记），根据报告离子的丰度可获得样品间相同肽段的定量信息，再经过软件处理得到蛋白质的定量信息。


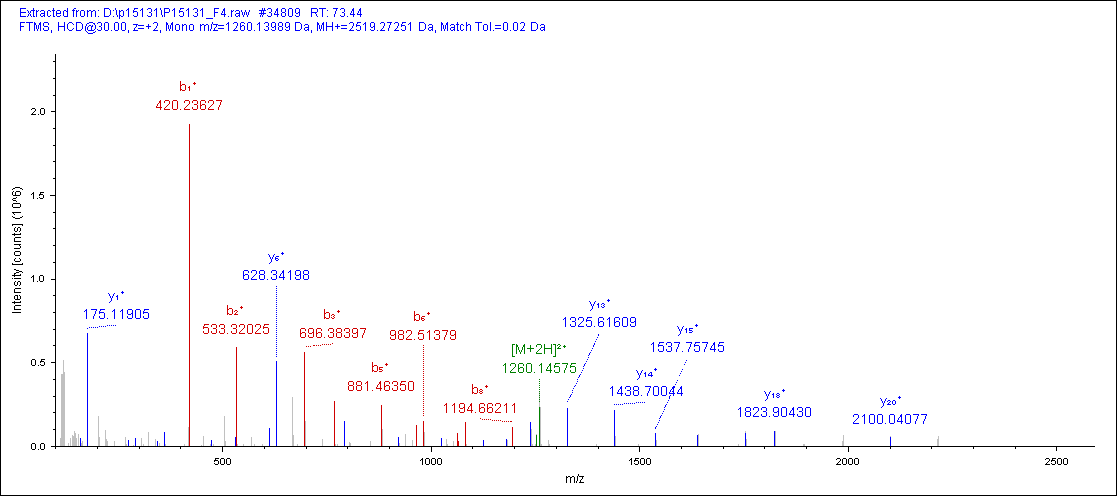

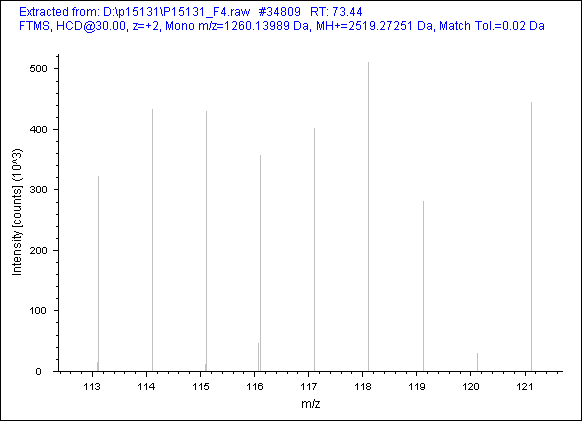


图2-1-3 iTRAQ实验中某肽段的二级质谱图示例

- 1. **项目流程**


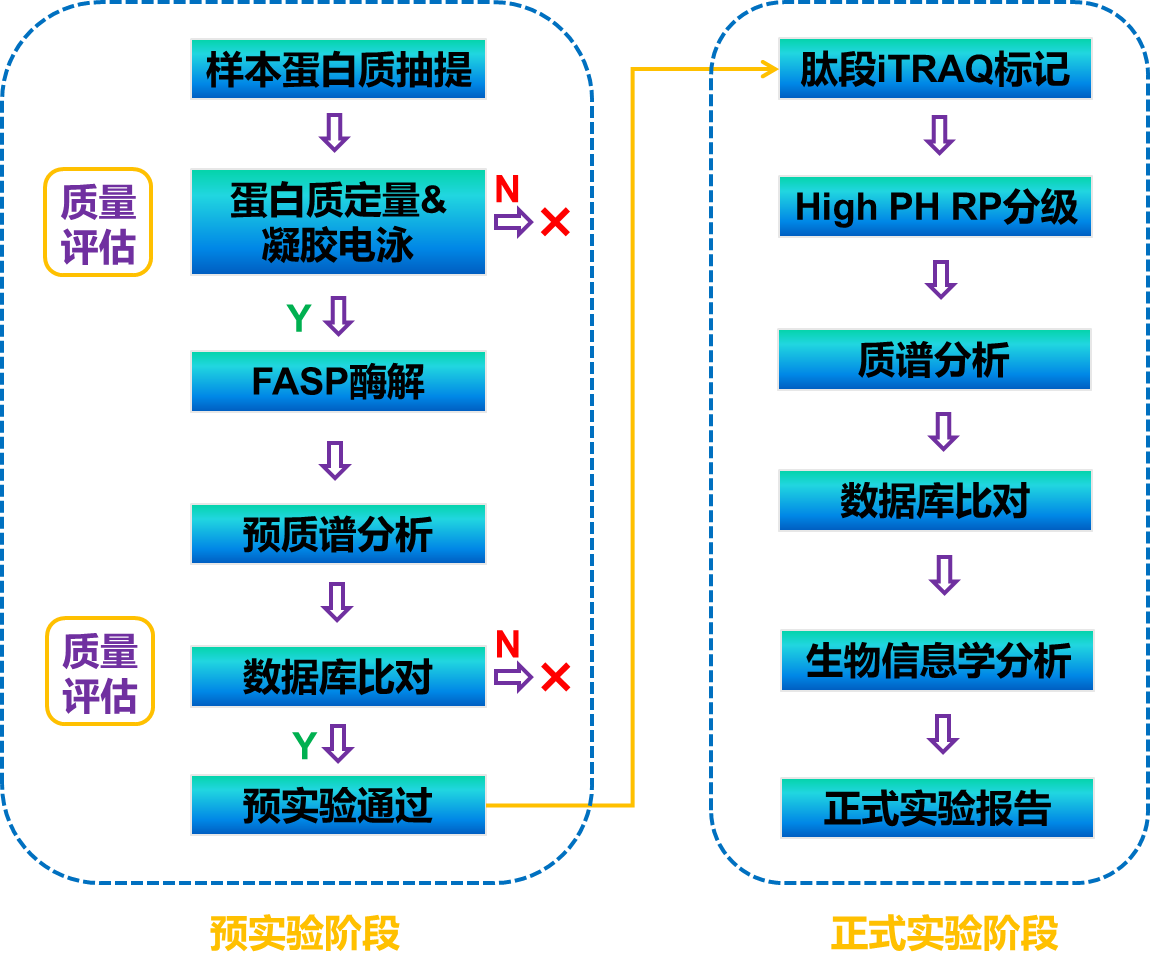


图2-2 吉凯基因iTRAQ分析项目流程图

**备注：**iTRAQ技术可以在一次实验中同时对多达8组样品的差异表达蛋白质进行分析，本项目开展分为**预实验**和**正式实验**两部分。**预实验**包括蛋白质提取、蛋白质定量跑胶、蛋白质酶解、预质谱分析、数据库比对、以及出具预实验报告等步骤。其中，**蛋白质定量跑胶**以及**预质谱数据库比对结果**为重要**质量控制点**。**正式实验**是在预实验的基础上进行，对预实验中质控合格的样本继续进行iTRAQ肽段标记、分级、质谱分析，数据库比对，生物信息学分析并且出具最后的实验报告。

预实验的目的在于：1）质控和评价蛋白质质量和样本间的蛋白质表达谱平行性；2）质控和评价蛋白质总量和浓度；3）质控和评价用于查询的蛋白质序列数据库选取是否合理（该评价对于没有基因组测序的物种尤其重要）。

- 1. **数据分析流程**


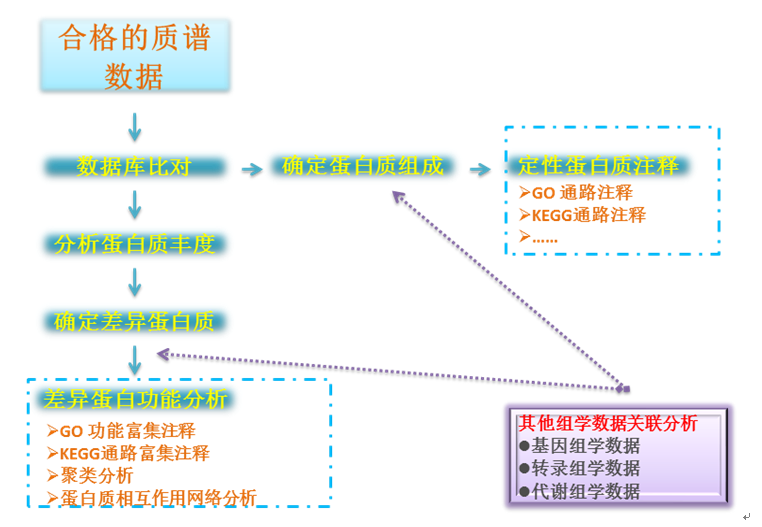


图2-3 iTRAQ定量蛋白质组学数据分析流程图

**备注：**在iTRAQ数据分析过程中，通常首先对质谱原始数据进行数据库查询和结果评价，并对质量控制合格的数据进行后续分析，包括可信肽段及蛋白质的鉴定和筛选、蛋白质定量分析和差异表达蛋白质筛选，以及根据客户需求进行基础或者高级的生物信息学分析等。

1. **质谱原始数据处理**
   1. **质谱raw文件处理**

本项目使用高分辨质谱仪Q Exactive Plus（Thermo Fisher Scientific）进行iTRAQ定量蛋白质组学分析。Q Exactive Plus是Orbitrap类型质谱仪，使用快速HCD模式 （Higher Energy Collisional Dissociation）获得MS2图谱，可改善低质量数离子的传递，从而提高灵敏度和定量性能，尤其适用于使用同位素标签的实验。同时，Q Exactive Plus提供强大的蛋白质组学鉴定能力和丰富的数据处理软件群，为提供完整的定量蛋白质组学数据打下坚实的基础。

本项目使用Proteome Discoverer 2.1（Thermo Fisher Scientific）软件^[2]^将Q Exactive Plus产生的原始图谱文件(.raw文件)转化为.mgf文件，通过软件内置的工具提交到MASCOT2.6服务器进行数据库检索。然后再通过Proteome Discoverer 2.1将MASCOT服务器上形成的查库文件（.dat文件）传回软件，根据FDR<0.01的标准对数据进行筛选，获得高度可信的定性结果。

- 1. **数据库选择**

选择适当的蛋白质序列数据库是对质谱数据进行蛋白质定性分析的基础和关键步骤。通常数据库建立的数据来源主要有以下几种：

1. 综合性蛋白质数据库，如NCBInr、UniProt等；
2. 特定物种的蛋白质数据库，如拟南芥（TAIR）、水稻（RAP DB）、家蚕（silkdb）等；
3. 已测序物种由测序结果翻译而成的蛋白质序列数据。

**本项目使用数据库为：Uniprot_MusMusculus_84433_20180123**

**（下载时间：2018-01-23，下载链接：<http://www.uniprot.org>）。**

- 1. **蛋白质定性和定量分析参数**

MASCOT是蛋白质组学定性分析中的金标准，本项目采用MASCOT 2.6进行数据库搜索，详细参数见表3-1。

表3-1 MASCOT鉴定参数

| Item | Value |
| --- | --- |
| - Protein Database | - Uniprot_MusMusculus_84433_20180123 |
| - Enzyme | - Trypsin |
| - Max Missed Cleavages | - 2 |
| - Instrument | - ESI-TRAP |
| - Precursor Mass Tolerance | - ± 20 ppm |
| - Fragment Mass Tolerance | - 0.1Da |
| - Use Average Precursor Mass | - False |
| - Modification Groups   From Quan Method | - iTRAQ 8plex |
| - Dynamic modifications | - Oxidation(M), Acetyl(Protein N-term) - Deamidated(NQ) |
| - Static modifications | - Carbamidomethyl(C) |
| - Database pattern | - decoy |
| - Peptide FDR | - ≤0.01 |

在定量分析过程中，本项目采用Proteome Discoverer 2.1软件对肽段报告离子峰强度值进行抽提，并对定量值进行归一化处理^[2]^，详细参数见表3-2。

表3-2 Proteome Discoverer 2.1定量分析参数

| Item | Value |
| --- | --- |
| Peak integration | - Integration Window Tolerance: 20 ppm - Integration Method: Most Confident Centroid |
| Scan Event Filters | - Mass Analyzer: FTMS - MS Order: MS2 - Activation Type: HCD |
| Quantification-General | - Peptides to Use : Unique |
| Normalization Scaling | - Normalization Mode: Total Peptide Amount - Scaling Mode: On Channels Average(Per File) |

1. **质谱鉴定和定量结果评估**
   1. **肽段离子得分分布**


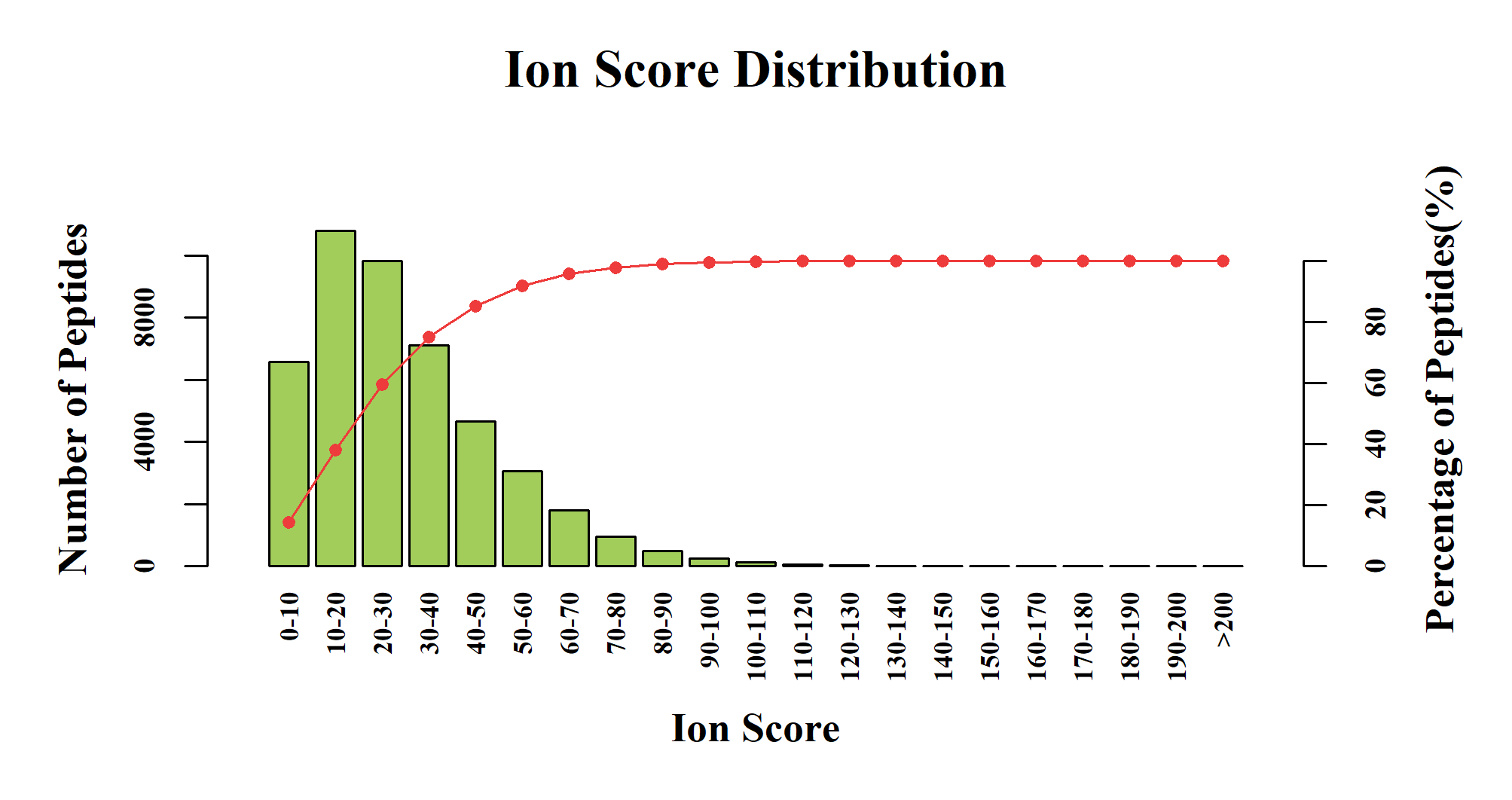


图4-1 肽段离子得分分布图

**备注：**横坐标为MASCOT肽段得分；主纵坐标Number of Peptides对应图中的柱状图，表示鉴定到的具有对应离子得分的肽段数量；次纵坐标对应图中的累积曲线，表示不高于对应离子得分的肽段累积百分比。

本实验中质谱数据采集自Q Exactive Plus高分辨率质谱仪，能够获得高质量的MS1和MS2图谱。然后使用MASCOT这种严格的分析工具对MS图谱数据进行分析，最终获得每张MS2图谱的得分。每一套iTRAQ数据在定性分析工作中均使用FDR<0.01作为筛选标准。

输出文件：

1. Evaluation文件夹\\图4-1 肽段离子得分分布图
   1. **蛋白质相对分子质量分布**

**
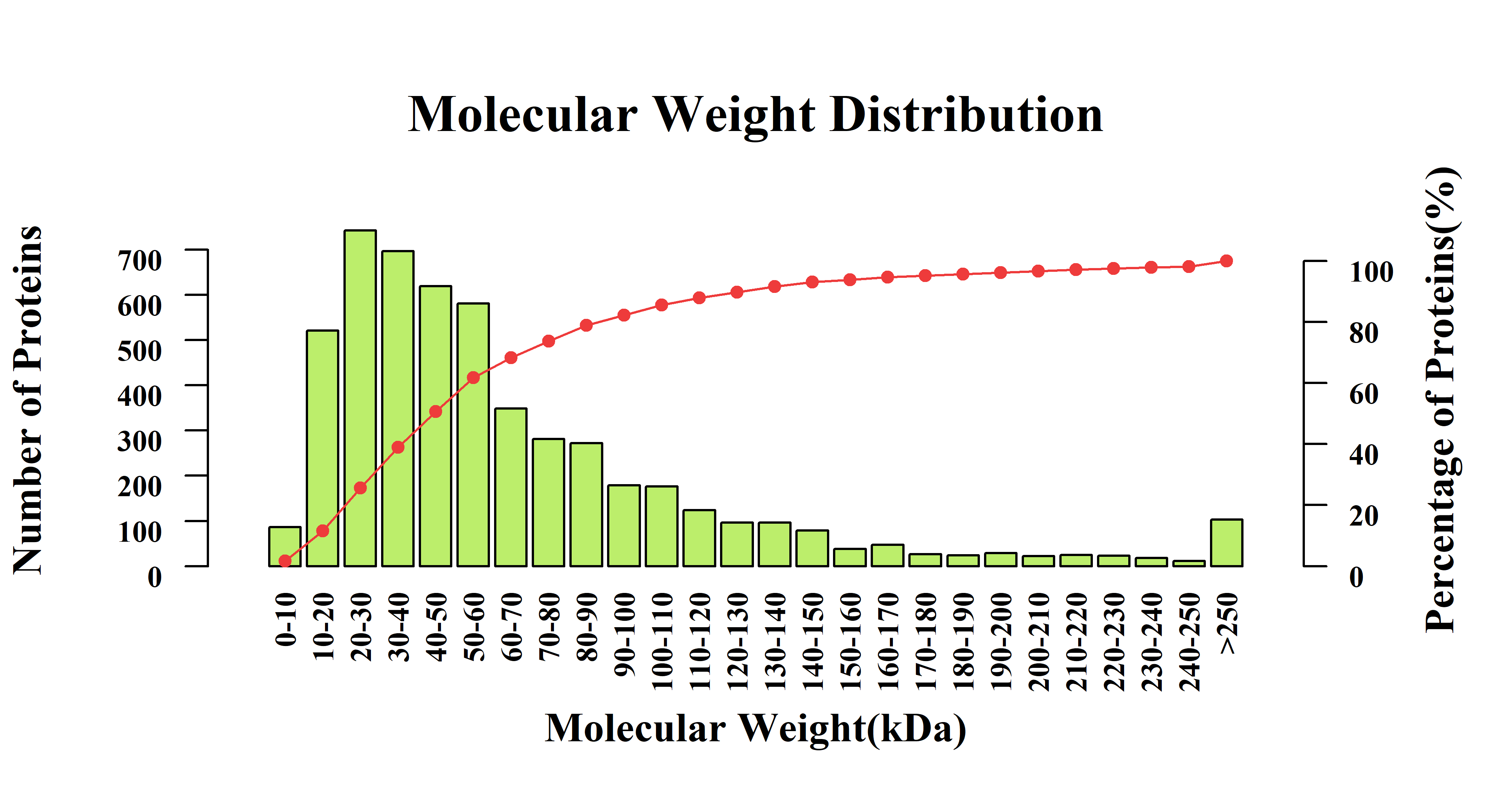
**

图4-2 鉴定蛋白质相对分子质量分布图

**备注：**横坐标为鉴定到的蛋白质的相对分子质量；主纵坐标Number of Proteins对应图中的柱状图，表示鉴定到的具有对应相对分子质量的蛋白质数量；次纵坐标对应图中的累积曲线，表示具有不高于对应相对分子质量的蛋白质的累积百分比。

输出文件：

1. Evaluation文件夹\\图4-2 鉴定蛋白质相对分子质量分布图
   1. **蛋白质等电点分布**


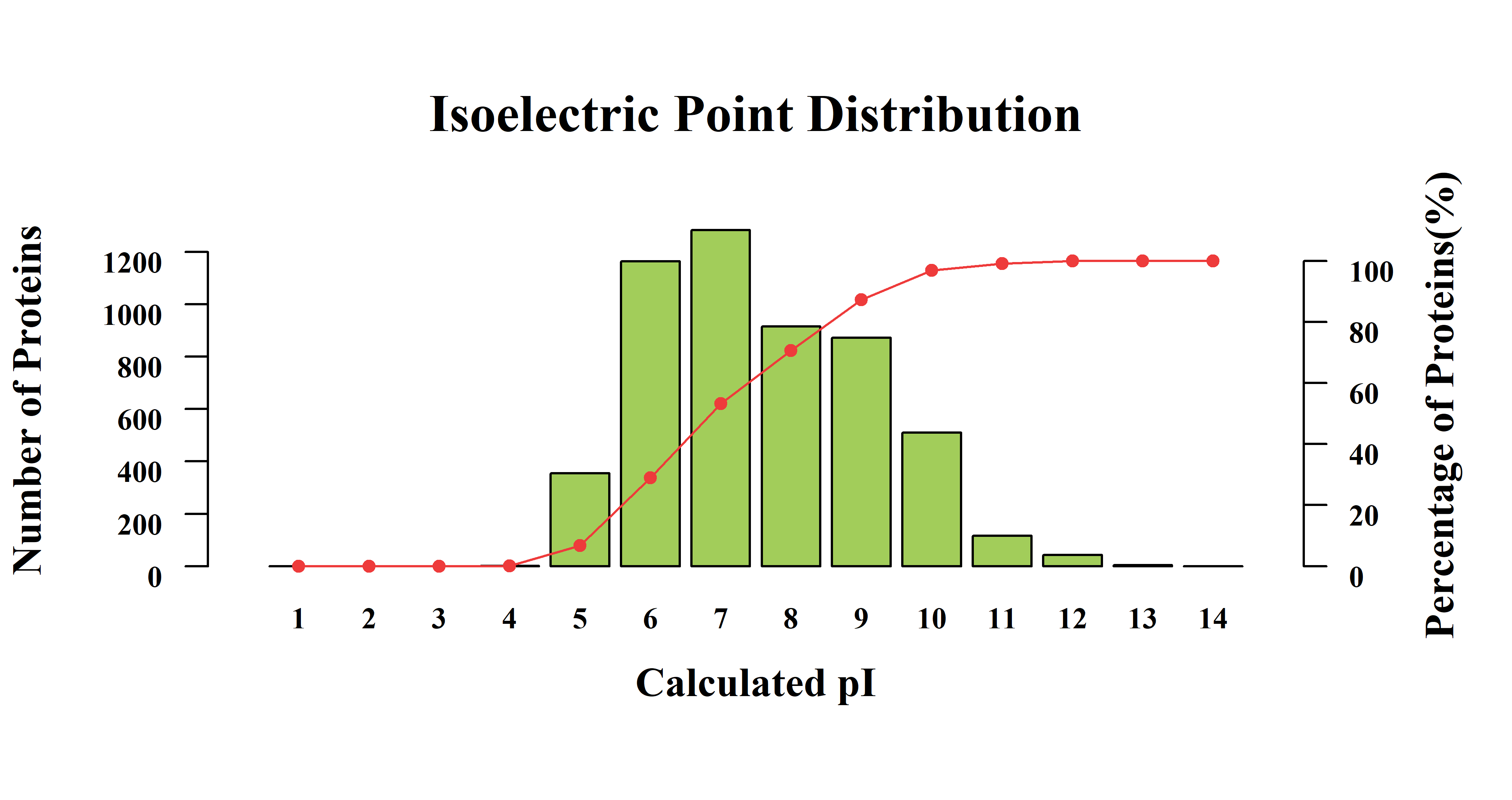


图4-3 鉴定蛋白质等电点分布图

**备注：**横坐标为鉴定到的蛋白质的等电点；主纵坐标Number of Proteins对应图中的柱状图，表示鉴定到的具有对应等电点的蛋白质数量；次纵坐标对应图中的累积曲线，表示等电点不高于对应数值的蛋白质的累积百分比。

输出文件：

1. Evaluation文件夹[\\图4-3 鉴定蛋白质等电点分布](file:///\\\\Figure_4-3_pI_Distribution)
   1. **肽段序列长度分布**

**
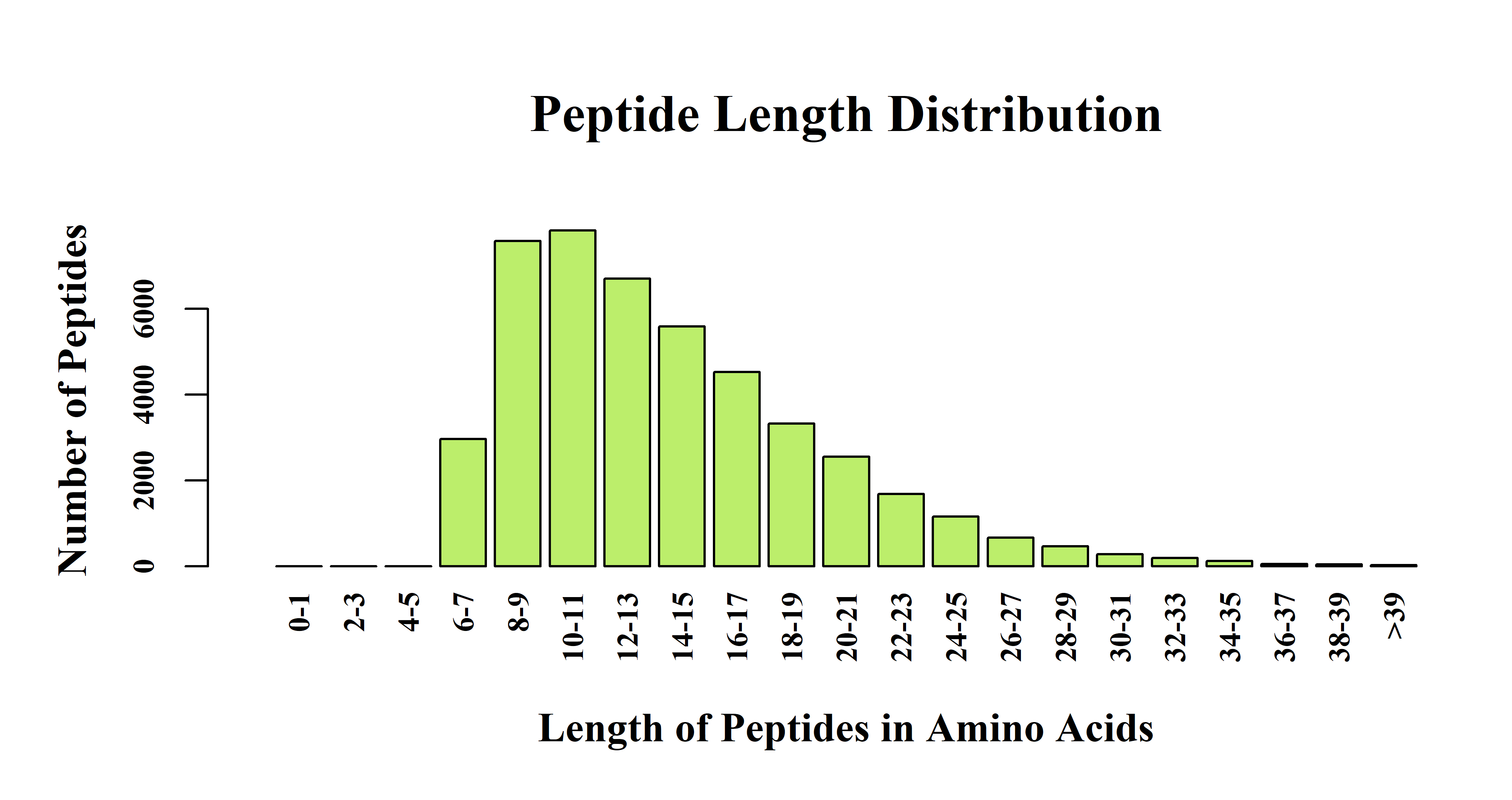
**

图4-4 肽段序列长度分布图

**备注：**横坐标为鉴定到的肽段序列的氨基酸个数；纵坐标为鉴定到的肽段数量。

输出文件：

1. Evaluation文件夹\\图4-4 肽段序列长度分布
   1. **蛋白序列覆盖度分布**


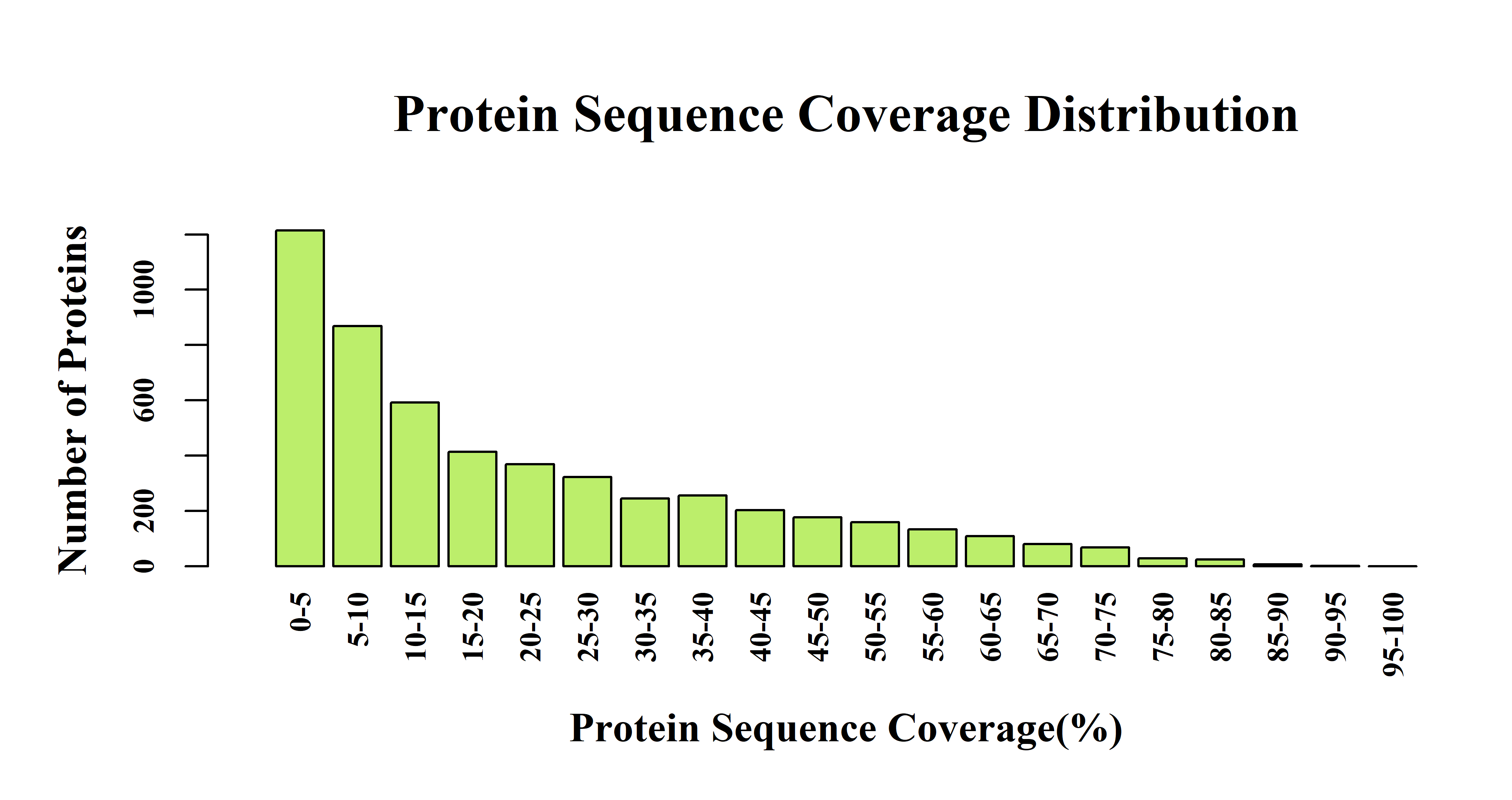


图4-5 蛋白质序列覆盖度分布图

**备注：**该图显示鉴定到的不同覆盖度的蛋白质比例分布情况。横坐标为鉴定到的蛋白质序列覆盖百分比；纵坐标为鉴定到的蛋白质数量。

输出文件：

1. Evaluation文件夹[\\图4-5 蛋白质序列覆盖度分布](file:///\\\\Figure_4-5_Protein_Sequence_Coverage_Distribution)图
   1. **鉴定肽段数量分布**

**
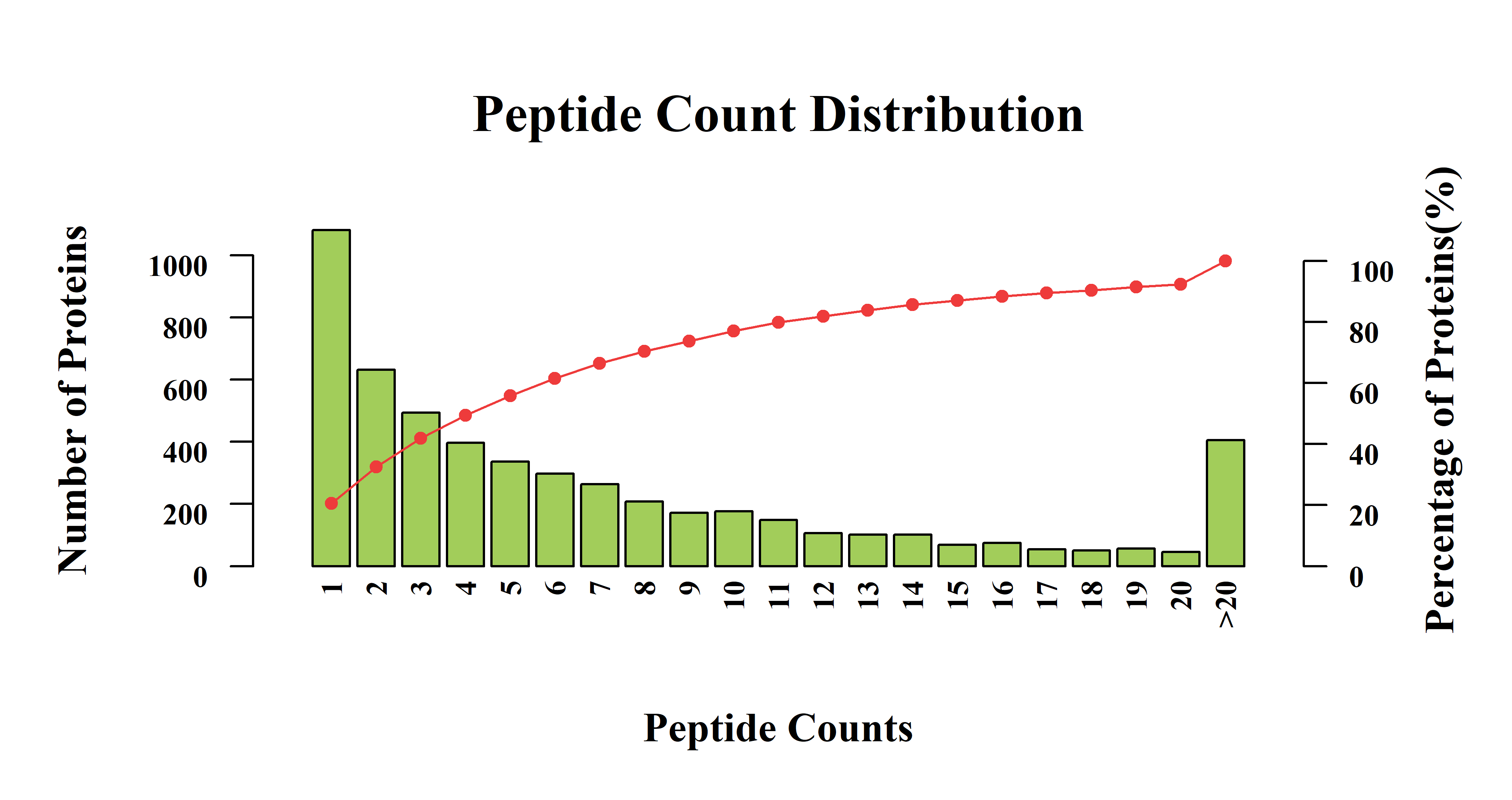
**

图4-6 鉴定肽段数量分布图

**备注：**该图显示鉴定蛋白质所对应的鉴定肽段数量分布情况。横坐标为鉴定蛋白质的肽段数量；主纵坐标Number of Proteins对应图中的柱状图，表示对应鉴定肽段数量的蛋白质数目；次纵坐标对应图中的累积曲线，表示鉴定蛋白质的肽段数量不高于对应数值的蛋白质累积百分比。

输出文件：

1. Evaluation文件夹\\图4-6 鉴定肽段数量分布图
   1. **蛋白质丰度比分布**


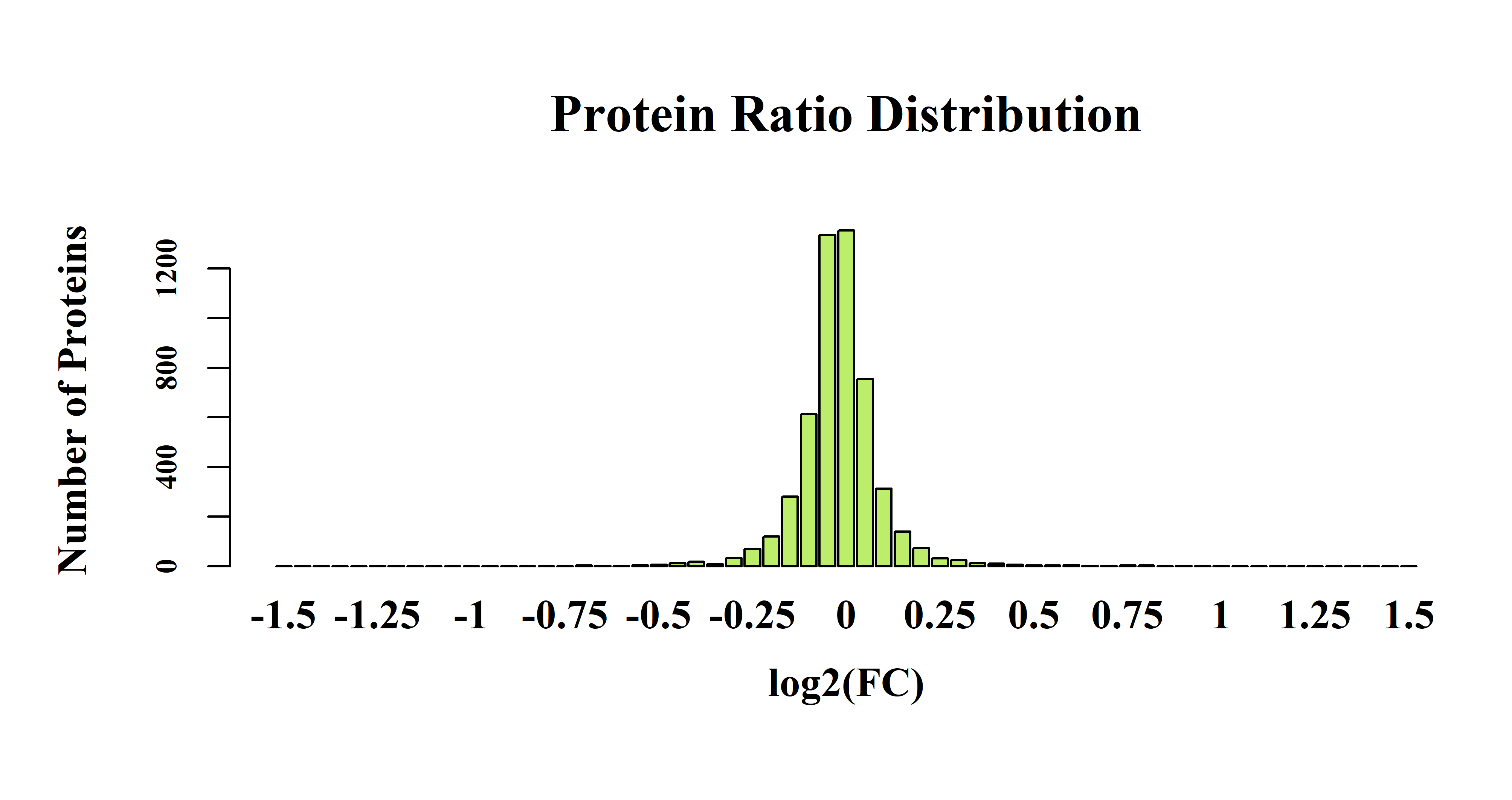


图4-7 蛋白质丰度比分布图

**备注：**横坐标为差异倍数（以2为底的对数变换）；纵坐标为鉴定到的蛋白质数量。该图显示两组样品定量结果中大部分蛋白质的丰度比值接近1。

输出文件：

1. Evaluation文件夹\\图4-7蛋白质丰度比分布图
   1. **火山图（Volcano plot）**


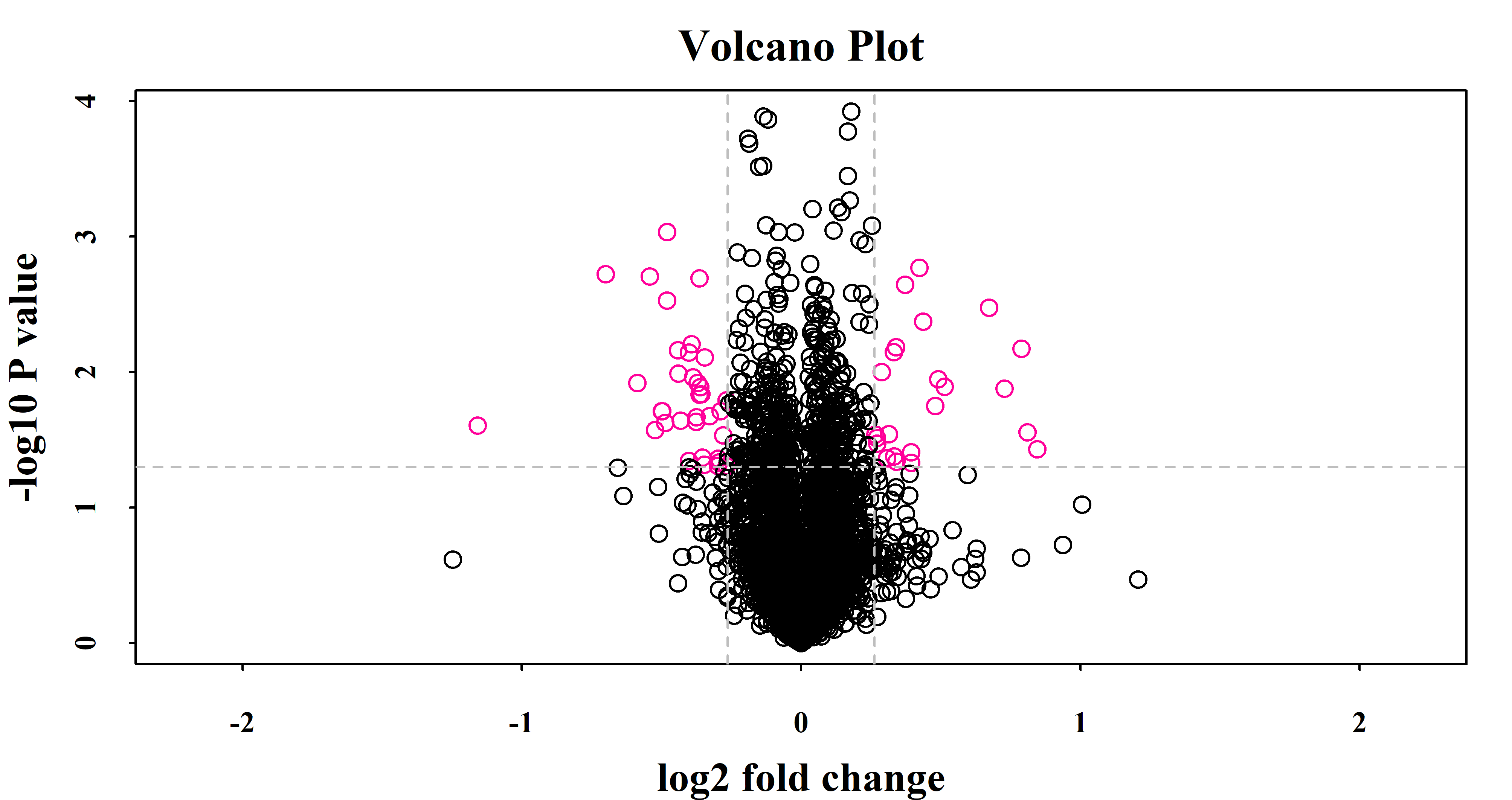


图4-8 火山图

**备注：**采用两组样本间的蛋白质表达差异倍数（Fold change）和P value值两个因素共同绘制火山图，用于显示两组样本数据的显著性差异。横坐标为差异倍数（以2为底的对数变换）；纵坐标为P value值（以10为底的对数变换）。

输出文件：

1. Evaluation文件夹\\图4-8 火山图
2. **生物信息分析内容**

在蛋白质组学中，通过凝胶电泳、质谱等技术产生的海量数据代表了生物体内发生的全部过程及其变化。从这些庞大而复杂的实验数据中寻找生物体的改变以及引起这些改变的源头和机制，是蛋白质组生物信息学的主要任务。

在定量蛋白质组学分析中，常用的生物信息学分析方法包括（但不局限于）：

- 显著性差异分析
- GO注释及富集分析
- KEGG通路注释及富集分析
- 聚类分析
- 蛋白质相互作用网络分析
  1. **Gene Ontology (GO) 功能注释**

基因本体（Gene Ontology）是一个标准化的功能分类体系，提供了一套动态更新的标准化词汇表，并以此从三个方面描述生物体中基因和基因产物的属性：参与的生物过程（Biological Process），分子功能（Molecular Function）和细胞组分（Cellular Component）^[3]^。

通常情况下，对目标蛋白质集合的GO功能注释的步骤和流程^[4,5]^大致可以归纳如下：


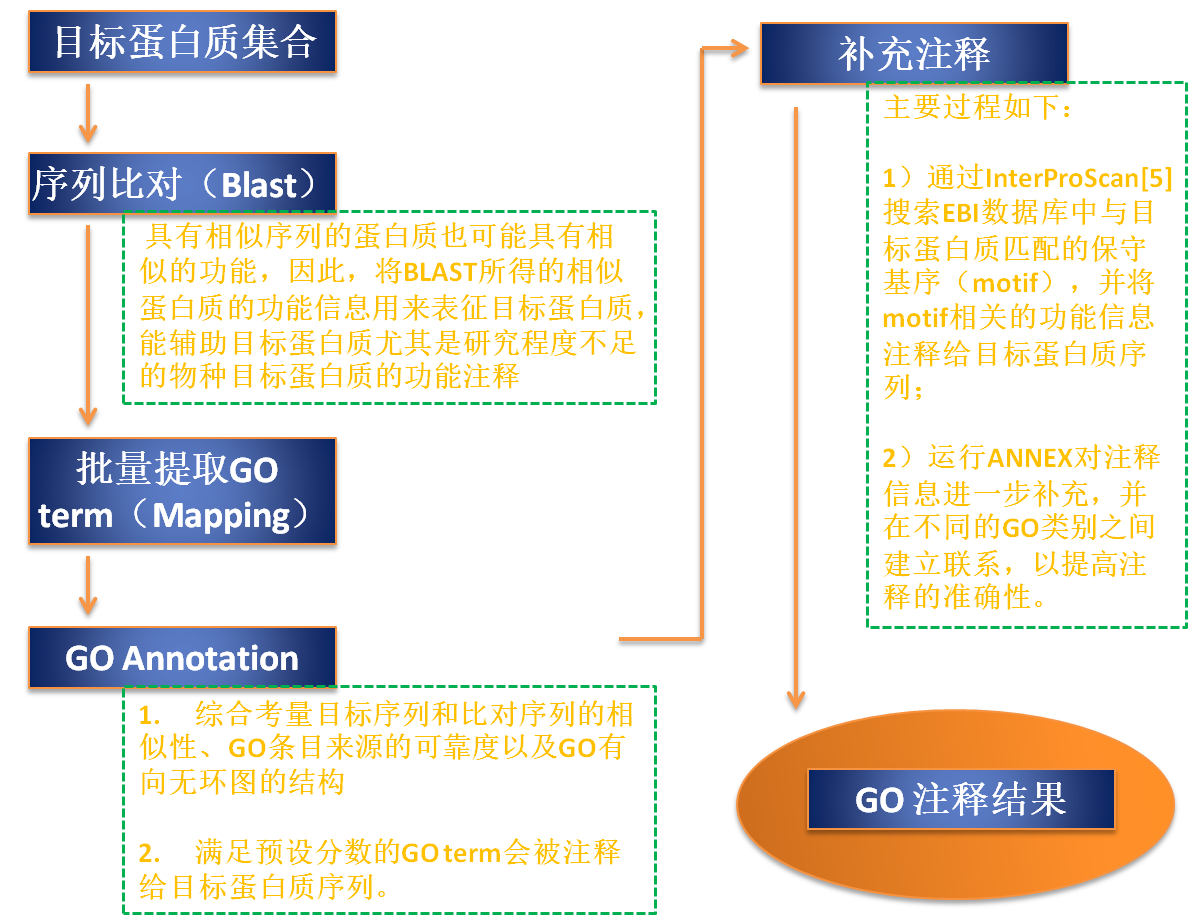


图5-1-1 GO功能注释流程图


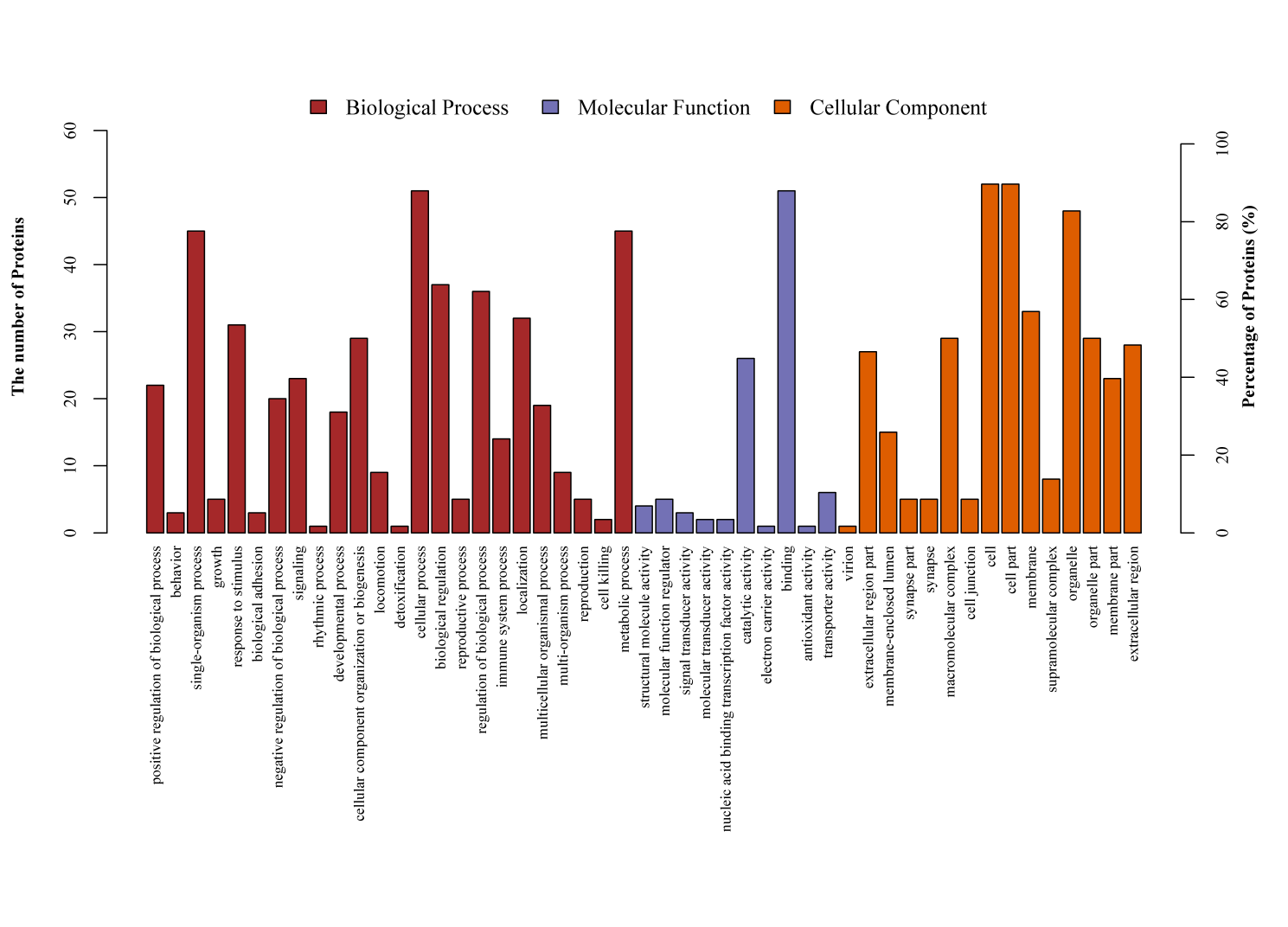


图5-1-2 GO注释结果的level 2统计（A VS B）

输出文件：

1. GO分析结果文件夹\\GO功能注释统计表
   1. **差异表达蛋白质GO富集分析**

对目标蛋白质集合的GO注释可以从参与的生物学过程、具有的分子功能和所处的细胞组分三个方面对这些蛋白质进行分类。各个分类比例虽然可以在一定程度上反映实验设计中生物学处理对各个分类的影响程度大小，但是单纯依据该比例来评价各个分类受影响的显著程度是不准确的，还需要同时考虑各个分类在总体蛋白质集合（例如：实验中全部定性的蛋白质，该物种所有已知的蛋白质等）中的分布情况。

通常情况下，GO注释的显著性富集分析是通过Fisher精确检验（Fisher’s Exact Test）来评价某个GO term蛋白质富集度的显著性水平（图5-2）。


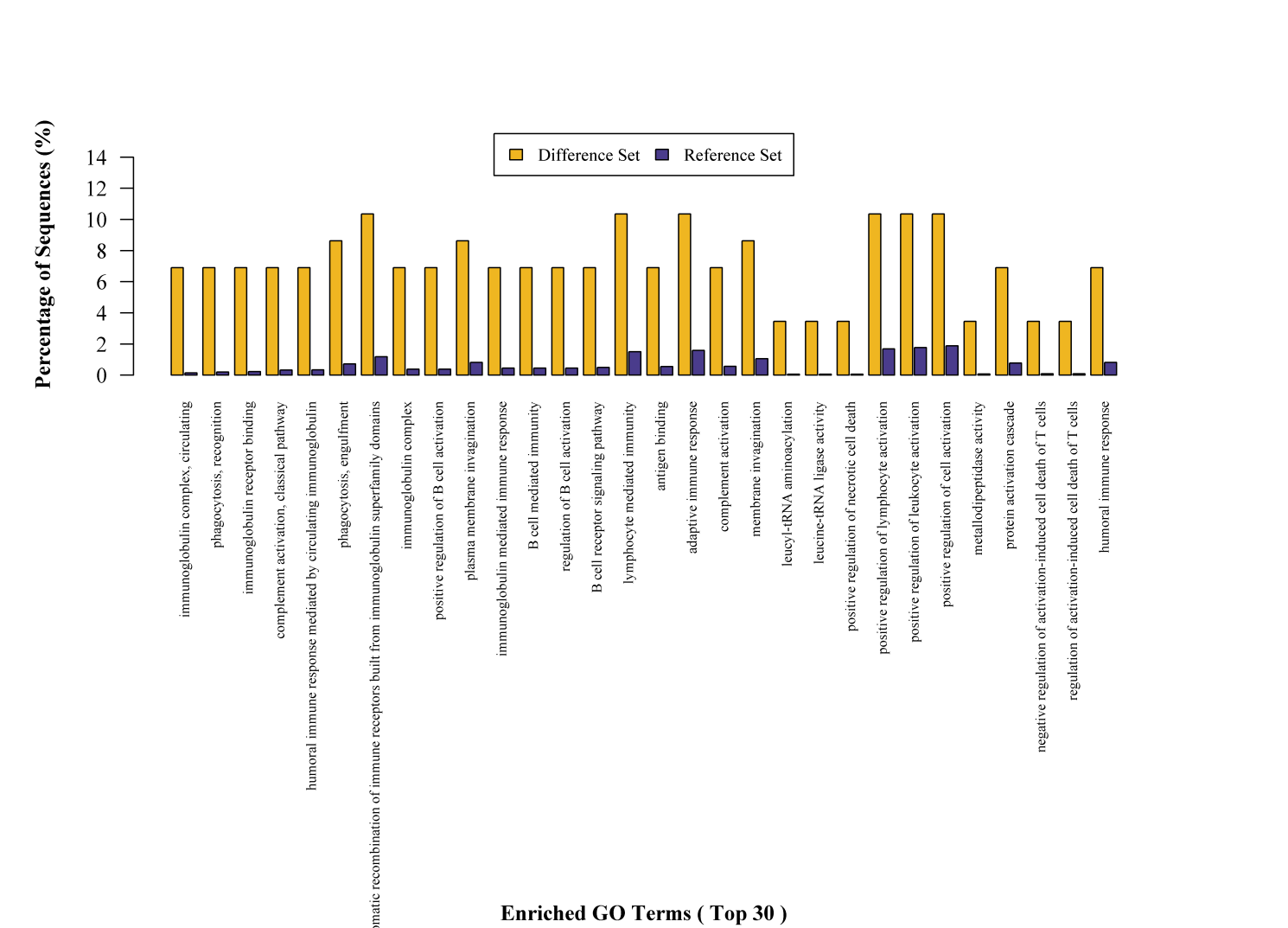


图5-2 显著富集的GO term统计（A VS B）

输出文件：

1. GO分析结果文件夹\\GO功能注释统计表
   1. **KEGG通路注释**

在生物体中，蛋白质并不独立行使其功能，而是不同蛋白质相互协调完成一系列生化反应以行使其生物学功能。因此，通路分析是更系统、全面地了解细胞的生物学过程、性状或疾病的发生机理、药物作用机制等最直接和必要的途径。

KEGG^[6]^是常用于通路研究的数据库之一。通常情况下，对目标蛋白质集合进行基于KEGG数据库通路注释的步骤和流程大致可以归纳如下：

**目标蛋白**

**Homologs**

**Blast to KEGG GENES**

**Cut off by Bi-Directional**

**Hit**

**Ortholog Candidates**

**Grouping by KO**

**KO groups**

**Ranking of KO**

**Pathway Mapping**

**Scoring by Probability and Heuristics**

图5-3-1 KEGG通路注释流程图


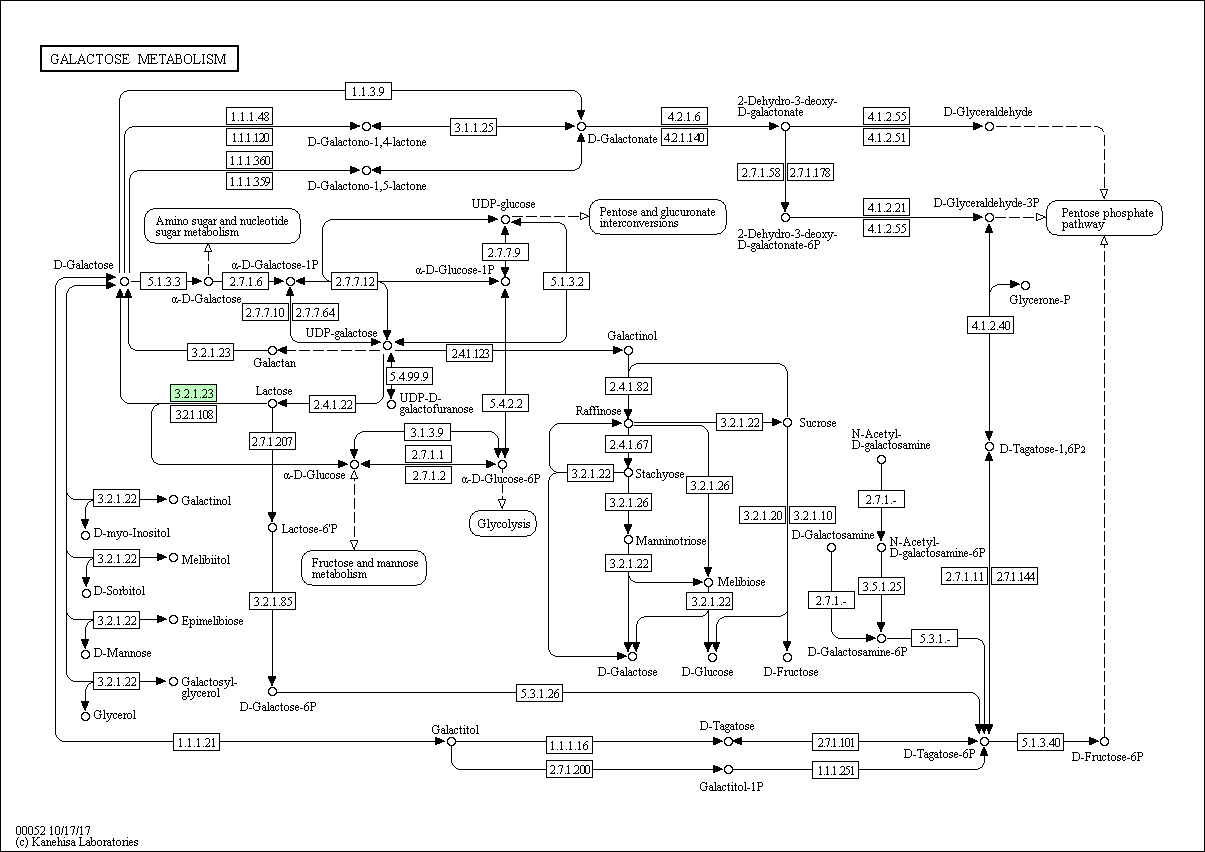


图5-3-2 KEGG信号通路注释（A VS B）

输出文件：

1. KEGG分析结果文件夹\\KEGG通路注释统计表

KEGG分析结果文件夹\\map文件夹

- 1. **差异表达蛋白质KEGG通路富集分析**

KEGG通路富集分析方法与GO富集分析相似，即以KEGG通路为单位，以所有定性蛋白质为背景，通过Fisher精确检验（Fisher’s Exact Test），来分析计算各个通路蛋白质富集度的显著性水平，从而确定受到显著影响的代谢和信号转导途径（图5-4）。


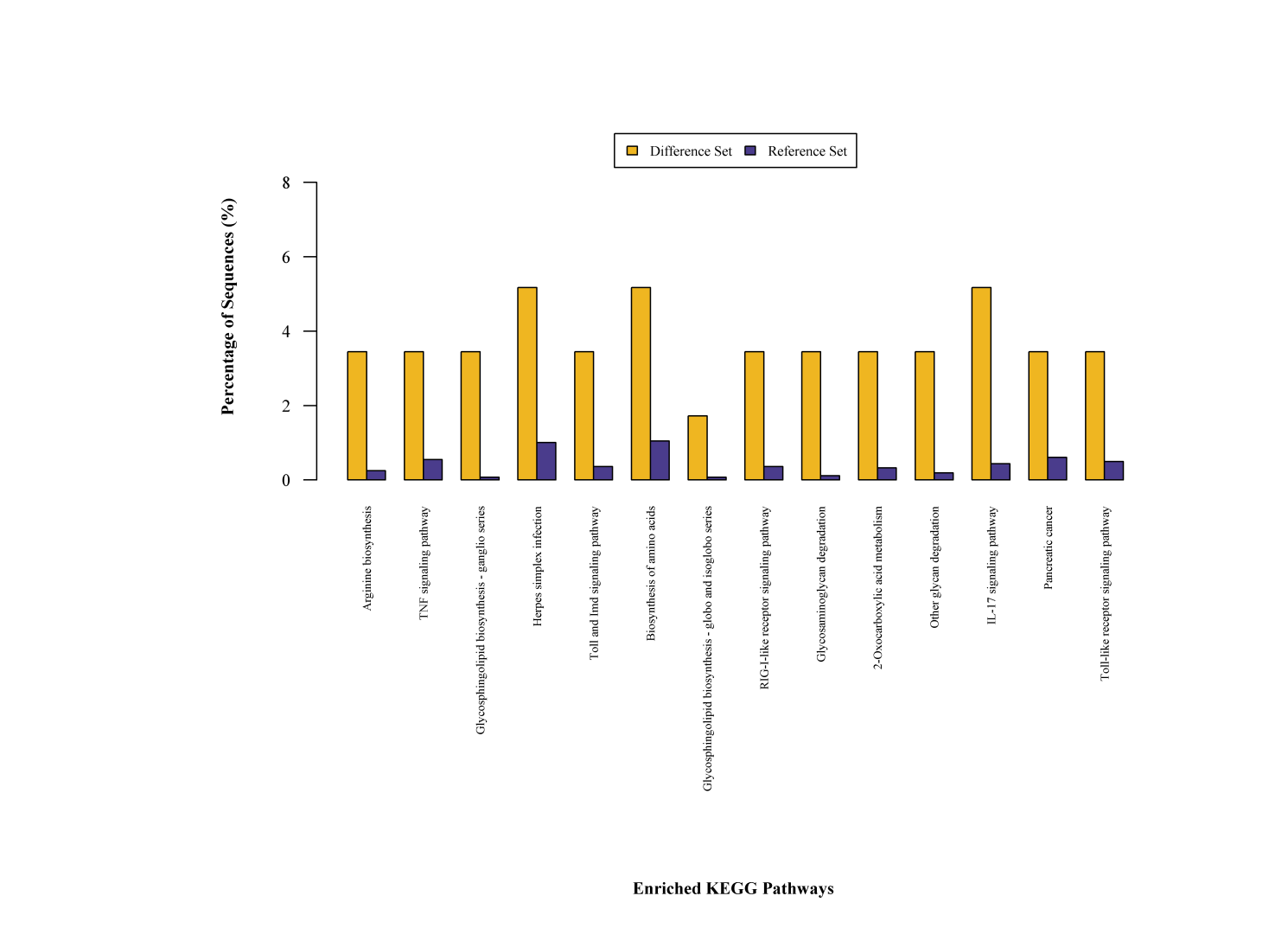


图5-4 显著富集的KEGG通路统计（A VS B）

输出文件：

1. KEGG分析结果文件夹\\KEGG通路注释统计表

KEGG分析结果文件夹\\map文件夹

- 1. **蛋白质聚类分析（Clustering）**

聚类分析是一种常用的探索性数据分析方法，其目的是在相似性的基础上对数据进行分组、归类。聚类分组的结果中，组内的数据模式相似性较高，而组间的数据模式相似性较低。

在聚类分析过程中，聚类算法会对样本（Sample）和变量（Variable，在蛋白质组学研究中通常指蛋白质的定量信息）两个维度进行分类。对样本的聚类结果可以检验所筛选的目标蛋白质的合理性，即这些目标蛋白质表达量的变化可否代表生物学处理对样本造成的显著影响；目标蛋白质的聚类结果可以帮助我们从蛋白质集合中区分具有不同表达模式的蛋白质子集合，具有相近表达模式的蛋白质可能具有相似的功能或者参与相同的生物学途径，或者在通路中处于临近的调控位置（图5-5）。


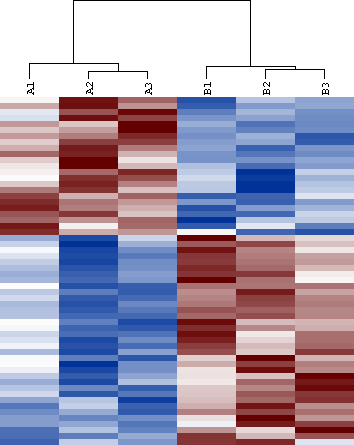

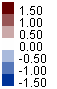


图5-5 聚类分析结果（A VS B）

输出文件：

1. 聚类分析结果文件夹
2. **参考文献**
3. Ross PL, Huang YN, et al. Multiplexed protein quantitation in Saccharomyces cerevisiae using amine-reactive isobaric tagging reagents. Mol Cell Proteomics. 2004; 3(12): 1154-69.
4. Proteome Discoverer Version 2.1. Thermo Fisher Scientific Inc. 2014.
5. Ashburner M, Ball CA, et al. Gene ontology: tool for the unification of biology. The Gene Ontology Consortium. Nat Genet. 2000; 25(1): 25-9.
6. Götz S, García-Gómez JM, et al. High-throughput functional annotation and data mining with the Blast2GO suite. Nucleic Acids Res. 2008; 36(10): 3420-35.
7. Quevillon E, Silventoinen V, et al. InterProScan: protein domains identifier. Nucleic Acids Res. 2005; 33(Web Server issue): W116-20.
8. Kanehisa M, Goto S, et al. KEGG for integration and interpretation of large-scale molecular data sets. Nucleic Acids Res. 2012; 40(Database issue): D109-14.
9. **输出文件及保存位置**

| **输出文件** | **保存位置** |
| --- | --- |
| - 蛋白质鉴定列表 | 质谱鉴定和定量结果文件夹\\蛋白质鉴定列表 |
| - 肽段鉴定列表 | 质谱鉴定和定量结果文件夹\\肽段鉴定列表 |
| - 蛋白质定量和差异分析列表 | 质谱鉴定和定量结果文件夹\\蛋白质定量和差异分析列表 |
| - 肽段离子得分分布图 | Evaluation文件夹\\肽段离子得分分布图 |
| - 鉴定蛋白质相对分子质量分布图 | Evaluation文件夹\\鉴定蛋白质相对分子质量分布图 |
| - 鉴定蛋白质等电点分布图 | Evaluation文件夹\\鉴定蛋白质等电点分布图 |
| - 肽段序列长度分布图 | Evaluation文件夹\\肽段序列长度分布图 |
| - 蛋白质序列覆盖度分布图 | Evaluation文件夹\\蛋白质序列覆盖度分布图 |
| - 鉴定肽段数量分布图 | Evaluation文件夹\\鉴定肽段数量分布图 |
| - 蛋白质丰度比分布图 | Evaluation文件夹\\蛋白质丰度比分布图 |
| - 火山图 | Evaluation文件夹\\火山图 |
| - GO功能注释结果 | GO分析结果文件夹\\GO功能注释统计表 |
| - 差异表达蛋白质GO富集分析结果 | GO分析结果文件夹\\GO功能注释统计表 |
| - KEGG通路注释结果 | KEGG分析结果文件夹\\KEGG通路注释统计表  KEGG分析结果文件夹\\map文件夹 |
| - 差异表达蛋白质KEGG通路富集结果 | KEGG分析结果文件夹\\KEGG通路注释统计表  KEGG分析结果文件夹\\map文件夹 |
| - 蛋白质聚类分析结果 | 聚类分析结果文件夹 |
