# Supplementary material for: A novel co-target of ACY1 governing plasma membrane translocation of SphK1 contributes to inflammatory and neuropathic pain
Source: iScience. 2023 May 28;26(6):106989. doi: 10.1016/j.isci.2023.106989 (PMC10291574; doi:10.1016/j.isci.2023.106989)
Supplement: Data S1. Data file of exported proteomics datasets, related to Figure 1 [file mmc2.zip › Date S1/1-M-GSGC0160906正式实验报告/预实验报告/iTRAQ预实验结果说明.docx]

**Q exactive系列质谱仪为高精度仪器，一般满足筛选条件unique peptide≥1的蛋白质即为可信蛋白质，如果采用更严格的筛选条件可设置为unique peptide≥2。**

**附件1. 蛋白质鉴定列表**

| 表头 | 定义 | 描述 |
| --- | --- | --- |
| Accession | 蛋白质登录号 | 蛋白质序列数据库（FASTA database）中的蛋白质编号 |
| Gene Name | 基因名 | 显示Fasta header列中注释的基因名称，如果数据库中的注释信息不完整或没有基因名称，则不显示该内容。 |
| Description | 蛋白质信息描述 | 基于蛋白质序列的数据库中的蛋白质功能描述。 |
| Coverage | 肽段覆盖率 | 鉴定到的氨基酸数目占蛋白质总氨基酸数目的比例。The percentage of the protein sequence covered by identified peptides |
| Peptides | 肽段数 | The number of distinct peptide sequences in the protein group （该蛋白质（组）的所有鉴定肽段数目） |
| PSMs | 匹配到肽段的质谱图谱总数 | 全称是peptide spectrum matches，为该蛋白质组的所有肽段匹配到全部质谱图的数量。The total number of identified peptide sequences for the protein, including those redundantly identified. |
| Unique Peptides | 唯一肽段数 | The number of peptide sequences unique to a protein group （该蛋白质（组）的特有肽段数目） |
| AAs | 氨基酸个数 | 蛋白质的氨基酸总数 |
| MW [kDa] | 分子量 | 蛋白质的理论分子量。该分子量是软件根据数据库中的蛋白质序列计算得到的。如果用以参加计算的蛋白质序列不是完整的全长序列，比如由转录组翻译而来的蛋白质序列，由此计算得到的分子量会小于完整蛋白质的分子量。 |
| calc. pI | 等电点 | 蛋白质的理论等电点 |

**附件2. 肽段鉴定列表**

| **表头** | **定义** | **描述** |
| --- | --- | --- |
| Sequence | 肽段氨基酸序列 | 描述肽段氨基酸的组成 |
| Modifications | 修饰 | 描述修饰氨基酸、位置及修饰方式。如： C(Carbamidomethyl)：半胱氨酸修饰；M(Oxidation)：甲硫氨酸氧化；S/T/Y（Phosphorylation）：丝氨酸/苏氨酸/酪氨酸磷酸化 |
| Qvality PEP | 后验错误概率 | The posterior error probability (PEP) is the probability that the observed PSM is incorrect. 相当于局部的FDR（Local FDR）。This value essentially operates as a p-value, where smaller is better. For example, if the PEP associated with (EAMRPK, *s*) is 5 percent, there is a 95 percent chance that the EAMRPK peptide was in the mass spectrometer when spectrum *s* was generated. The FDR measures the error rate associated with a collection of PSMs, and the PEP measures the probability of error for a single PSM. |
| Qvality q-value | 最小的错误发生率 | A q-value is the minimal false discovery rate at which the identification is considered correct. 相当于全局的FDR （Global FDR），报告结果给出的所有鉴定数据都满足FDR<0.01的筛选标准. q-values are estimated using the distribution of scores from the decoy database search. A q-value of 0.01 for the EAMRQPK peptide matching spectrum, *s*, means that if you try all possible FDR thresholds, 1 percent is the minimal FDR threshold at which the PSM of EAMRQPK to *s* appears in the output list. Although the q-value is associated with a single PSM, it also depends on the data set that the PSM occurs in. |
| PSMs | 匹配到肽段的质谱图谱总数 | 全称是peptide spectrum matches，为该肽段匹配到全部质谱图的数量。The total number of identified peptide sequences for the protein, including those redundantly identified. |
| Master Protein Accessions | 蛋白质登录号 | 蛋白质序列数据库（FASTA database）中的蛋白质编号 |
| Missed Cleavages | 漏切位点数量 | 鉴定肽段序列中含有胰蛋白酶漏切位点（即K和R）的数量 |
| Theo. MH+ [Da] | 肽段分子量 | 带一个电荷（质子化）的肽段理论分子量 |
| Abundances XXX | 肽段丰度 | 对应样品中肽段的相对丰度 |
| Ions Score Mascot | 肽段得分 | MASCOT肽段得分 |
| Charge Mascot | 电荷 | 肽段电荷数 |
| DeltaM [ppm] Mascot | 理论分子量和实验分子量的差值 | 肽段理论分子量和实验测得分子量的差异 |
| RT [min] Mascot | 保留时间 | 全称为retention time，指被分离样品组分从进样开始到柱后出现该组分浓度极大值时的时间，即从进样开始到出现某组分色谱峰的顶点时为止所经历的时间，称为此组分的保留时间，用RT表示，常以分（min）为时间单位。 |
