# Supplementary figures and images for: A novel co-target of ACY1 governing plasma membrane translocation of SphK1 contributes to inflammatory and neuropathic pain (part 2 of 2)
Source: iScience. 2023 May 28;26(6):106989. doi: 10.1016/j.isci.2023.106989 (PMC10291574; doi:10.1016/j.isci.2023.106989)

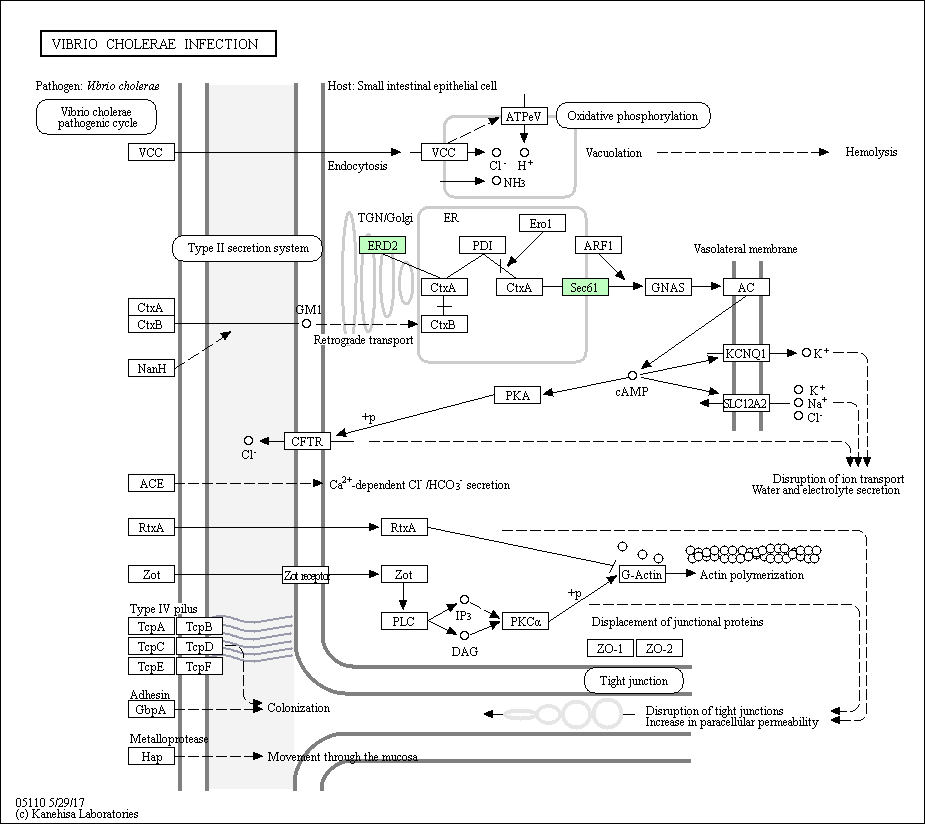

Supplement: Data S1. Data file of exported proteomics datasets, related to Figure 1 [file mmc2.zip › Date S1/1-M-GSGC0160906正式实验报告/KEGG分析结果文件夹/map/map05110.png]

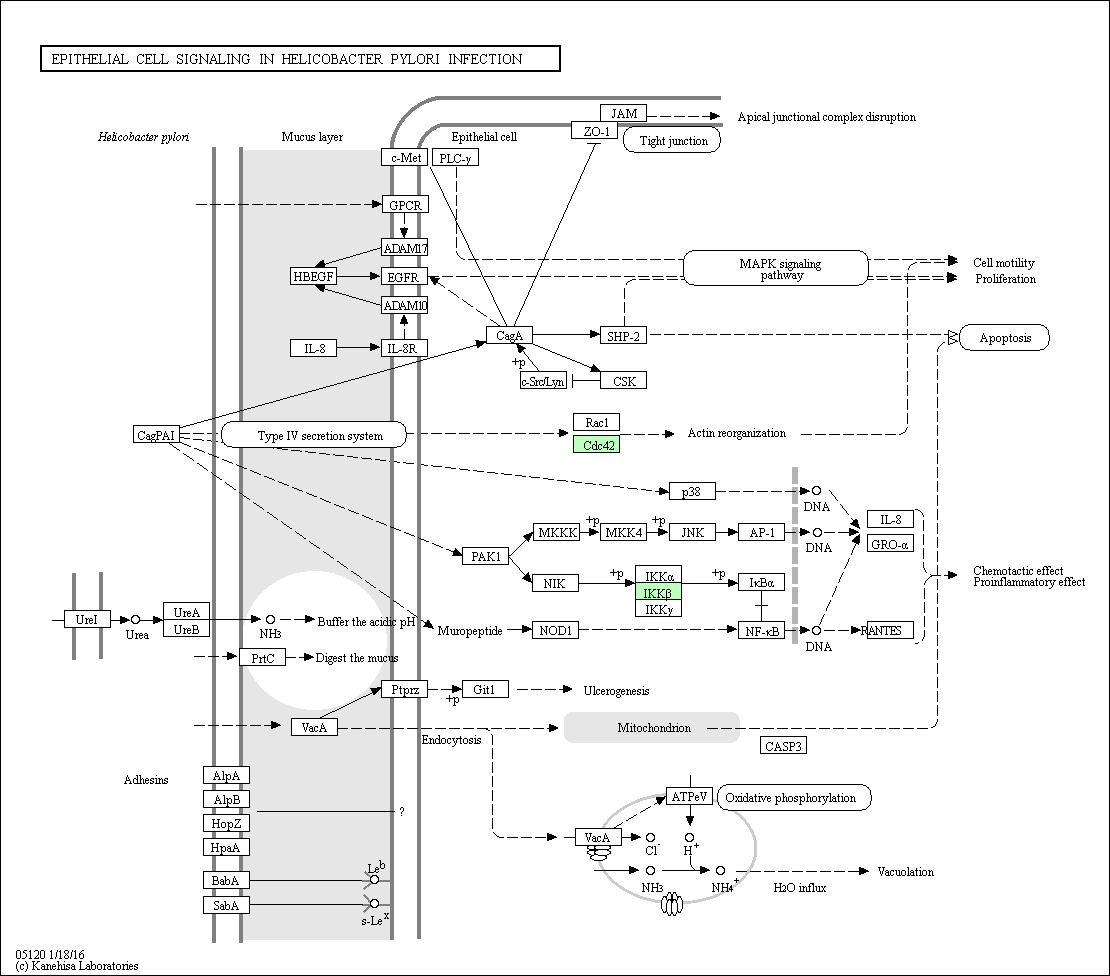

Supplement: Data S1. Data file of exported proteomics datasets, related to Figure 1 [file mmc2.zip › Date S1/1-M-GSGC0160906正式实验报告/KEGG分析结果文件夹/map/map05120.png]

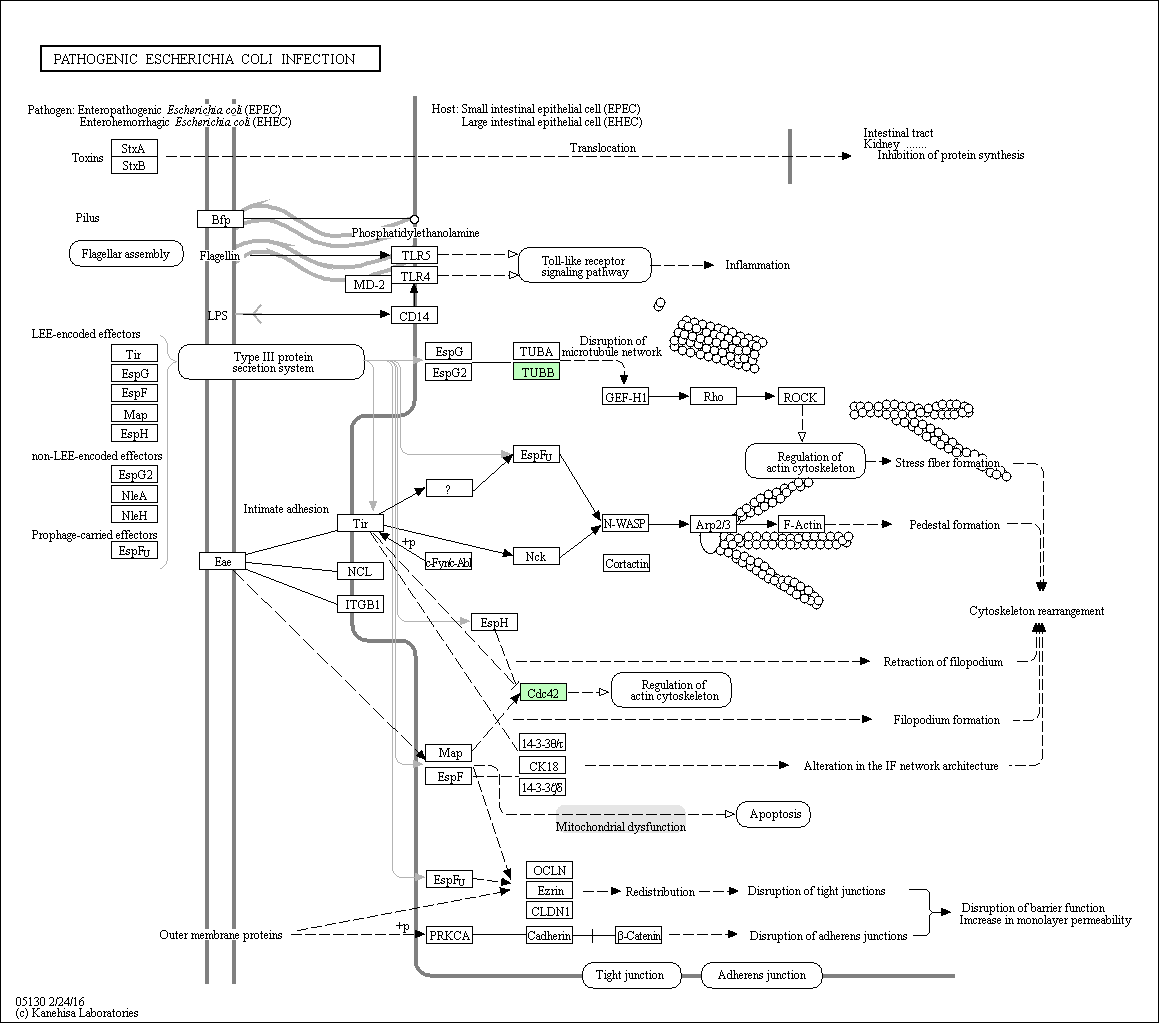

Supplement: Data S1. Data file of exported proteomics datasets, related to Figure 1 [file mmc2.zip › Date S1/1-M-GSGC0160906正式实验报告/KEGG分析结果文件夹/map/map05130.png]

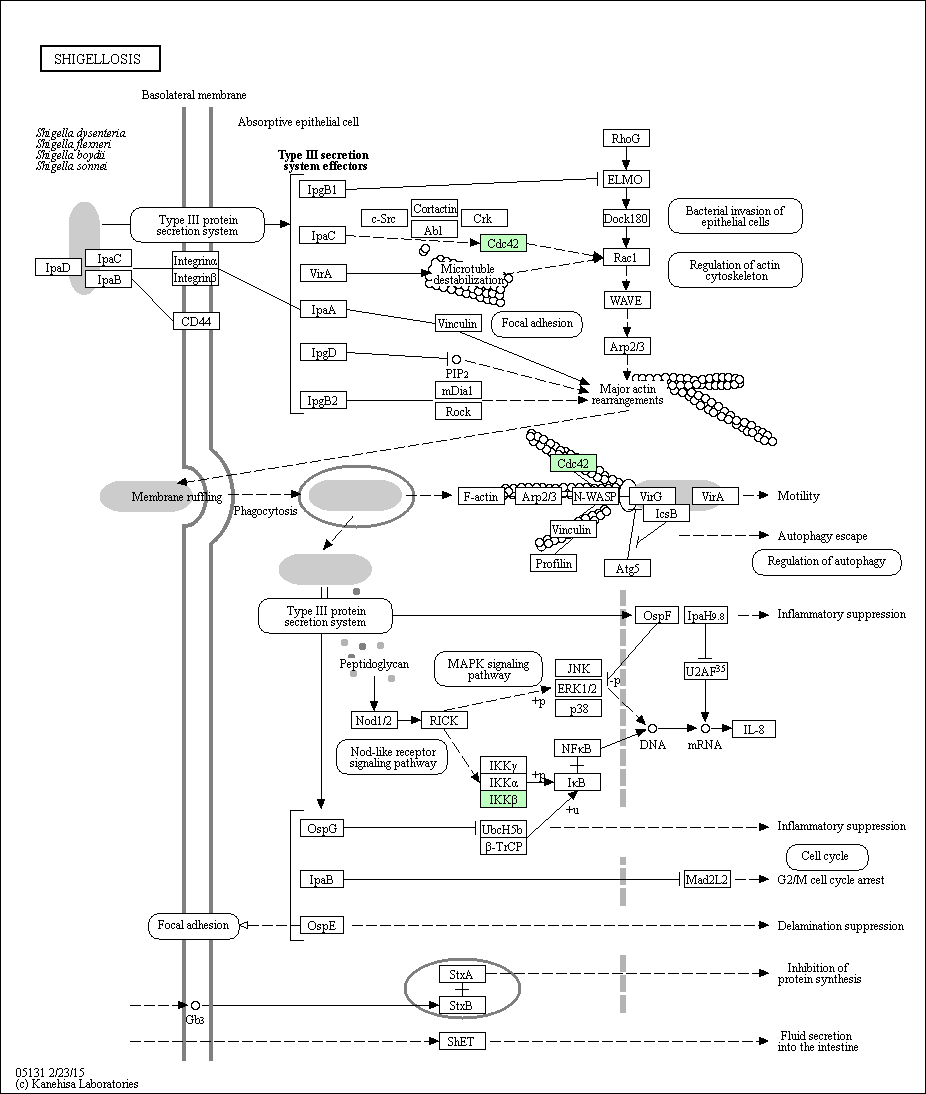

Supplement: Data S1. Data file of exported proteomics datasets, related to Figure 1 [file mmc2.zip › Date S1/1-M-GSGC0160906正式实验报告/KEGG分析结果文件夹/map/map05131.png]

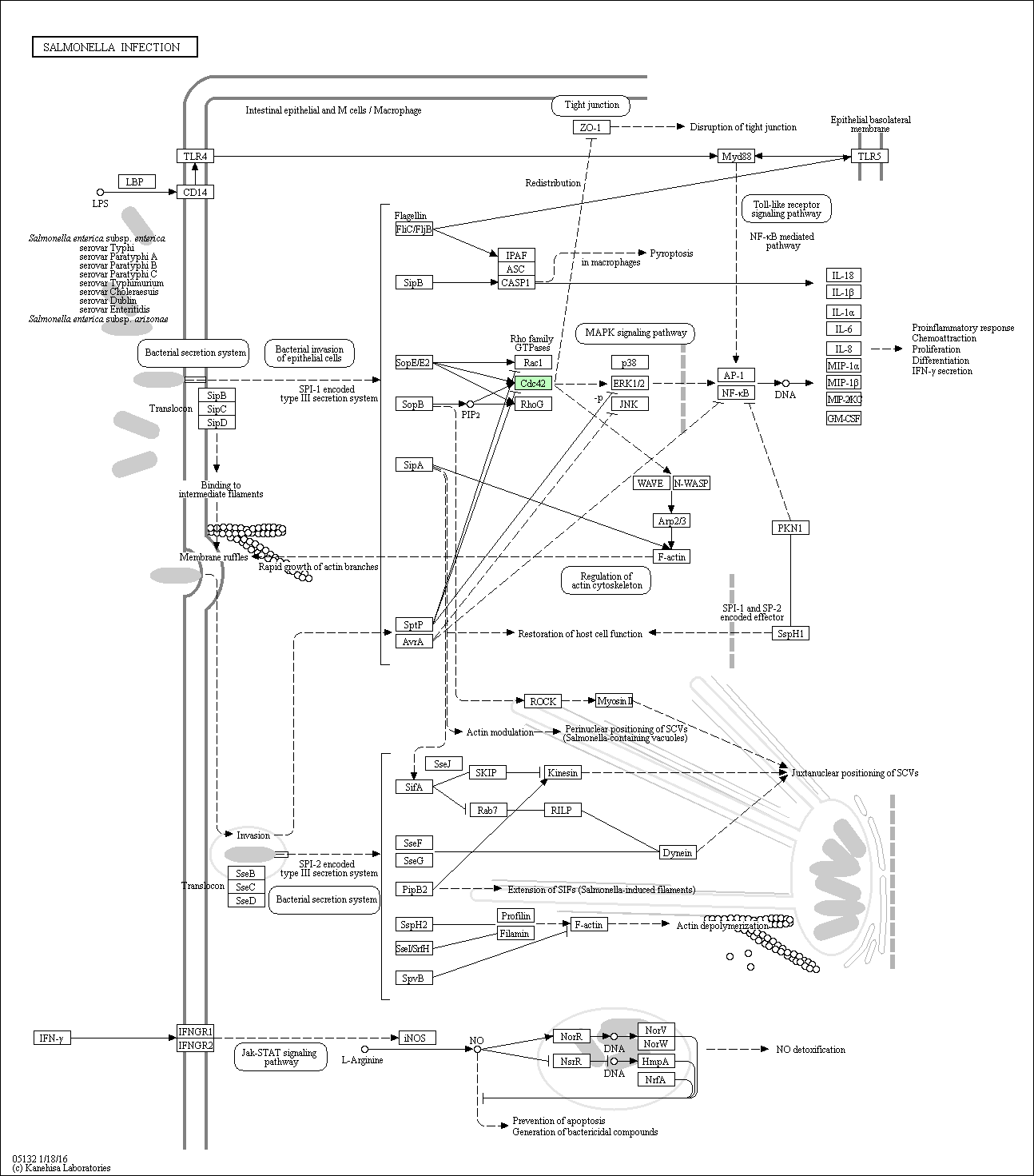

Supplement: Data S1. Data file of exported proteomics datasets, related to Figure 1 [file mmc2.zip › Date S1/1-M-GSGC0160906正式实验报告/KEGG分析结果文件夹/map/map05132.png]

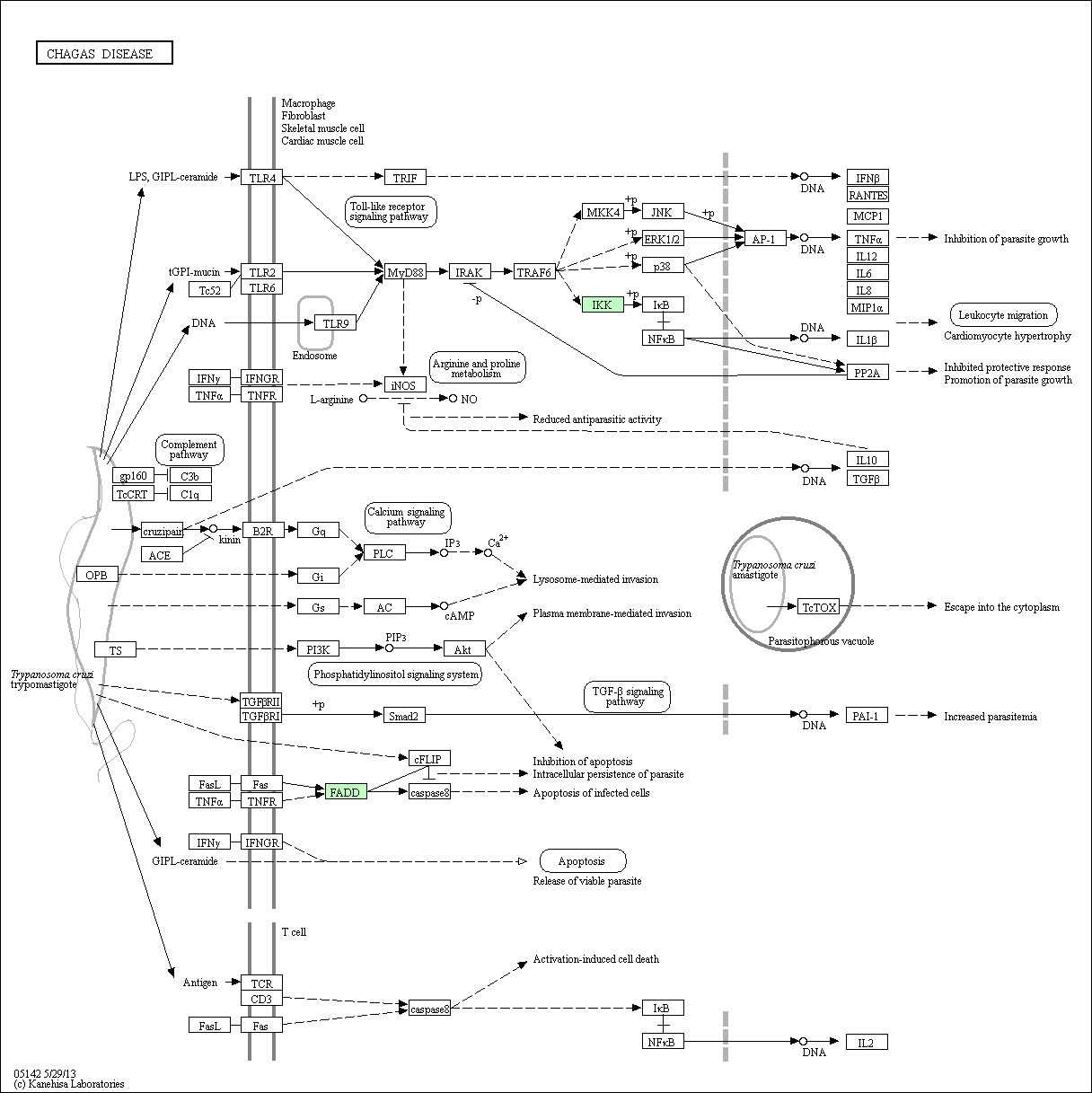

Supplement: Data S1. Data file of exported proteomics datasets, related to Figure 1 [file mmc2.zip › Date S1/1-M-GSGC0160906正式实验报告/KEGG分析结果文件夹/map/map05142.png]

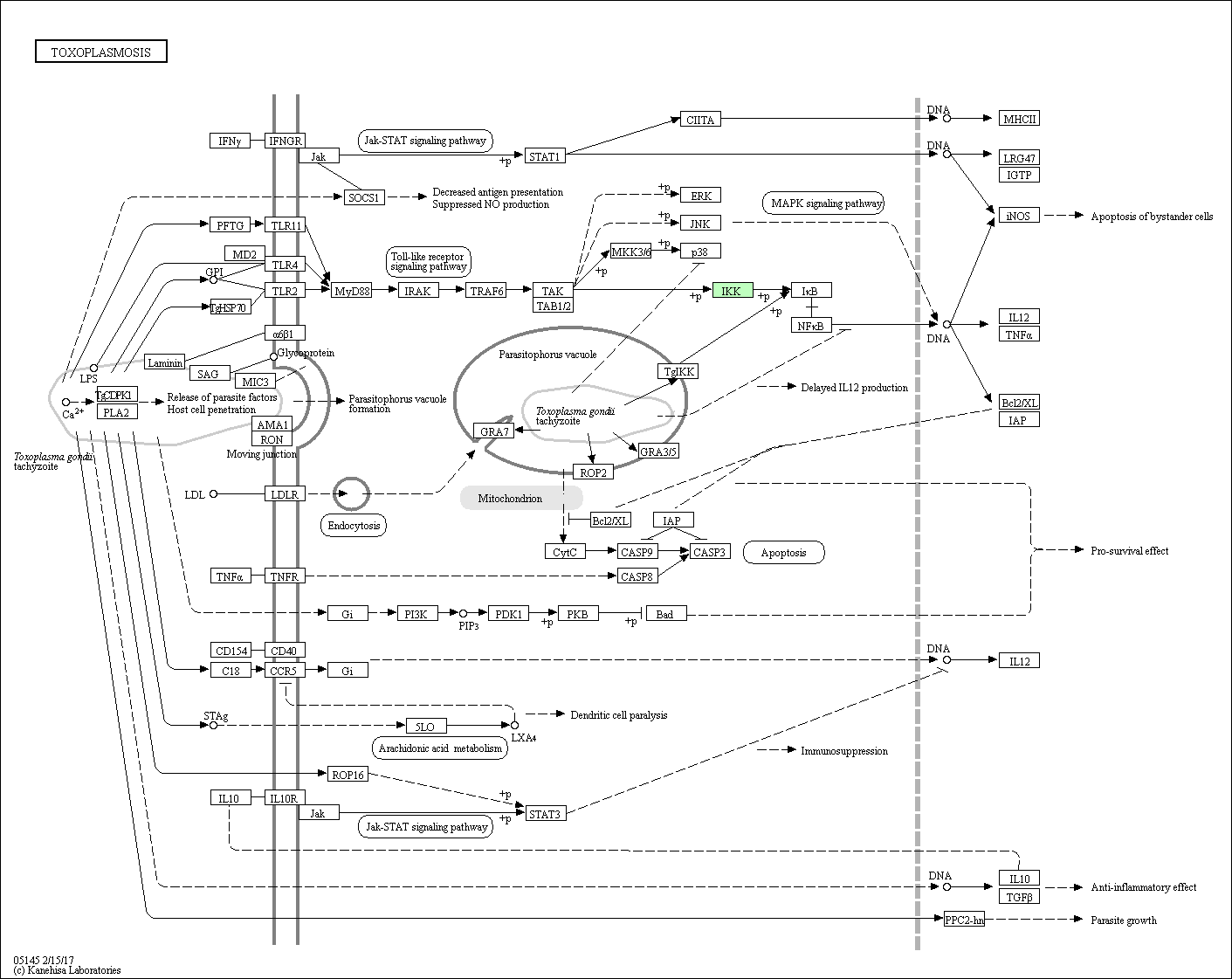

Supplement: Data S1. Data file of exported proteomics datasets, related to Figure 1 [file mmc2.zip › Date S1/1-M-GSGC0160906正式实验报告/KEGG分析结果文件夹/map/map05145.png]

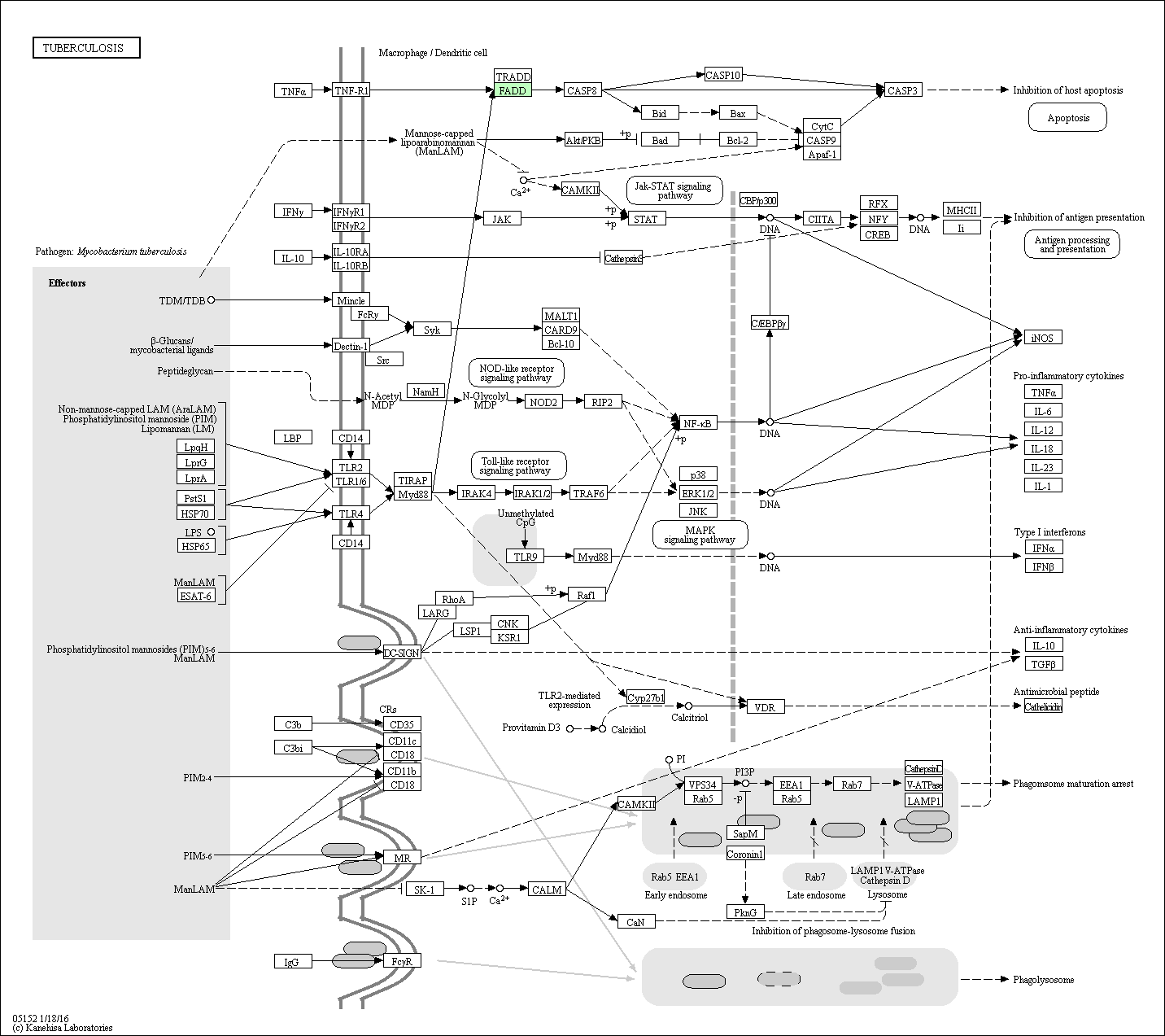

Supplement: Data S1. Data file of exported proteomics datasets, related to Figure 1 [file mmc2.zip › Date S1/1-M-GSGC0160906正式实验报告/KEGG分析结果文件夹/map/map05152.png]

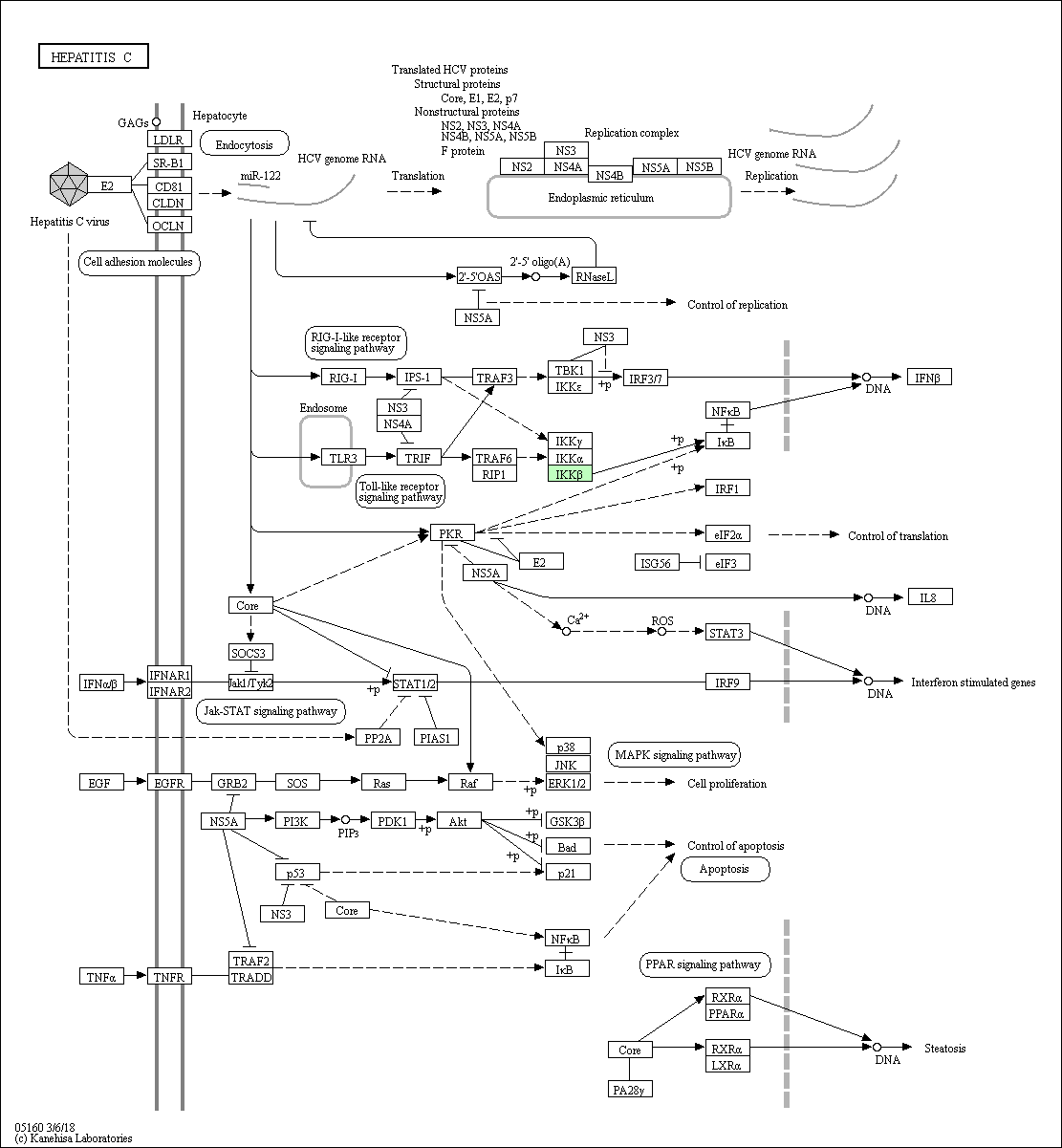

Supplement: Data S1. Data file of exported proteomics datasets, related to Figure 1 [file mmc2.zip › Date S1/1-M-GSGC0160906正式实验报告/KEGG分析结果文件夹/map/map05160.png]

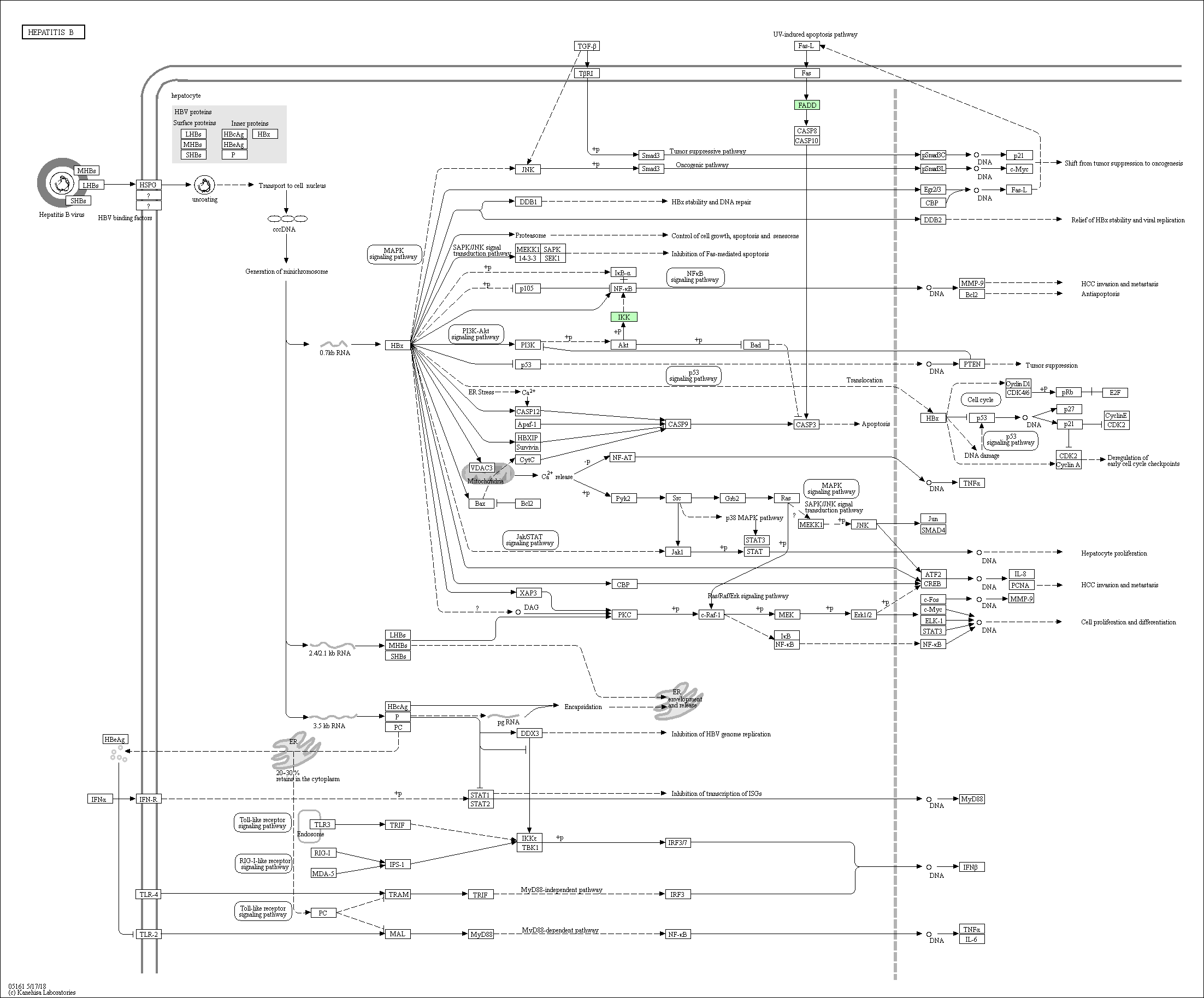

Supplement: Data S1. Data file of exported proteomics datasets, related to Figure 1 [file mmc2.zip › Date S1/1-M-GSGC0160906正式实验报告/KEGG分析结果文件夹/map/map05161.png]

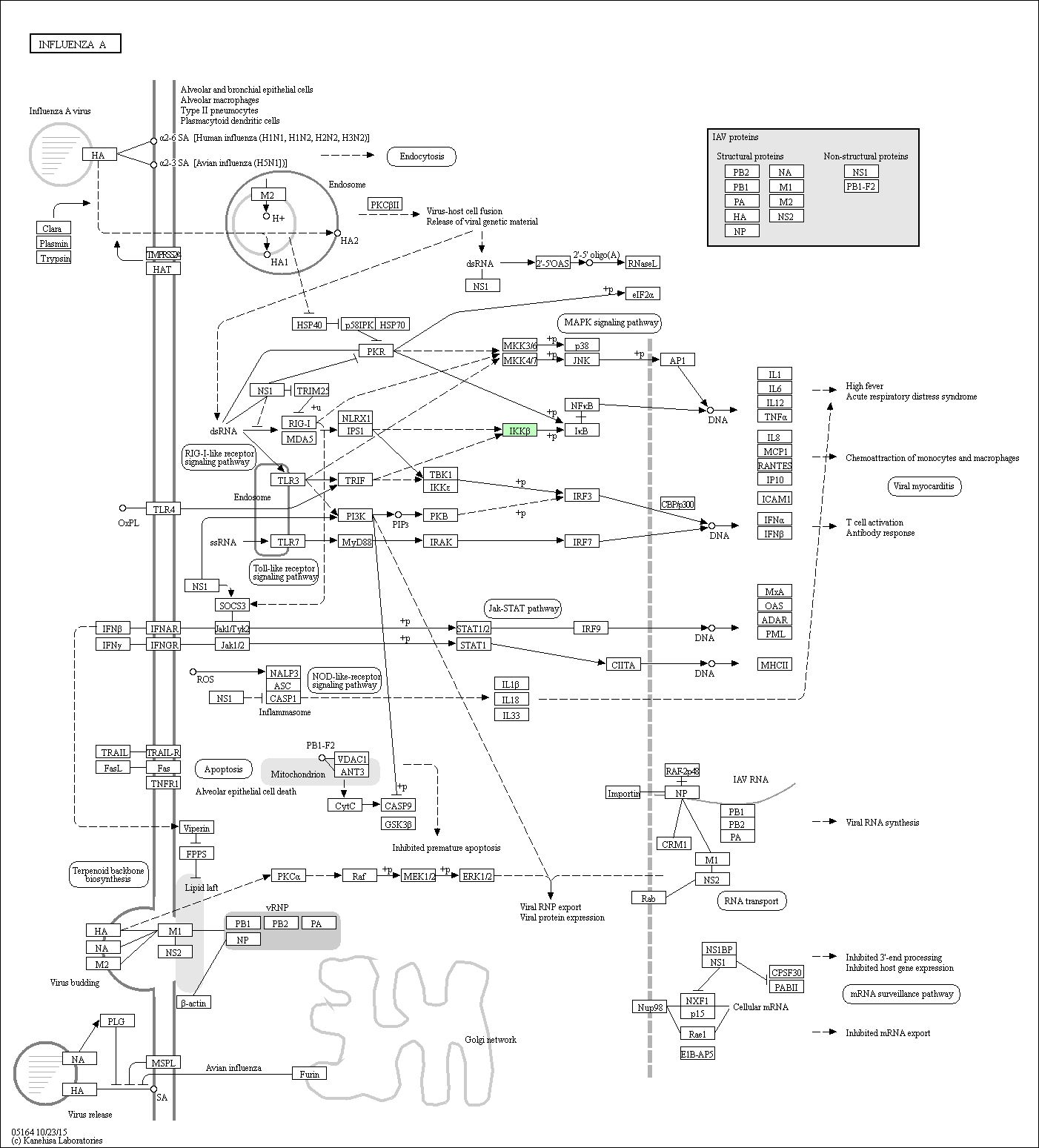

Supplement: Data S1. Data file of exported proteomics datasets, related to Figure 1 [file mmc2.zip › Date S1/1-M-GSGC0160906正式实验报告/KEGG分析结果文件夹/map/map05164.png]

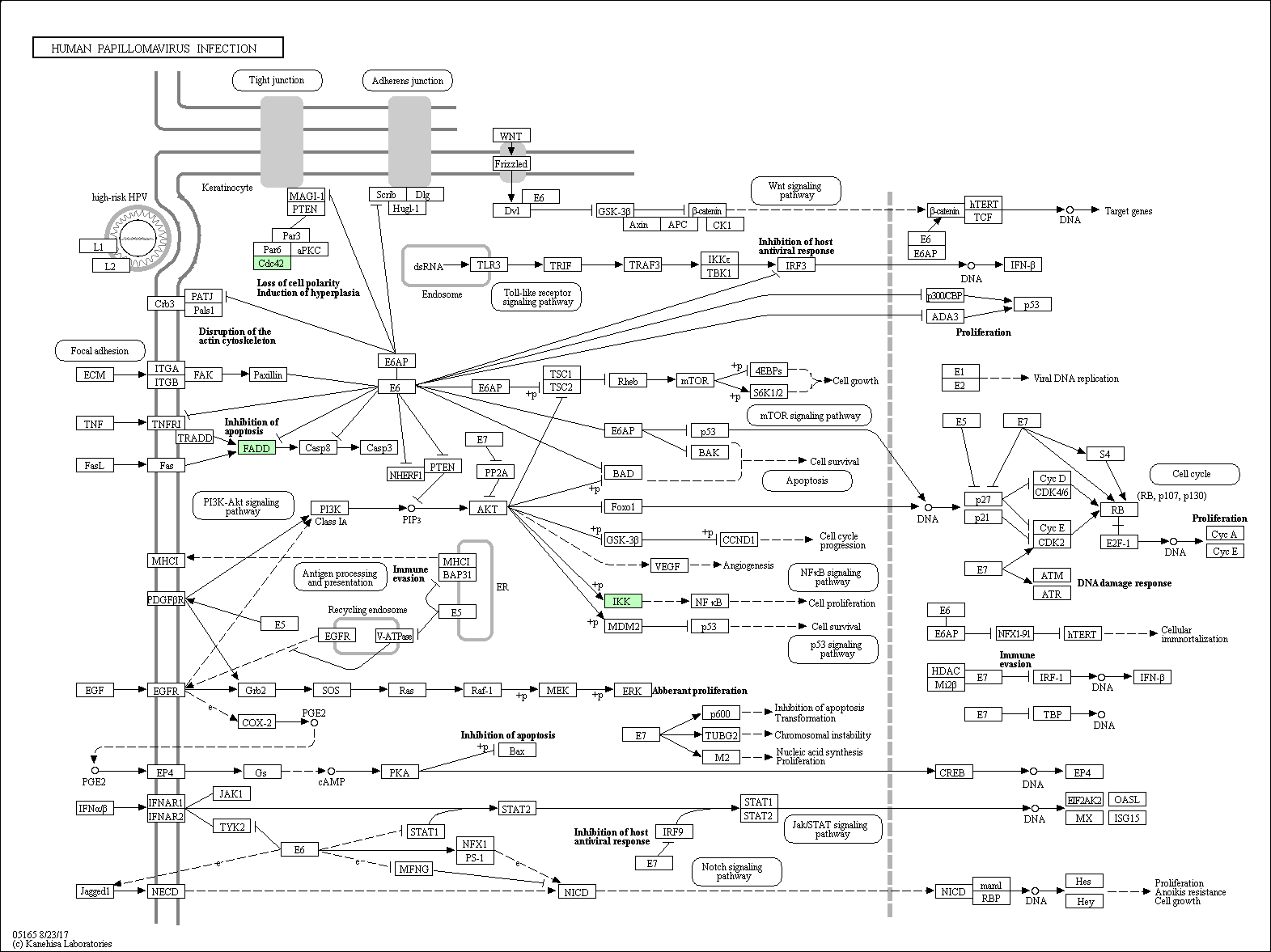

Supplement: Data S1. Data file of exported proteomics datasets, related to Figure 1 [file mmc2.zip › Date S1/1-M-GSGC0160906正式实验报告/KEGG分析结果文件夹/map/map05165.png]

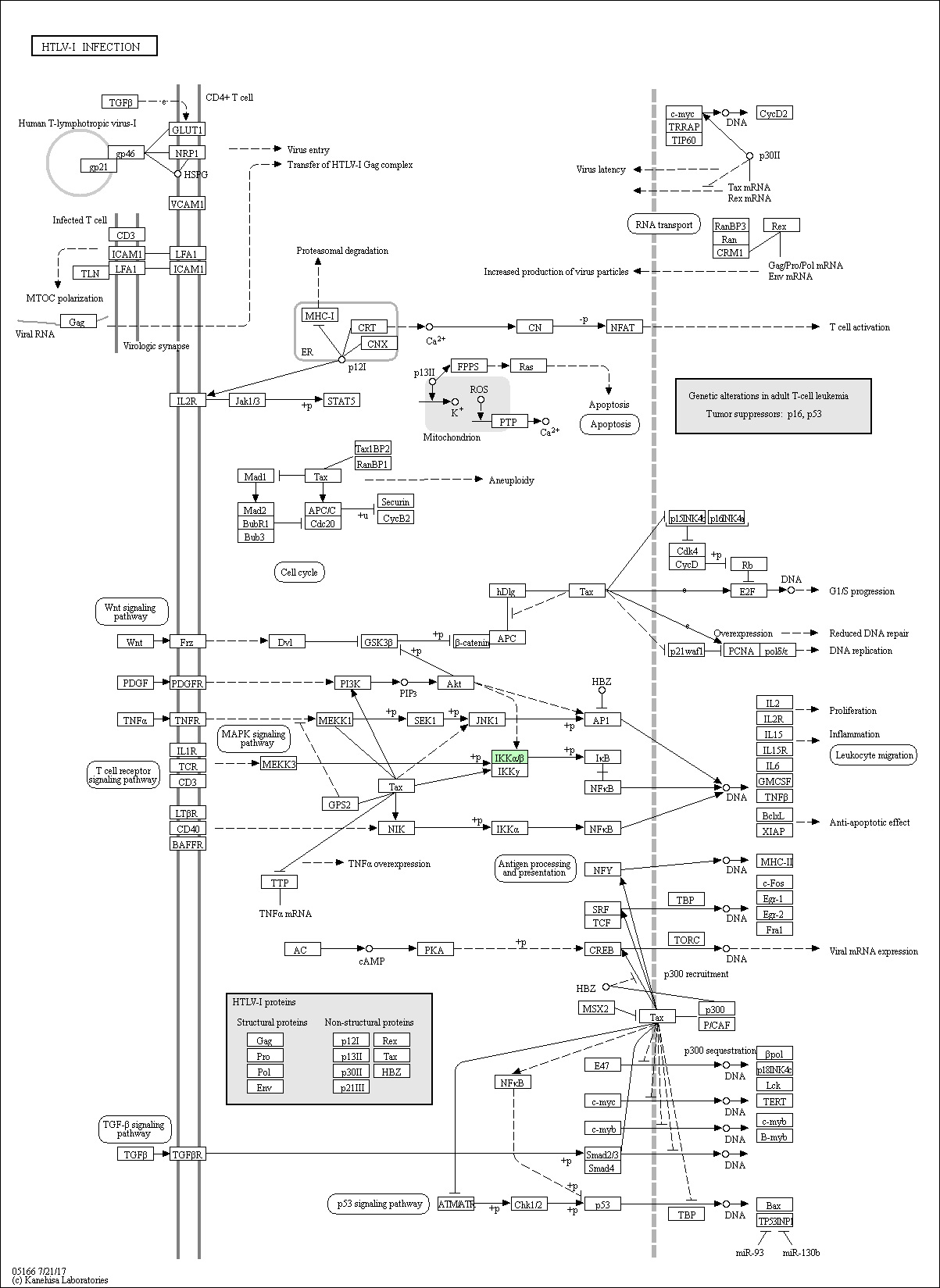

Supplement: Data S1. Data file of exported proteomics datasets, related to Figure 1 [file mmc2.zip › Date S1/1-M-GSGC0160906正式实验报告/KEGG分析结果文件夹/map/map05166.png]

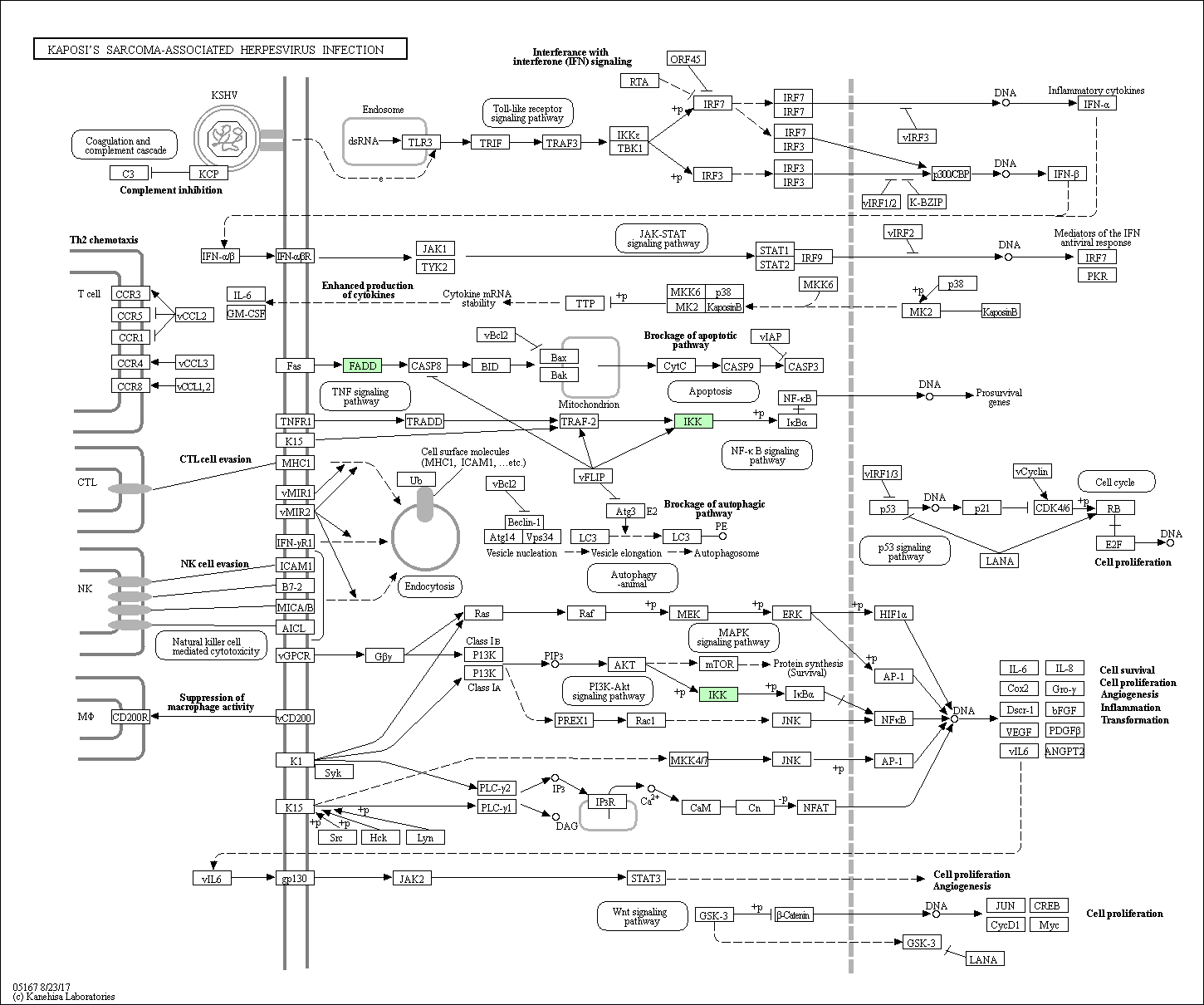

Supplement: Data S1. Data file of exported proteomics datasets, related to Figure 1 [file mmc2.zip › Date S1/1-M-GSGC0160906正式实验报告/KEGG分析结果文件夹/map/map05167.png]

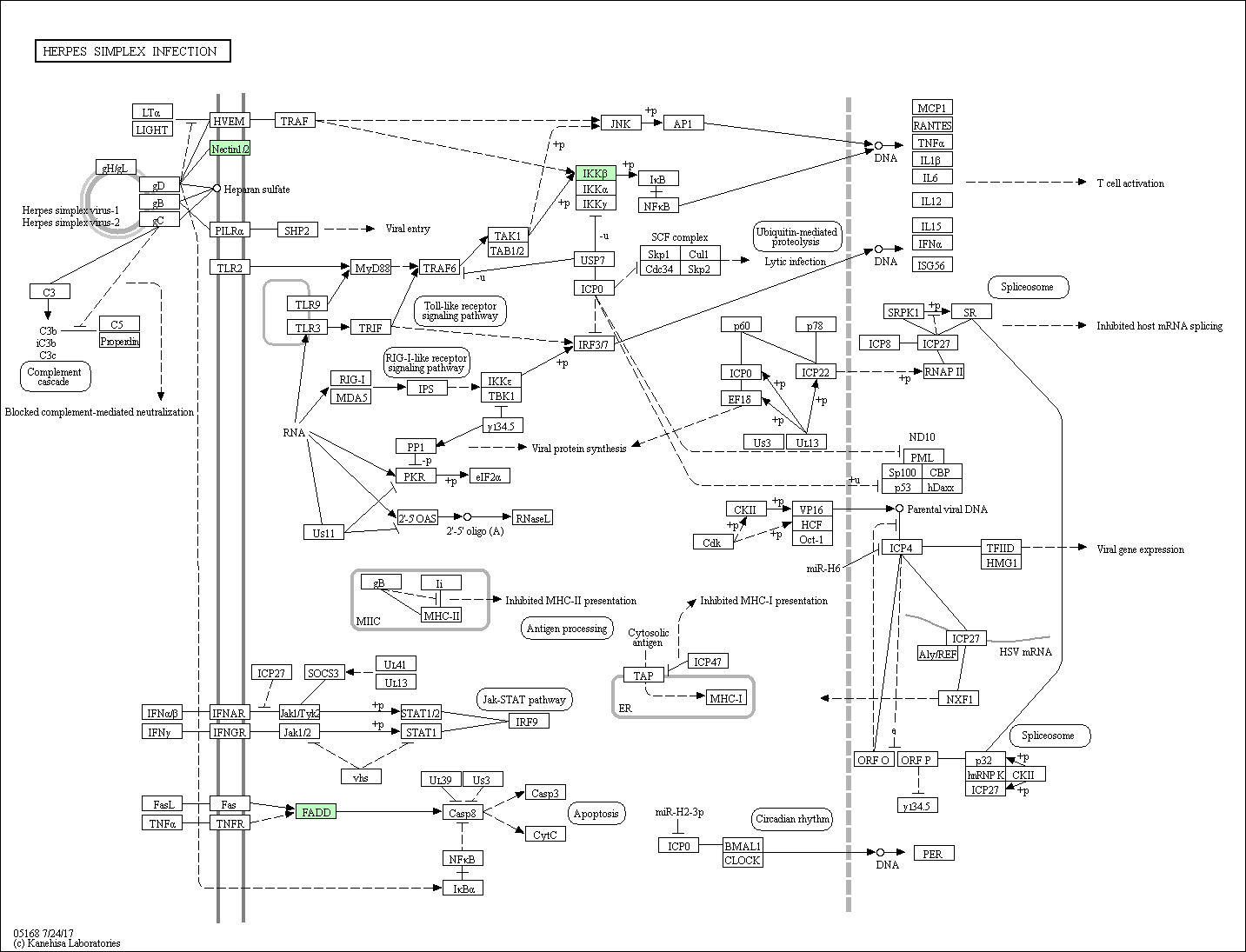

Supplement: Data S1. Data file of exported proteomics datasets, related to Figure 1 [file mmc2.zip › Date S1/1-M-GSGC0160906正式实验报告/KEGG分析结果文件夹/map/map05168.png]

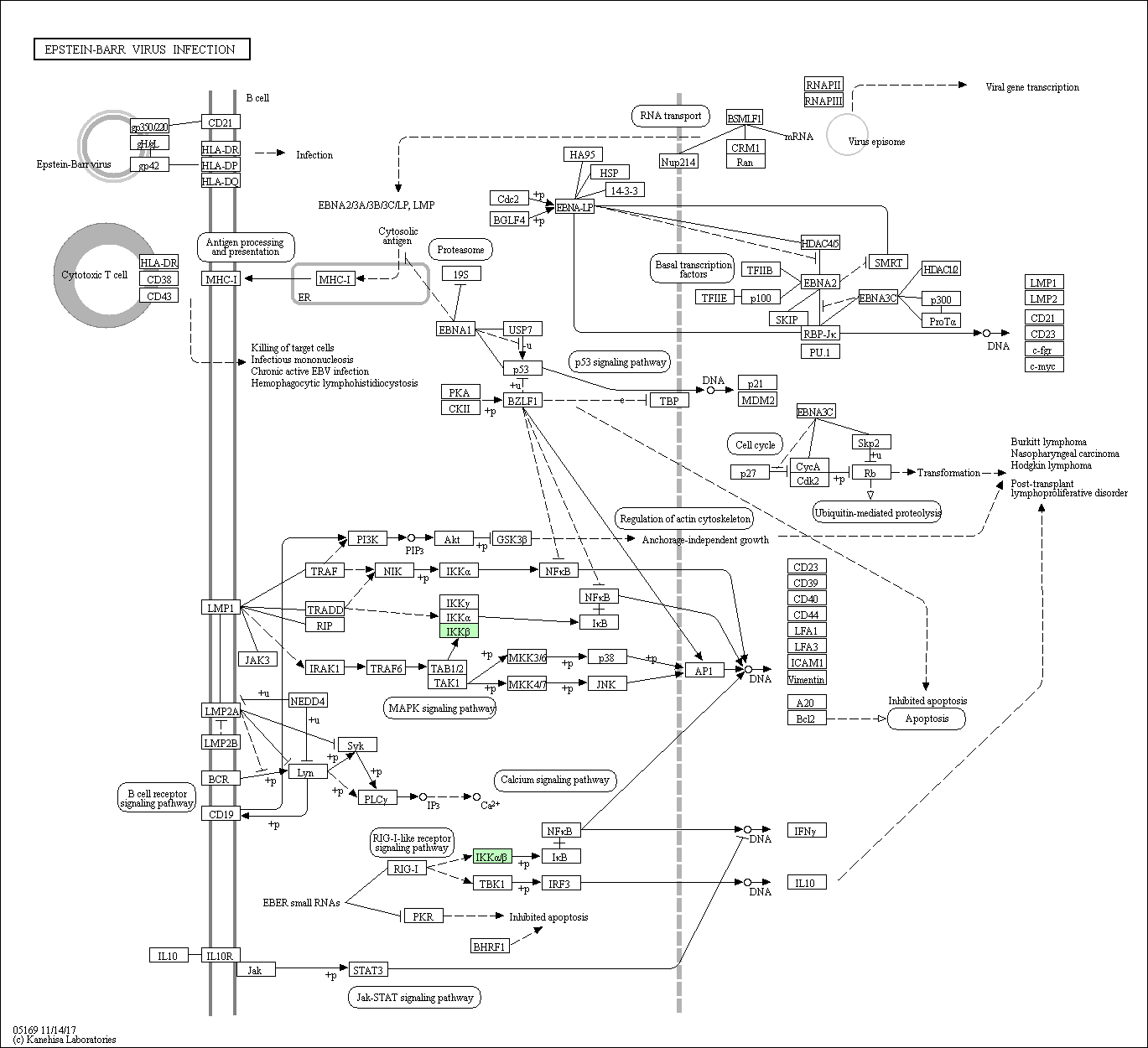

Supplement: Data S1. Data file of exported proteomics datasets, related to Figure 1 [file mmc2.zip › Date S1/1-M-GSGC0160906正式实验报告/KEGG分析结果文件夹/map/map05169.png]

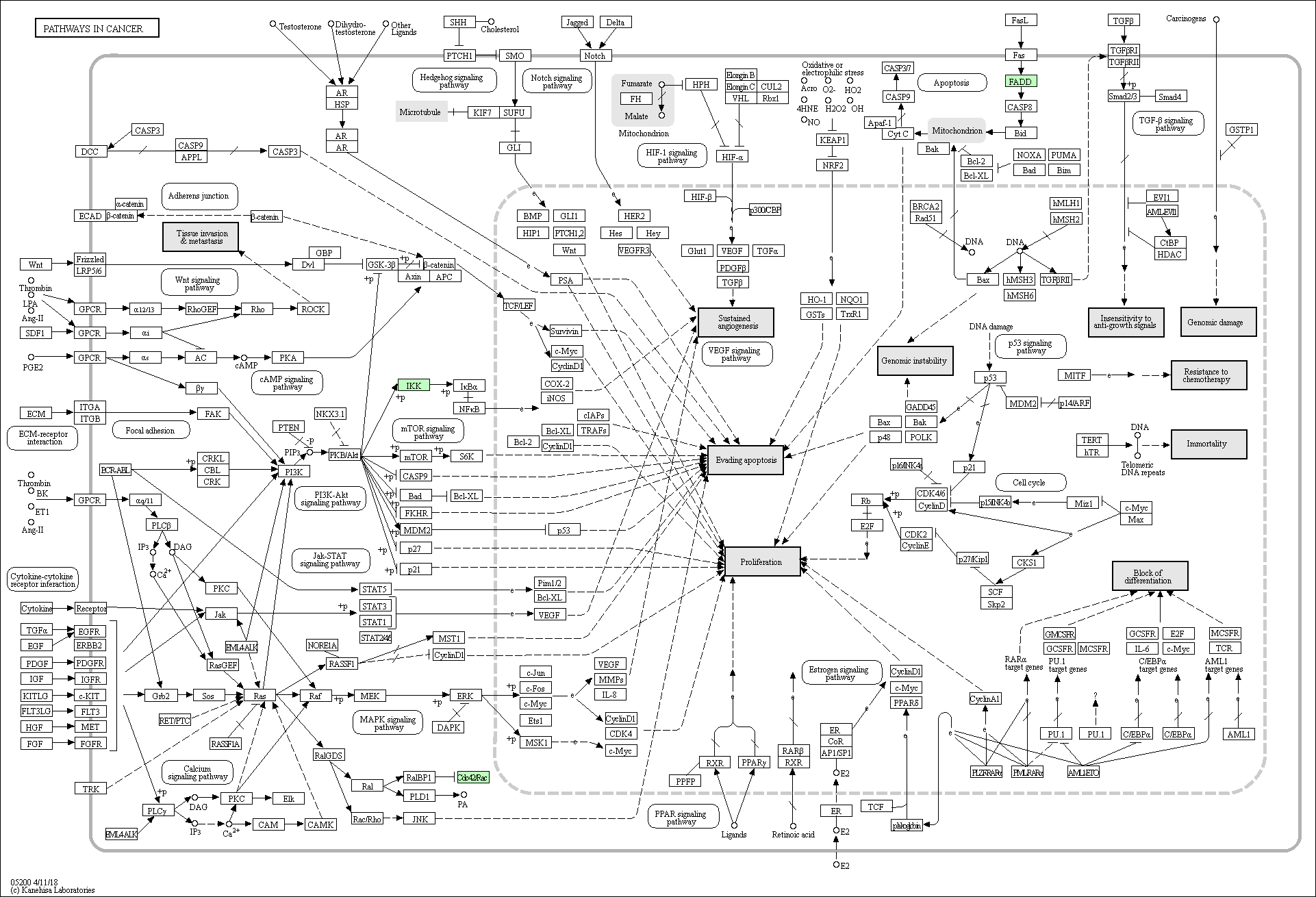

Supplement: Data S1. Data file of exported proteomics datasets, related to Figure 1 [file mmc2.zip › Date S1/1-M-GSGC0160906正式实验报告/KEGG分析结果文件夹/map/map05200.png]

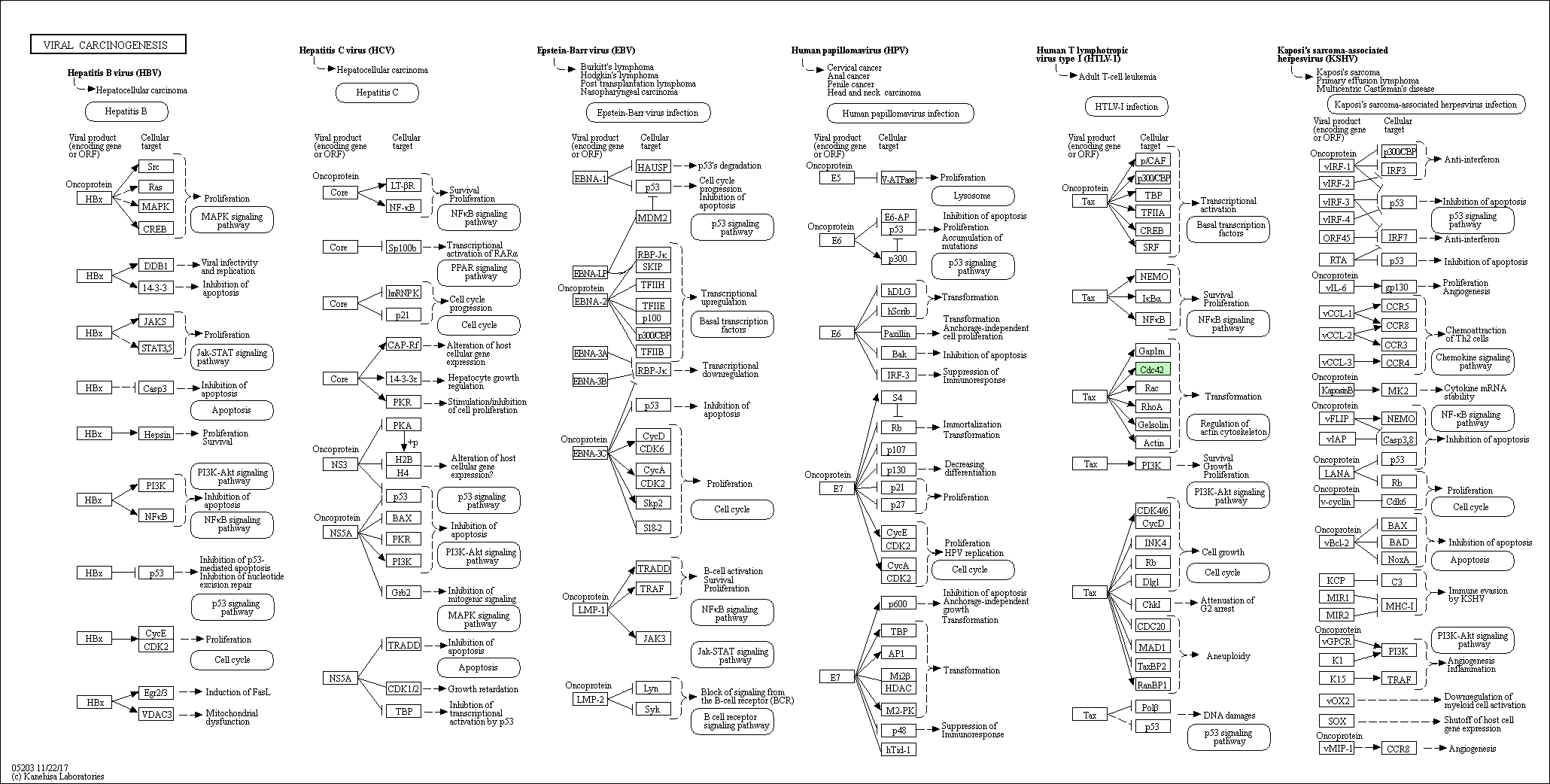

Supplement: Data S1. Data file of exported proteomics datasets, related to Figure 1 [file mmc2.zip › Date S1/1-M-GSGC0160906正式实验报告/KEGG分析结果文件夹/map/map05203.png]

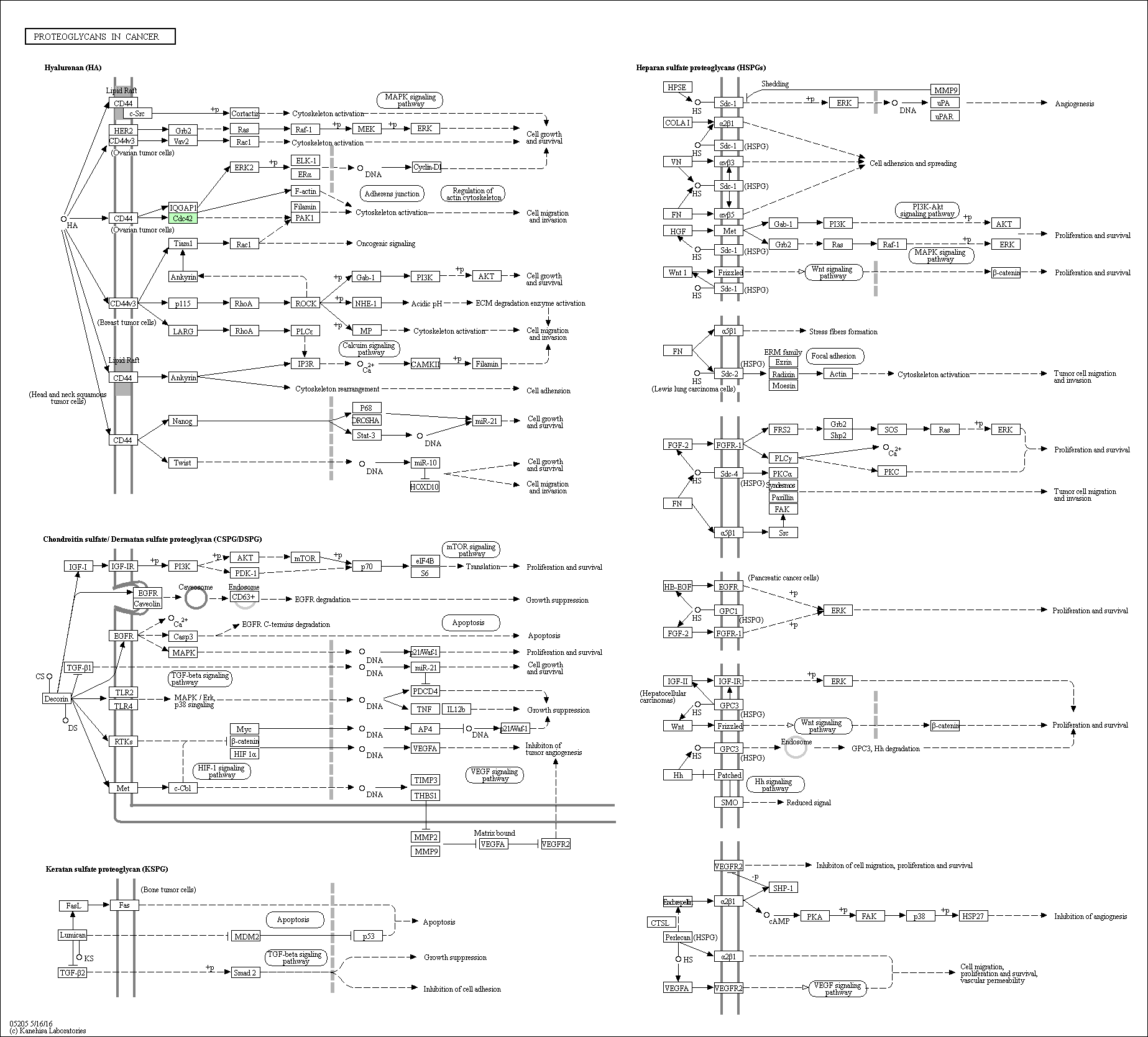

Supplement: Data S1. Data file of exported proteomics datasets, related to Figure 1 [file mmc2.zip › Date S1/1-M-GSGC0160906正式实验报告/KEGG分析结果文件夹/map/map05205.png]

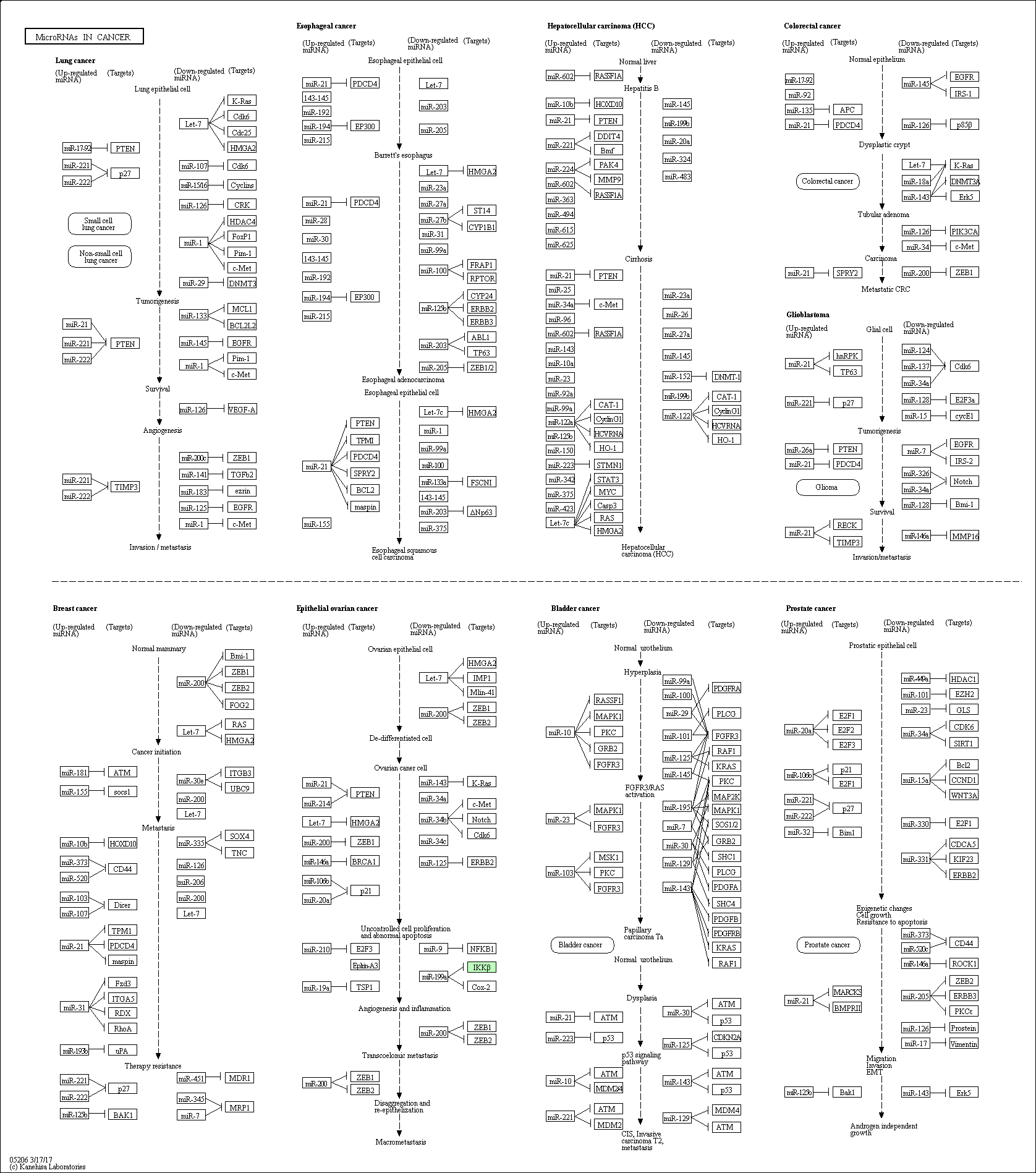

Supplement: Data S1. Data file of exported proteomics datasets, related to Figure 1 [file mmc2.zip › Date S1/1-M-GSGC0160906正式实验报告/KEGG分析结果文件夹/map/map05206.png]

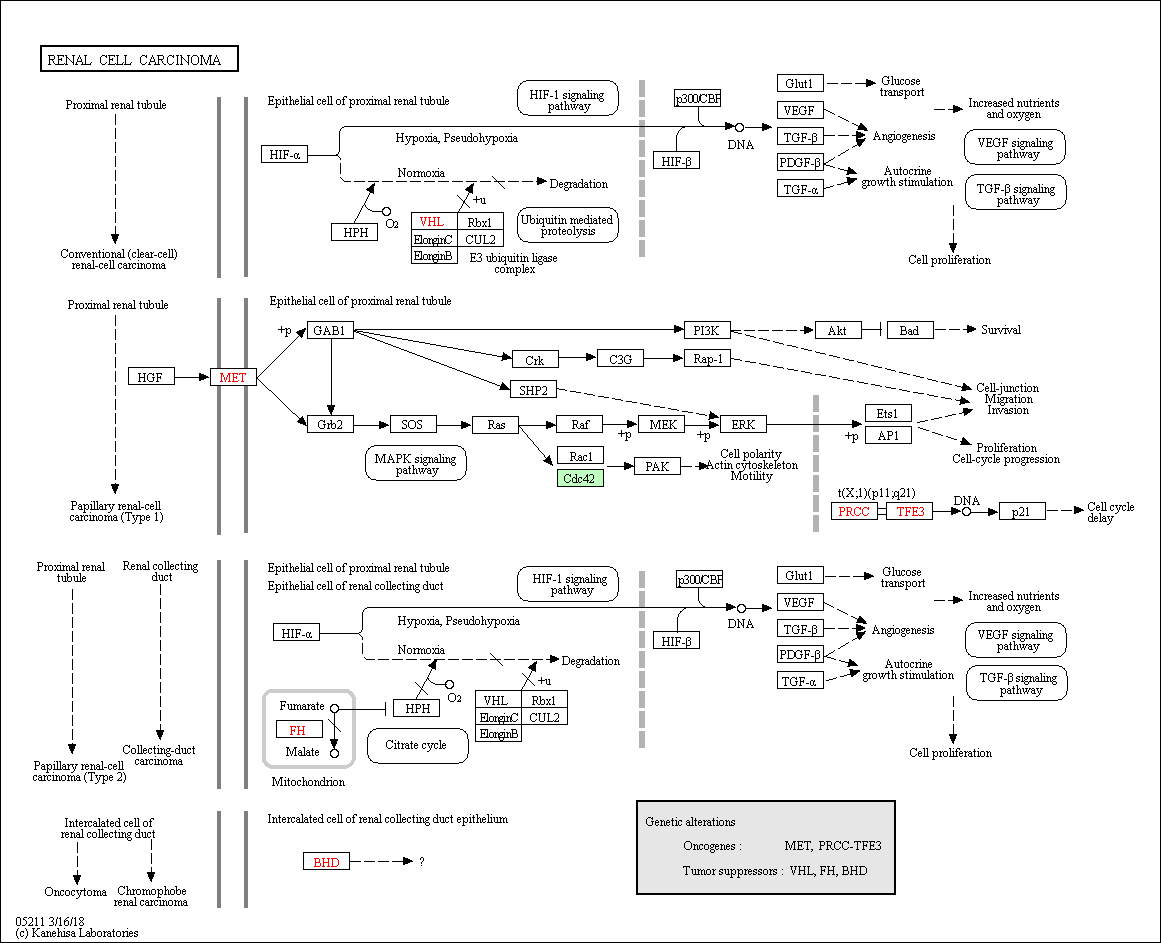

Supplement: Data S1. Data file of exported proteomics datasets, related to Figure 1 [file mmc2.zip › Date S1/1-M-GSGC0160906正式实验报告/KEGG分析结果文件夹/map/map05211.png]

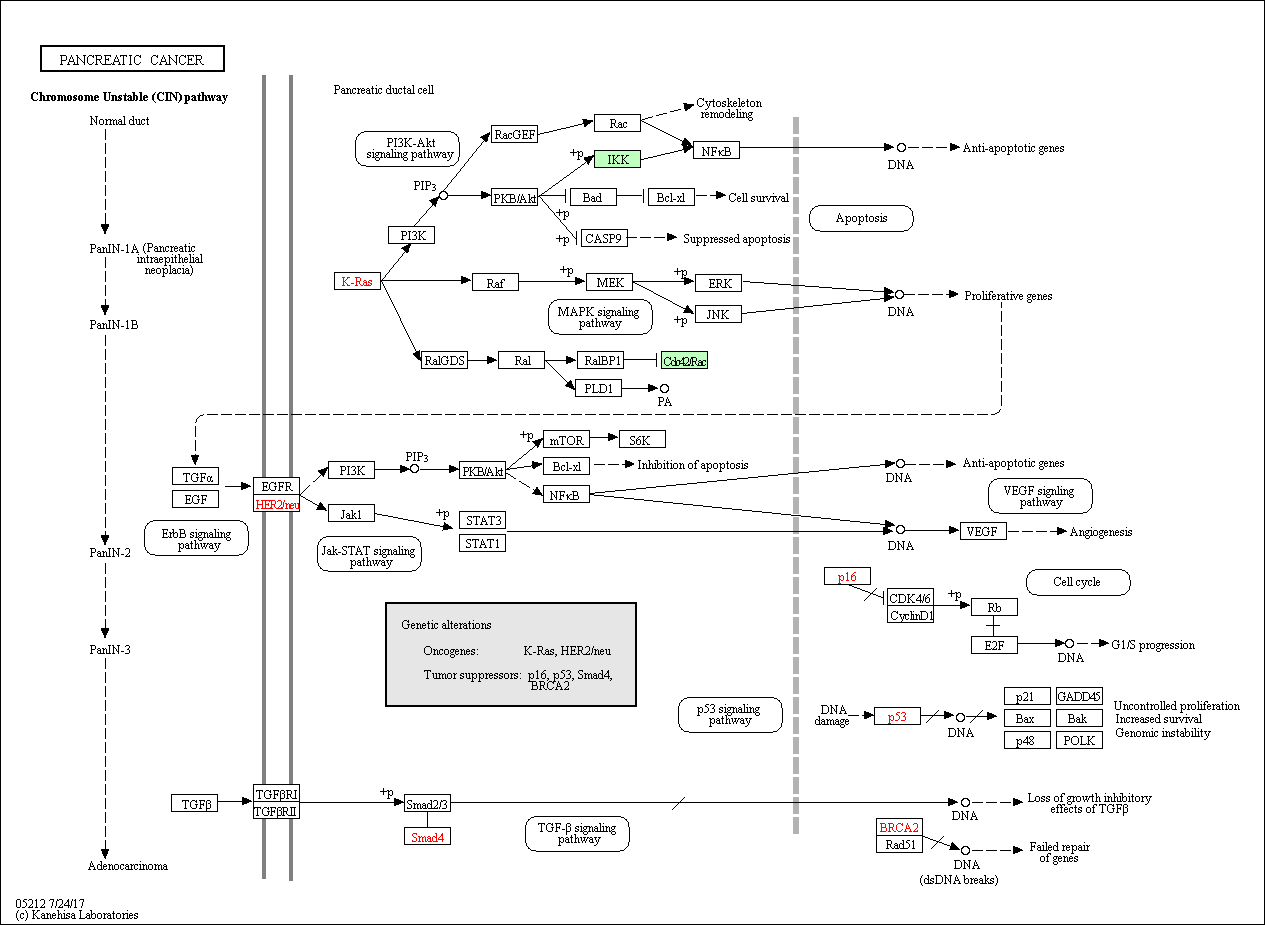

Supplement: Data S1. Data file of exported proteomics datasets, related to Figure 1 [file mmc2.zip › Date S1/1-M-GSGC0160906正式实验报告/KEGG分析结果文件夹/map/map05212.png]

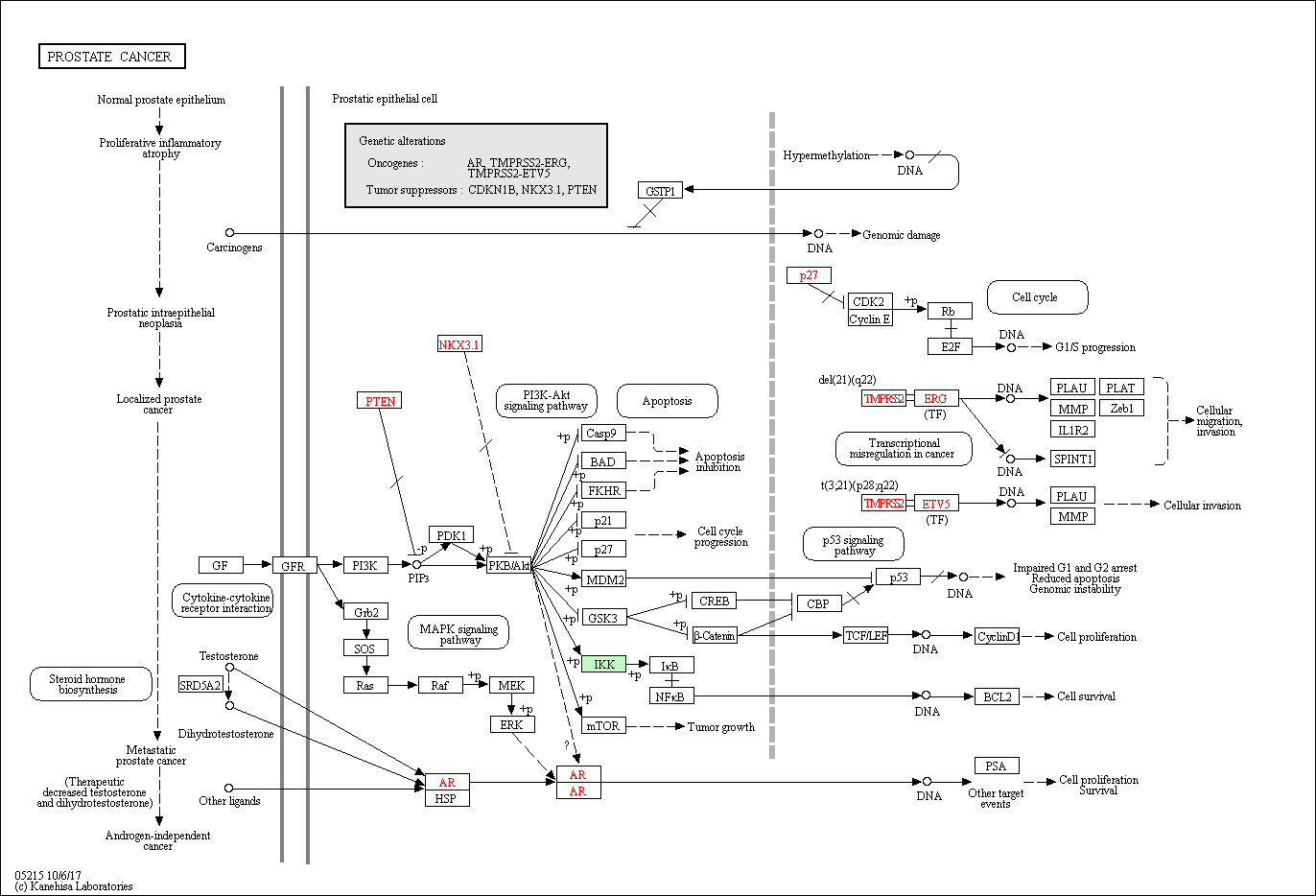

Supplement: Data S1. Data file of exported proteomics datasets, related to Figure 1 [file mmc2.zip › Date S1/1-M-GSGC0160906正式实验报告/KEGG分析结果文件夹/map/map05215.png]

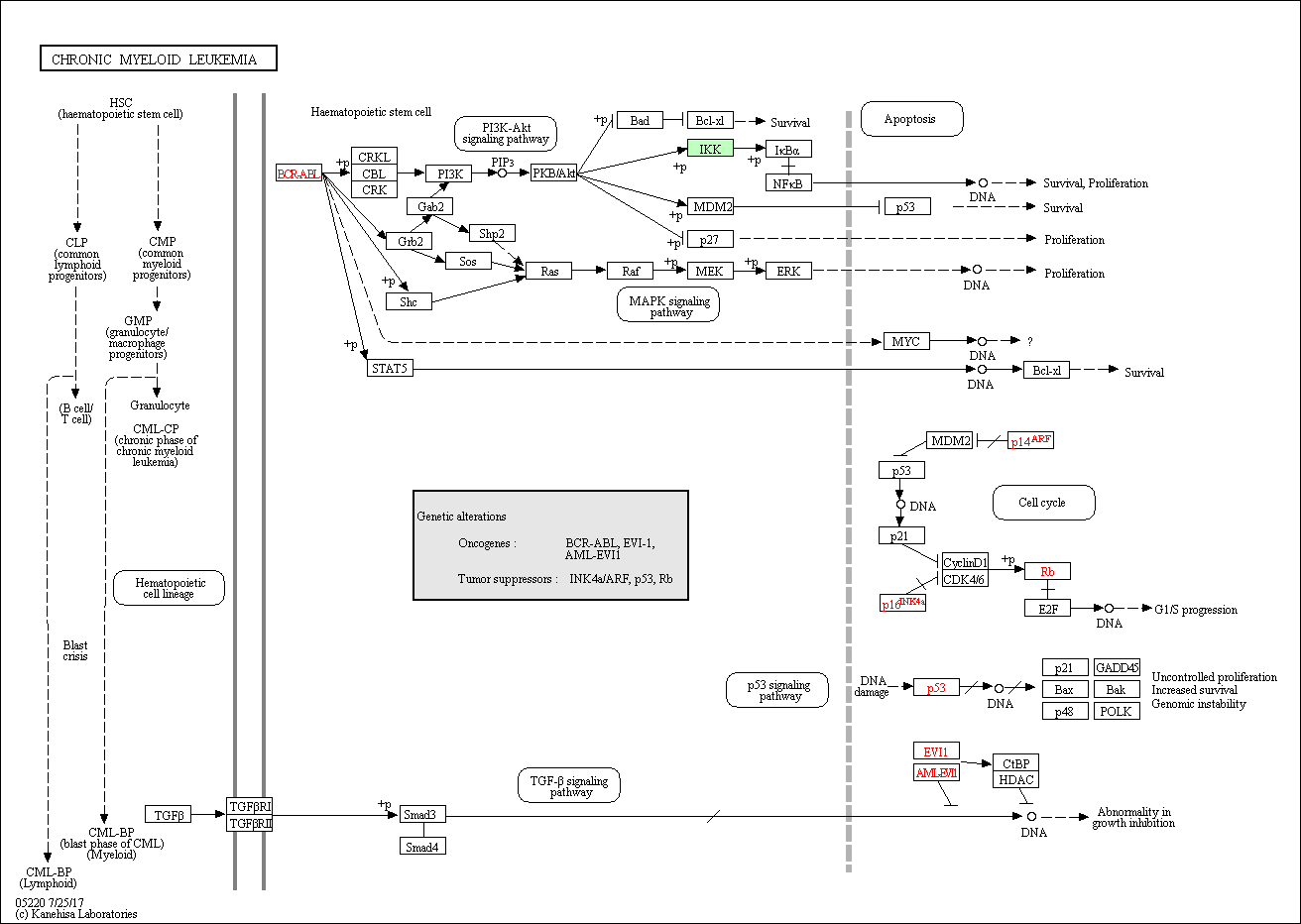

Supplement: Data S1. Data file of exported proteomics datasets, related to Figure 1 [file mmc2.zip › Date S1/1-M-GSGC0160906正式实验报告/KEGG分析结果文件夹/map/map05220.png]

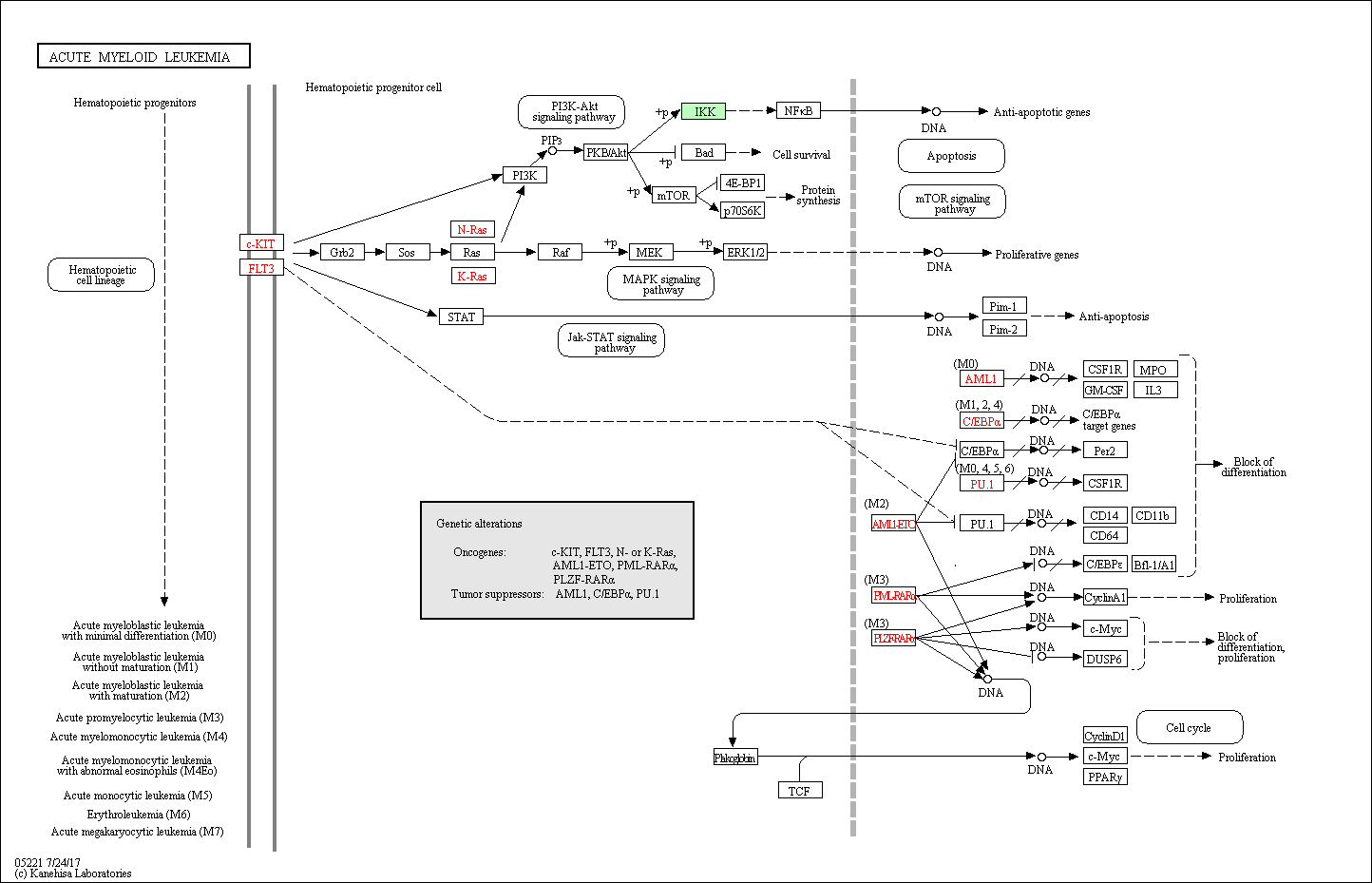

Supplement: Data S1. Data file of exported proteomics datasets, related to Figure 1 [file mmc2.zip › Date S1/1-M-GSGC0160906正式实验报告/KEGG分析结果文件夹/map/map05221.png]

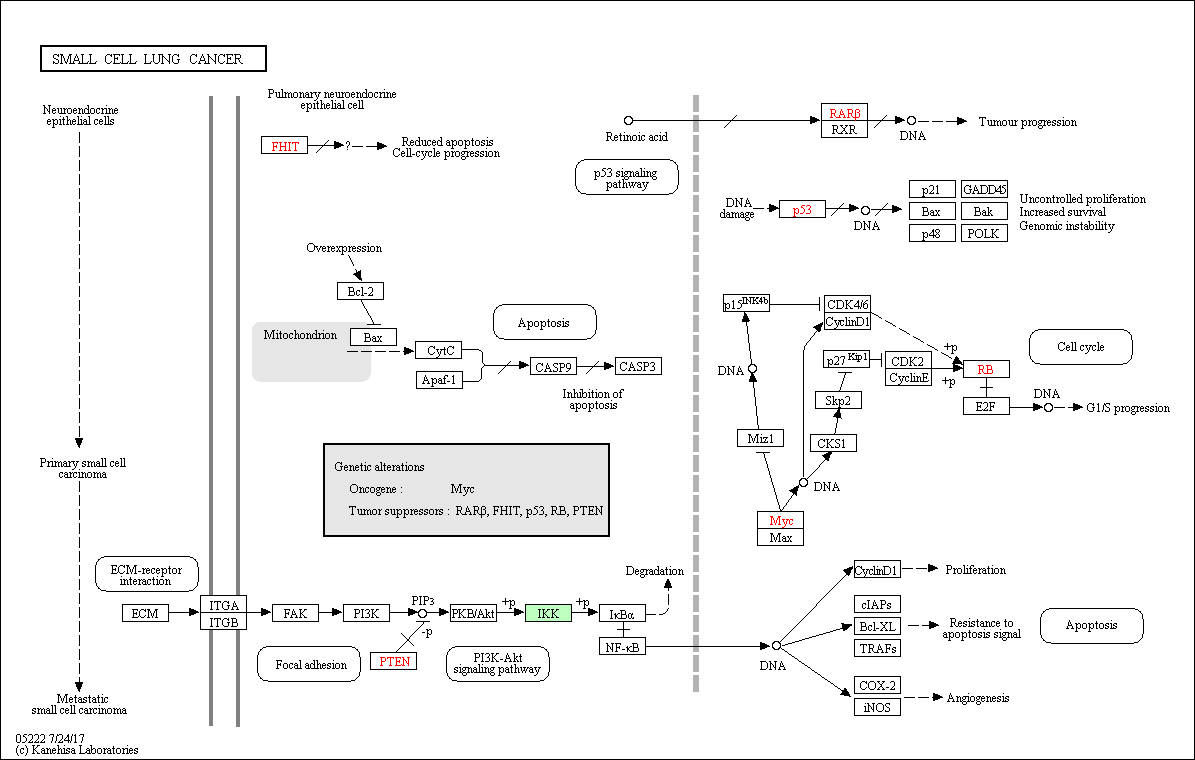

Supplement: Data S1. Data file of exported proteomics datasets, related to Figure 1 [file mmc2.zip › Date S1/1-M-GSGC0160906正式实验报告/KEGG分析结果文件夹/map/map05222.png]

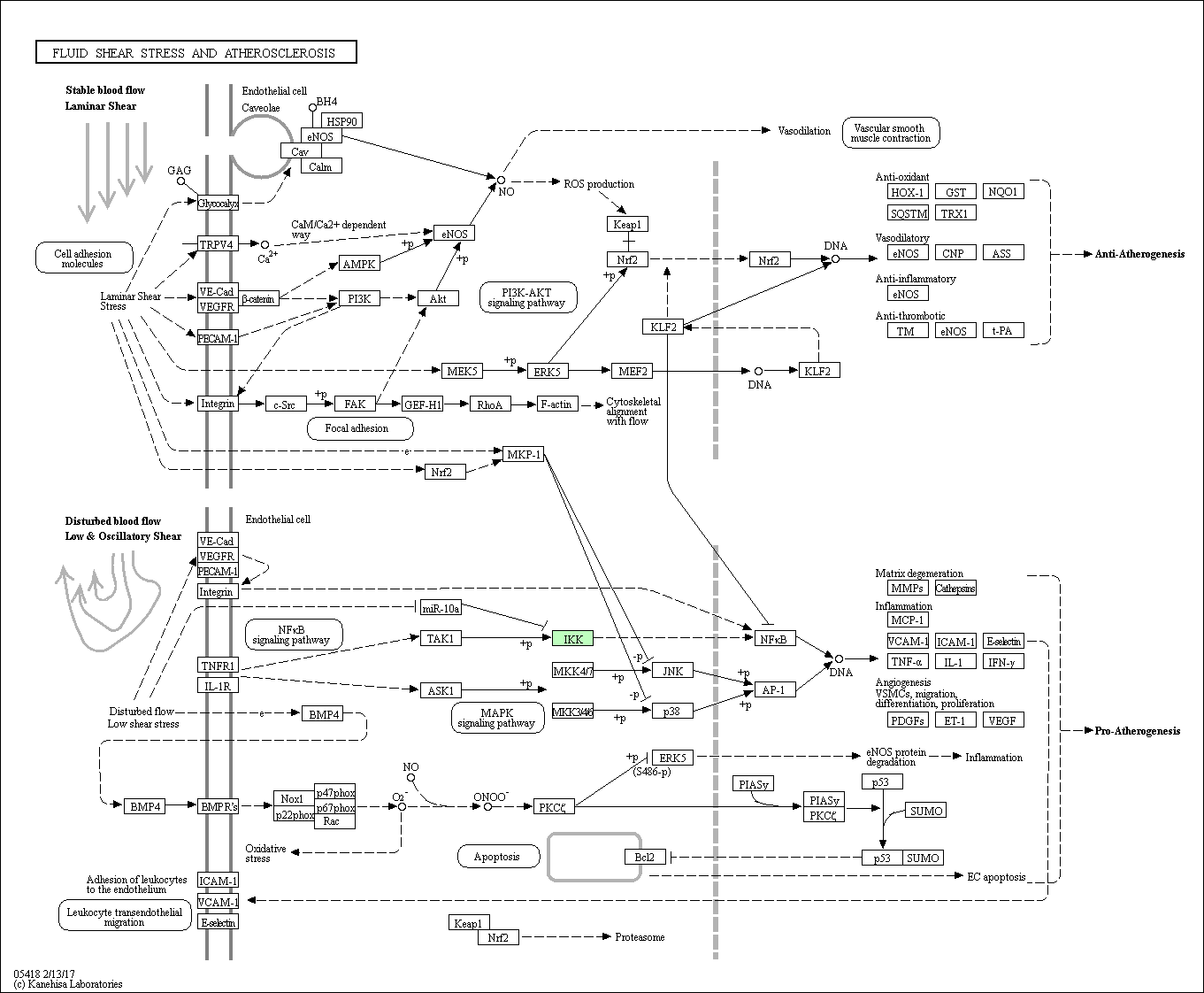

Supplement: Data S1. Data file of exported proteomics datasets, related to Figure 1 [file mmc2.zip › Date S1/1-M-GSGC0160906正式实验报告/KEGG分析结果文件夹/map/map05418.png]

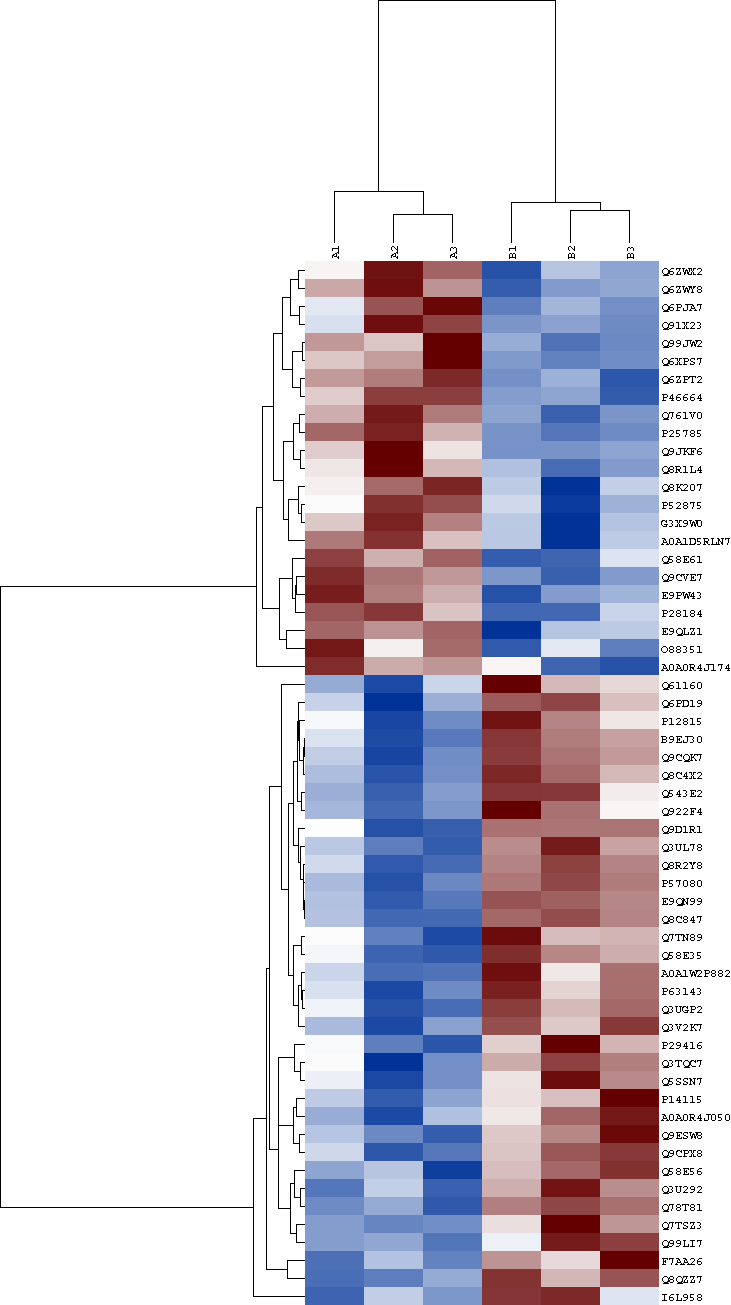

Supplement: Data S1. Data file of exported proteomics datasets, related to Figure 1 [file mmc2.zip › Date S1/1-M-GSGC0160906正式实验报告/聚类分析结果文件夹/cluster_data.png]

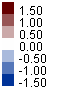

Supplement: Data S1. Data file of exported proteomics datasets, related to Figure 1 [file mmc2.zip › Date S1/1-M-GSGC0160906正式实验报告/聚类分析结果文件夹/cluster_data_colorbar.png]

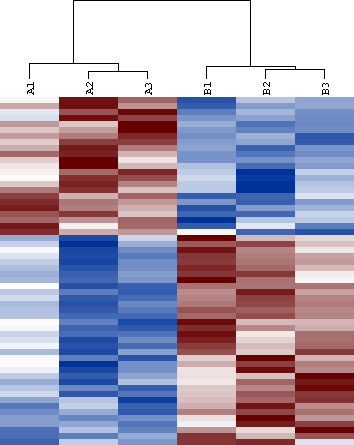

Supplement: Data S1. Data file of exported proteomics datasets, related to Figure 1 [file mmc2.zip › Date S1/1-M-GSGC0160906正式实验报告/聚类分析结果文件夹/cluster_data_smallsize.png]

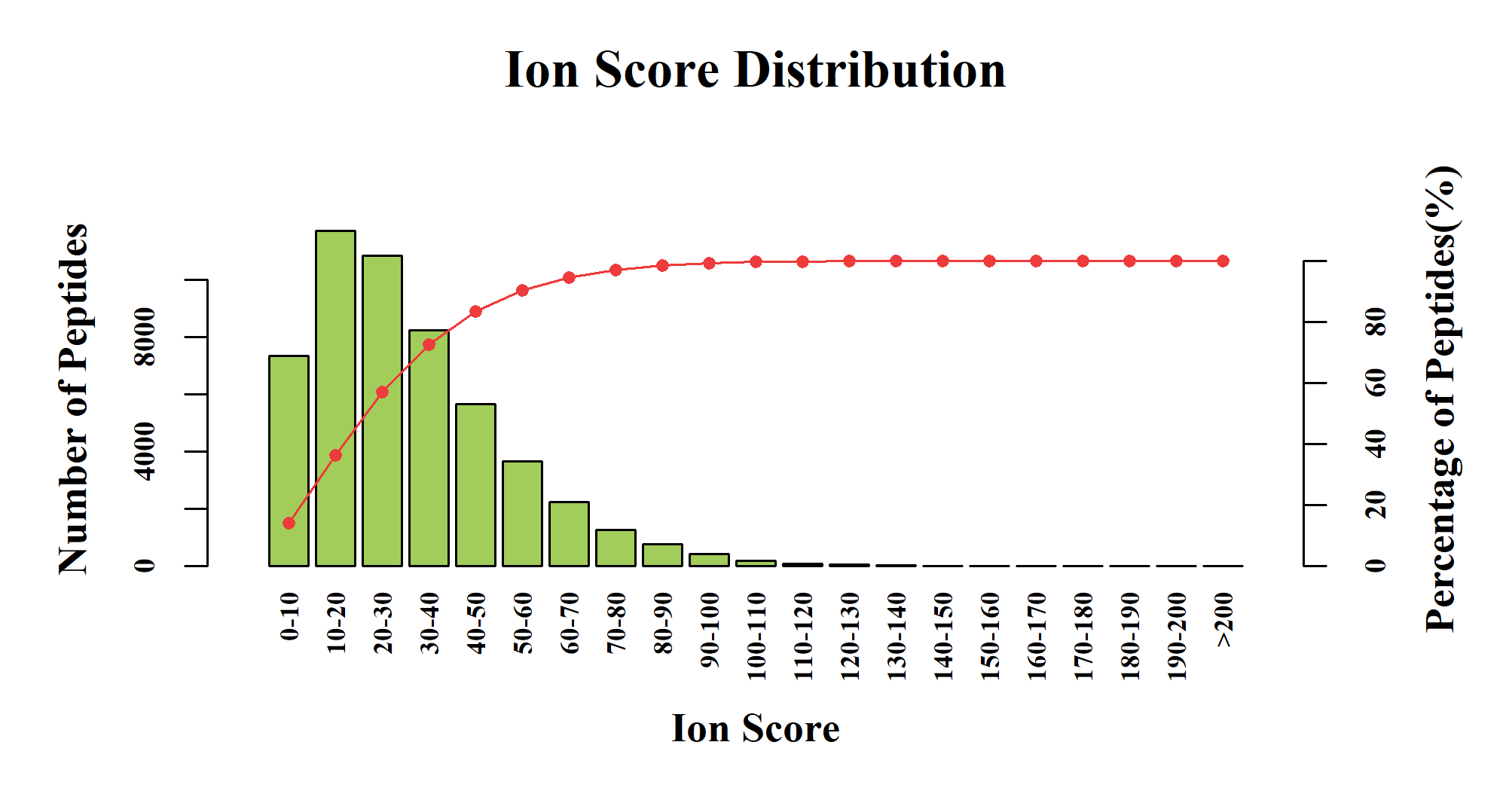

Supplement: Data S1. Data file of exported proteomics datasets, related to Figure 1 [file mmc2.zip › Date S1/2-M-GSGC0157983正式实验报告/Evaluation/图4-1 肽段离子得分分布图.tif]

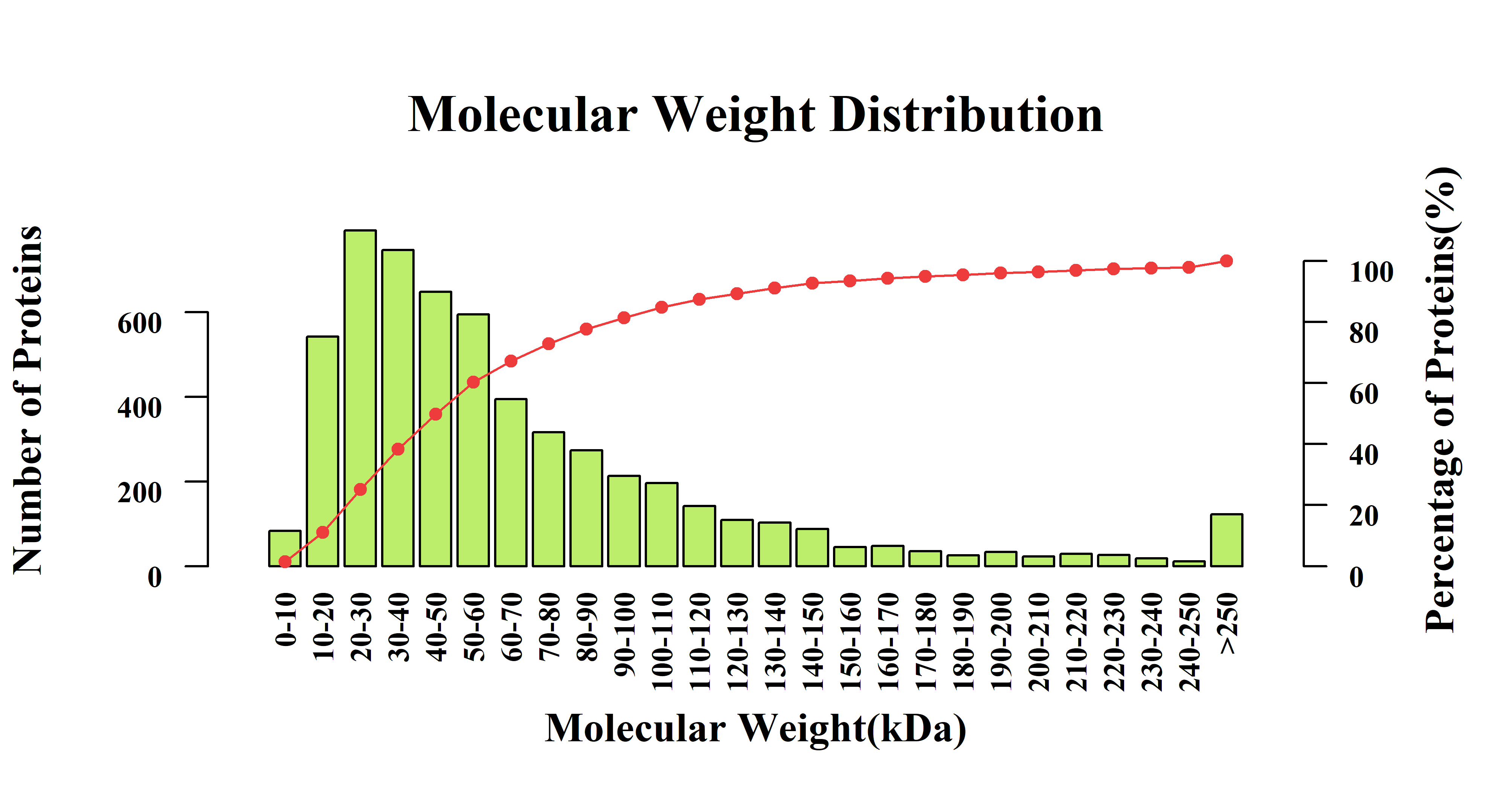

Supplement: Data S1. Data file of exported proteomics datasets, related to Figure 1 [file mmc2.zip › Date S1/2-M-GSGC0157983正式实验报告/Evaluation/图4-2 鉴定蛋白质相对分子质量分布图.tif]

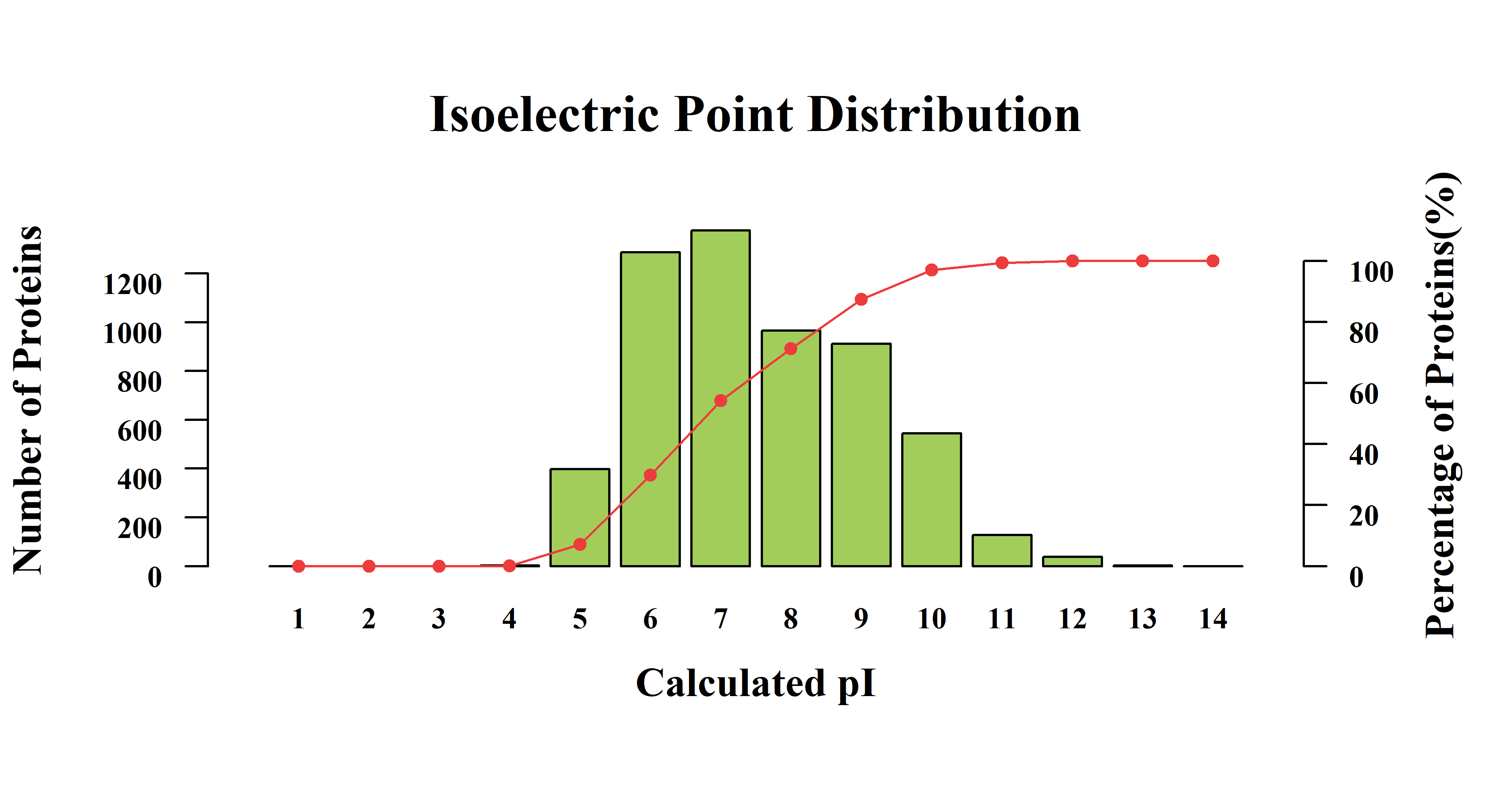

Supplement: Data S1. Data file of exported proteomics datasets, related to Figure 1 [file mmc2.zip › Date S1/2-M-GSGC0157983正式实验报告/Evaluation/图4-3 鉴定蛋白质等电点分布图.tif]

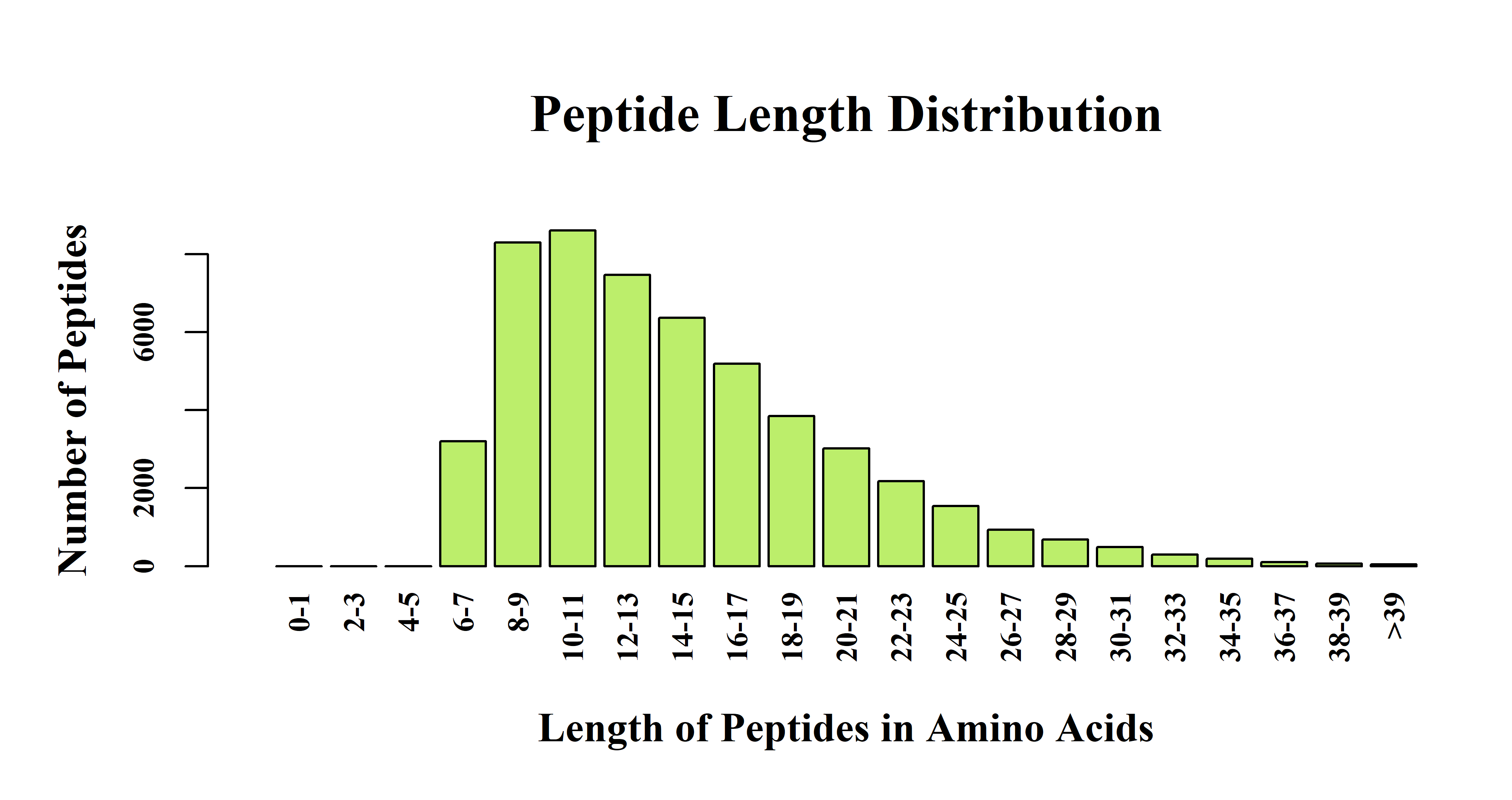

Supplement: Data S1. Data file of exported proteomics datasets, related to Figure 1 [file mmc2.zip › Date S1/2-M-GSGC0157983正式实验报告/Evaluation/图4-4 肽段序列长度分布图.tif]

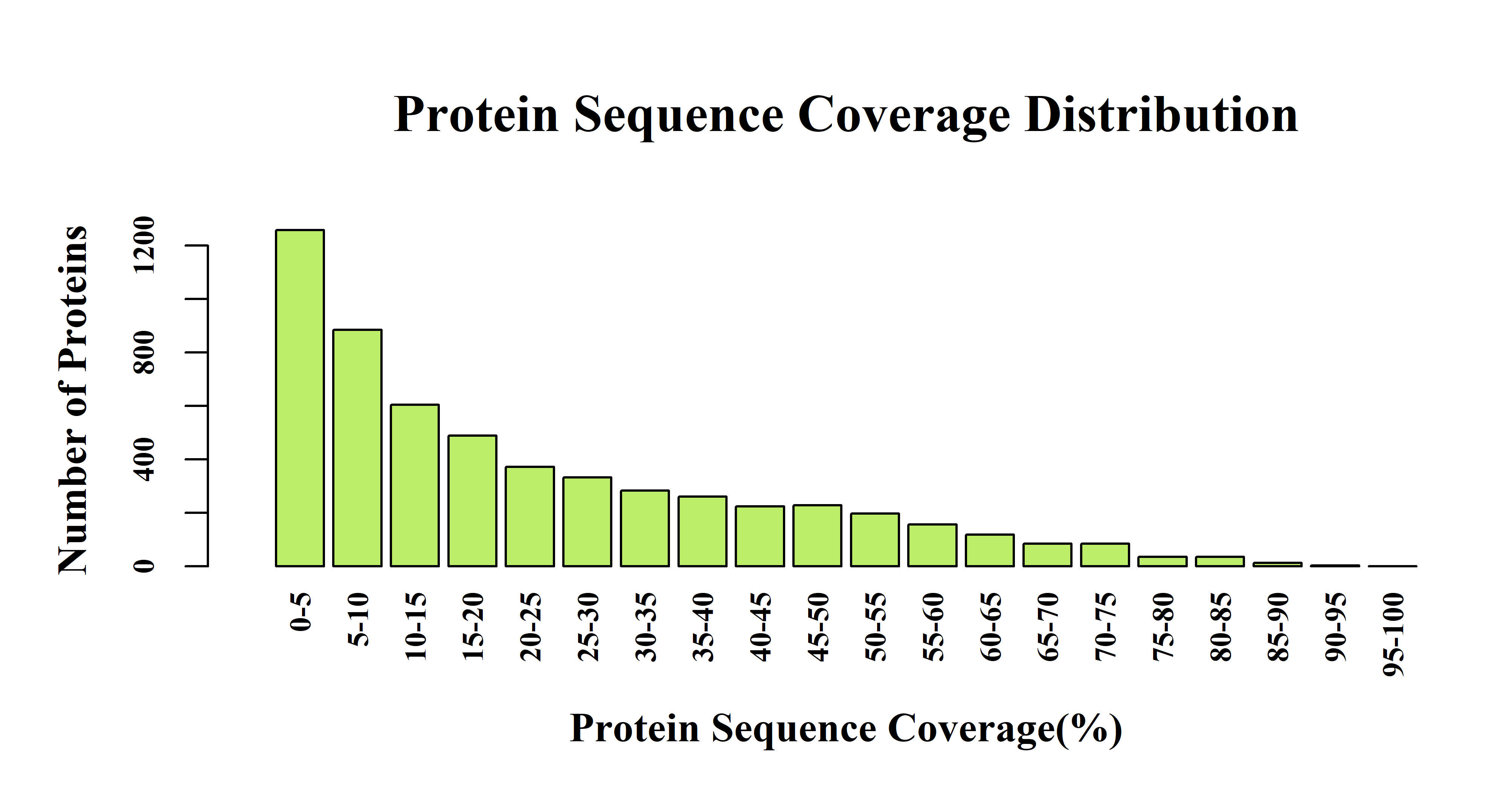

Supplement: Data S1. Data file of exported proteomics datasets, related to Figure 1 [file mmc2.zip › Date S1/2-M-GSGC0157983正式实验报告/Evaluation/图4-5 蛋白质序列覆盖度分布图.tif]

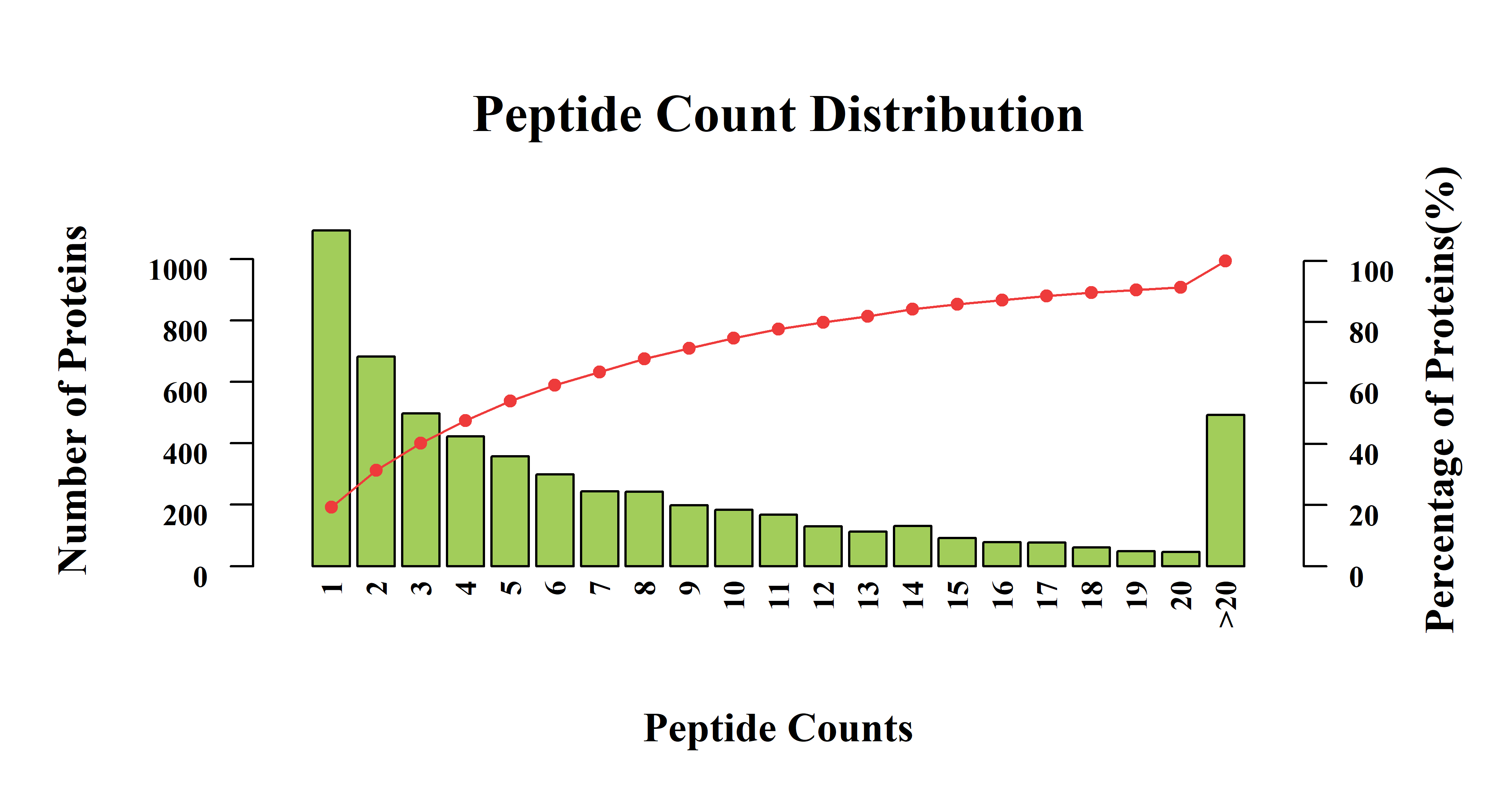

Supplement: Data S1. Data file of exported proteomics datasets, related to Figure 1 [file mmc2.zip › Date S1/2-M-GSGC0157983正式实验报告/Evaluation/图4-6 鉴定肽段数量分布图.tif]

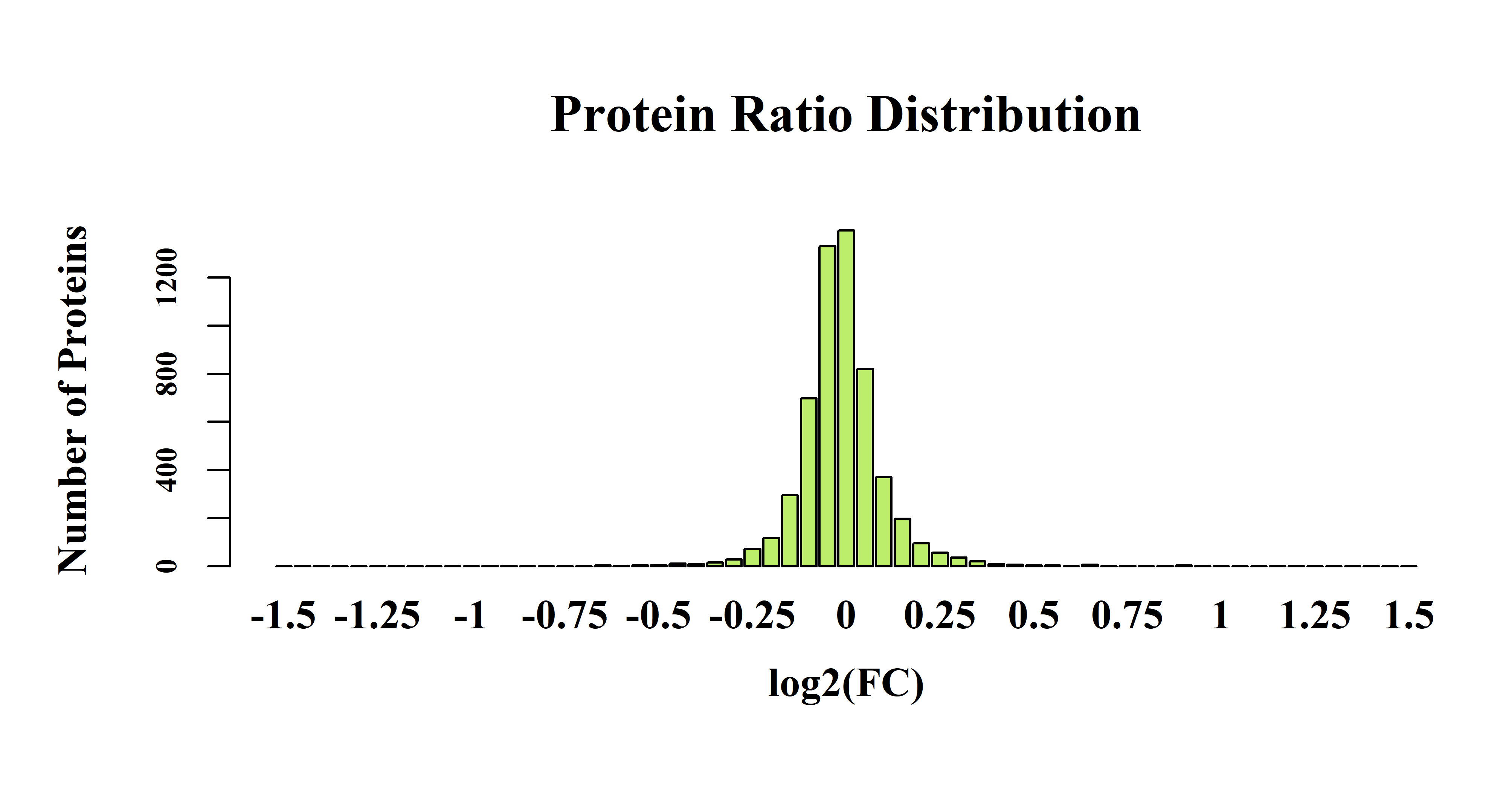

Supplement: Data S1. Data file of exported proteomics datasets, related to Figure 1 [file mmc2.zip › Date S1/2-M-GSGC0157983正式实验报告/Evaluation/图4-7 蛋白质丰度比分布图.tif]

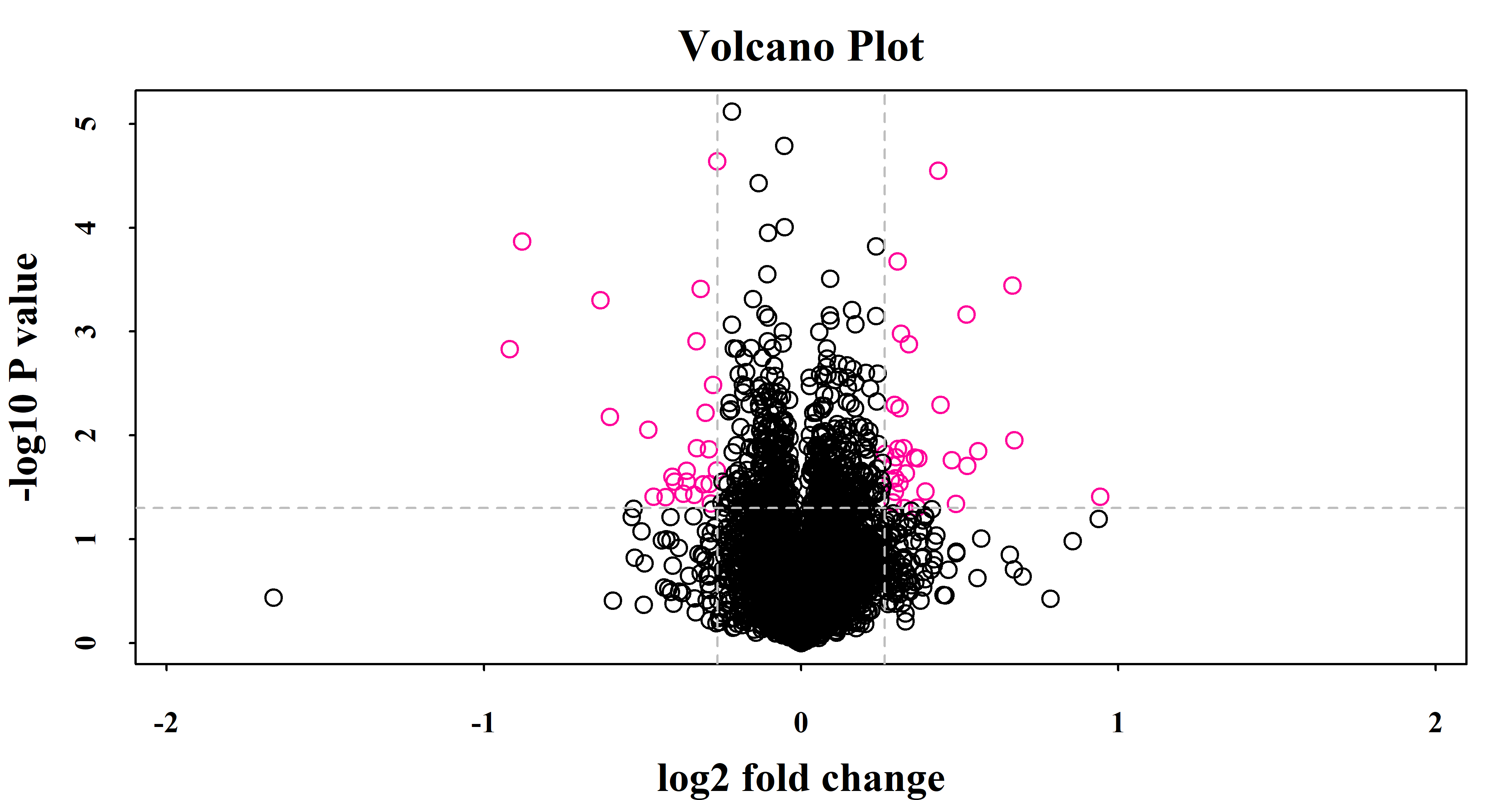

Supplement: Data S1. Data file of exported proteomics datasets, related to Figure 1 [file mmc2.zip › Date S1/2-M-GSGC0157983正式实验报告/Evaluation/图4-8 火山图.tif]

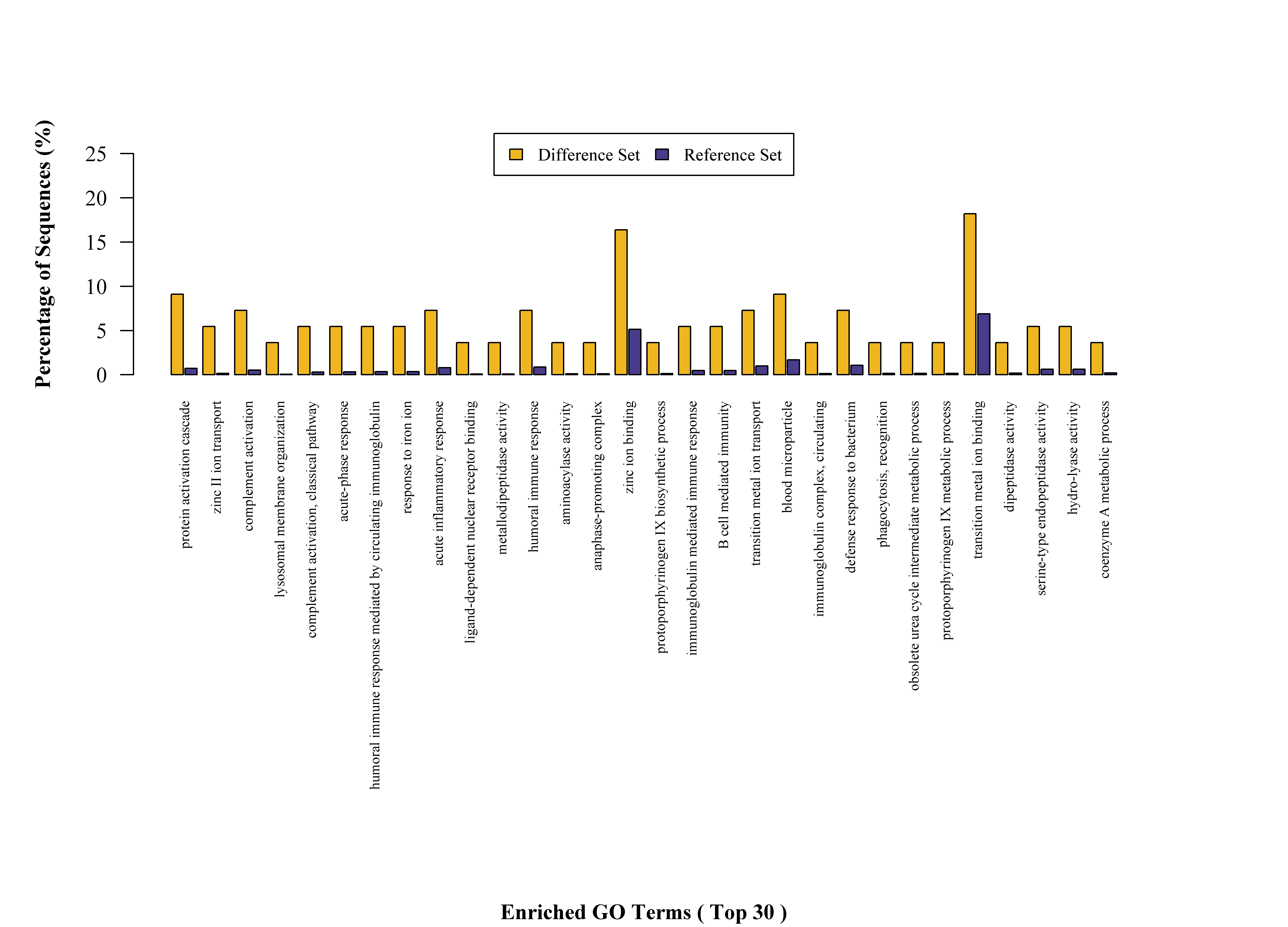

Supplement: Data S1. Data file of exported proteomics datasets, related to Figure 1 [file mmc2.zip › Date S1/2-M-GSGC0157983正式实验报告/GO分析结果文件夹/enrich_go.tiff]

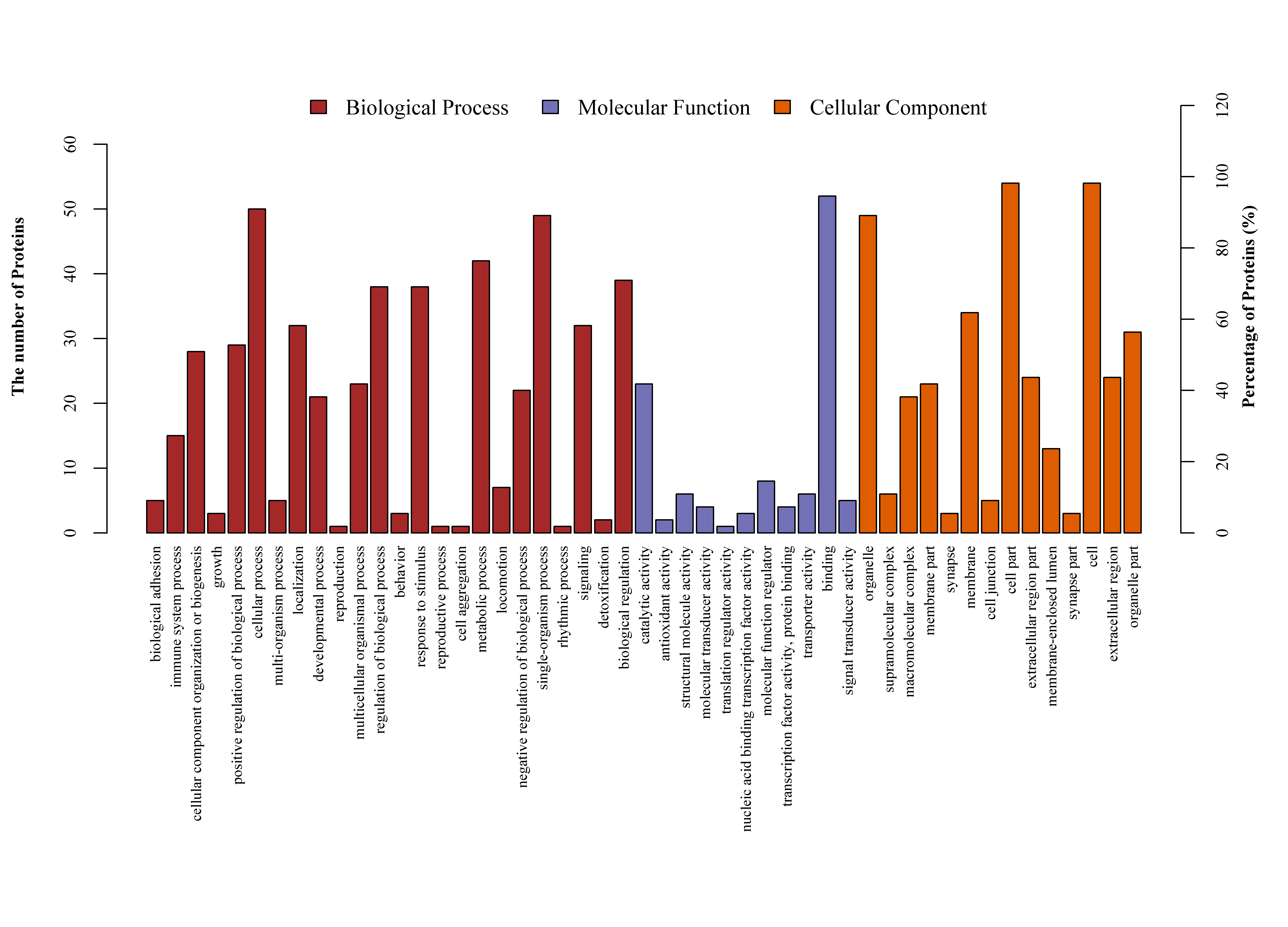

Supplement: Data S1. Data file of exported proteomics datasets, related to Figure 1 [file mmc2.zip › Date S1/2-M-GSGC0157983正式实验报告/GO分析结果文件夹/GOLevel2.tif]

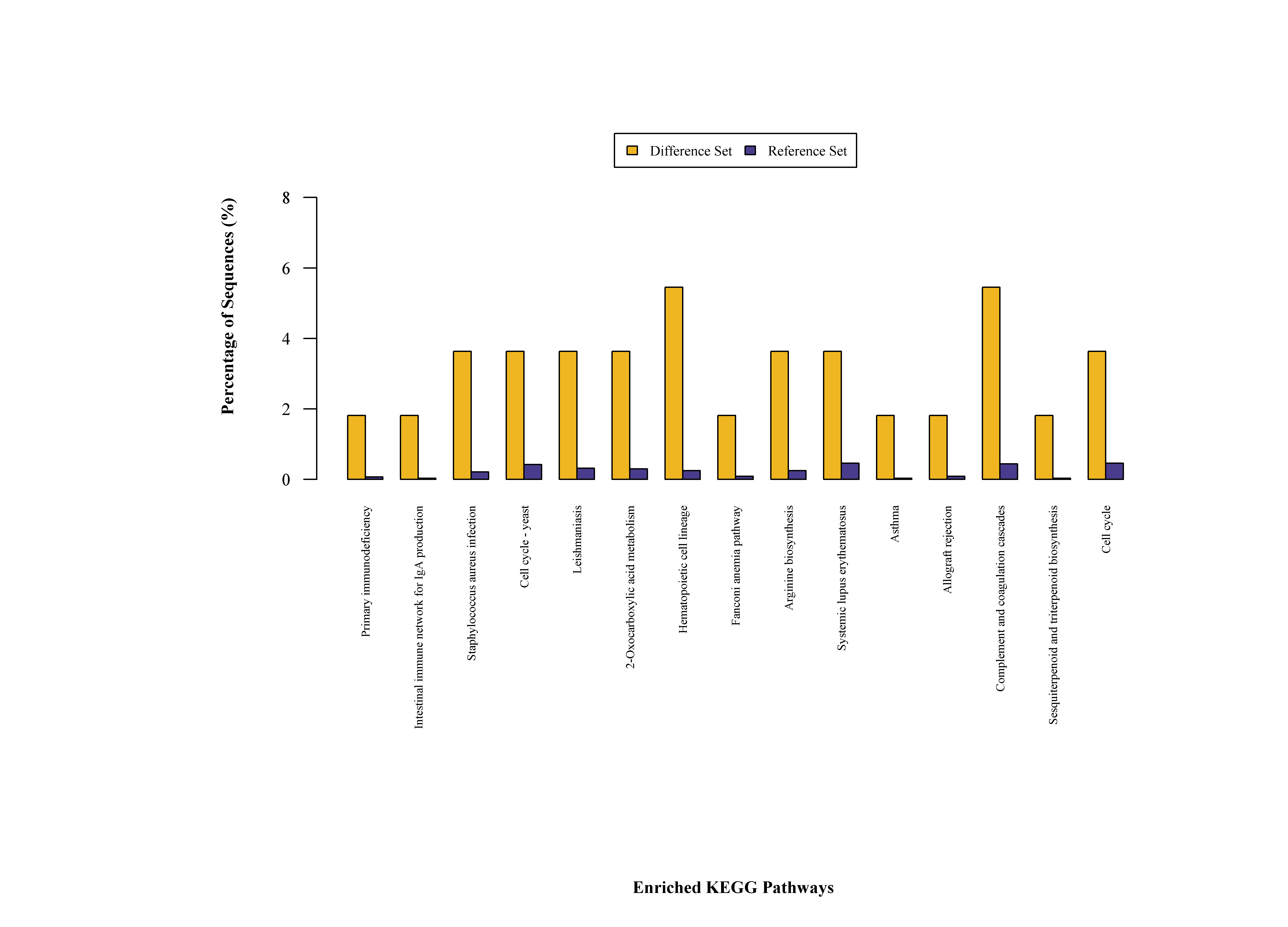

Supplement: Data S1. Data file of exported proteomics datasets, related to Figure 1 [file mmc2.zip › Date S1/2-M-GSGC0157983正式实验报告/KEGG分析结果文件夹/enrich_kegg.tiff]

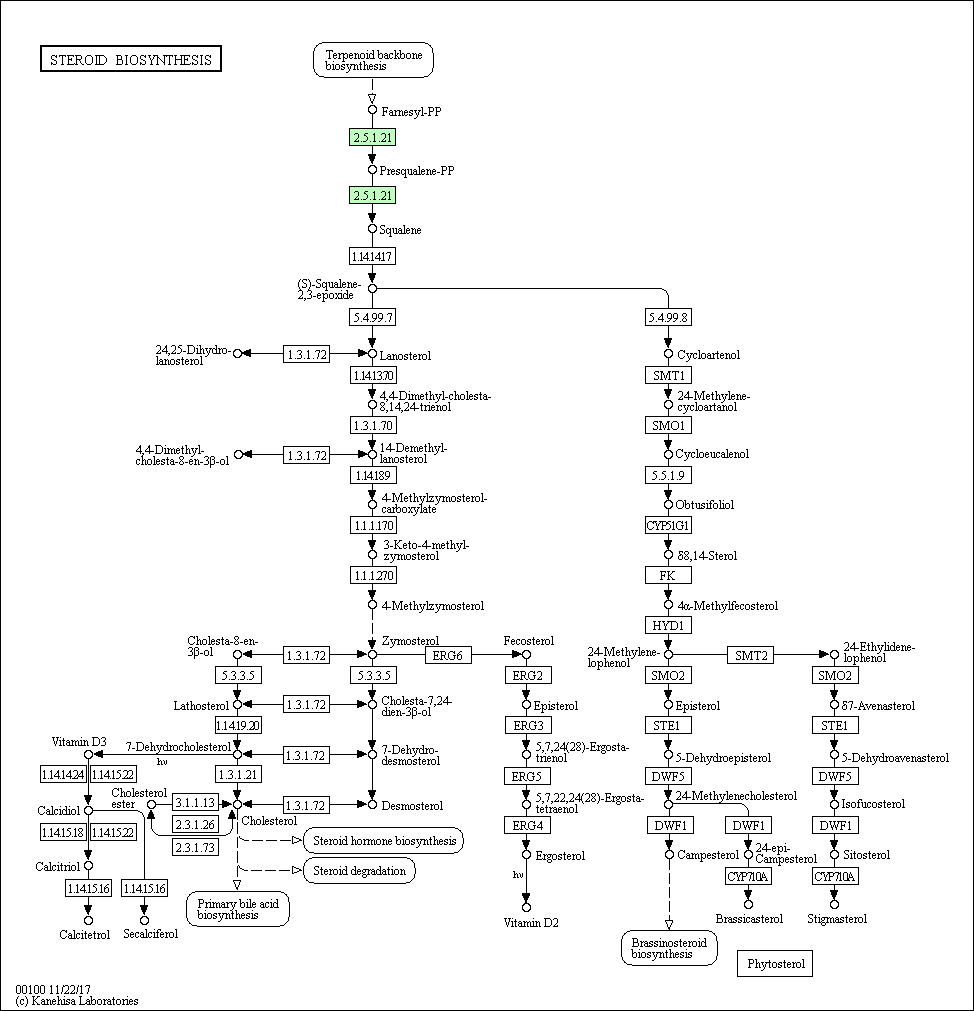

Supplement: Data S1. Data file of exported proteomics datasets, related to Figure 1 [file mmc2.zip › Date S1/2-M-GSGC0157983正式实验报告/KEGG分析结果文件夹/map/map00100.png]

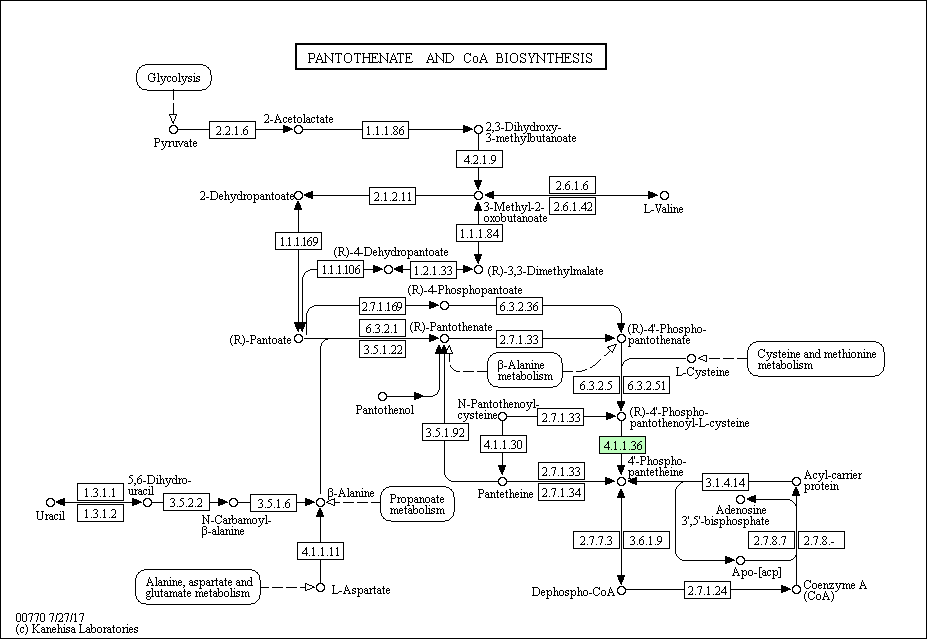

Supplement: Data S1. Data file of exported proteomics datasets, related to Figure 1 [file mmc2.zip › Date S1/2-M-GSGC0157983正式实验报告/KEGG分析结果文件夹/map/map00770.png]

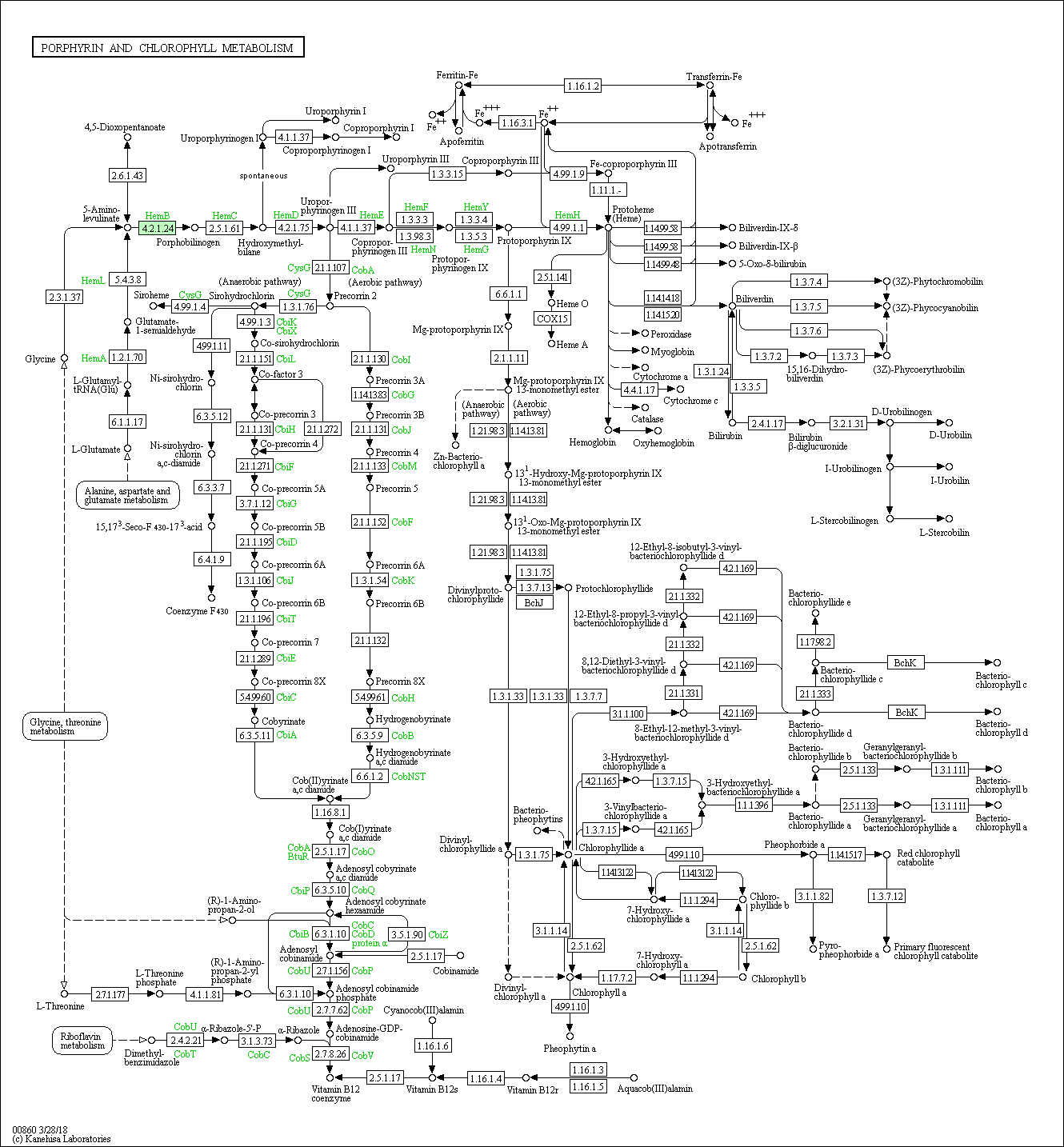

Supplement: Data S1. Data file of exported proteomics datasets, related to Figure 1 [file mmc2.zip › Date S1/2-M-GSGC0157983正式实验报告/KEGG分析结果文件夹/map/map00860.png]

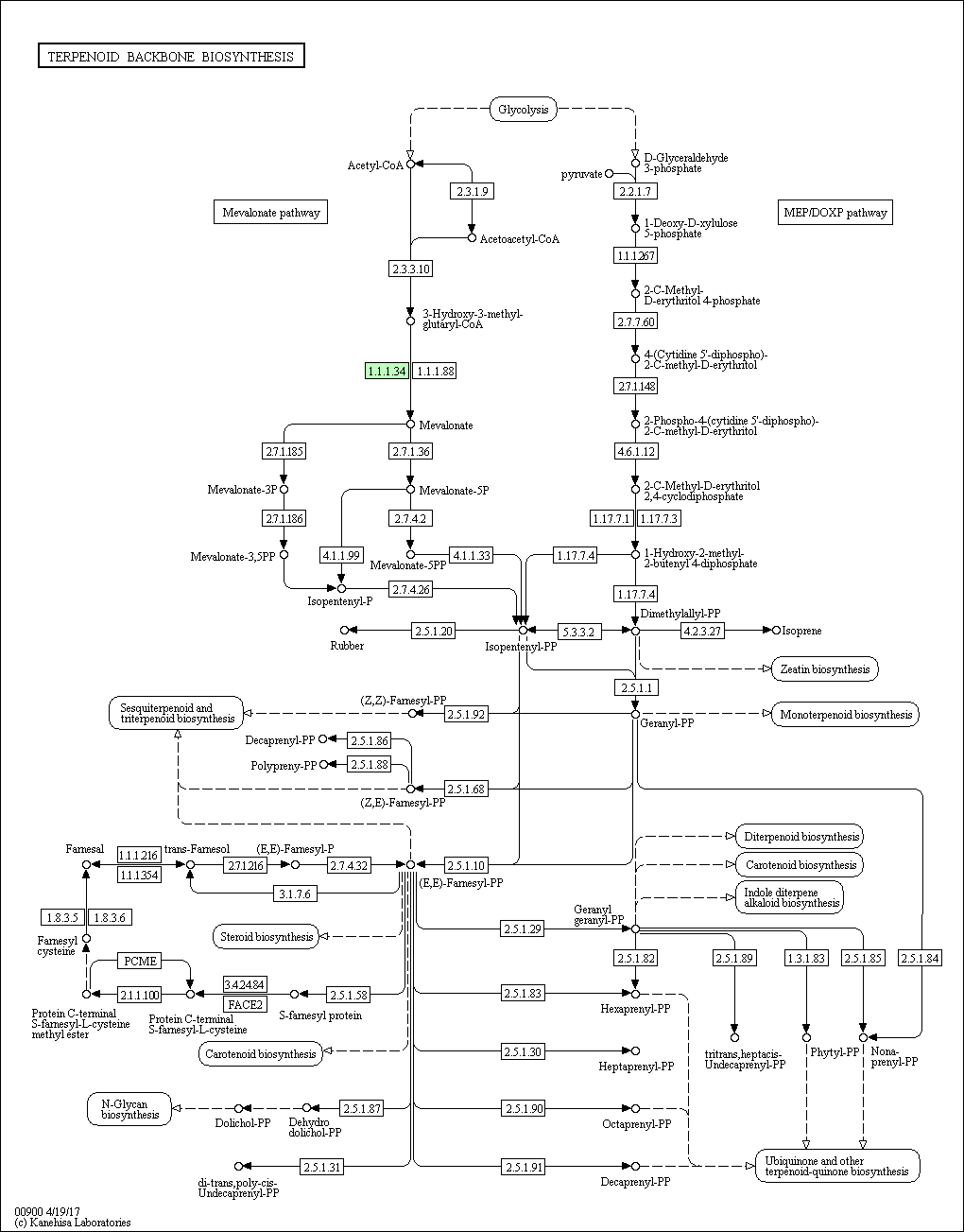

Supplement: Data S1. Data file of exported proteomics datasets, related to Figure 1 [file mmc2.zip › Date S1/2-M-GSGC0157983正式实验报告/KEGG分析结果文件夹/map/map00900.png]

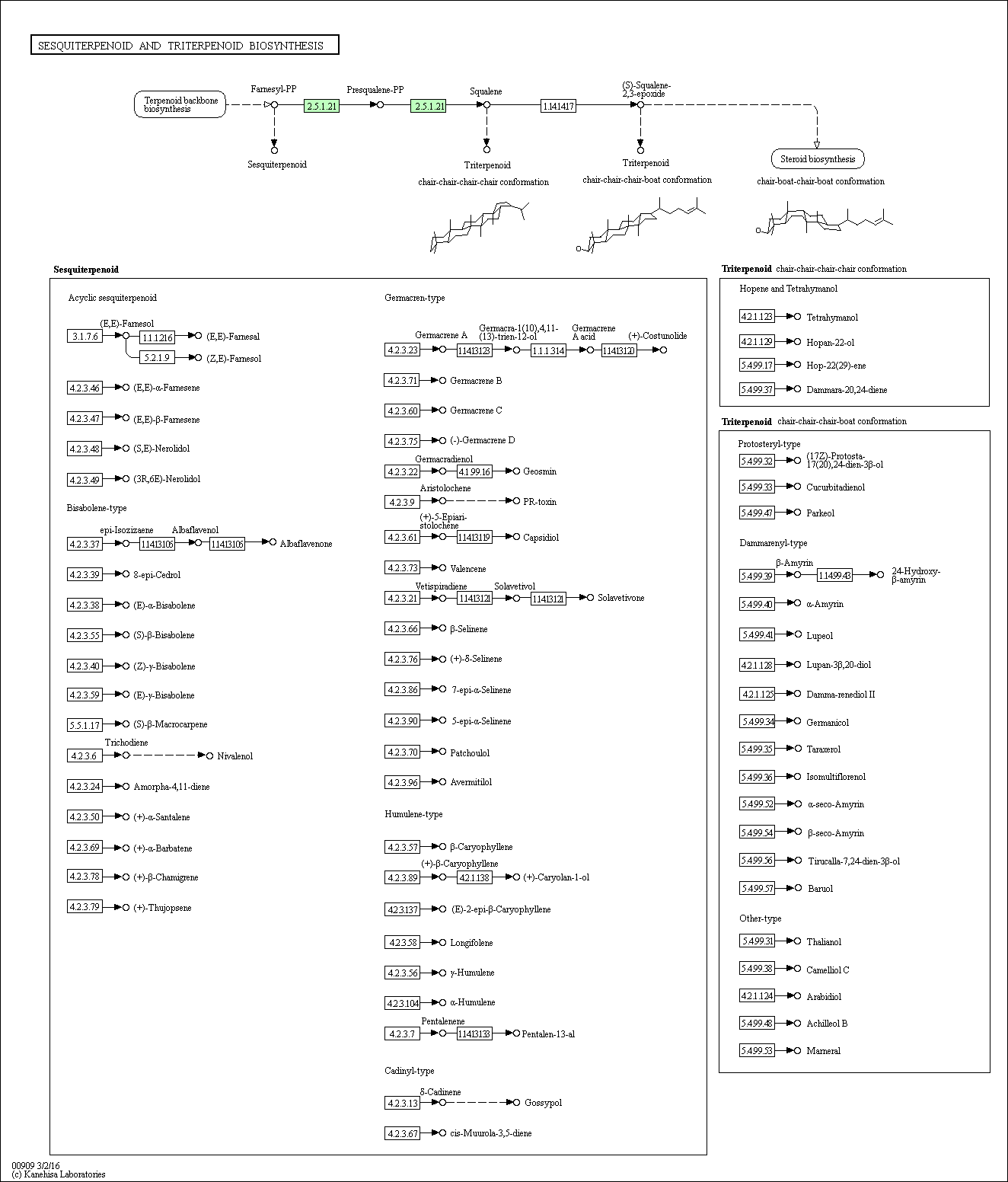

Supplement: Data S1. Data file of exported proteomics datasets, related to Figure 1 [file mmc2.zip › Date S1/2-M-GSGC0157983正式实验报告/KEGG分析结果文件夹/map/map00909.png]

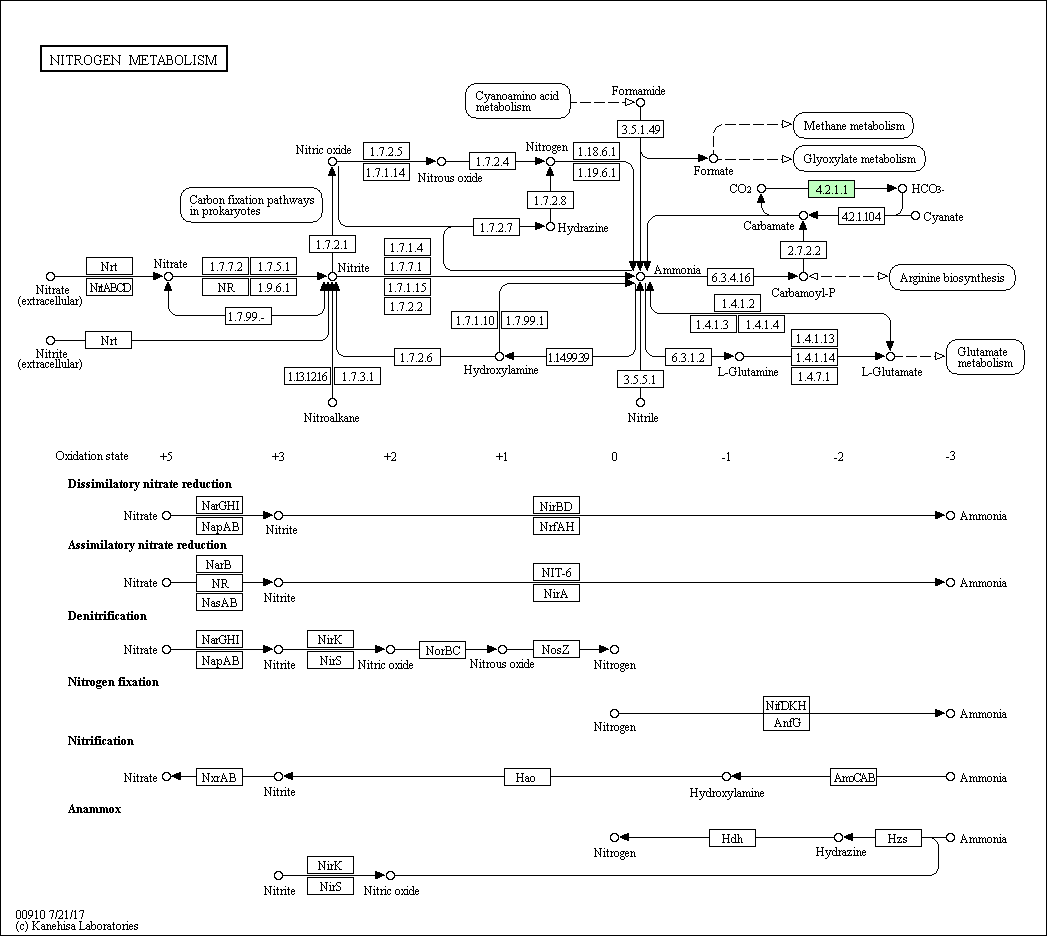

Supplement: Data S1. Data file of exported proteomics datasets, related to Figure 1 [file mmc2.zip › Date S1/2-M-GSGC0157983正式实验报告/KEGG分析结果文件夹/map/map00910.png]

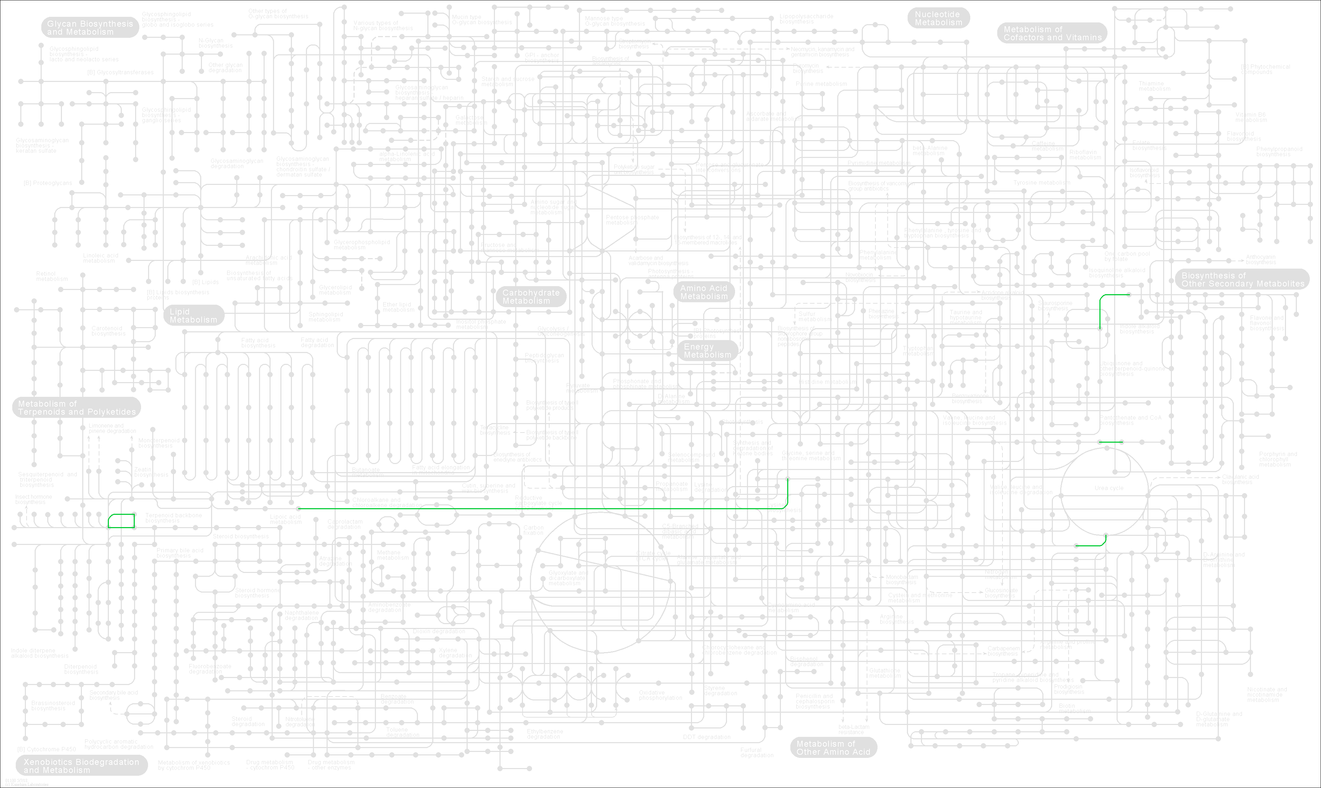

Supplement: Data S1. Data file of exported proteomics datasets, related to Figure 1 [file mmc2.zip › Date S1/2-M-GSGC0157983正式实验报告/KEGG分析结果文件夹/map/map01100_0.353765.png]

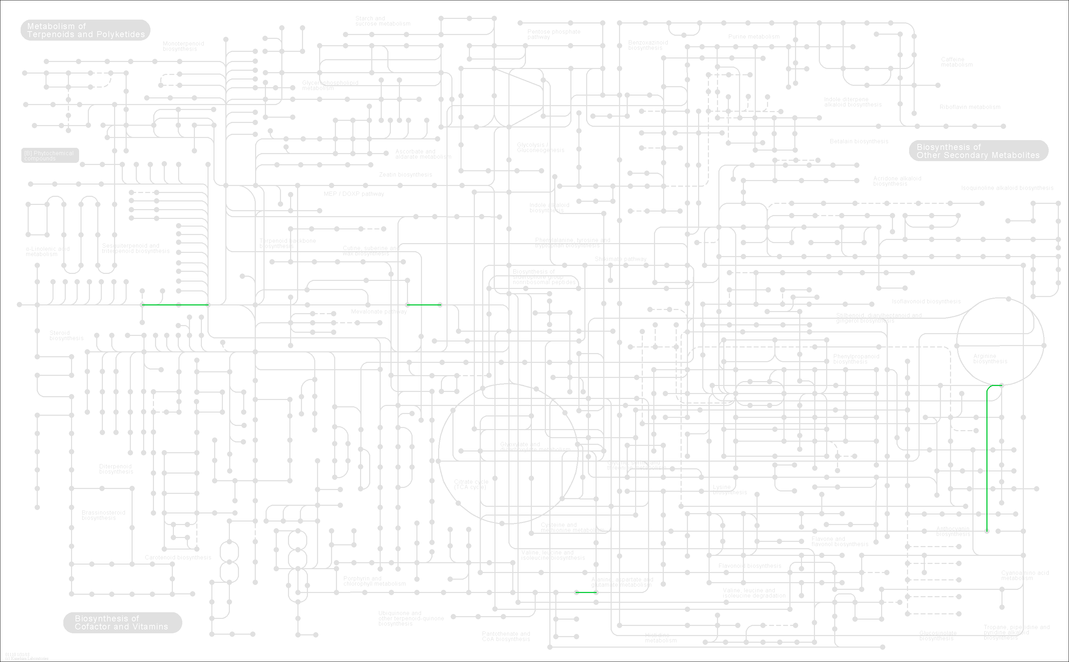

Supplement: Data S1. Data file of exported proteomics datasets, related to Figure 1 [file mmc2.zip › Date S1/2-M-GSGC0157983正式实验报告/KEGG分析结果文件夹/map/map01110_0.353770.png]

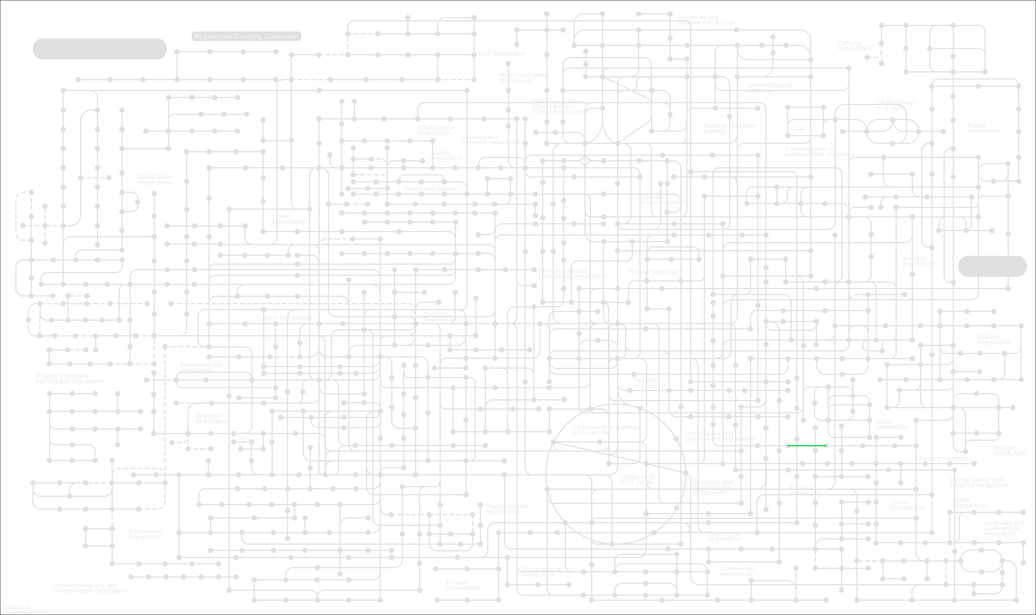

Supplement: Data S1. Data file of exported proteomics datasets, related to Figure 1 [file mmc2.zip › Date S1/2-M-GSGC0157983正式实验报告/KEGG分析结果文件夹/map/map01120_0.353771.png]

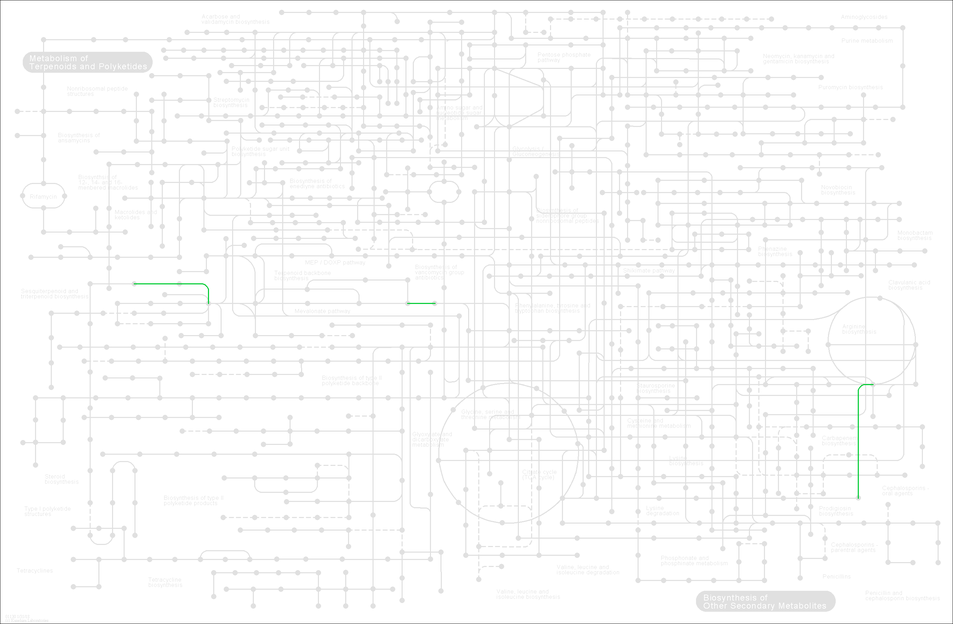

Supplement: Data S1. Data file of exported proteomics datasets, related to Figure 1 [file mmc2.zip › Date S1/2-M-GSGC0157983正式实验报告/KEGG分析结果文件夹/map/map01130_0.353778.png]

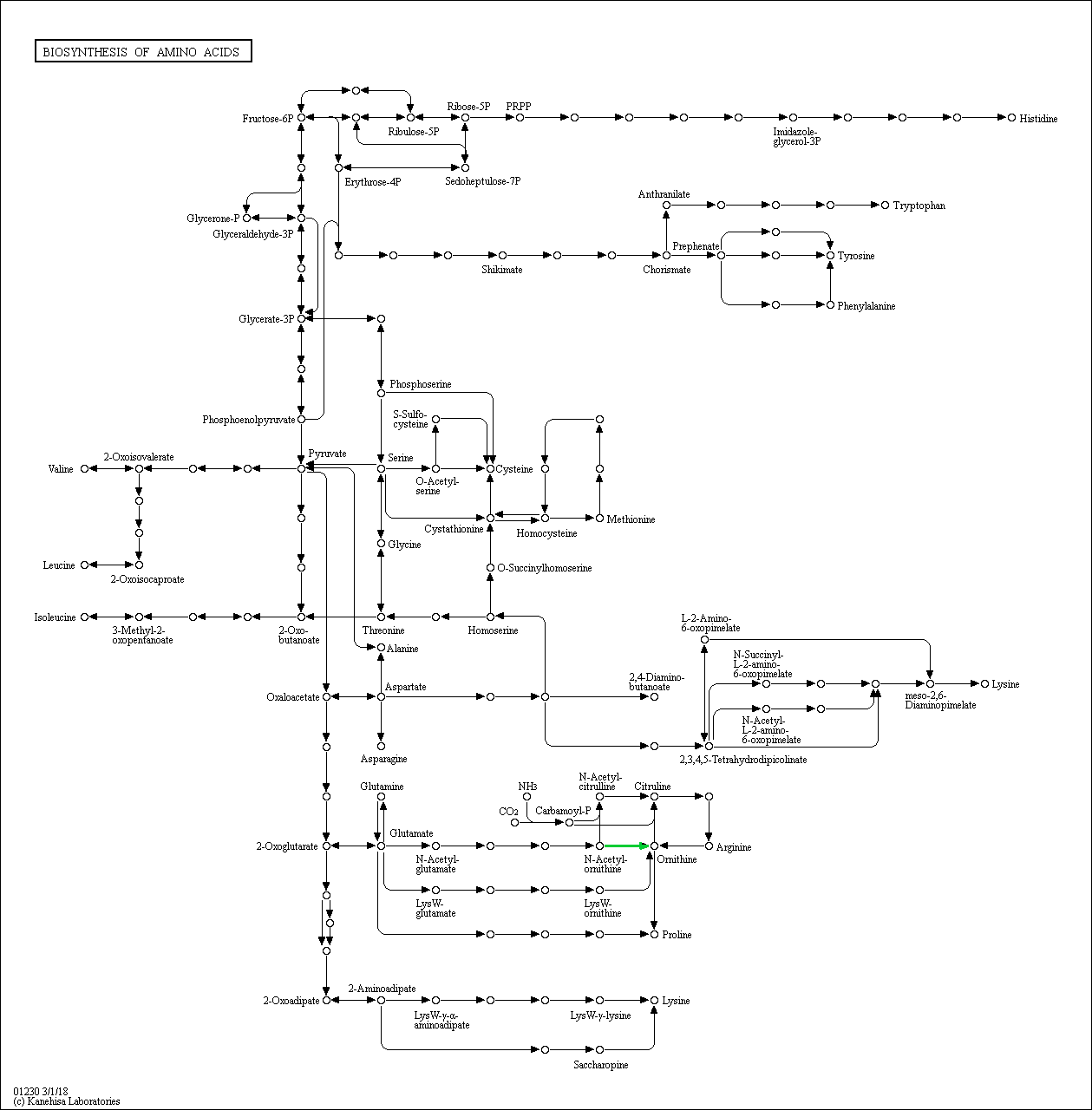

Supplement: Data S1. Data file of exported proteomics datasets, related to Figure 1 [file mmc2.zip › Date S1/2-M-GSGC0157983正式实验报告/KEGG分析结果文件夹/map/map01230.png]

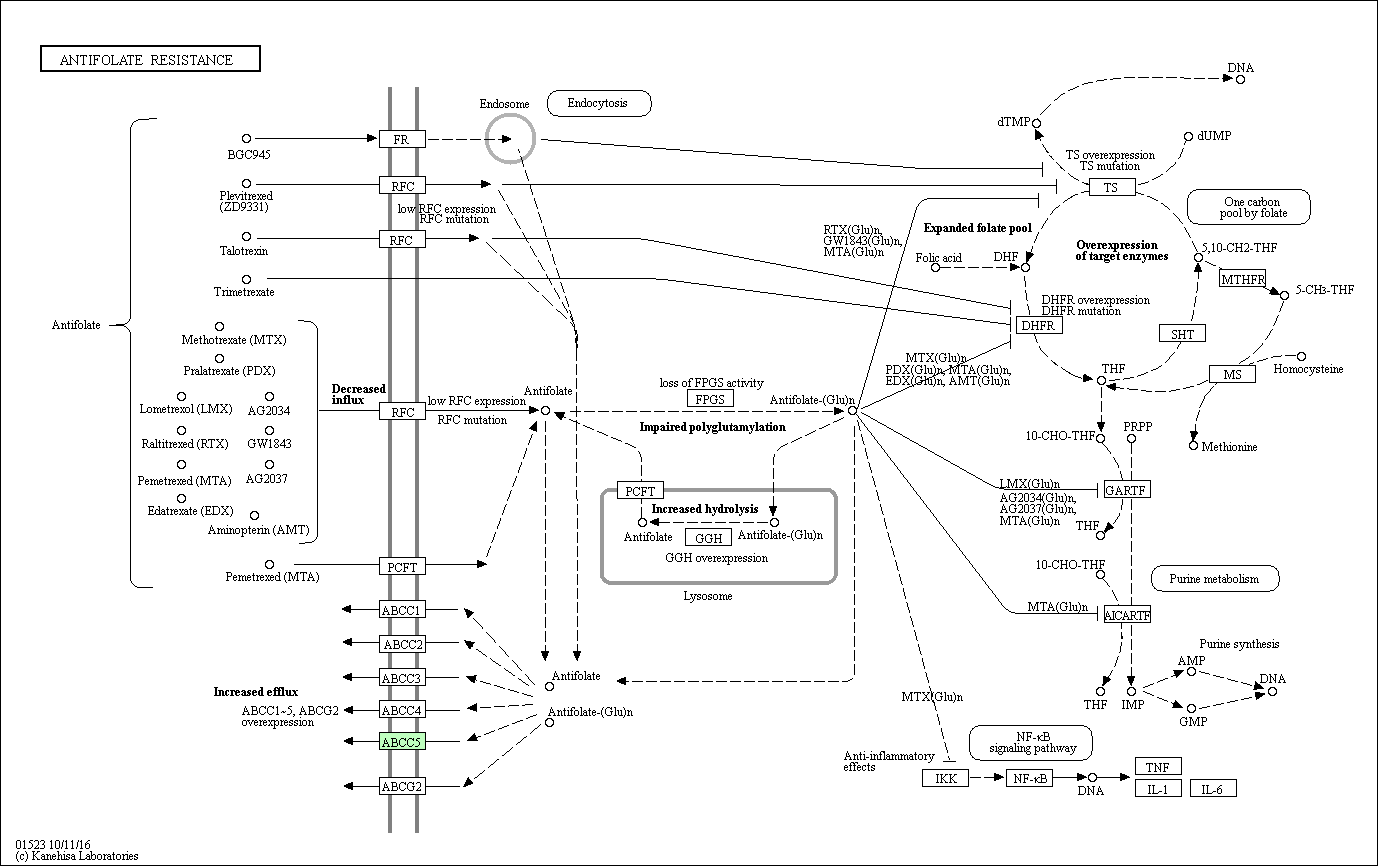

Supplement: Data S1. Data file of exported proteomics datasets, related to Figure 1 [file mmc2.zip › Date S1/2-M-GSGC0157983正式实验报告/KEGG分析结果文件夹/map/map01523.png]

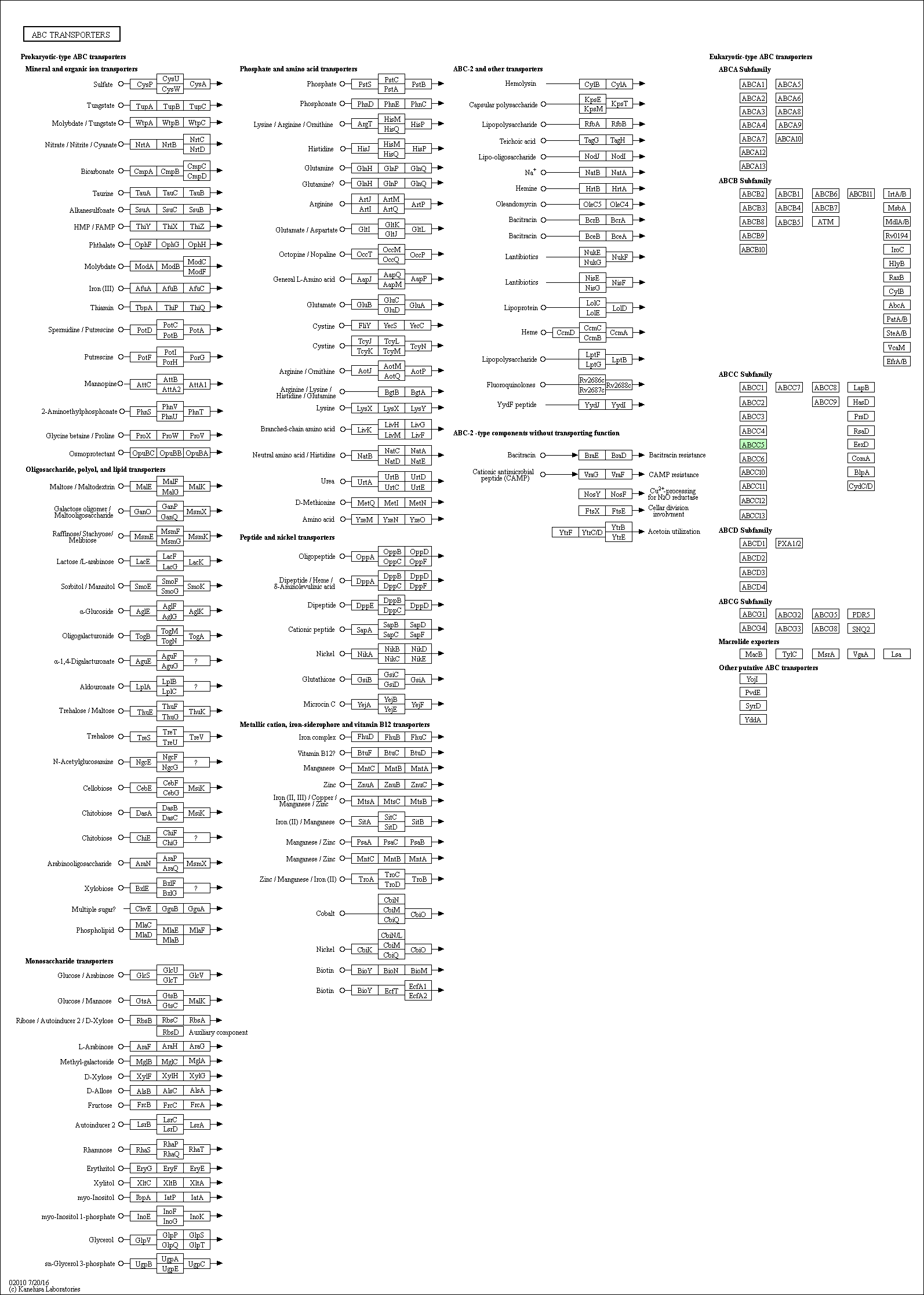

Supplement: Data S1. Data file of exported proteomics datasets, related to Figure 1 [file mmc2.zip › Date S1/2-M-GSGC0157983正式实验报告/KEGG分析结果文件夹/map/map02010.png]

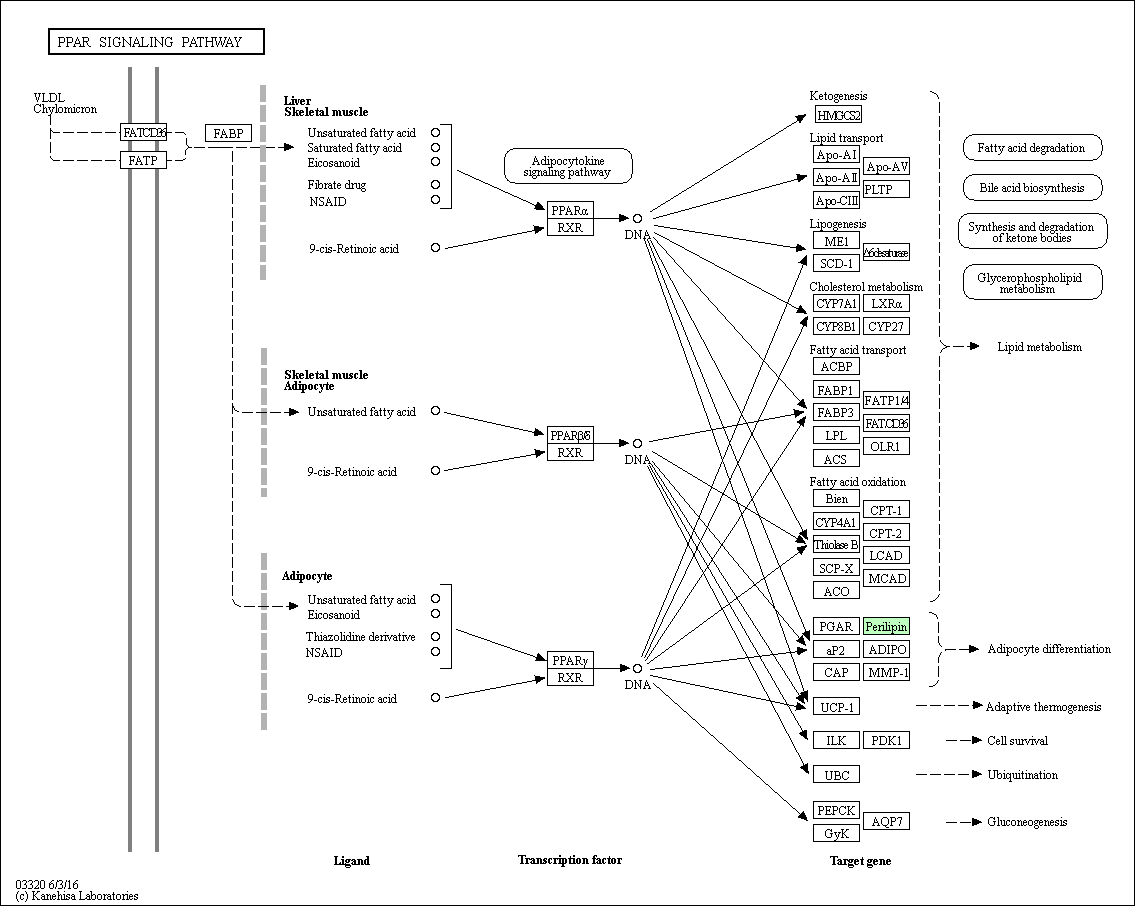

Supplement: Data S1. Data file of exported proteomics datasets, related to Figure 1 [file mmc2.zip › Date S1/2-M-GSGC0157983正式实验报告/KEGG分析结果文件夹/map/map03320.png]

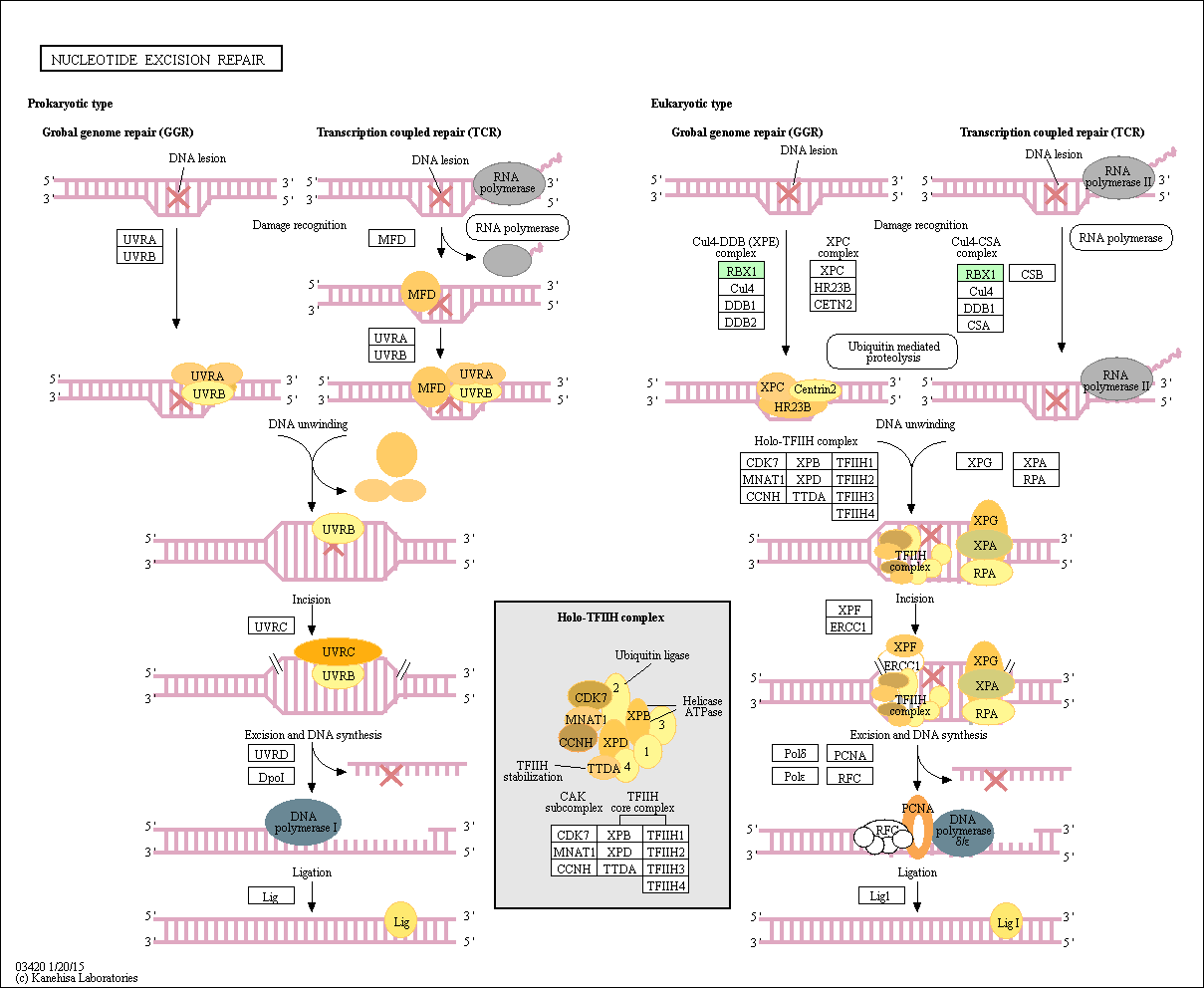

Supplement: Data S1. Data file of exported proteomics datasets, related to Figure 1 [file mmc2.zip › Date S1/2-M-GSGC0157983正式实验报告/KEGG分析结果文件夹/map/map03420.png]

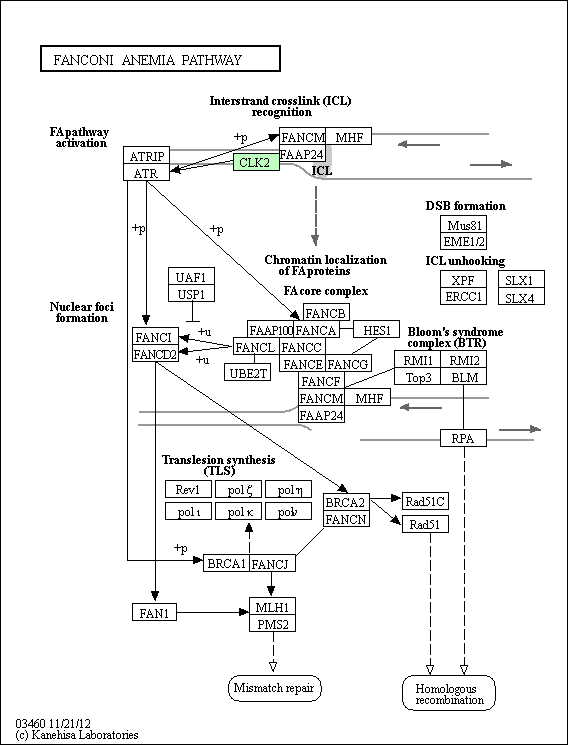

Supplement: Data S1. Data file of exported proteomics datasets, related to Figure 1 [file mmc2.zip › Date S1/2-M-GSGC0157983正式实验报告/KEGG分析结果文件夹/map/map03460.png]

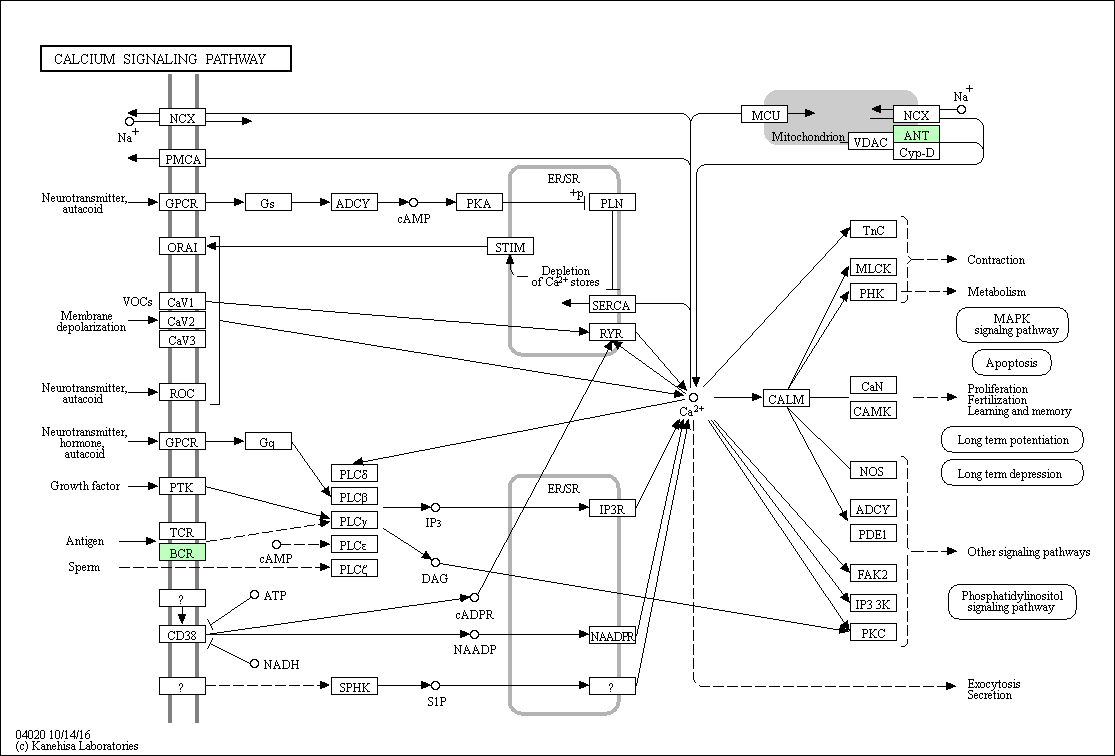

Supplement: Data S1. Data file of exported proteomics datasets, related to Figure 1 [file mmc2.zip › Date S1/2-M-GSGC0157983正式实验报告/KEGG分析结果文件夹/map/map04020.png]

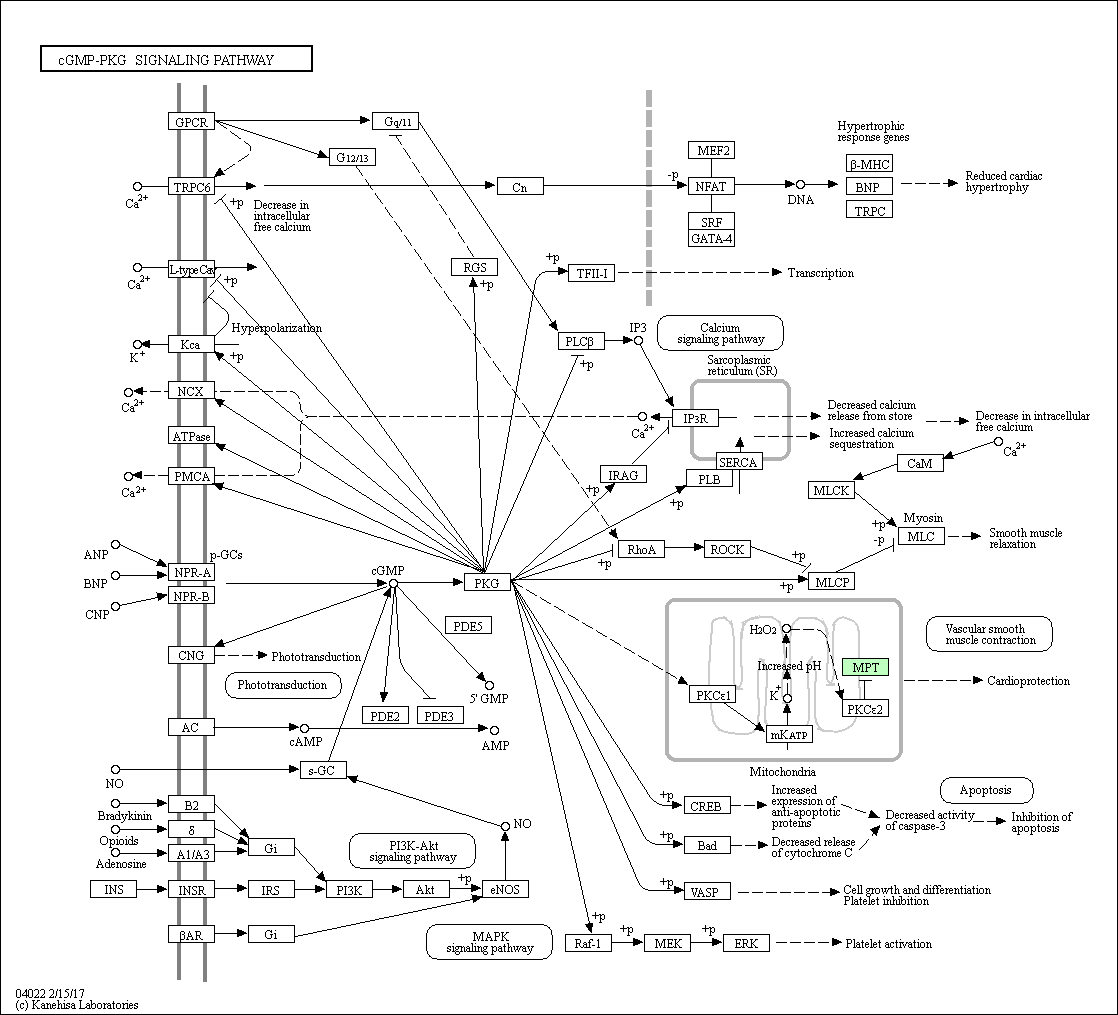

Supplement: Data S1. Data file of exported proteomics datasets, related to Figure 1 [file mmc2.zip › Date S1/2-M-GSGC0157983正式实验报告/KEGG分析结果文件夹/map/map04022.png]

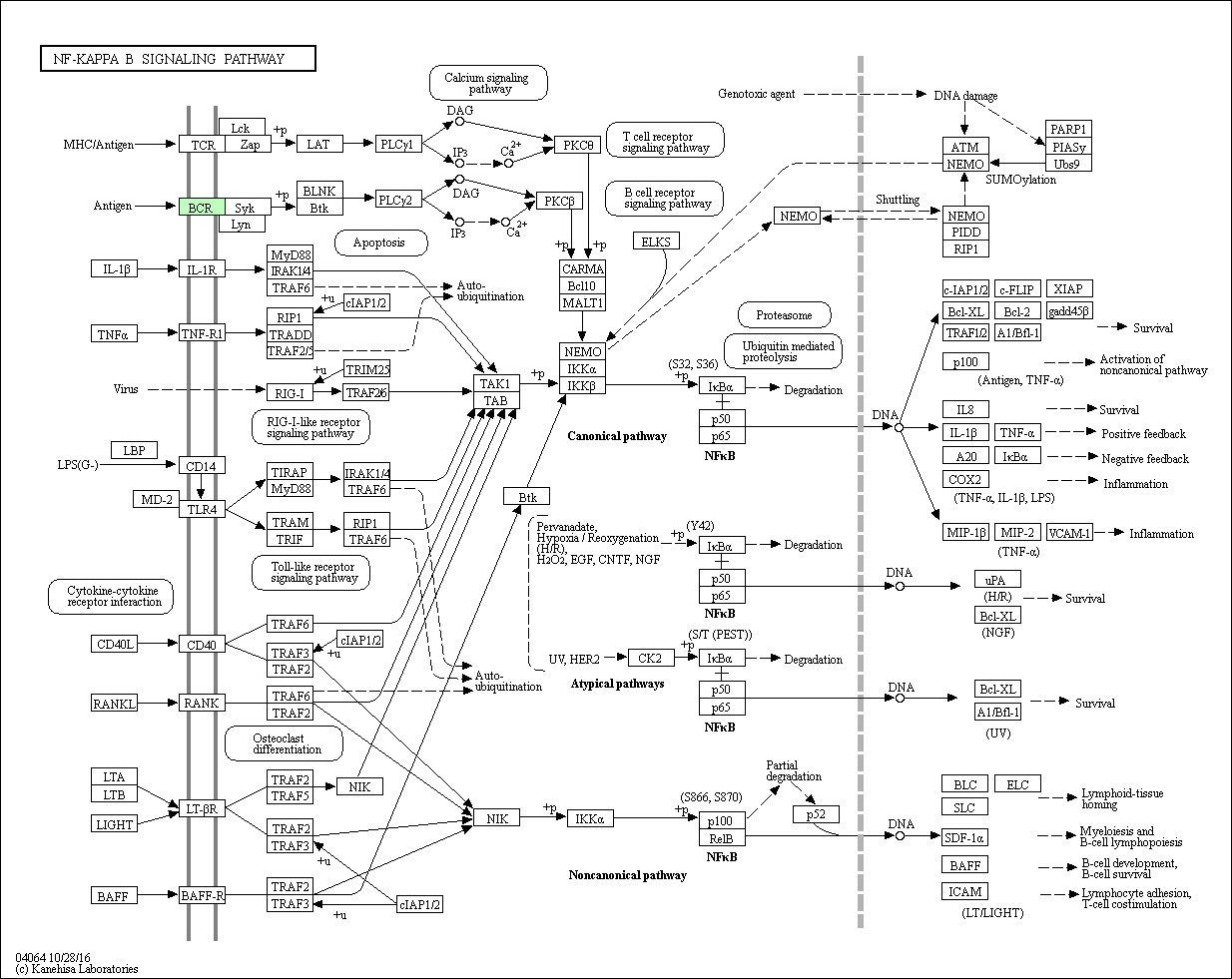

Supplement: Data S1. Data file of exported proteomics datasets, related to Figure 1 [file mmc2.zip › Date S1/2-M-GSGC0157983正式实验报告/KEGG分析结果文件夹/map/map04064.png]

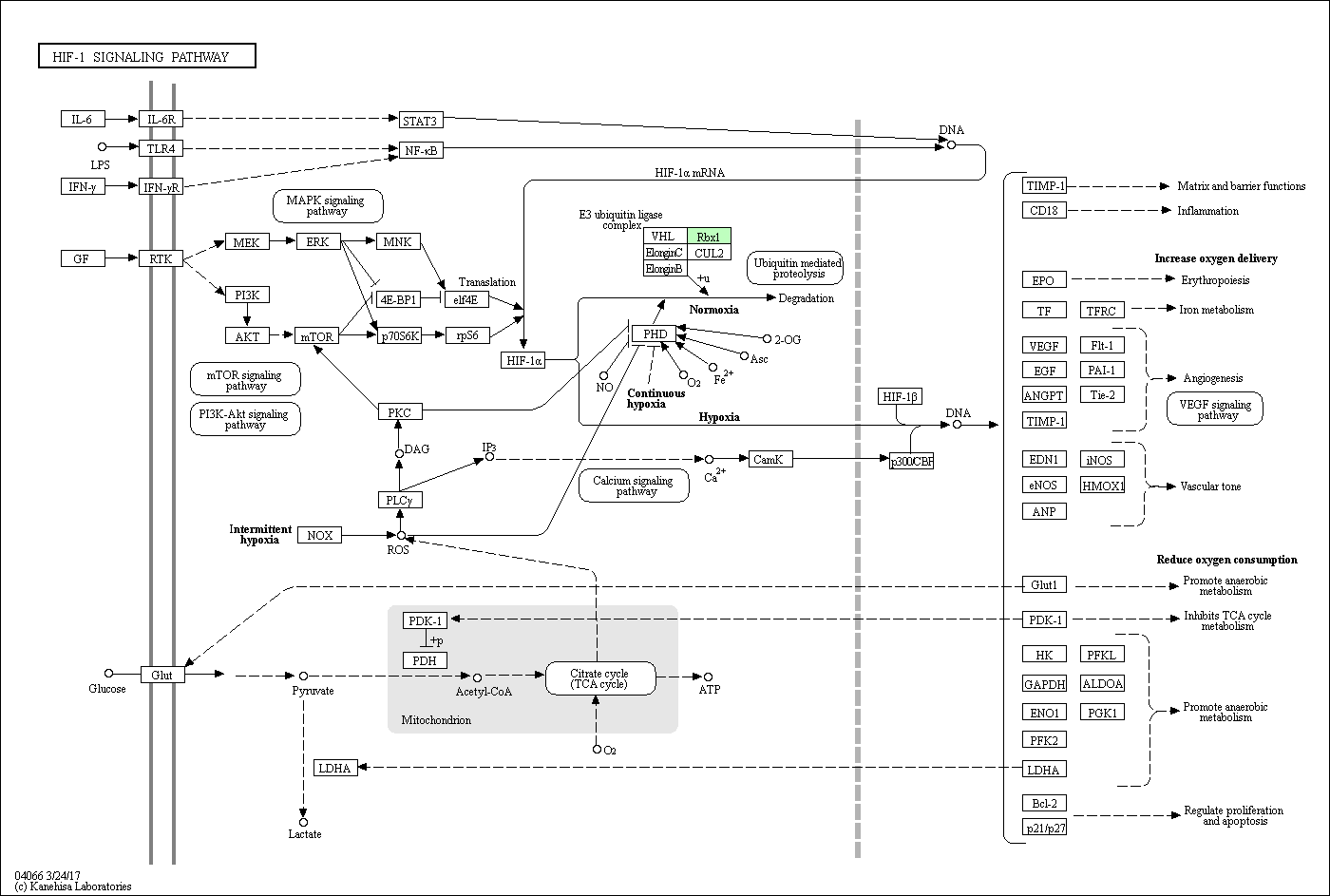

Supplement: Data S1. Data file of exported proteomics datasets, related to Figure 1 [file mmc2.zip › Date S1/2-M-GSGC0157983正式实验报告/KEGG分析结果文件夹/map/map04066.png]

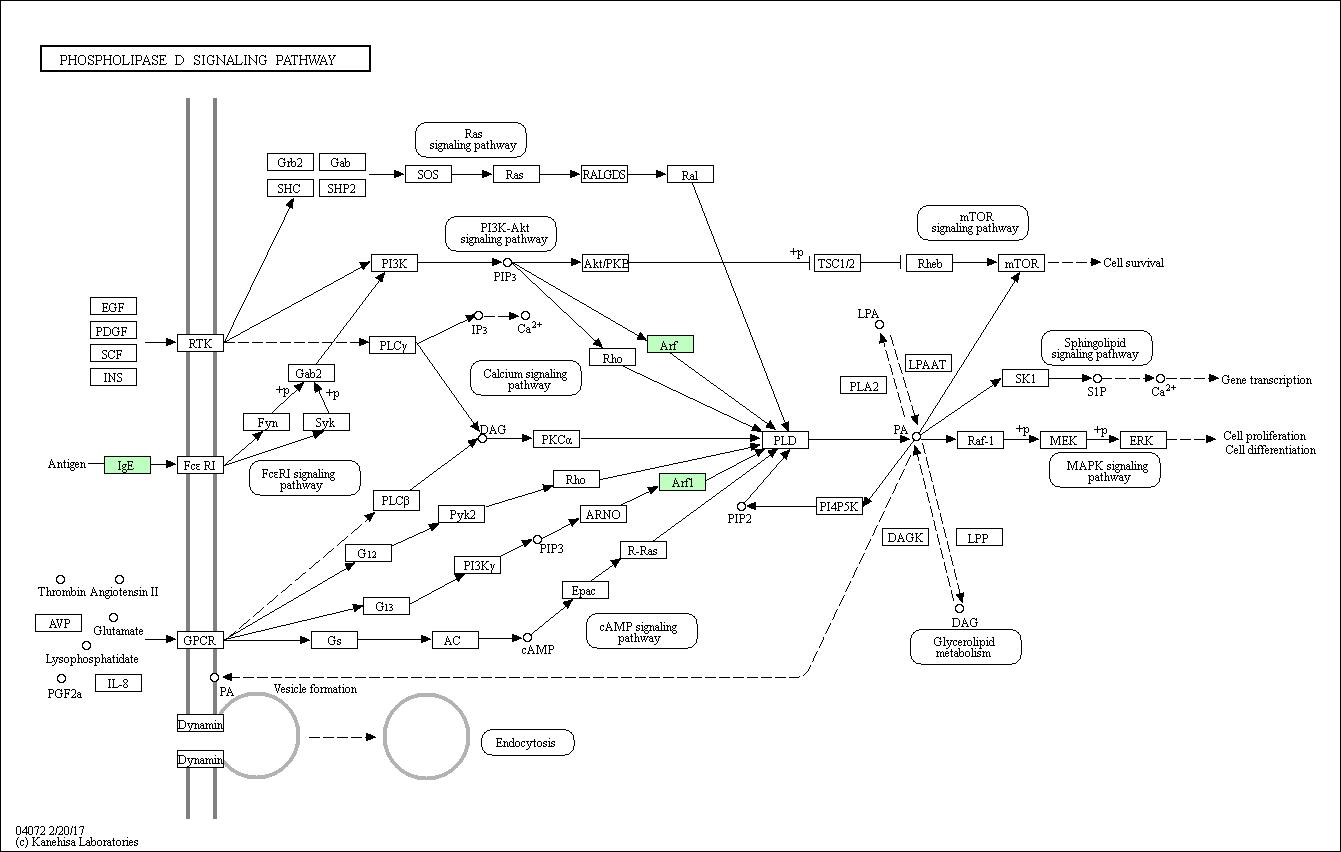

Supplement: Data S1. Data file of exported proteomics datasets, related to Figure 1 [file mmc2.zip › Date S1/2-M-GSGC0157983正式实验报告/KEGG分析结果文件夹/map/map04072.png]

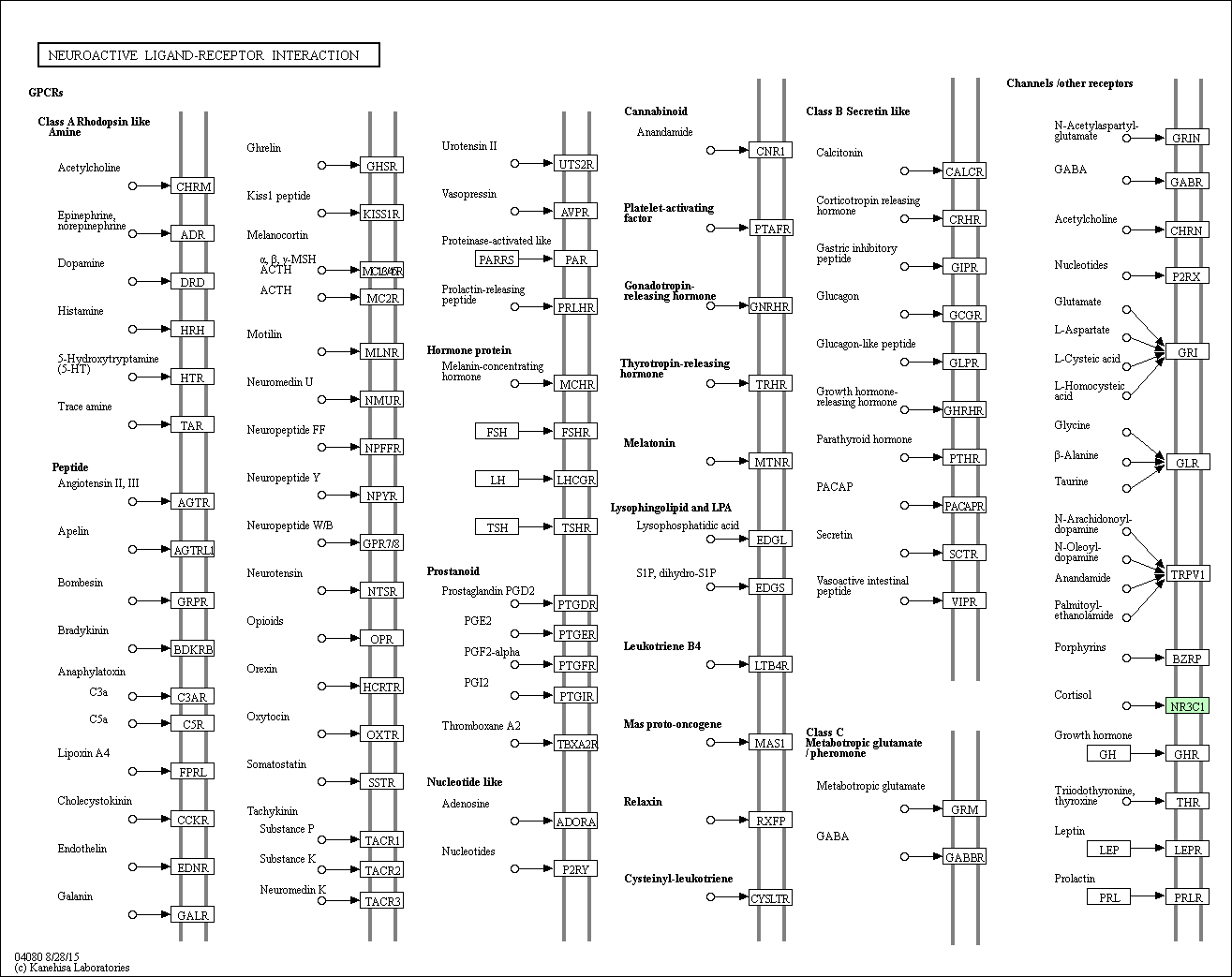

Supplement: Data S1. Data file of exported proteomics datasets, related to Figure 1 [file mmc2.zip › Date S1/2-M-GSGC0157983正式实验报告/KEGG分析结果文件夹/map/map04080.png]

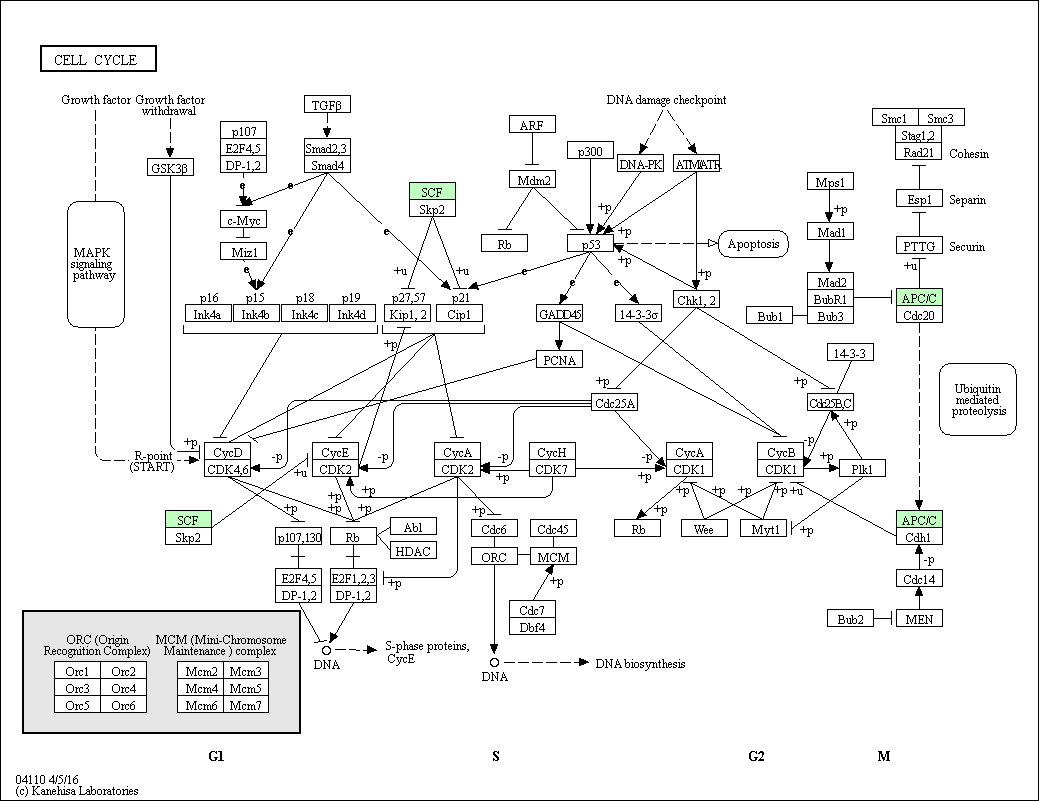

Supplement: Data S1. Data file of exported proteomics datasets, related to Figure 1 [file mmc2.zip › Date S1/2-M-GSGC0157983正式实验报告/KEGG分析结果文件夹/map/map04110.png]

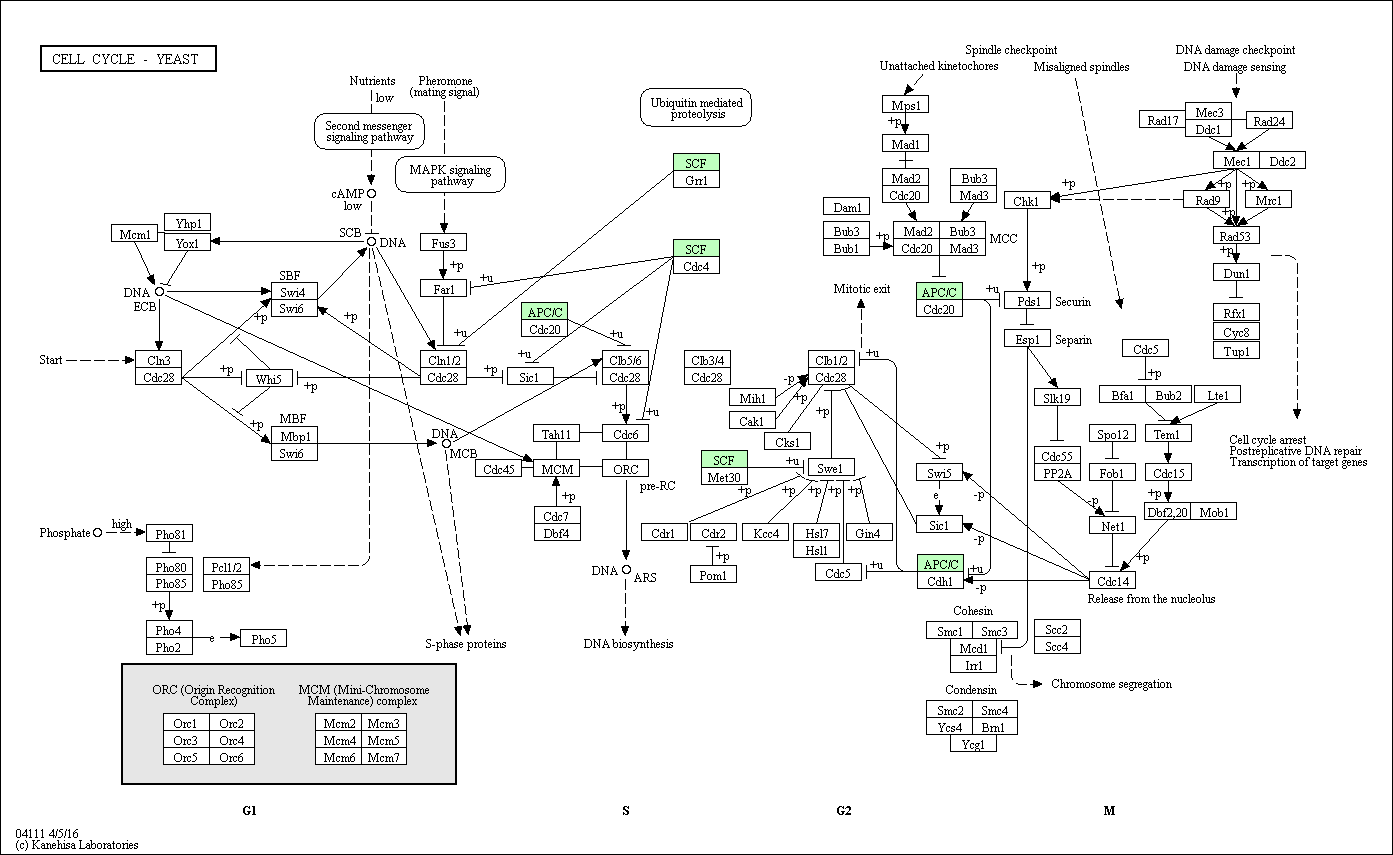

Supplement: Data S1. Data file of exported proteomics datasets, related to Figure 1 [file mmc2.zip › Date S1/2-M-GSGC0157983正式实验报告/KEGG分析结果文件夹/map/map04111.png]

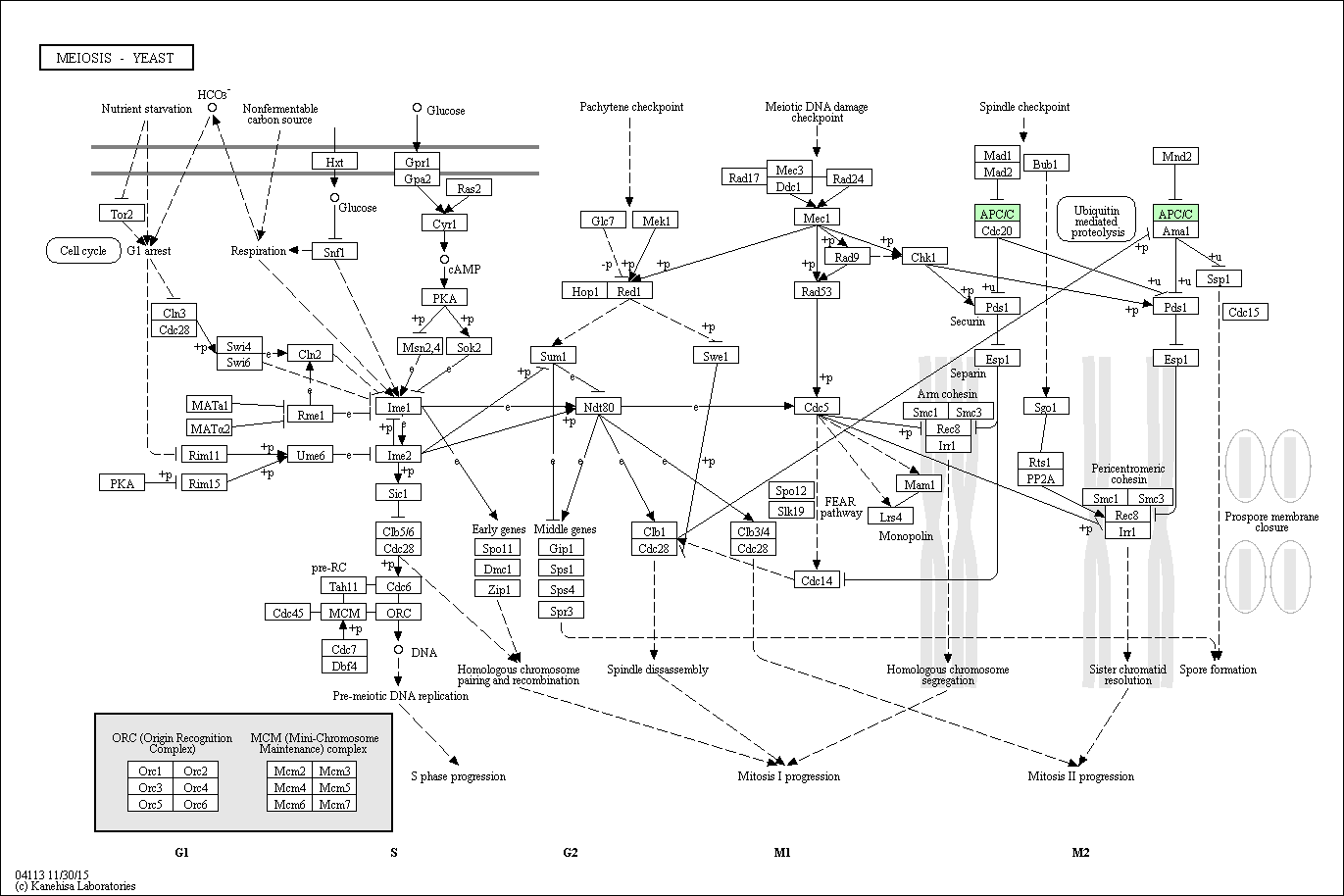

Supplement: Data S1. Data file of exported proteomics datasets, related to Figure 1 [file mmc2.zip › Date S1/2-M-GSGC0157983正式实验报告/KEGG分析结果文件夹/map/map04113.png]

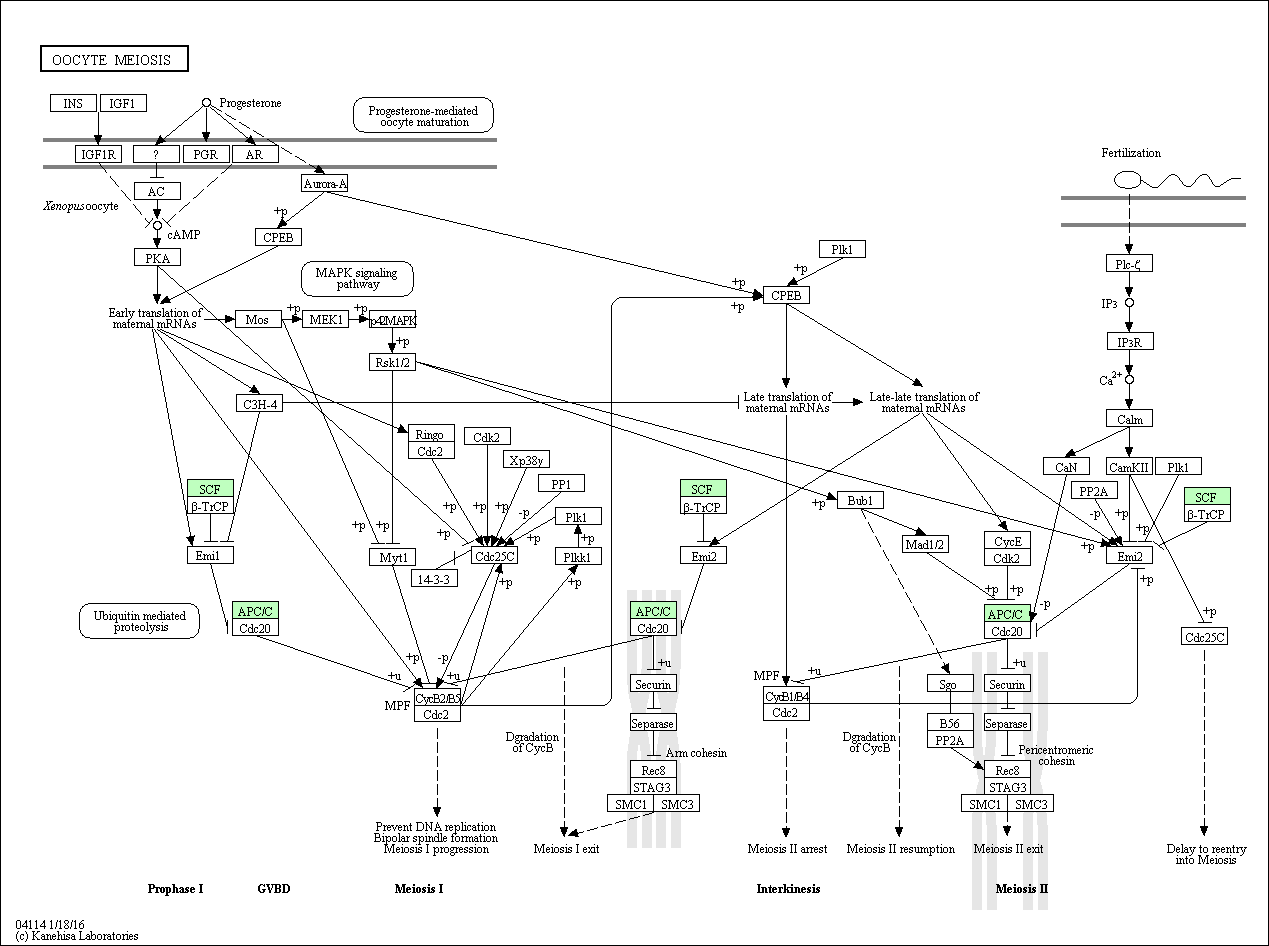

Supplement: Data S1. Data file of exported proteomics datasets, related to Figure 1 [file mmc2.zip › Date S1/2-M-GSGC0157983正式实验报告/KEGG分析结果文件夹/map/map04114.png]

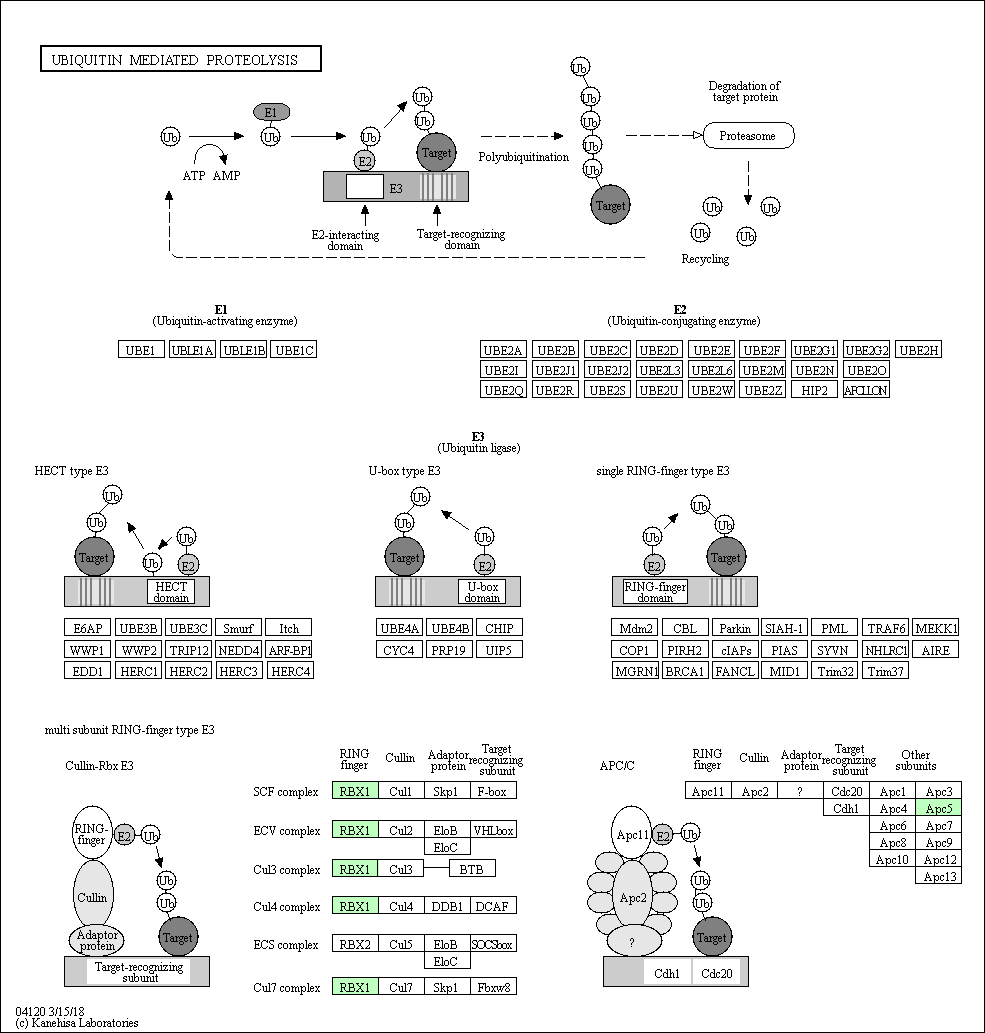

Supplement: Data S1. Data file of exported proteomics datasets, related to Figure 1 [file mmc2.zip › Date S1/2-M-GSGC0157983正式实验报告/KEGG分析结果文件夹/map/map04120.png]

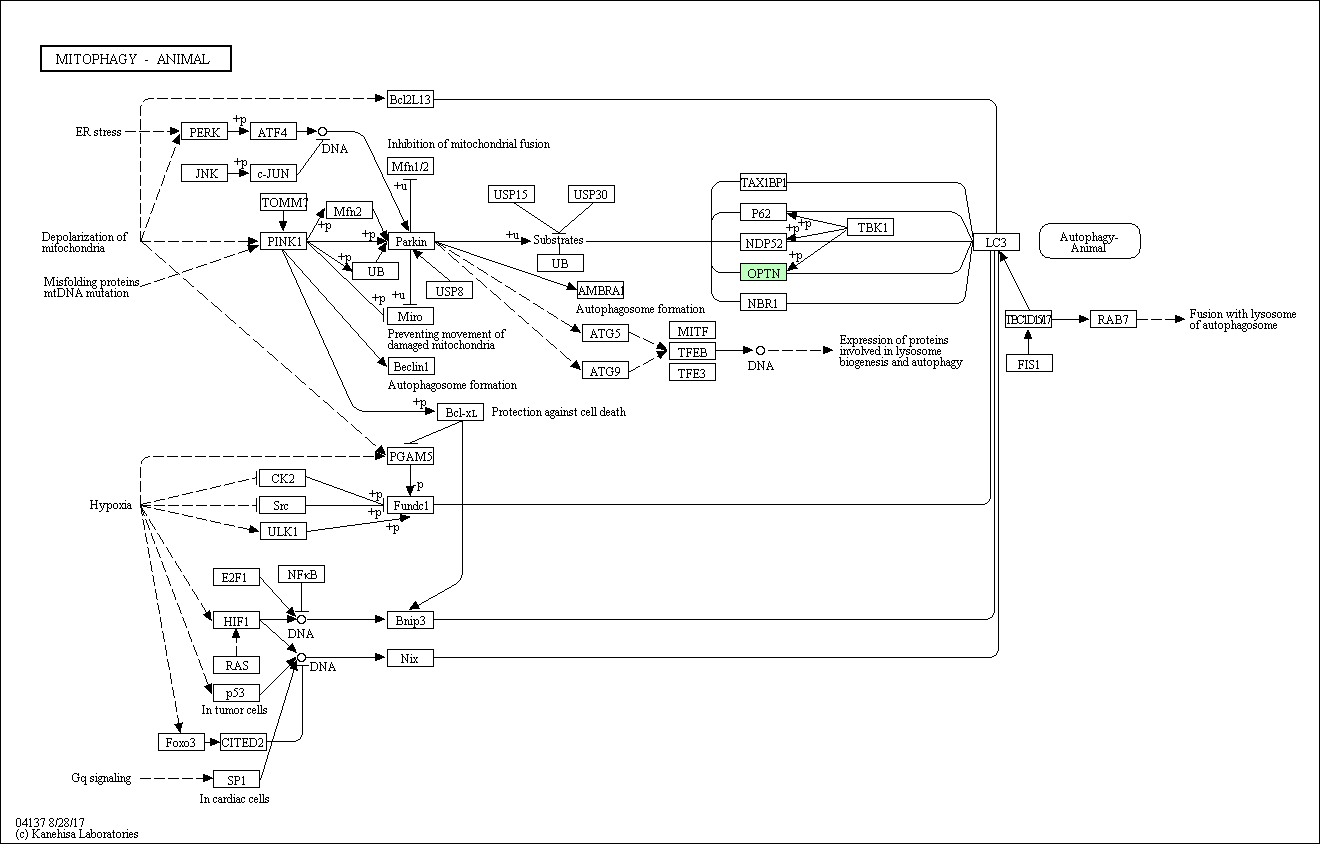

Supplement: Data S1. Data file of exported proteomics datasets, related to Figure 1 [file mmc2.zip › Date S1/2-M-GSGC0157983正式实验报告/KEGG分析结果文件夹/map/map04137.png]

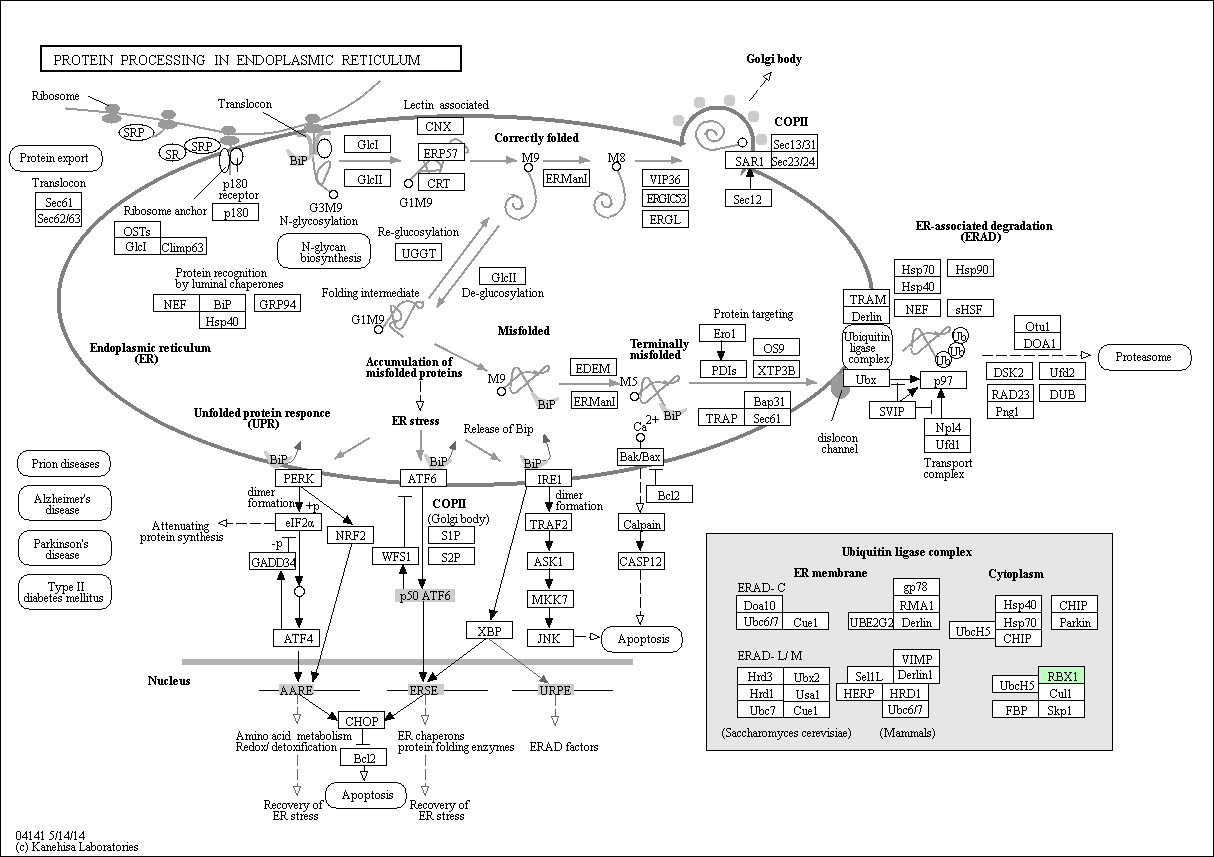

Supplement: Data S1. Data file of exported proteomics datasets, related to Figure 1 [file mmc2.zip › Date S1/2-M-GSGC0157983正式实验报告/KEGG分析结果文件夹/map/map04141.png]
