# Supplementary material for: A novel co-target of ACY1 governing plasma membrane translocation of SphK1 contributes to inflammatory and neuropathic pain
Source: iScience. 2023 May 28;26(6):106989. doi: 10.1016/j.isci.2023.106989 (PMC10291574; doi:10.1016/j.isci.2023.106989)
Supplement: Data S1. Data file of exported proteomics datasets, related to Figure 1 [file mmc2.zip › Date S1/2-M-GSGC0157983正式实验报告/预实验报告/M-GSGC0157983预实验报告.docx]

| **蛋白质样本质检结果和预备实验报告** | | | | | | | | | | | | | | | | | | | | | | | | | | | | | | | | | | | | | | | | |
| --- | --- | --- | --- | --- | --- | --- | --- | --- | --- | --- | --- | --- | --- | --- | --- | --- | --- | --- | --- | --- | --- | --- | --- | --- | --- | --- | --- | --- | --- | --- | --- | --- | --- | --- | --- | --- | --- | --- | --- | --- |
| **项目信息** | | | | | | | | | | | | | | | | | | | | | | | | | | | | | | | | | | | | | | | | |
| **合同编号** | | | | | | M-GSGC0157983 | | | | | | | | | | | | | | | | **样本类型** | | | | | | 小鼠背根神经节 | | | | | | | | | | | | |
| **样本数量** | | | | | | 6 | | | | | | | | | | | | | | | | **报告日期** | | | | | | 2018/4/27 | | | | | | | | | | | | |
| **检测方法** | | | | | | | | | | | | | | | | | | | | | | | | | | | | | | | | | | | | | | | | |
| **抽提类型** | | | | | |  | | | | | | | | | | | |  | | | | | | | | | | | | | | | | | | | | | | |
| **抽提方法** | | | | | | 匀浆+SDT裂解 | | | | | | | | | | | | | | | | | | | | | | | | | | | | | | | | | | |
| **质检方法** | | | | | | SDS-PAGE电泳  LC-MS/MS（仪器：QE;软件：PD/MASCOT） | | | | | | | | | | | | | | | | | | | | | | | | | | | | | | | | | | |
| **检测结果** | | | | | | | | | | | | | | | | | | | | | | | | | | | | | | | | | | | | | | | | |
| **编号** | | | 1 | | | 2 | 3 | | | | 4 | | | | 5 | | | | 6 | | | | 7 | | | | 8 | | | | 9 | | | 10 | | | 11 | | 12 | |
| **样本名称** | | | A1 | | | A2 | A3 | | | | B1 | | | | B2 | | | | B3 | | | |  | | | |  | | | |  | | |  | | |  | |  | |
| **定量结果** | **浓度(μg/μl)** | | 11.6 | | | 12.0 | 10.1 | | | | 13.8 | | | | 11.9 | | | | 11.5 | | | |  | | | |  | | | |  | | |  | | |  | |  | |
|  | **体积(μl)** | | 180 | | | 180 | 180 | | | | 180 | | | | 180 | | | | 180 | | | |  | | | |  | | | |  | | |  | | |  | |  | |
|  | **总量(μg)** | | 2088 | | | 2160 | 1818 | | | | 2484 | | | | 2142 | | | | 2070 | | | |  | | | |  | | | |  | | |  | | |  | |  | |
|  | **样品评价** | | a | | | a | a | | | | a | | | | a | | | | a | | | |  | | | |  | | | |  | | |  | | |  | |  | |
| **质谱分析**  **结果** | Database 1 | | Uniprot_MusMusculus_84433_20180123 | | | | | | | | | | | | | | | | | | | | | | | | | | | | | | | | | | | | | |
|  | Protein group | | 2323 | | | 2212 | 2175 | | | | 2286 | | | | 2254 | | | | 2257 | | | |  | | | |  | | | |  | | |  | | |  | |  | |
|  | Protein group  (Unique Peptide≥2) | | 1629 | | | 1570 | 1516 | | | | 1615 | | | | 1556 | | | | 1572 | | | |  | | | |  | | | |  | | |  | | |  | |  | |
|  | Database 2 | | (如有多次查库结果请罗列) | | | | | | | | | | | | | | | | | | | | | | | | | | | | | | | | | | | | | |
|  | Protein group | |  | | |  |  | | | |  | | | |  | | | |  | | | |  | | | |  | | | |  | | |  | | |  | |  | |
|  | Protein group  (Unique Peptide≥2) | |  | | |  |  | | | |  | | | |  | | | |  | | | |  | | | |  | | | |  | | |  | | |  | |  | |
|  | **质谱评价** | | A | | | A | A | | | | A | | | | A | | | | A | | | |  | | | |  | | | |  | | |  | | |  | |  | |
| **报告结论：1.定量以及SDS-PAGE结果显示蛋白质质量好，总量足够，且样本间平行性较好。**  **2.蛋白预质谱显示酶解正常，色谱质谱行为正常。** | | | | | | | | | | | | | | | | | | | | | | | | | | | | | | | | | | | | | | | | |
| **检测图谱和附件** | | | | | | | | | | | | | | | | | | | | | | | | | | | | | | | | | | | | | | | | |
| **SDS-PAGE电泳图谱** | | | | | | | | | | | | | | | | | | | | | | | | | | | | | | | | | | | | | | | | |
|  | | | | | | | | | | | | | | | | | | | | | | | | | | | | | | | | | | | | | | | | |
| **质谱分析Basepeak图谱** | | | | | | | | | | | | | | | | | | | | | | | | | | | | | | | | | | | | | | | | |
|  | | | | | | | | | | | | | | | | | | | | | | | | | | | | | | | | | | | | | | | | |
| **电子版附表** | | | | | | | | | | | | | | | | | | | | | | | | | | | | | | | | | | | | | | | | |
| 蛋白质鉴定简表 6套；肽段鉴定简表6套 | | | | | | | | | | | | | | | | | | | | | | | | | | | | | | | | | | | | | | | | |
| 结果说明： | | | | | | | | | | | | | | | | | | | | | | | | | | | | | | | | | | | | | | | | |
| （1）样品评价 | | | | | | | | | | | | | | | | | | | | | | | | | | | | | | | | | | | | | | | | |
| a. 质量满足实验要求（电泳条带清晰），且总量满足2次或者2次以上实验 | | | | | | | | | | | | | | | | | | | | | | | | | | | | | | | | | | | | | | | | |
| b. 质量满足实验要求（电泳条带清晰），且总量满足1次不足2次以上实验 | | | | | | | | | | | | | | | | | | | | | | | | | | | | | | | | | | | | | | | | |
| c. 质量不完全满足实验要求（电泳条带模糊，可能降解），存在实验风险 | | | | | | | | | | | | | | | | | | | | | | | | | | | | | | | | | | | | | | | | |
| d. 质量满足实验要求（电泳条带清晰），但总量不满足1次实验，建议客户重新送样 | | | | | | | | | | | | | | | | | | | | | | | | | | | | | | | | | | | | | | | | |
| e. 质量不满足实验要求（几乎未抽提到蛋白质），不建议进行后续实验 | | | | | | | | | | | | | | | | | | | | | | | | | | | | | | | | | | | | | | | | |
| （2）质谱评价 | | | | | | | | | | | | | | | | | | | | | | | | | | | | | | | | | | | | | | | | |
| A. 组内和组间的所有样本平行性好 | | | | | | | | | | | | | | | | | | | | | | | | | | | | | | | | | | | | | | | | |
| B. 组内平行性好，组间平行性较差(lablefree项目对该项可以不考虑) | | | | | | | | | | | | | | | | | | | | | | | | | | | | | | | | | | | | | | | | |
| C. 组内的部分样本不平行，建议重新送样 | | | | | | | | | | | | | | | | | | | | | | | | | | | | | | | | | | | | | | | | |
| **备注** | | | | | | | | | | | | | | | | | | | | | | | | | | | | | | | | | | | | | | | | |
| 检测人员 | |  | | | | | | 核验人员 | | | | | | |  | | | | | | | | | | | | | 技术负责人 | | | | | | |  | | | | | |
|  | | | |  |  | | | |  |  | |  |  |  | |  |  |  | |  |  | | |  |  |  | | |  |  | |  |  | |  |  | |  | |  |

**附录——Basepeak解读说明**

LC-MS/MS分析过程是酶解后的肽段先经过色谱柱进行分离，然后串联进入质谱检测器中进行一级和二级质谱分析。该方法适用于复杂样品的分析，可以增加低丰度蛋白质被鉴定出的几率。Basepeak图是在该过程中将每个时间点质谱检测信号最强的肽段的强度值连续描绘得到的图谱。如下图所示，横坐标为肽段在色谱中的保留时间，纵坐标为质谱信号强度，主要峰上的数字标记分别为信号峰强度最高的肽段的保留时间和质荷比。Basepeak图主要反应样品的色谱分离度、肽段信号强度（可以结合上样体积判断样品酶解情况）、样品中蛋白质的构成复杂程度等。如果从色谱图上看到在不同时间洗脱的峰较多，而且相对丰度较高，则说明样品中的肽段种类较多，复杂程度较高。另一方面，可以通过比较不同样品信号峰的分布情况以及质谱信号强度的相似度，来判断样品酶解后肽段的平行性，从而为各样品间平行性的判断提供依据。
